# Structural and Functional Siderophore Remodeling by Enzymatic Delipidation

**DOI:** 10.1002/anie.2621546

**Published:** 2026-07-24

**Authors:** Elena Herzog, Keishi Ishida, Evelyn M. Molloy, Ron Hermenau, Kirstin Scherlach, Christian Hertweck

**Affiliations:** ^1^ Dept. of Biomolecular Chemistry Leibniz Institute for Natural Product Research and Infection Biology Jena Germany; ^2^ Institute of Microbiology Faculty of Biological Sciences Friedrich Schiller University Jena Jena Germany; ^3^ Cluster of Excellence Balance of the Microverse Friedrich Schiller University Jena Jena Germany

**Keywords:** bacteria, biosynthesis, diazeniumdiolate, natural products, peptides

## Abstract

Bacteria employ specialized metabolites called siderophores to acquire scarce iron, but these molecules may serve additional ecological roles. Here, we reveal that *Pandoraea* species, including environmental isolates and opportunistic pathogens typically acquired from the environment, produce bifunctional lipopeptides that undergo enzymatic remodeling to switch from promoting bacterial motility to optimizing iron capture. Through genome mining and metabolic profiling, we discovered pandorachelins, diazeniumdiolate‐containing siderophores. Comprehensive NMR analysis, derivatization, and isotope labeling established that pandorachelin A is a head‐to‐tail‐fused homodetic cyclopeptide, revising a recently proposed structure. We identified the elusive biosynthetic precursor, pandorachelin B, as a lipocyclopeptide with a lactone moiety and N‐terminal fatty acid. A specialized acylase (PdnM) cleaves the lipid tail of pandorachelin B, triggering an O→N acyl shift that contracts the ring and transforms the biological function: the lipopeptide enables bacterial swarming through surfactant activity, while the delipidated product exhibits enhanced iron‐chelating capacity but no motility promotion. Genetic knockouts, enzyme reconstitution, and phenotypic assays confirm this maturation sequence. The functional switch correlates with ecological niche across *Pandoraea* species, revealing a sophisticated strategy for niche colonization and nutrient acquisition. These findings identify PdnM as a potential antivirulence target and expand the functional repertoire of bacterial siderophores.

## Graphical abstract

A surprising functional switch in bacterial siderophores was discovered through genome mining and the analysis of environmental and pathogenic *Pandoraea* species. Pandorachelin B, a lipocyclopeptide that promotes swarming motility, is cleaved by a specialized acylase, yielding a ring‐contracted, homodetic cyclopeptide, pandorachelin A, which exhibits enhanced iron‐chelating capacity but does not promote motility.

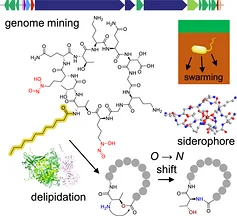

## 1 Introduction

Iron is central to the fundamental cellular processes of living organisms. Yet, under aerobic, physiological conditions, the ferric form (Fe^3^^+^) predominates and is barely soluble or tightly bound to biomolecules. To cope with this persistent limitation, environmental and pathogenic microorganisms have evolved advanced molecular tactics. A widespread strategy is the secretion of siderophores, specialized metabolites with multidentate chelating groups that capture ferric iron with exceptionally high affinity [1]. The resulting soluble iron‐siderophore complex is imported into the cells by specific receptor systems, and the iron is liberated. Siderophore production and uptake plays key roles in highly diverse niches, within microbiomes, and at the pathogen‐host interface [2]. Such microbial interactions are considered the evolutionary playground in which non‐classical siderophore functions have emerged [3]. For example, bacteria may harness siderophores to acquire non‐iron metal ions such as copper [4, 5, 6, 7], to cope with metal‐ and ROS‐induced stress, and to modulate metal‐dependent host immune responses [8]. Furthermore, siderophores can act as antimicrobials keeping competitors at bay [9]. The most elaborate examples of this are natural siderophore‐antibiotic conjugates that function as Trojan horses [10, 11]. Remarkably, there are even siderophores that can trap and release bacterial signaling molecules [12].

Non‐classical siderophore functions may also be conferred by chelating groups that differ from the typical catecholate, α‐hydroxy carboxylate, and hydroxamate residues [1]. Our discovery of gramibactin, a novel siderophore produced by the rhizosphere bacterium *Paraburkholderia graminis* [13], expanded the repertoire of known chelators in siderophores to the bidentate diazeniumdiolate (*N*‐nitroso‐hydroxylamine) ligand, which is extremely rare in nature (Figure S1) [14, 15]. In addition to efficiently coordinating Fe(III) [13, 16], we found that the diazeniumdiolate group of the amino acid building block graminine (Gra) is a nitric oxide (NO) donor, similar to NONOates in pharmaceutical products [17]. In the plant context, NO has been implicated in iron homeostasis, fitness, root growth and stress tolerance. Intriguingly, in vitro assays and in vivo monitoring experiments revealed that the iron chelators also liberate NO in plant roots [18]. Besides enzymatic cleavage leading to NO release, photoreactivity of the diazeniumdiolate induces NO formation, which may come into play in aquatic environments [19]. Indeed, genome mining based on genes for diazeniumdiolate biosynthesis indicated that such unusual siderophores are more prevalent in nature than previously assumed [18].

**FIGURE 1:**
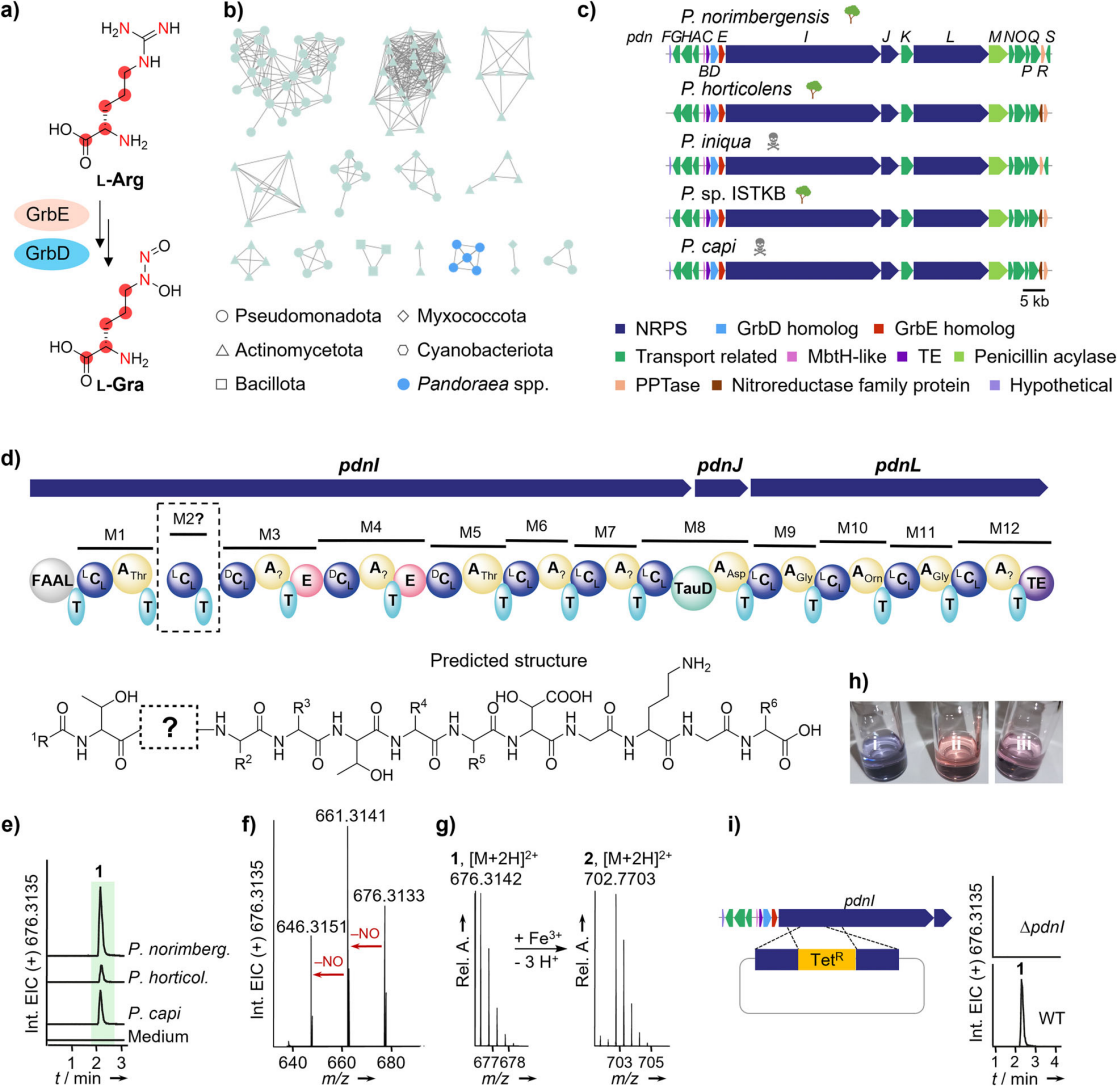
Genome mining for diazeniumdiolate siderophores in the genomes of *Pandoraea* species. (a) Biosynthetic conversion of l‐Arg to l‐Gra by GrbD and GrbE. (b) Sequence similarity network analysis of GrbD homologs, distinguished by phylum. Homologous from *Pandoraea strains* are colored blue. (c) Representation of the highly conserved BGCs of *Pandoraea* spp. TE, thioesterase type II; PPTase, 4'‐phosphopantetheinyl transferase. Skull indicates clinical isolate, tree indicates environmental isolate. (d) Model of the *pdn* NRPS assembly line with predicted substrates accepted by the adenylation domains and structure prediction with R^1^ for unknown fatty acid chain. FAAL, fatty acyl‐AMP ligase domain; C, condensation domain; T, thiolation domain; A, adenylation domain; E, epimerization domain; TauD, l‐aspartate‐*β*‐hydroxylase domain, TE, thioesterase domain, and R^2^−R^6^ [5] for unknown amino acids. (e) HPLC‐HRMS (ESI+) (EIC, extracted ion chromatogram, *m/z* 676.3135 ± 0.5 ppm, NL: 3.50 E8) profiles of the *P. capi*, *P. horticolens*, *P. norimbergensis*, and medium extracts. ESI, electrospray ionization; NL, normalized intensity level. (f) HR‐ESI‐MS/MS (ESI^+^, hcd 10) spectra of pandorachelin A (17) at *m/z* 676.3135 [M+2H]^2+^ revealing NO losses characteristic for the presence of diazeniumdiolate groups. Hcd, higher‐energy collisional dissociation. (g) Isotope pattern of pandorachelin A (1) (found *m/z* 676.3142 [M + 2H]^2+^) and the corresponding iron complex 2 (found *m/z* 702.7703 [M + 2H]^2+^). (h) Liquid CAS assay results with (left) water, (middle) pandorachelin A (1), and (right) EDTA after 20 min. (i) Representation of P. norimbergensis Δ*pdnI* mutant construction strategy by double crossover with a tetracycline resistance gene. (j) HPLC‐HRMS (ESI+) (EIC, extracted ion chromatogram, *m/z* 676.3135 ± 0.5 ppm) profiles of *P. norimbergensis* wild type (WT) and *P. norimbergensis* Δ*pdnI* extracts.

Gramibactin assembly involves a non‐ribosomal peptide synthetase (NRPS) that incorporates the unusual diazeniumdiolate‐substituted amino acid Gra, which is produced by two proteins GrbD and GrbE (Figure 1a) [18, 19]. Homology searches using the cognate genes from the gramibactin (*grb*) biosynthesis gene cluster (BGC) suggested that various bacterial phyla harbor related BGCs, in particular Gram‐negatives [18, 20, 21, 22, 23]. Given their importance in various microbial interactions, including those involving pathogens, we sought to identify similar siderophore systems in clinically relevant bacteria. Indeed, during our survey of *grb*‐like gene loci, we noted that members of the genus *Pandoraea* may possess the genetic capacity to produce diazeniumdiolate siderophores [18]. Named after the mythological figure Pandora, these Gram‐negative bacteria are widespread, opportunistic pathogens that can be acquired from the environment. *Pandoraea* species pose a severe health threat as they have been increasingly observed to infect immunocompromised patients and those with cystic fibrosis. They are alarmingly resistant to antibiotic treatment [24, 25]. Thus, identifying specialized metabolites such as siderophores that promote niche colonization and capture ferric iron from the host is important for setting the basis for therapeutic intervention. This point is underscored by our recent discovery that antibacterial siderophores (pandorabactins) produced by *Pandoraea* pathogens affect the lung microbiota of cystic fibrosis patients [9]. However, there is limited information on the biosynthetic potential of *Pandoraea* species, and the nature of the predicted diazeniumdiolate siderophores remains enigmatic.

Here, we report that a distinct group of *Pandoraea* strains, including environmental and clinical isolates, contain a BGC that is linked to the production of a family of diazeniumdiolate siderophores. We fully elucidate the structures of pandorachelin A and B, a matured congener and the immediate product of the assembly line, respectively. We demonstrate that a designated acylase is required to promote a unique delipidation/ring contraction sequence. Finally, we show that this structural transformation of the lipopeptide influences essential functions of the producer with respect to host colonization and iron acquisition, thereby expanding the functional catalogue of non‐canonical siderophores to include swarming motility promotion.

## 2 Results and Discussion

### 2.1 A Conserved Gene Locus for the Biosynthesis of Diazeniumdiolate Siderophores in *Pandoraea* spp

To investigate the prevalence of potential diazeniumdiolate siderophore BGCs in the genomes of Pandoraea strains, we used the GrbD amino acid sequence and generated a sequence similarity network (SSN) by BLAST analysis and the EFI‐EST tool [26, 27] (Figures 1b and S2). We used these data to create a genome neighborhood with the EFI‐GNT tool [26, 27] and identified a BGC, which we named *pdn* (Figure 1c), that is present in the genomes of five *Pandoraea* species. In addition to P. norimbergensis, which was isolated from lake sediments, this clade comprises the soil‐dwelling xenobiont degraders *Pandoraea horticolens* and *Pandoraea iniqua* [28], a yet taxonomically unidentified *Pandoraea* species from the rhizosphere of trees growing in Indian backwaters [29, 30], and *Pandoraea capi*, which was isolated from the sputum of a pneumonia patient in the USA (Table S1) [28]. Interestingly, the pandorabactin BGC is absent from the genomes of these five *Pandoraea* species harboring the *pdn* BGC [9]. Thus, it appears that these gene loci are mutually exclusive.

All *pdn* gene loci are highly conserved in the five strains and consist of genes for ABC transporters (PdnH, PdnN, PdnP, PdnQ), a permease (PdnO), outer membrane efflux pumps (PdnG, PdnA), and TonB receptors (PdnK, PdnS) that are characteristic for siderophore release and uptake [31, 32]. The *pdn* BGCs contain genes that encode a multimodular NRPS assembly line (PdnI, PdnJ, PdnL) and accessory components such as a phosphopantetheinyl transferase (PdnR) and a thioesterase (PdnC). A bioinformatic analysis by antiSMASH [33] indicated that the NRPS deviates from textbook examples since one module is split (module 8), and one module lacks an adenylation domain (module 2). According to the colinearity rule, one would predict that the NRPS product is a peptide composed of eleven or twelve amino acids, depending on the function of the incomplete second module. The deduced adenylation domain specificities predicted the incorporation of Thr, Asp, Gly, and Orn building blocks (Figure 1d, Tables S3−S5). The presence of a TauD domain in module 8 suggested that the aspartate is hydroxylated. The occurrence of genes (*pdnD* and *pdnE*), coding for variants of GrbD and GrbE that are essential for Gra biosynthesis [18], implied that at least one diazeniumdiolate‐substituted amino acid would be incorporated into the peptide backbone. Furthermore, since the first NRPS module harbors a fatty acyl‐AMP ligase (FAAL) domain [34], we assumed that a fatty acid is attached to the N‐terminus of the peptide (Figure 1d).

To test whether strains harboring the *pdn* gene locus produce diazeniumdiolate‐bearing siderophores, we first subjected *P. norimbergensis* to metabolic profiling. Using high‐performance liquid chromatography with diode array detection coupled to high‐resolution mass spectrometry (HPLC‐DAD‐HRMS), we analyzed the extracts of *P. norimbergensis* cultivated under different conditions. We detected a metabolite (**1**, named pandorachelin A) with an *m/z* 676.3142 [M + 2H]^2+^ that falls within the estimated mass range of the predicted peptide (Figure 1e). MS/MS fragmentation of **1** showed the sequential loss of two NO units, which is a hallmark of diazeniumdiolate residues (Figure 1f). We found that **1** is produced most abundantly in minimal medium alongside a metabolite **2** of *m/z* of 702.7703 [M + 2H]^2+^. Its mass shift compared to **1** and isotopic pattern indicated the presence of iron, signifying that **2** likely represents the iron complex of **1** (Figure 1g). A positive reaction of **1** in the chrome azurol S (CAS) assay [35] provided additional evidence of its function as a siderophore (Figures 1h and S4).

We sought to determine whether pandorachelin A (**1**) originates from the *pdn* BGC by generating a targeted mutant with an impaired NRPS. Therefore, we used a counter‐selection approach with a modified *pheS* system [18, 36] (Table S6−S8) to insert a tetracycline resistance gene into the NRPS‐encoding *pdnI* gene of *P. norimbergensis* (Figures 1i and S5). Examination of the metabolic profile of *P. norimbergensis* Δ*pdnI* showed that pandorachelin A (**1**) production is abrogated, thus linking the *pdn* BGC to the biosynthesis of **1** (Figure 1i). Two additional strains with the *pdn* genotype, namely *P. horticolens* LMG 31112 and *P. capi* LMG 20602 (Figure 1e), were demonstrated by metabolic profiling and subsequent MS/MS fragmentation analyses to produce pandorachelin A (**1**) (Figures S6 and S7).

### 2.2 Pandorachelin A is a Head‐to‐Tail‐Fused, Homodetic Cyclopeptide

**FIGURE 2:**
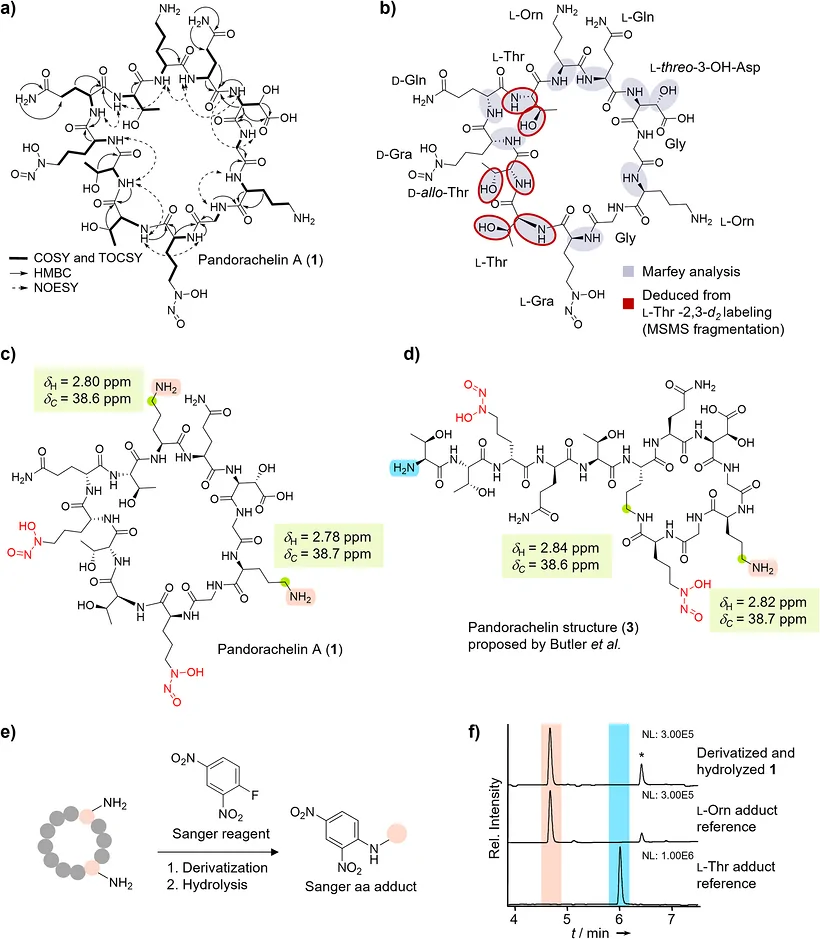
Structure elucidation of pandorachelin A. (a) Planar structure of pandorachelin A (**1**) analyzed by NMR with key correlations of ^1^H‐^1^H‐COSY, ^1^H‐^1^H‐TOCSY ^1^H‐^13^C‐HMBC and ^1^H‐^1^H‐NOESY. (b) Absolute configuration of pandorachelin A (**1**), analyzed by the Marfey method (violet) and isotope labeling experiments (green). (c) Structure of pandorachelin A (**1**) with highlighted chemical shifts of the chemically equivalent l‐Orn side chains. (d) Alternative structure of pandorachelin (**3**) proposed by Butler et al., with highlighted chemical shifts of the l‐Orn side chains. (e) Scheme of pandorachelin A (**1**) derivatization with Sanger's reagent followed by acidic hydrolysis to obtain modified amino acid (aa) fragments. (f) HPLC profiles (340 nm) of hydrolyzed **1**, and authentic references, Sanger adducts of l‐Orn and l‐Thr. * Sanger's reagent.

To elucidate the structure of **1**, we extracted a culture of *P. norimbergensis* with XAD‐16 resin. Purification by preparative reversed‐phase HPLC afforded 10.8 mg/L of pandorachelin A (**1**). Based on ^1^H‐ and ^13^C‐NMR spectra, we identified 18 protons with chemical shifts characteristic of amide protons (6.82–8.25 ppm) and 15 carbonyl carbons (168.7–174.1 ppm). ^1^H‐^1^H‐TOCSY and ^1^H‐^1^H‐COSY spectra showed three spin systems corresponding to threonines. We detected signals for two glycine residues and two closely related spin systems, which we assigned to ornithines, with four protons resonating in the amide proton range that could be associated with the side chains. The spin systems of two other amino acids resembled those of ornithines, but the distinct chemical shifts of two δ‐methylene groups (*δ*_C_ = 61.4 and 61.5 ppm; *δ*_H_ = 4.05 and 4.06 ppm) indicated the presence of two graminine residues. The overlapping protons of two ornithine and two graminine residues were assigned by 1D‐TOCSY and ^1^H‐^13^C‐HSQC‐TOCSY analyses (Figures S9 and S63). Glutamine residues were identified by two couples of ^1^H‐^13^C‐HMBC by correlations from amide protons (*δ*_H_ = 6.86 or 7.31 ppm) to γ‐carbonyl carbon (*δ*_C_ = 174.0 ppm), and amide protons (*δ*_H_ = 6.82 or 7.23 ppm) to γ‐carbonyl carbon (*δ*_C_ = 174.1 ppm). The remaining spin system was assigned to β‐hydroxyaspartate (3‐OH‐Asp) by the presence of a hydroxymethine at the β‐position (*δ*_H_ = 4.51, *δ*_C_ = 70.4 ppm). The 12 spin systems were connected using ^1^H‐^13^C‐HMBC and ^1^H‐^1^H‐NOESY data, revealing a cyclic peptide linked through an amide bond formed by the amino and carboxy termini of the dodecapeptide (Figure 2a, Table S9, Figures S8−S11, S56–S66). The proposed structure was supported by the MS/MS fragmentation pattern of **1** (Figures S12−S14).

While this manuscript was in preparation, the discovery of a diazeniumdiolate siderophore (pandorachelin) from *P. norimbergensis* was reported independently by Butler et al. in a study focusing on the photoreactivity of diazeniumdiolates [37]. The NMR data (^1^H, ^13^C, and HSQC) comparison between **1** and **3** (from Butler's group) reveals that the ^13^C‐NMR and ^1^H‐^13^C‐HSQC data in particular are highly similar (Figure S66 and Table S10). Yet, the proposed structure **3** (Figure 2d) differs from our data interpretation with respect to the absolute configuration and the ring topology, as elaborated below. We determined the absolute configuration of **1** by derivatizing its hydrolysis products with Marfey's reagent, followed by HPLC‐HRMS analysis, and bioinformatic analyses (Table S11, Figure S15). Both ornithine residues were found to be of the l‐configuration. The 3‐OH‐Asp was identified as l‐*threo*‐3‐OH‐Asp. We detected both enantiomers of glutamine and graminine. Labeling experiments with deuterated l‐glutamine and l‐arginine, the biosynthetic precursor of l‐graminine [19], were not helpful in assigning the positions of enantiomers. Therefore, we employed bioinformatic predictions based on the types of condensation (C) domains and the occurrence of epimerase (E) domains in the glutamine‐ and graminine‐incorporating NRPS modules.

The structure **3** put forward by the Butler group suggests an l‐configuration for all three threonine moieties [37]. In contrast, our Marfey analysis revealed the presence of l‐Thr and d‐*allo*‐Thr. To rule out potential epimerization during acidic hydrolysis, we performed the reaction with deuterated acid and observed no sign of epimerization. The assignment is also in good agreement with the NRPS architecture: Threonine building blocks would be incorporated by modules 1, 2, and 5. The C domain of the third module is predicted to catalyze the peptide bond formation between a d‐configured and an l‐configured amino acid. We propose that the second module introduces l‐threonine, which is subsequently converted into d‐*allo*‐threonine by the epimerization domain of the third module. Interestingly, module 2 is incomplete as it lacks a designated A domain. Since all *pdn* BGCs of *Pandoraea* strains code for NRPS assembly lines with this incomplete module, we rule out the possibility of a genome misassembly. A plausible explanation is that the A domain of module 1 loads Thr onto the PCPs of both module 1 and module 2. Iterative function of A domains has been observed previously [38], making this the most plausible scenario. To unequivocally allocate the positions of the l‐Thr and d‐*allo*‐Thr units, we supplemented the culture with l‐Thr‐2,3‐d_2_, before isolating labeled **1** and subjecting it to MS/MS. The number of deuterium labels in the MS fragments enabled us to determine the precise positions of all threonine residues, which matched the predicted configurations (Figures 2b, S16, and S17). These data provide robust support for our proposed structure (l‐Thr_1_, d‐allo‐Thr_2_, l‐Thr_3_).

Our structural elucidation suggests that **1** is a homodetic cyclopeptide that results from a head‐to‐tail amide bond formation between the N‐terminal l‐threonine and the C‐terminal l‐graminine. The Butler group proposed an alternative amide linkage, involving the side chain amine of ornithine with the carbonyl group of the C‐terminal amino acid [37]. Such ω‐amide bonds are unusual for TE‐mediated chain release and would rather require ATP‐grasp enzymes [39]. Furthermore, experimental support for this proposed structure is lacking; key ^1^H‐^13^C‐HMBC correlations required to confirm the connectivity between every amino acid were not reported in the Butler study. Moreover, the structure is not plausible because NMR shifts of amidated and free ornithine side chains should differ. In contrast, all NMR data display identical ^1^H‐ and ^13^C‐ chemical shifts for both ornithine side chains, particularly at C_δ_, indicating chemically equivalent ornithine side chains (Figure 2c,d).

To verify our proposed structure of a head‐to‐tail fused homodetic cyclopeptide, we derivatized **1** with Sanger's reagent. We then acid‐hydrolyzed the peptide and compared the products with authentic reference compounds (Orn, Thr, and Sanger conjugates) by HPLC‐HRMS. If the cyclopeptide were formed by amide linkage via the Orn side chain, Sanger conjugates of both threonine and ornithine would be expected. However, only the free ornithine side chains could be derivatized in our proposed head‐to‐tail cyclized peptide, since the threonine amine is part of the peptide backbone. Our HPLC‐HRMS analysis revealed only derivatized Orn, which strongly supports our structural assignment (Figure 2e,f). The head‐to‐tail cyclic architecture of pandorachelin A (**1**), combined with the presence of the FAAL domain in the NRPS, suggests that this specialized metabolite may result from post‐assembly maturation of a lipidated precursor.

### 2.3 Discovery of a Cryptic Lipocyclopeptide Progenitor

The discovery of a lipidated progenitor was motivated by the apparent discrepancy between the structure of **1** and the in‐silico structure prediction of a lipo(cyclo)peptide. Specifically, the deduced *pdn* NRPS contains an intact, N‐terminal FAAL domain that is expected to prime the peptide assembly with an activated fatty acid. However, the isolated peptide lacks such a lipid tail. Consequently, we reasoned that **1** may not be the primary product of the assembly line but the result of an enigmatic maturation.

**FIGURE 3:**
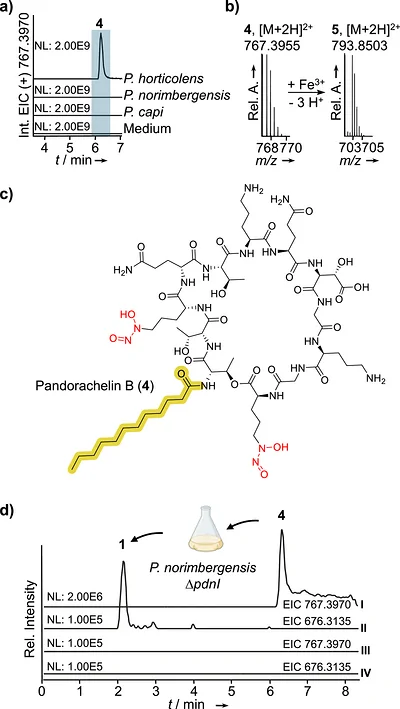
Discovery and structure elucidation of pandorachelin B (**4**). (a) HPLC‐HRMS (ESI+) (EIC, extracted ion chromatogram, *m/z* 767.3970 ± 0.5 ppm) profiles of extracts of *P. horticolens*, *P. norimbergensis* and *P. capi* cultivated in minimal medium, and of extracted minimal medium as negative control. (b) Isotope pattern of pandorachelin B (**4**) (found *m/z* 767.3955 [M + 2H]^2+^) and the corresponding iron complex **5** (found *m/z* 793.8503 [M + 2H]^2+^). (c) Structure of pandorachelin B (**4**), deduced from NMR and MS analyses. (d) HPLC‐HRMS (ESI+) (EIC, extracted ion chromatogram, *m/z* 676.3135 ± 0.5 ppm for 1 and 767.3970 ± 0.5 ppm for **4**) profiles of extracts of *P. norimbergensis* Δ*pdnI* culture supplemented with pandorachelin B (**4**) (traces I and II), and *P. norimbergensis* Δ*pdnI* as negative control (trawith ces III and IV).

To identify potential biosynthetic precursors of **1**, we revisited the metabolic profiles of *Pandoraea* strains. By HPLC‐HRMS we detected a compound **4** with *m/z* 767.3955 [M + 2H]^2+^ in a *P. horticolens* extract (Figure 3a). Based on the related MS/MS fragmentation patterns of **4** and **1** we concluded that **4** is a lipid‐modified congener of **1** and named it pandorachelin B (Figures S18 and S19). We also detected small amounts of a metabolite with *m/z* 793.8503 [M + 2H]^2+^ that corresponds to the Fe complex of **4** (Figure 3b). To obtain sufficient amounts of **4** for structural elucidation, we purified the extract of a *P. horticolens* culture and isolated 7.68 mg L^−1^ of pandorachelin B (**4**) after preparative HPLC. We elucidated the structure of pandorachelin B (**4**) by a series of 1D‐ and 2D‐NMR experiments. These data, together with the MS/MS data, indicated that **4** and **1** possess an identical peptide backbone. However, ^1^H‐^1^H‐TOCSY and ^1^H‐^13^C‐HMBC analysis showed that a dodecanoyl side chain is attached to the amino group of the N‐terminal threonine of **4** (Figures 3c, S20–S24, and S67–S73, and Table S12). In addition, 2D NMR data suggested that **4** differs from **1** by a lactone linkage between the N‐terminal threonine and the l‐Gra moiety, which was supported by the hydrolysis of pandorachelin B (**4**) with a mild base and MS/MS analysis (Figures S25–S27). The discovery of pandorachelin B (**4**) as the lipidated presumed progenitor of pandorachelin A (**1**) reconciles the apparent conflict between the cognate NRPS architecture and the structure of pandorachelin A (**1**) and raises questions about the biosynthetic relationship between the lipopeptide and homodetic cyclopeptide.

### 2.4 Enzymatic Delipidation Remodels Cyclopeptide Architecture

While the dodecapeptide backbones of **1** and **4** are colinear with the deduced pdn NRPS assembly line, their overall structures markedly differ in the cyclization mode (homodetic cyclopeptide vs. lactone) and in the absence or presence of an N‐terminal fatty acid. A plausible biosynthetic model linking **1** and **4** would involve an O→N acyl shift transforming a lactone into a homodetic cyclopeptide. Yet, this rearrangement can only take place when the amino group in the N‐terminal Thr is not acylated. Since the FAAL domains appear identical in the NRPSs of both *P. norimbergensis* and *P. horticolens* (Figure S28), we assumed that lipopeptide biosynthesis is primed with an activated fatty acid and terminated by thioesterase‐catalyzed lactone formation. The subsequent cleavage of the fatty acyl amide bond would liberate the free amino group to initiate the O→N acyl shift, similar to the S→N acyl shift observed in the biosynthesis of autoinducing peptides (AIPs) of Clostridia [40]. Whereas the ester cleavage would require enzyme catalysis, the subsequent rearrangement would occur spontaneously.

**FIGURE 4:**
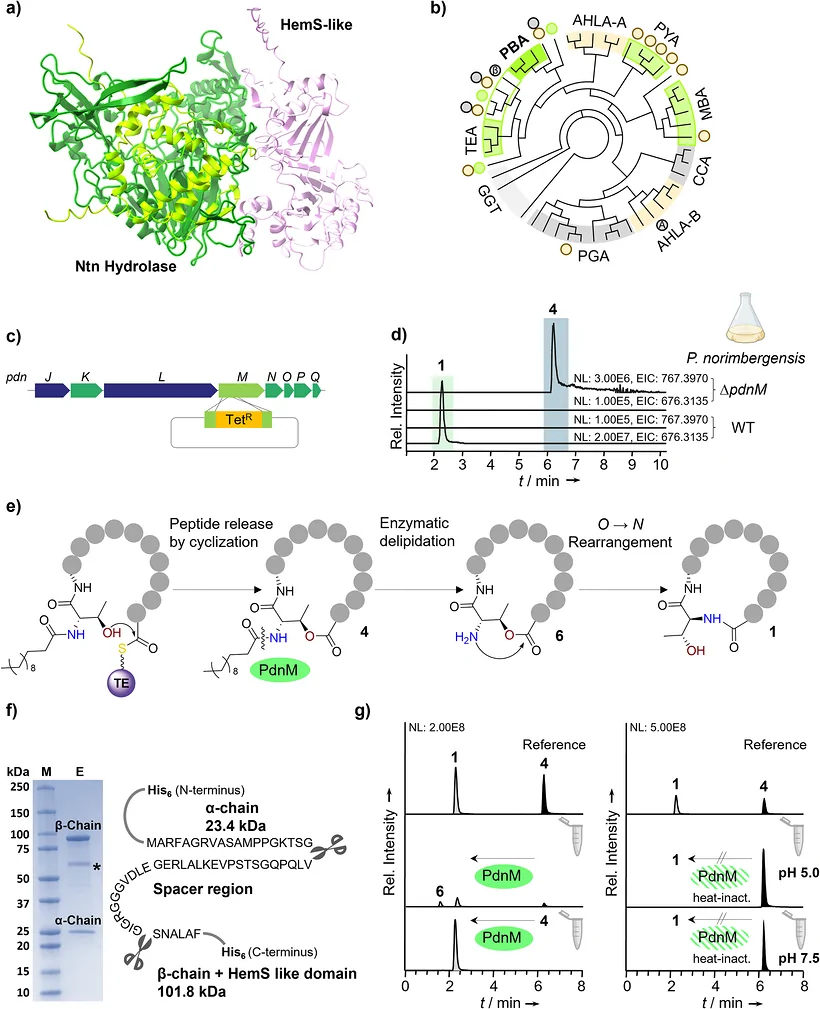
Enzymatic conversion of pandorachelins by delipidation and rearrangement. (a) Structure model of PdnM (AlphaFold3) with α‐chain shown in lime, β‐chain shown in green of Ntn hydrolase, and HemS‐like domain shown in pink. (b) Maximum likelihood phylogenetic tree for PdnM and related acylase enzymes. Color codes indicate similar and/or same substrates. GGT, γ‐glutamyltranspeptidase; PGA, penicillin G acylase; AHLA‐B, acyl homoserine lactone (AHL) acylase type B; CCA, cephalosporin C acylase; MBA, marinobactin acylase; PYA, pyoverdine I precursor acylase; AHLA‐A, AHL acylase type A; PBA, pandorachelin B acylase; TEA, telomycin acylase. Small colored circles show reported acylase activities. “A” and “β” in gray circle indicate ampicillin and β‐lactam, respectively. (c) *P. norimbergensis* Δ*pdnM* mutant construction strategy by double crossover with a tetracycline resistance gene. (d) HPLC‐HRMS (ESI+) (EIC, extracted ion chromatogram, 767.3970 ± 0.5 ppm for **4** and *m/z* 676.3135 ± 0.5 ppm for **1**) profiles of *P. norimbergensis* Δ*pdnM* and wild‐type (WT). (e) Model for cyclopeptide formation by TE‐mediated lactone formation to yield **4**, and fatty acid cleavage by PdnM, followed by an O→*N* acyl shift to give **1**. (f) SDS‐PAGE analysis of purified PdnM‐cHis_6_ (E: 250 mm imidazole‐containing buffer) α‐ and β‐chains from *E. coli* Rosetta2 (DE3) pET26‐*pdnM*. Asterisk indicates degraded PdnM‐His_6_ (β‐chain); (M: molecular marker) with proposed cleavage sites and molecular weights of proteins obtained by PdnM autoproteolysis. (g) In vitro assay of PdnM‐catalyzed biotransformation at pH 5.0 and 7.5. HPLC‐MS (ESI+), EIC, *m/z* 767.3970 ± 0.5 ppm (black) for **4** and *m/z* 676.3135 ± 0.5 ppm (grey) for **1** and **6**.

To test this model, we performed a series of biotransformation experiments and functional studies. First, we added the lipocyclopeptide **4** to cultures of the *P. norimbergensis* Δ*pdnI* mutant that lacks an intact NRPS. The HPLC‐HRMS analysis of culture extracts showed the conversion of **4** into the delipidated cyclopeptide **1** (Figure 3d). Consequently, the lipopeptide may be transformed enzymatically. Examination of the *pdn* BGCs revealed a gene (*pdnM*) tentatively encoding an enzyme distantly related to penicillin acylases, which could be a candidate biocatalyst mediating the fatty acid cleavage. The encoded enzyme, PdnM, appears to be highly specialized, as BLASTp identified only twelve homologous enzymes, most of which are encoded in *Pandoraea* genomes (Table S13). A structure prediction (AlphaFold3) [41] suggests that PdnM consists of two domains (Figure 4a): an N‐terminal domain that is highly similar to the N‐terminal nucleophile (NTN) hydrolase family with a typical αββα‐sandwich core architecture (NTN hydrolase fold) [42], and a C‐terminal HemS‐like domain with similarity to heme degrading/trafficking enzymes of pathogenic bacteria [43]. The amino acid sequence of the NTN‐like domain shares features with PvdQ, a signal peptide sequence of a periplasmic protein (Figure S29), six cysteine residues for three disulfide bonds, and a Ser‐His‐Asp catalytic triad of serine proteases (Figure S30) [44]. A phylogenetic analysis of PdnM‐type enzymes and NTN hydrolase family acylases shows that pandorachelin B acylases form a distinct clade. Overall, the cladogram consists of two major groups: (I) penicillin G acylase (PGA) [45], cephalosporin C acylase (CCA) [46], and quorum‐quenching acyl homoserine (AHL) acylases [47], and (II) lipopeptide acylases, such as pyoverdin I precursor acylase (PvdQ) [48], marinobactin hydrolase (BntA, MhtA) [49], telomycin precursor acylase (Tem25) [50], and aculeacin acylase (AAC) [51] (Figures 4b and S31 and S32, Table S14). The phylogenetic distinctness of PdnM and its restriction to *Pandoraea* genomes hints this acylase has evolved specifically for pandorachelin maturation, rather than being a promiscuous housekeeping enzyme.

To verify whether PdnM is responsible for the cleavage of pandorachelin B (**4**), we generated a *pdnM*‐deficient mutant. To achieve this goal, we inserted a tetracycline resistance cassette by homologous double crossover, similar to the protocol for generating *P. norimbergensis* Δ*pdnI*. The successful construction of *P. norimbergensis* Δ*pdnM* was confirmed by PCR (Figures 4c and S33, Table S7 and S8). Metabolic profiling of *P. norimbergensis* Δ*pdnM* revealed the exclusive production of pandorachelin B (**4**), with no detectable pandorachelin A (**1**) (Figure 4d). These findings support a biosynthetic model in which the *pan* NRPS assembles an acylated dodecapeptide, which is released by a thioesterase‐mediated lactone formation. After cyclization, PdnM cleaves the fatty acid to yield an intermediate pandorachelin A lactone (**6**), and the liberated amino group initiates an O→N acyl shift to produce the homodetic cyclopeptide **1** (Figure 4e).

To test whether PdnM alone is sufficient for the deacylation and rearrangement of pandorachelin B (**4**), we performed in vitro activity experiments using recombinant PdnM. Considering that PdnM is a periplasmic protein, we removed the signal peptide‐encoding gene fragment (Figures 4f and S29). Furthermore, due to an autoproteolytic feature of PdnM, we cloned *pdnM* into the pET26b vector with N‐ and C‐terminus His_6_ tags and heterologously expressed it in *Escherichia coli*. Periplasmic lysates of *E. coli* cultured at 16°C were subjected to Ni‐NTA purification, yielding mature PdnM‐cHis_6_ as a heterodimer of α‐ and β‐chains that result from autoproteolysis (Figures 4f and S34−S36) [52, 53, 54]. We performed the pandorachelin B (**4**) deacylation activity assay using ∼100 nm of mature PdnM and 100 µm of pandorachelin B (**4**) in 50 mm HEPES buffer at pH 7.5. HPLC‐HRMS analysis of the enzymatic reaction mixture revealed that PdnM completely deacylates pandorachelin B (**4**) to yield exclusively pandorachelin A (**1**) (Figure 4g).

To clarify whether the O→N shift is enzyme‐catalyzed or a spontaneous process that occurs after the delipidation, we sought to dissect both steps. In organic synthesis, the mechanistically related O‐acyl isopeptide method for preparing difficult sequence‐containing peptides, has been reported to be pH‐dependent. The O→N‐intramolecular rearrangement takes place rapidly at a neutral pH of 7.4, very slowly at a pH of 4.9, with a half‐life time of ∼3 h, and is stalled at a pH of 2.0 [55]. By analogy, we assumed that an intermediary pandorachelin A lactone (**6**) would be detectable at lower pH if PdnM's primary role were delipidation. To test this, we performed the enzyme assay with PdnM at various pH levels and monitored the product profiles by LC‐HRMS (Figures 4g and S37). We found that PdnM can hydrolyze pandorachelin B (**4**) across the entire pH range (5.0 to 8.0). Furthermore, we detected pandorachelin A lactone (**6**) in the assay mixture at pH 5.0, where the lactone form is stabilized (Figures 4g and S38−S44). However, we also detected a comparable amount of pandorachelin A (**1**), which was unexpected given the short time frame of the reaction (0.5 h). While it seems that the primary function of PdnM is the delipidation of pandorachelin B (**4**), this finding points to an auxiliary role of PdnM in the subsequent O→N‐rearrangement of the resulting pandorachelin A lactone (**6**), a process that also occurs spontaneously under physiological conditions.

Taken together, our phylogenetic and functional analyses reveal that PdnM represents a new type of lipopeptide acylase, which differs from previously known enzymes of this class [56]. The experiments demonstrate that PdnM is essential and sufficient for cyclopeptide delipidation, setting the stage for an O→N acyl migration to produce a ring‐contracted, homodetic peptide.

### 2.5 The Structural Switch Impacts Swarming and Siderophore Functions

**FIGURE 5:**
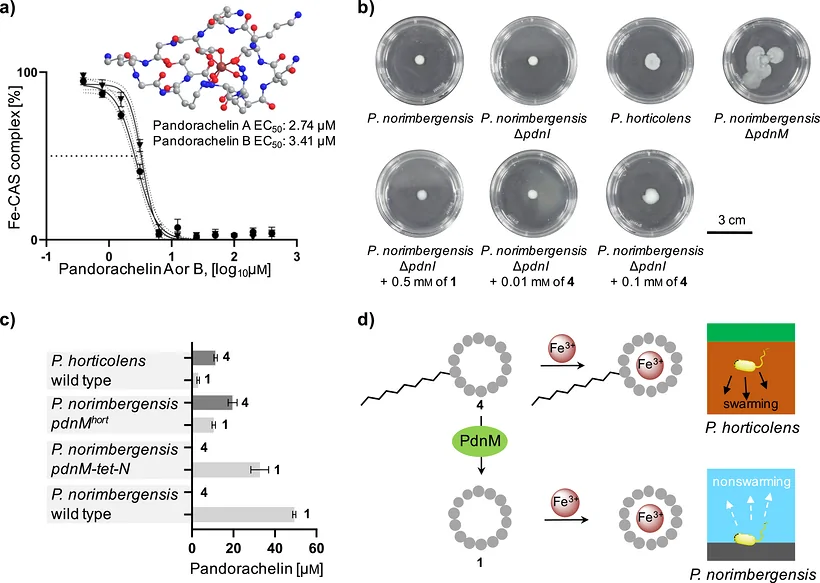
Biological functions of pandorachelins. (a) Quantitative liquid CAS assay analysis with pandorachelin A (**1**) and B (**4**), and the corresponding EC_50_ values. (b) Comparison of swarming motility of wild‐type *P. norimbergensis, P. norimbergensis* Δ*pdnI*, wild‐type *P. horticolens*, and *P. norimbergensis* Δ*pdnM*. *P. norimbergensis* Δ*pdnI* was also exposed to the listed concentrations of **1** or **4** (each 10 µL culture spots on soft agar). (c) Production of pandorachelin A (**1**) and pandorachelin B (**4**) by *P. horticolens* wild type, *P. norimbergensis* *pdnM^hort^*, *P. norimbergensis pdnM‐tet‐N*, and *P. norimbergensis* wild type. The error bars indicate the standard deviation. (d) Plausible adaptive strategy depending on PdnM activity. The soil‐dwelling *P. horticolens* produces predominantly lipopeptide 4, which confers motility by swarming, whereas the aquatic, non‐swarming *P. norimbergensis* produces predominantly the delipidated form with improved iron‐binding.

The remarkable structural remodeling catalyzed by PdnM prompted us to investigate whether it plays a functional role in the bacterial life cycle. We can rule out the possibility of enzymatic cleavage promoting the release of bound iron, as has been shown for specific fungal siderophores or some bacteria [57, 58], since MS data and the CAS assay indicate that both **1** and **4** bind iron. To investigate whether the structural change affected the siderophore activity, we performed a quantitative evaluation of the iron‐binding capacities. By titration experiments using EDTA as positive control, we found that the homodetic cyclopeptide **1** (EC_50_: 2.74 µM) is a slightly more potent siderophore than the lipocyclopeptide precursor **4** (EC_50_: 3.41 µM) (Figure 5a), meaning that the cleavage reaction strengthens binding of iron rather than releasing it.

Since lipopeptides can have biosurfactant properties that facilitate bacterial motility [59], we next examined whether the structural transformation of pandorachelins confers a change in the swarming phenotype. We cultivated *P. norimbergensis* Δ*pdnM* (solely producing the lipopeptide, pandorachelin B (**4**)), the wild type (solely producing the delipidated cyclopeptide, pandorachelin A (**1**)), and *P. norimbergensis* Δ*pdnI* (negative control) on minimal medium soft agar. For comparison, we also tested *P. horticolens* because it produces both compounds. We found that both wild‐type *P. norimbergensis* and *P. norimbergensis* Δ*pdnI* (which both lack lipopeptide **4**) are incapable of swarming. In stark contrast, *P. norimbergensis* Δ*pdnM* shows a pronounced swarming phenotype. In comparison, we observed a slight swarming ability of *P. horticolens* (Figure 5b). HPLC‐HRMS analysis of extracts of the soft agar plates detected only pandorachelin B (**4**) from *P. norimbergensis* Δ*pdnM* and lower amounts from wild‐type *P. horticolens*, which correlates with the strength of their swarming phenotypes (Figure S45).

To directly confirm the effect of pandorachelin B (**4**) on swarming, we performed a chemical complementation assay. Addition of 0.5 mm pandorachelin A (**1**) did not affect the swarming motility of *P. norimbergensis* Δ*pdnI*. In contrast, the phenotype of *P. norimbergensis* Δ*pdnI* supplemented with 0.1 mm pandorachelin B (**4**) was similar to that of *P. horticolens* (Figures 5c). These findings show that bacterial swarming critically depends on the primary product of the biosynthetic pathway, the lipidated pandorachelin (**4**). However, swarming ability is abated upon PdnM‐mediated lipopeptide cleavage and ring contraction. While this processed product **1** has lost its biosurfactant property, it exhibits improved iron‐chelating efficacy (Figure 5d). The correlation between pandorachelin B (**4**) levels and swarming phenotypes across strains supports this functional relationship. The dual function of the pandorachelins could be ecologically relevant and represent an adaptive strategy.

Intriguingly, we noted that the ratio of individual pandorachelins correlates with the producer's habitat: *P. horticolens* (soil) produces both forms (**1** and **4**) and swarms, whereas *P. norimbergensis* (aquatic) produces predominantly the delipidated form and presumably does not require swarming for dispersal. We deemed the acylase PdnM a possible candidate to control these processes, enabling the bacteria to adapt to the specific environmental requirements. To experimentally probe this, we constructed a *pdnM* gene swap mutant, *P. norimbergensis pdnM^hort^*, in which the *pdnM* gene of *P. norimbergensis* was replaced with the homologue of *P. horticolens* (Figure S46). LC‐HRMS profiling showed that this mutant mirrors the *P. horticolens* chemotype, producing a similar ratio of pandorachelin A (**1**) and B (**4**) (Figures 5c and S47−S55, Table S15). To exclude polar effects, we constructed a control strain (*P. norimbergensis pdnM‐tet‐N*), in which a tetracycline resistance gene was inserted downstream of *pdnM* in *P. norimbergensis*. Notably, this mutant shows a similar chemotype to the wild type, exclusively producing pandorachelin A (**1**) (Figures 5c and S47, Table S15). These results suggest that metabolic profiles are not dependent on gene expression levels. Rather, the PdnM variant from *P. horticolens* exhibits lower activity than the PdnM variant from *P. norimbergensis*. This difference has an immediate impact on the chemical mediators that influence niche‐adaptive properties. The mechanisms affecting PdnM activity and the evolutionary trajectories that have shaped pandorachelin profiles across different *Pandoraea* habitats are intriguing avenues for future investigation.

Enzymatic modulations of chemical mediators [60] represent sophisticated adaptive strategies that have emerged in microbial interactions, including predator defense [61, 62], host‐protection by enzymatic lipopeptide cleavage of bacterial virulence and niche factors [63, 64], and a delipidation‐based, chemical radar system to sense and kill microbial predators [65]. Here, we expand this theme to encompass a bifunctional swarming mediator and siderophore. Notably, the ramifications of pandorachelin modulation are diametrically opposed to the consequences of peptide delipidation in pyoverdine biosynthesis in which PvdQ‐mediated removal of the fatty acid tail is a prerequisite to chromophore formation and thus iron chelation [48, 66]. In this case, no swarming motility could be observed for the *pvdQ* deletion strain producing the lipidated pyoverdin precursor [66, 67], likely due to an impairment of iron import [68]. Our findings that swarming of pandorachelin producers depends on the lipopeptide precursor and that delipidation diminishes motility while improving iron binding brings about a new perspective on siderophore maturation and inspires the search for similar roles in related lipopeptide and siderophore biosynthetic pathways.

## 3 Conclusion

In this study, we reveal an unprecedented functional switch in bacterial siderophores: *Pandoraea* species produce a lipopeptide that promotes swarming motility, then enzymatically convert it into a more potent iron chelator. This transformation represents a sophisticated strategy for niche colonization and nutrient acquisition, enhancing our understanding of how bacteria tune specialized metabolites in response to environmental demands. Central to this was our unambiguous structural revision of pandorachelin A (**1**) as a head‐to‐tail‐fused, homodetic cyclopeptide, and the discovery of the elusive lipocyclopeptide progenitor, pandorachelin B (**4**). Our data shed light on an impressive functional switch of these chemical mediators, catalyzed by PdnM as a novel type of lipopeptide acylase.

Our findings reveal that pandorachelins exemplify a sophisticated bacterial strategy: the repurposing of a single metabolite to adapt to ecological niches. The enzymatic alteration of a motility‐promoting lipopeptide to an optimized iron chelator not only broadens the functional repertoire of siderophores but also identifies PdnM as a potential antivirulence target for combating antibiotic‐resistant *Pandoraea* infections. As these opportunistic pathogens increasingly threaten immunocompromised patients, understanding the molecular basis of their colonization and persistence becomes critical. More broadly, this work challenges the traditional view of siderophores as dedicated iron scavengers [3] and suggests that other NRPS‐derived metabolites may harbor similar cryptic dual functions awaiting discovery. The pandorachelin system thus serves as both a compelling example of evolutionary ingenuity and a roadmap for developing innovative therapeutic interventions that exploit the Achilles' heels of bacterial pathogens.

## Author Contributions

**Elena Herzog**: investigation, visualization, formal analysis, methodology, writing – original draft, validation. **Ron Hermenau**: investigation, formal analysis. **Keishi Ishida**: supervision, formal analysis, investigation, validation, conceptualization, methodology, writing – original draft, visualization. **Christian Hertweck**: conceptualization, writing – review and editing, writing – original draft, resources, project administration, visualization, supervision, funding acquisition. **Evelyn M. Molloy**: supervision, writing – review and editing, formal analysis. **Kirstin Scherlach**: formal analysis, supervision, validation.

## Patient/Participant Consent Statement

No patient or participant consent is required.

## Conflicts of Interest

The authors declare no conflicts of interest.

## Supporting information




**Supporting File**: anie73703‐sup‐0001‐SuppMat.pdf.

## Data Availability

The data that supports the findings of this study are available in the Supporting Information of this article.
