# Supplementary material for: Structural and Functional Siderophore Remodeling by Enzymatic Delipidation
Source: Angew Chem Int Ed Engl. 2026 Jul 24;65(39):e2621546. doi: 10.1002/anie.2621546 (PMC13592322; doi:10.1002/anie.2621546)
Supplement: Supplementary file 1 [file ANIE-65-e2621546-s001.pdf]

Table of Contents

Table of Contents 1

Figures 2

Tables 4

General methods 5

Experimental procedures 6

Sequence similarity network (SSN) 6

Bacterial strains and culturing conditions 8

Analysis of the putative *pdn* biosynthesis gene cluster 8

Preparation of MM9 medium 10

Preparation of MM9 medium soft agar 11

Preparation of brain heart infusion broth 11

Preparation of CAA medium 11

Preparation of ABG medium 11

Siderophore production in liquid media by different *Pandoraea* strains 11

Isolation of pandorachelin A (1) 11

Isolation of pandorachelin B (4) 12

CAS assay 12

Generation of *pdnI* mutant of *P. norimbergensis* DSM11628 13

Extraction of *P. norimbergensis* Δ*pdnI* 16

Detection of the compounds 16

Determination of the structure of pandorachelin A (1) 17

Marfey's analysis of pandorachelin A (1) 24

Preparation and isolation of ^{2}H-labeled pandorachelin A (1) 25

MS/MS spectra of pandorachelin A (1) labeled with L-Thr-2,3-d_2 26

Preparation and isolation of ^{15}N-labeled pandorachelin A (1) 26

Sanger's analysis of pandorachelin A (1) 27

Determination of the structure pandorachelin B (4) 27

Basic hydrolysis of pandorachelin B (4) 32

Alignment of the amino sequences of the FAAL domains 34

Biotransformation of pandorachelin B (4) by *P. norimbergensis* Δ*pdnI* 35

Bioinformatic analysis of PdnM 35

Preparation of maximum likelihood phylogenetic tree 39

Generation of *pdnM* mutant of *P. norimbergensis* DSM11628 41

Extraction of *P. norimbergensis* Δ*pdnM* 41

Heterologous production and purification of PdnM (His$_6$-α-chain and β-chain-His$_6$) 41

In-gel tryptic digestion of PdnM 42

Acylase activity assay of PdnM 45

Swarming assay 48

Extraction of the soft agar 49

Generation of *pdnM^{hort}* mutant and its negative control mutant of *P. norimbergensis* DSM11628 50

Extraction and quantitative analysis of pandorachelin production by *P. norimbergensis*, *P. horticolens*, *P. norimbergensis* *pdnM^{hort}*, and *P. norimbergensis pdnM-tet-N* 51

NMR spectra of pandorachelin A (1) 55

NMR spectra of pandorachelin B (4) 60

References 64

Figures

Figure S1. Structure of gramibactin. 6
Figure S2. Complete sequence similarity network of GrbD homologs, annotated with UniProt accession numbers. 7
Figure S3. Putative *pdn* biosynthesis gene cluster in the genome of *Pandoraea norimbergensis* with the locus tag of *pdnI*: ctg1_2484. 8
Figure S4. CAS agar plate with cultivated *P. norimbergensis*. 12
Figure S5. PCR confirmation of *P. norimbergensis* mutants. 16
Figure S6. HR-ESI-MS/MS (ESI$^+$, hcd 25) spectra of pandorachelin A (**1**) at *m/z* 676.3135 $[M+2H]^{2+}$ produced by *P. norimbergensis* (top), *P. horticolens* (middle), and *P. capi* (bottom). 16
Figure S7. HR-ESI-MS/MS (ESI$^+$, hcd 30) spectra of pandorachelin A (**1**) at *m/z* 676.3135 $[M+2H]^{2+}$ produced by *P. norimbergensis* (top), *P. horticolens* (middle), and *P. capi* (bottom). 17
Figure S8. Structure elucidation of pandorachelin A (**1**) with key correlations of ^{1}H-^{1}H-COSY, ^{1}H-^{1}H-TOCSY ^{1}H-^{13}C-HMBC and ^{1}H-^{1}H-NOESY. 17
Figure S9. 1D-TOCSY spectrum of amide proton (L-ornitine 2) pandorachelin A (**1**) in DMSO-d_6 (600 MHz). 22
Figure S10. FTIR spectrum of pandorachelin A (**1**). 22
Figure S11. UV-Vis spectrum of pandorachelin A (**1**). 22
Figure S12. MS/MS (ESI$^+$) fragmentation of pandorachelin A (**1**) 23
Figure S13. HR-ESI-MS/MS (ESI$^+$, hcd 30) spectra of pandorachelin A (**1**) at *m/z* 1351.6201 $[M+H]^+$. Bold ions highlight those shared with the dataset from Butler's group. 23
Figure S14. MS/MS fragmentation pattern of pandorachelin A (**1**). 24
Figure S15. HPLC-HRMS (ESI+) (EIC, extracted ion chromatogram) of pandorachelin A (**1**) hydrolysate and reference compounds. 25
Figure S16. MS/MS (ESI$^+$) fragmentation of ^{2}H-labeled pandorachelin A (**1**) 26
Figure S17. MS/MS fragmentation pattern of ^{2}H-labeled pandorachelin A (**1**). 26
Figure S18. MS/MS (ESI$^+$) fragmentation of pandorachelin B (**4**) 27
Figure S19. MS/MS fragmentation pattern of pandorachelin B (**4**). 28
Figure S20. Structure elucidation of pandorachelin B (**4**) with key correlations of ^{1}H-^{1}H-COSY, ^{1}H-^{1}H-TOCSY, ^{1}H-^{13}C-HMBC and ^{1}H-^{1}H-ROESY. 28
Figure S21. 1D-TOCSY spectrum of amide proton (L-ornitine 1) pandorachelin B (**4**) in DMSO-d_6 (600 MHz). 31

Figure S22. 1D-TOCSY spectrum of amide proton (L-ornitine 2) pandorachelin B (**4**) in DMSO-d_6 (600 MHz). 31
Figure S23. FTIR spectrum of pandorachelin B (**4**). 31
Figure S24. UV-Vis spectrum of pandorachelin B (**4**). 32
Figure S25. HPLC-HRMS (ESI+) (EIC, extracted ion chromatogram) of the hydrolysis of pandorachelin B (**4**) 32
Figure S26. MS/MS (ESI$^+$) fragmentation of linearized pandorachelin B (**4**). 33
Figure S27. MS/MS fragmentation pattern of linearized pandorachelin B (**4**). 33
Figure S28. Alignment of amino acid sequences of the FAAL domain in PdnI from *P. norimbergensis*, *P. horticolens* and *P. capi*. 34
Figure S29. Prediction of signal peptide sequence by SignalP 6.0. 35
Figure S30. Multiple sequence alignment of PdnM and PvdQ. The 37
Figure S31. Function of fatty acid acylases found in bacteria. 38
Figure S32. Maximum likelihood phylogenetic tree for PdnM and related acylase enzymes. 39
Figure S33. PCR confirmation of *P. norimbergensis* Δ*pdnM* mutants. 41
Figure S34. Fingerprint analysis of peptide fragments of tryptic-digested His$_6$-PdnM α-chain. 42
Figure S35. Fingerprint analysis of peptide fragments of tryptic-digested PdnM-His$_6$ β-chain. 43
Figure S36. Fingerprint analysis of peptide fragments of tryptic-digested PdnM-His$_6$ β-chain (degraded, asterisk in Figure 4f). 44
Figure S37. Acylase activity of PdnM demonstrating the conversion of pandorachelin B (**4**) 45
Figure S38. MS/MS (ESI$^+$, *m/z* 676.3135 $[M+2H]^{2+}$ hcd20) fragmentation comparison of pandorachelin A (**1**) and pandorachelin A lactone (**6**). 45
Figure S39. MS/MS (ESI$^+$, *m/z* 676.3135 $[M+2H]^{2+}$ hcd20, *m/z* 100–650) fragmentation comparison of pandorachelin A (**1**) and pandorachelin A lactone (**6**). 46
Figure S40. MS/MS (ESI$^+$, *m/z* 676.3135 $[M+2H]^{2+}$ hcd20, *m/z* 550–1400) fragmentation comparison of pandorachelin A (**1**) and pandorachelin A lactone (**6**). 46
Figure S41. MS/MS (ESI$^+$, *m/z* 676.3135 $[M+2H]^{2+}$ hcd30) fragmentation comparison of pandorachelin A (**1**) and pandorachelin A lactone (**6**). 46
Figure S42. MS/MS (ESI$^+$, *m/z* 676.3135 $[M+2H]^{2+}$ hcd30, *m/z* 100–700) fragmentation comparison of pandorachelin A (**1**) and pandorachelin A lactone (**6**). 47
Figure S43. MS/MS (ESI$^+$, *m/z* 676.3135 $[M+2H]^{2+}$ hcd30, *m/z* 700–1400) fragmentation comparison of pandorachelin A (**1**) and pandorachelin A lactone (**6**). 47
Figure S44. MS/MS fragmentation pattern of linearized pandorachelin A lactone (**6**). 48
Figure S45. HPLC-HRMS (ESI+) (EIC, extracted ion chromatogram) of pandorachelin A (**1**) (*m/z* 676.3135 $[M+2H]^{2+}$) and pandorachelin B (**4**) (*m/z* 767.3970 $[M+2H]^{2+}$) of *P. norimbergensis* wild type, *P. norimbergensis* Δ*pdnM* and *P. norimbergensis* Δ*pdnI* and *P. horticolens* soft agar extracts after 3 days. 49
Figure S46. Generation and PCR confirmation of double-crossover mutants *P. norimbergensis* $pdnM^{hort}$. a) Schematic *pdnM* gene swap in *P. norimbergensis* by double-crossover event. b) PCR confirmation of *P. norimbergensis* $pdnM^{hort}$. 50
Figure S47. Preparation and PCR confirmation of double-crossover mutants *P. norimbergensis pdnM-tet-N*. a) Schematic tet^R gene insertion in *P. norimbergensis* by double-crossover event. b) PCR confirmation of *P. norimbergensis pdnM-tet-N*. 51
Figure S48. RP-HPLC profile of pandorachelin A (**1**) at different concentrations. 51
Figure S49. The standard concentration obtained by RP-HPLC analysis of pandorachelin A (**1**). 52
Figure S50. RP-HPLC profile of pandorachelin B (**4**) at different concentrations. 52
Figure S51. The standard concentration obtained by RP-HPLC analysis of pandorachelin B (**4**). 52
Figure S52. RP-HPLC profile of the XAD16 extracts of *P. norimbergensis* wild-type cultures. **1**: pandorachelin A. 52
Figure S53. RP- HPLC profile of the XAD16 extracts of *P. horticolens* wild-type cultures. **1**: pandorachelin A, **4**: pandorachelin B. 53

Figure S54. RP-HPLC profile of the XAD16 extracts of *P. norimbergensis pdnM^{hort}* cultures. **1**: pandorachelin A, **4**: pandorachelin B. 53
Figure S55. RP-HPLC profile of the XAD16 extracts of *P. norimbergensis pdnM-tet-N* cultures. **1**: pandorachelin A. 53
Figure S56. ^{1}H-NMR spectrum of pandorachelin A (**1**) in DMSO-d_6 (600 MHz). 55
Figure S57. ^{13}C-NMR spectrum of pandorachelin A (**1**) in DMSO-d_6 (150 MHz). 55
Figure S58. ^{13}C-Jmod-NMR spectrum of pandorachelin A (**1**) in DMSO-d_6 (150 MHz, C and CH_2 are positive, CH and CH_3 are negative). 56
Figure S59. ^{1}H-^{13}C-HSQC spectrum of pandorachelin A (**1**) in DMSO-d_6 (600 MHz). 56
Figure S60. ^{1}H-^{13}C-HMBC spectrum of pandorachelin A (**1**) in DMSO-d_6 (600 MHz). 57
Figure S61. ^{1}H-^{1}H-COSY spectrum of pandorachelin A (**1**) in DMSO-d_6 (600 MHz). 57
Figure S62. ^{1}H-^{1}H-TOCSY spectrum of pandorachelin A (**1**) in DMSO-d_6 (600 MHz). 58
Figure S63. ^{1}H-^{13}C-HSQC-TOCSY spectrum of pandorachelin A (**1**) in DMSO-d_6 (500 MHz). 58
Figure S64. ^{1}H-^{1}H-NOESY spectrum of pandorachelin A (**1**) in DMSO-d_6 (600 MHz). 59
Figure S65. ^{1}H-^{15}N-HMBC spectrum of ^{15}-N-labeled pandorachelin A (**1**) in DMSO-d_6/CD_3NO_2 3:1 (500 MHz 59
Figure S66. ^{1}H-^{13}C-HSQC spectrum comparison of pandorachelin A (**1**) in DMSO-d_6 (600 MHz) (blue) and pandorachelin (**3**) from the Bulter group (orange). 60
Figure S67. ^{1}H-NMR spectrum of pandorachelin B (**4**) in DMSO-d_6 (600 MHz). 60
Figure S68. ^{13}C-NMR spectrum of pandorachelin B (**4**) in DMSO-d_6 (150 MHz). 61
Figure S69. ^{1}H-^{13}C-HSQC spectrum of pandorachelin B (**4**) in DMSO-d_6 (600 MHz, CH and CH_3 blue colored, CH_2 black colored). 61
Figure S70. ^{1}H-^{13}C-HMBC spectrum of pandorachelin B (**4**) in DMSO-d_6 (600 MHz). 62
Figure S71. ^{1}H-^{1}H-COSY spectrum of pandorachelin B (**4**) in DMSO-d_6 (600 MHz). 62
Figure S72. ^{1}H-^{1}H-TOCSY spectrum of pandorachelin B (**4**) in DMSO-d_6 (600 MHz). 63
Figure S73. ^{1}H-^{1}H-ROESY spectrum of pandorachelin B (**4**) in DMSO-d_6 (600 MHz). 63

Tables

Table S1. Description of bacterial strains used in this study. 8
Table S2. Deduced proteins from the *pdn* biosynthesis gene cluster from *P. norimbergensis*. 9
Table S3. Predicted A domain substrate specificity of proteins encoded by *pdn* NRPS genes from *P. norimbergensis*. 10
Table S4. Predicted A domain substrate specificity of proteins encoded by *pdn* NRPS genes from *P. horticolens*. 10
Table S5. Predicted A domain substrate specificity of proteins encoded by *pdn* NRPS genes from *P. capi*. 10
Table S6. Antibiotic sensitivity test on LB agar plate. 13
Table S7. Primers used in this study. 14
Table S8. Plasmids used in this study. 15
Table S9. ^{1}H- and ^{13}C-NMR data of pandorachelin A (**1**) (600 MHz, DMSO-d_6). 18
Table S10. Comparison of our NMR data of ^{1}H- and ^{13}C NMR spectra in DMSO-d_6 of pandorachelin A (**1**) and the data of pandorachelin (**3**) detected by the Butler's group in DMSO-d_6 20
Table S11. Retention times in min of reference compounds and pandorachelin A (**1**) hydrolysate. 24
Table S12. ^{1}H- and ^{13}C-NMR data of pandorachelin B (**4**) (600 MHz, DMSO-d_6). 29
Table S13. Proteins that are homologous to PdnM of *Pandoraea norimbergensis*. 35
Table S14. List of acylases used for phylogenetic tree. 40
Table S15. Quantification of pandorachelins produced by *Pandoraea* species for 3 days. 54

General methods

HPLC-HRMS/MS

Tandem mass spectrometry measurements were performed on a QExactive Orbitrap High Performance Benchtop LC/MS (Thermo Fisher Scientific) with an electron spray ion (ESI) source and an Accela HPLC System, C18 column (Accucore C18 2.6 µm, 100 × 2.1 mm, Thermo Fisher Scientific) and the following solvents: acetonitrile + 0.1% formic acid and water + 0.1% formic acid at a flow rate of 0.2 mL · min^{-1}; gradient: 0–10 min 5→98% acetonitrile, hold 4 min at 98% acetonitrile, 14–14.1 min 98%→5% acetonitrile, hold 6 min at 5% acetonitrile.

NMR spectroscopy

NMR spectra were measured on a Bruker Avance DRX 500 MHz or Bruker Avance III 600 MHz spectrometer equipped with a cryo-platform and signals were referenced to the residual solvent signal DMSO-d_6 (2.50 ppm (^{1}H) or 39.52 ppm (^{13}C)) and CD_3NO_2 (4.37 ppm (^{1}H) or 0 ppm (^{15}N)), at a temperature of 300 K.

Chemicals

All chemicals and solvents were obtained from commercial suppliers (Roth, BD, Sigma Aldrich, Fluka, Alfa Aesar, Novabiochem, VWR, Cambridge Isotope Laboratories, Inc., Deutero) and used without further purification unless otherwise explained.

UV-Vis spectroscopy

A UV-1800 spectrometer (Shimadzu) with quartz glass cuvette (type 100-QS) with 10 mm path length was used to obtained UV-Vis spectra. The samples were measured at 21 °C in MeOH.

IR spectroscopy

Infrared spectra were measured with a Jasco Fourier Transform Infrared (FTIR) Spectrometer 4100 (Jasco) using the ATR technique.

Optical rotation

Specific optical rotation of the enantiopure compounds was measured with a P-1020 (Jasco) in a quartz glass cuvette with 50 mm path length at a wavelength of 589 nm (Na-D line). The concentration was given in 10 mg/mL.

MALDI-TOF-MS

MALDI-TOF-MS analysis of in-gel tryptic-digested proteins was performed using a timsTOF fleX MALDI-2 instrument (Bruker Daltonics, Billerica MA, USA). To prepare the matrix/peptide mixtures for analysis, 0.5 µL of the tryptic-digested sample was mixed with 0.5 µL of matrix solution, α-cyano-4-hydroxycinnamic acid (CHCA, 10 mg · mL^{-1}) in acetonitrile/0.1% formic acid (3:1), on a target plate. The mixture was dried at room temperature. All mass spectra were acquired in positive ion mode and a mass range of *m*/*z* 700-3200 per spectrum, with 1000 shots accumulated at 50% relative laser power and a repetition rate of 10 kHz, bursts of 200 shots from 5 random positions of the sample. Fingerprint analysis of the digested samples was performed by processing and analyzing the acquired data in Bruker Compass DataAnalysis 6.1, Bruker BioTools 3.2, and Sequence Editor 3.2.

HPLC-MS/MS

HPLC-MS/MS analyses of tryptic-digested samples was performed on a Vanquish UPLC system (Thermo Fisher Scientific) connected via ESI to a timsTOF flex MALDI-2 mass spectrometer (Bruker Daltonics) UPLC was carried out using a HypersilGOLD C8 column (Thermo Fisher Scientific, 150 × 2.1 mm, 1.9 µm particle size, 175 Å pore size) using the following gradient system: solvent A (water + 0.1% formic acid), solvent B (acetonitrile + 0.1% formic acid), 2% B for 2 min, to 10% B in 4.5 min, to 50% B in 30.5 min, to 98% B in in 2 min, hold 2 min, to 2% B in 1 min, at 25 °C oven temperature and a flow rate of 0.3 mL · min^{-1}. MS data was acquired in positive ion mode and a mass range of *m*/*z* 500–3,150 using Bruker Auto MS/MS with active exclusion (TOP5, 3 Hz, 100% duty cycle, 10k target intensity) and Focus mode detection.

Experimental procedures

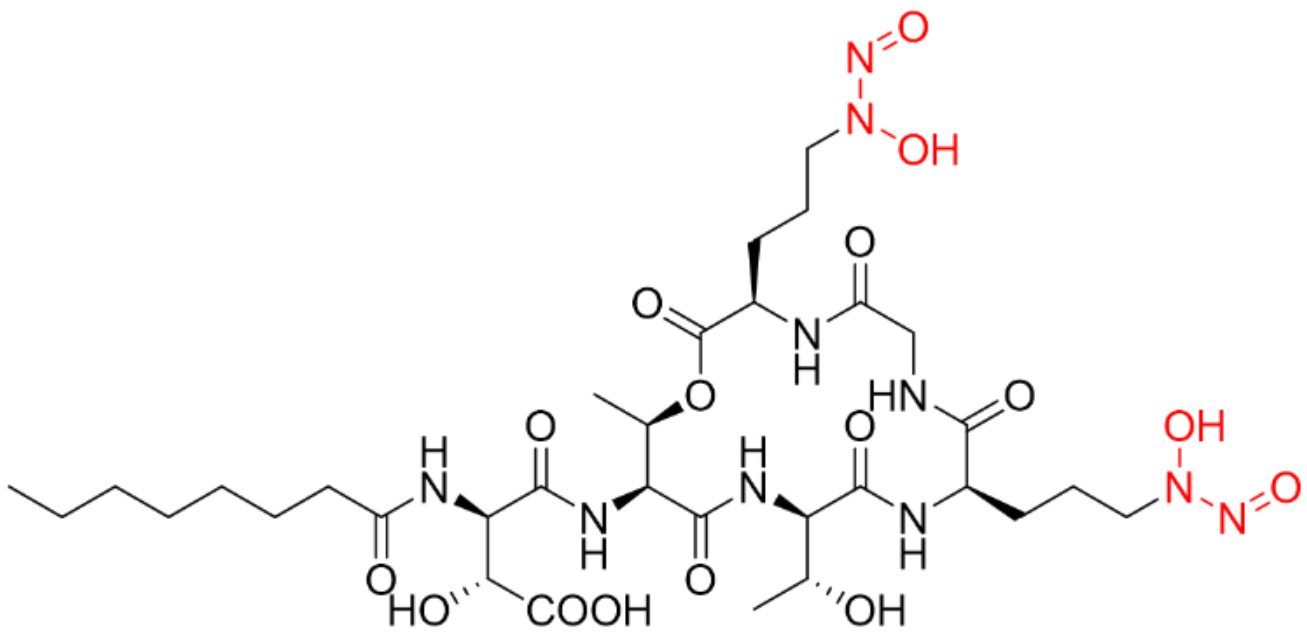

Figure S1. Structure of gramibactin.

Sequence similarity network (SSN)

The GrbD amino acid sequence from *P. norimbergensis* was used to construct a sequence similarity network (SSN) using EFI-EST with default settings. As the GrbD sequence from *P. capi* was absent from the UniProt database and was therefore not included in the network, its homologous sequence was manually added to the dataset. The modified FASTA file was then reanalyzed with EFI-EST. To finalize the SSN, an alignment score threshold of 200 was applied, and neighborhood connectivity was calculated. The finalized SSN was then submitted to EFI-GNT to generate the corresponding genome neighborhood analysis.[1, 2]

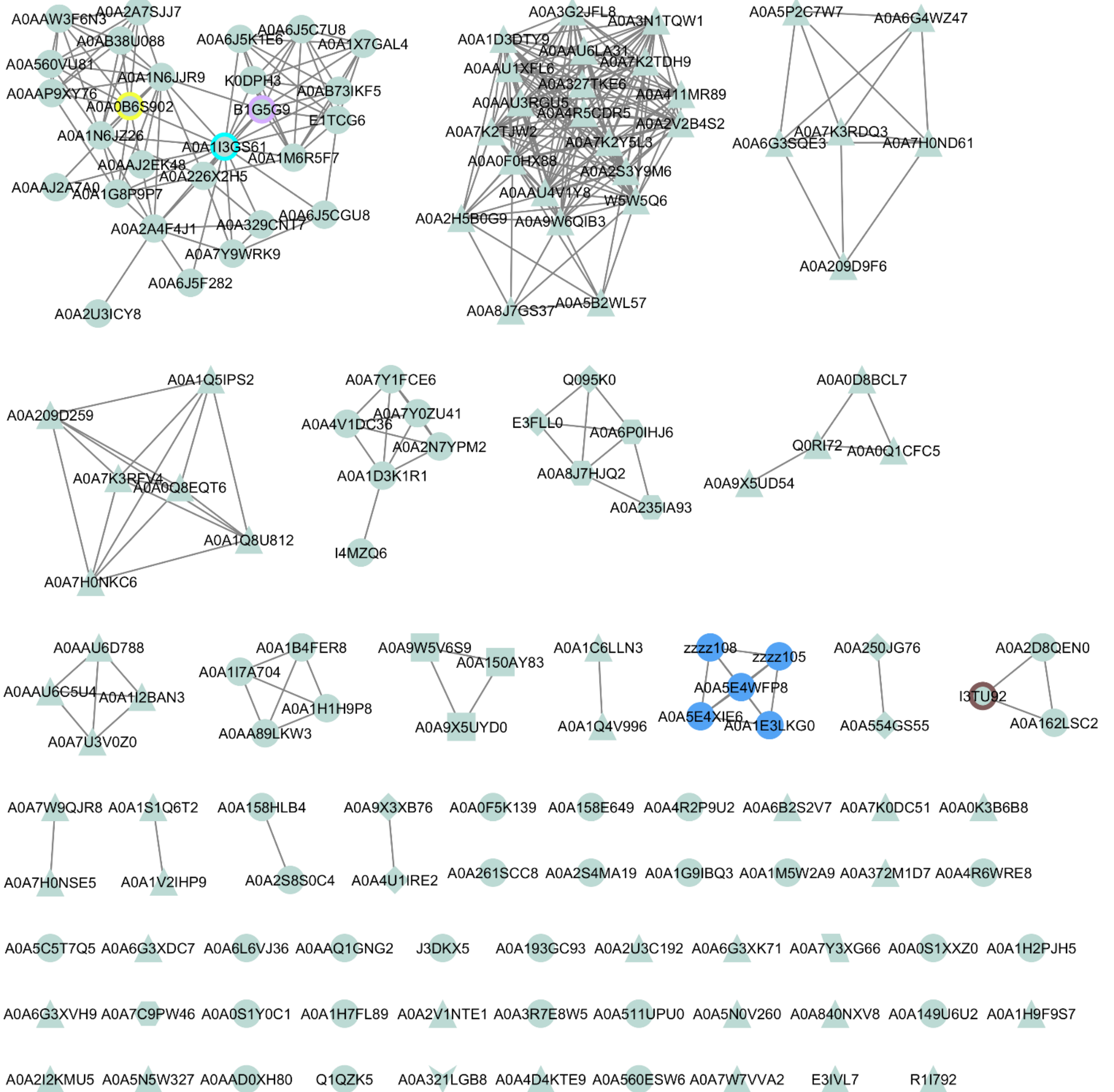

Figure S2: Complete sequence similarity network of GrbD homologs, annotated with UniProt accession numbers. Node outline colors indicate species: *B. plantarii* (yellow), *P. graminis* (lilac), *P. megapolitana* (blue), and *T. mobilis* KA081020-065 (brown). *Pandoraea* strains are colored blue. For *P. norimbergensis* and *P. capi*, identifiers zzzz108 and zzzz105 were assigned, respectively.

Bacterial strains and culturing conditions

Table S1. Description of bacterial strains used in this study.

Species	Strain	Character	Source or reference
Pandoraea norimbergensis	DSM11628 (CCUG 39188, LMG 18379)	Wild type; isolated from oxic water above sulfide containing lake sediment in Germany	DSMZ
	Δ*pdnI*	Tet^R gene inserted into *pdnI* gene	This study
	Δ*pdnM*	Tet^R gene inserted into *pdnM* gene	This study
	$pdnM^{hort}$	Native *pdnM* gene swapped with *pdnM* gene from *P. horticolens*	This study
	pdnM-tet-N	Tet^R gene inserted between *pdnM* and *pdnN* genes	This study
Pandoraea horticolens	LMG 31112 (CCUG 73379T)	Wild type; isolated from soil in Belgium	CCUG
Pandoraea capi	LMG 20602, (CCUG 45679T)	Wild type, isolated from sputum of woman with pneumonia in USA	CCUG
Escherichia coli	XL1 Blue	General cloning host strain	Stratagene
	TOP10	General cloning host strain	Invitrogen
	Rosetta2 (DE3)	Heterologous expression host strain with rare codons	Novagen
	Rosetta2 (DE3)::pET26-pdnM12	His_6-PanM-His_6 heterologous expression strain	This study

Analysis of the putative *pdn* biosynthesis gene cluster

Analyses of genome sequences of *P. norimbergensis*, *P. horticolens* and *P.capi* were performed by antiSMASH version 8.0.1[3] and NCBI BLASTp[4] (database: swiss-prot and non-redundant protein sequences). The putative *pdn* biosynthesis gene cluster from *P. norimbergensis* is depicted in Figure S3; the other strains showed analogous results. Predictions of A domain substrate specificity were performed using antiSMASH version 8.0.1[3] and a PKS/NRPS analysis tool.[5]

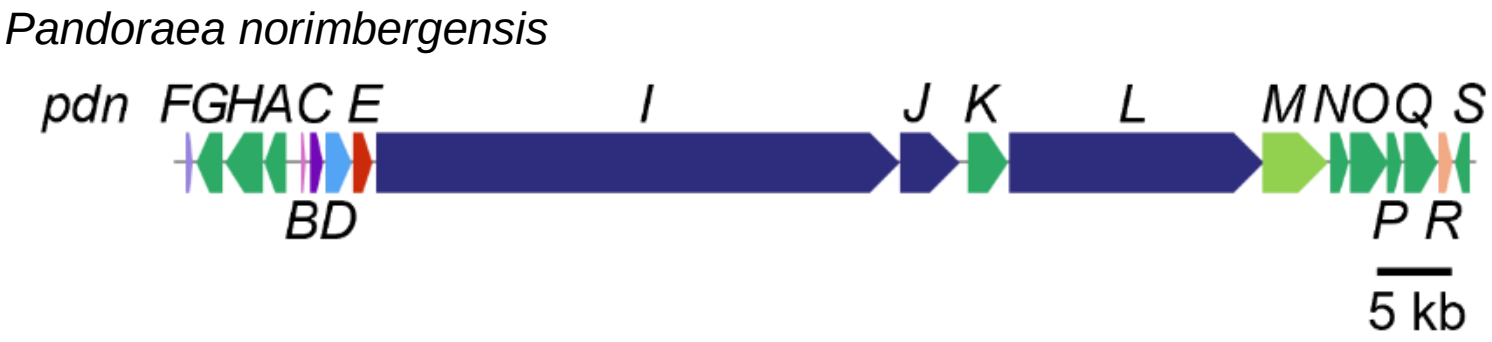

Figure S3. Putative *pdn* biosynthesis gene cluster in the genome of *Pandoraea norimbergensis* with the locus tag of *pdnI*: ctg1_2484.

Table S2. Deduced proteins from the *pdn* biosynthesis gene cluster from *P. norimbergensis*.

Protein	Size (aa)	Putative function	Sequence (identity/similarity).	Query coverage	Accession No.
PdnF	121	Hypothetical protein	Cupin domain-containing protein (88%/95%) *Bordetella* genomosp. 9	88%	WP_094845955.1
PdnG	496	RND efflux system, outer membrane lipoprotein	Pyoverdine export outer membrane protein OpmQ (46%/60%) *Pseudomonas aeruginosa* PAO1	93%	Q9I189.1
PdnH	686	ABC transporter ATP-binding protein	Pyoverdine export ATP-binding/permease protein PvdT (56%/71%) *Pseudomonas aeruginosa* UCBPP-PA14	98%	Q02MI4.1
PdnA	397	RND family efflux transporter MFP subunit	Pyoverdine export membrane fusion protein PvdR (57%/70%) *Pseudomonas putida* KT2440	90%	Q88F89.1
PdnB	82	MbtH family protein	Protein MbtH (48%/71%) *Mycobacterium tuberculosis variant bovis* AF2122/97	80%	P59965.1
PdnC	219	Thioesterase type II	Gramicidin S biosynthesis protein GrsT (33%/50%) *Aneurinibacillus migulanus*	92%	P14686.1
PdnD	483	GrbD	Nitrosourea synthase (27%/42%) *Streptomyces achromogenes* subsp. *streptozoticus*	84%	A0A411MR89.1
PdnE	364	GrbE	Putative N(omega)-hydroxy-L-arginine synthase DcsA (32%/47%) *Streptomyces lavendulae*	64%	D2Z024.1
PdnI	9401	NRPS	Mycosubtilin synthase subunit B (32%/50%) *Bacillus subtilis*	95%	Q9R9J0.1
PdnJ	1078	NRPS	Plipastatin synthase subunit C (30%/46%) *Bacillus subtilis* subsp. *subtilis* str. 168	82%	P39847.2
PdnK	758	TonB-dependent siderophore receptor	Fe(3+)-pyochelin receptor (35%/51%) *Pseudomonas aeruginosa* PAO1	88%	P42512.1
PdnL	4559	NRPS	Tyrocidine synthase 3 (34%/50%) *Brevibacillus parabrevis*	96%	O30409.1
PdnM	1179	Pandorachelin B acylase	Acyl-homoserine lactone acylase PvdQ (34%/49%) *Pseudomonas protegens* Pf-5	69%	Q4KCM5.1
PdnN	310	Iron compound ABC transporter	Ferric aerobactin-binding protein VatD (25%/46%) *Vibrio vulnificus*	90%	Q845T3.2
PdnO	669	Transport system permease protein	Iron(3+)-hydroxamate import system permease protein FhuB (48%/64%) *Salmonella enterica* subsp. *enterica* serovar *Typhimurium* str. LT2	98%	O87656.2
PdnP	285	ABC transporter ATP-binding protein	Iron(3+)-hydroxamate import ATP-binding protein FhuC (62%/74%) *Escherichia coli* K-12	89%	P07821.3
PdnQ	581	ABC transporter related protein	ATP-binding protein SyrD (33%/52%) *Pseudomonas syringae* pv. *syringae*	94%	P33951.1
PdnR	233	4'-Phosphopantetheinyl transferase	4'-Phosphopantetheinyl transferase HetI (40%/52%) *Nostoc* sp. PCC 7120	72%	P37695.2
PdnS		TonB protein	TonB family protein (48%/63%) *Trinickia* sp.	34%	HWX12357.1

Table S3. Predicted A domain substrate specificity of proteins encoded by *pdn* NRPS genes from *P. norimbergensis*. X is unknown amino acid.

A-domain	Stachelhaus code	antiSMASH	PKS/NRPS analysis
A1	D F W N V G M V H K	Thr	Thr
A2	D V H R T G L V A K	X	X
A3	D G E H H G T I T K	X	X
A4	D F W N I G M V H K	Thr	Thr
A5	D P E D H M T V T K	X	X
A6	D G E H H G T I T K	X	X
A7	D L T K V G H V G K	Asp	Asp
A8	D I L Q L G M I W K	Gly	X
A9	D G E D H G T V T K	Orn	Lys
A10	D I L Q L G L I W K	Gly	Gly
A11	D V H R T G L V A K	X	4-Hydroxy-L-phenylglycine

Table S4. Predicted A domain substrate specificity of proteins encoded by *pdn* NRPS genes from *P. horticolens*. X is unknown amino acid.

A-domain	Stachelhaus code	antiSMASH	PKS/NRPS analysis
A1	D F W N V G M V H K	Thr	Thr
A2	D V H R T G L V A K	X	4-Hydroxy-L-phenylglycine
A3	D G E H H G T I T K	X	X
A4	D F W N I G M V H K	Thr	Thr
A5	D P E D H M T V T K	X	X
A6	D G E H H G T I T K	X	X
A7	D L T K V G H V G K	Asp	Asp
A8	D I L Q L G M I W K	Gly	X
A9	D G E D H G T V T K	Orn	Lys
A10	D I L Q L G L I W K	Gly	Gly
A11	D V H R T G L V A K	X	4-Hydroxy-L-phenylglycine

Table S5. Predicted A domain substrate specificity of proteins encoded by *pdn* NRPS genes from *P. capi*. X is unknown amino acid.

A-domain	Stachelhaus code	antiSMASH	PKS/NRPS analysis
A1	D F W N V G M V H K	Thr	Thr
A2	D V H R T G L V A K	X	4-Hydroxy-L-phenylglycine
A3	D G E H H G T I T K	X	X
A4	D F W N I G M V H K	Thr	Thr
A5	D P E D H M T V T K	X	X
A6	D G E H H G T I T K	X	X
A7	D L T K V G H V G K	Asp	Asp
A8	D I L Q L G M I W K	Gly	X
A9	D G E D H G T V T K	Orn	Lys
A10	D I L Q L G L I W K	Gly	Gly
A11	D V H R T G L V A K	X	4-Hydroxy-L-phenylglycine

Preparation of MM9 medium[6, 7, 8]

For a liter of medium, the following solutions were prepared and combined. Solution A [350 g K_2HPO_4 and 100 g KH_2PO_4 dissolved in 1 L ddH_2O] and solution B [29.4 g NaCl, 50 g $(NH_4)_2SO_4$, 5 g $MgSO_4$ dissolved in 1 L ddH_2O] were prepared and autoclaved separately. To 900 mL ddH_2O, 2 g of an amino acid mixture [2 g each of L-Ala, L-Arg, L-Asn, L-Asp, L-Cys, L-Gln, L-Glu, Gly, L-Ile, L-Pro, L-Ser, L-Thr, L-Tyr, L-Val, L-Phe] was added and autoclaved. 20 mL each of solution A and B were added to the mixture. 16.7 mL of sterile L-Leu (100 mM), 5 mL of L-His (60 mM), 10 mL of L-Lys (100 mM), 10 mL of L-Trp (40 mM), 10 mL of L-Met (40 mM), and 20 mL of glucose (50% (*w/v*)) were added. As needed, 1 mL of a sterile trace element solution [per liter: 40 mg $ZnCl_2$, 200 mg $FeCl_3 \cdot 6\ H_2O$, 10 mg $CuCl_2 \cdot 2\ H_2O$, 10 mg $MnCl_2 \cdot 4\ H_2O$, 10 mg $Na_2B_4O_7 \cdot 10\ H_2O$, 10 mg $(NH_4)_6Mo_7O_{24} \cdot 4\ H_2O$] was added.

Preparation of MM9 medium soft agar

For one liter of MM9 medium soft agar, the MM9 medium was prepared as described above with the addition of 6 g agar to the amino acid mixture.

Preparation of brain heart infusion broth

For one liter of medium, 37 g of the commercial medium (Roth) was added to 1 L ddH_2O before autoclaving.

Preparation of CAA medium

One liter of medium was prepared by adding 5 g of iron-limited casamino acids, 1.18 g $K_2HPO_4 \cdot 3\ H_2O$ and 0.25 g $MgSO_4 \cdot 7\ H_2O$ to 1 L ddH_2O and before autoclaving.

Preparation of ABG medium[10]

ABG medium consists of three components A, B and G, which are combined in defined proportions to prepare the final medium. Component A was prepared by dissolving 16 g of $(NH_4)_2SO_4$, 48 g of Na_2HPO_4, 24 g of KH_2PO_4 and 24 g NaCl in 800 mL ddH_2O before autoclaving. Additionally, before autoclaving 0.005% yeast extract was added to component A to boost the growth. For component B, 2 mL of 1 M $MgCl_2 \cdot 6\ H_2O$, 0.2 mL of 500 mM $CaCl_2 \cdot 2\ H_2O$ and 0.3 mL of 10 mM $FeCl_3 \cdot 6\ H_2O$ were added to 800 mL ddH_2O before autoclaving. Component G consisted of 20% glycerol (*v*/*v*) in ddH_2O, which was autoclaved. For the final medium, 100 mL of A, 800 mL of B and 100 mL G were combined.

Siderophore production in liquid media by different *Pandoraea* strains

P. norimbergensis, *P. horticolens*, *P. capi* were cultivated overnight at 30 °C in MM9 medium supplemented with trace elements with orbital shaking at 120 rpm. *P. norimbergensis* and *P. horticolens* were then inoculated (starting OD_{600} of 0.05) into 0.5 L MM9 medium with trace elements. *P. capi* was inoculated (starting OD_{600} of 0.05) into 0.3 L MM9 medium with trace elements. The cultures were distributed in baffled Erlenmeyer flasks and incubated at 30 °C with orbital shaking at 120 rpm for four days in 1 L Erlenmeyer flasks. For extraction, 5% (*w*/*v*) Amberlite® XAD16 resin was used, which was prepared by washing with methanol (3×) and acetone (2×) before equilibration with ddH_2O. The resin was added to the supernatants after removal of cells by centrifugation at 5,000 × *g* for 15 min at room temperature, and they were shaken at 100 rpm at room temperature for 1.5 h. The resin was removed by filtration and subsequently eluted with methanol. Eluates were concentrated under reduced pressure to remove solvent, followed by lyophilization. The dried samples were subsequently re-dissolved in H_2O for HPLC-HRMS analysis.

Isolation of pandorachelin A (1)

P. norimbergensis was cultivated overnight at 30 °C in MM9 medium with trace elements with orbital shaking at 120 rpm. 1 L MM9 medium with trace elements was inoculated with *P. norimbergensis* (starting OD_{600} of 0.05), then distributed in 1 L baffled Erlenmeyer flasks and incubated at 30 °C with orbital shaking at 120 rpm for three days. Extraction was performed with Amberlite® XAD16 resin as described above.
Extracts were purified by preparative RP-HPLC using a Phenomenex Synergi 10 µm Hydro-RP (250 × 21.2 mm, 10 µm, 80 Å) (solvent A: H_2O + 0.1% TFA, solvent B: 83% aqueous acetonitrile, time program: 5 min 3% B, 3% B to 17% B in 30 min, 17% B to 100% B in 1 min, 6 min 100% B, flow rate 18 mL · min^{-1}), t_R = 22.4 min (pandorachelin A (**1**)). The compound was further purified by preparative RP-HPLC using a Nucleodur HTec (250 × 10 mm, 5 µm, 100 Å) (solvent A: H_2O + 0.1% TFA, solvent B: 83% aqueous acetonitrile, time program: 2 min 3% B, 3% B to 30% B in 25 min, 30% B to 100% B in 1 min, 8 min 100% B, flow rate 5 mL · min^{-1}), t_R = 14.8 min (pandorachelin A (**1**)). The collected fraction was freeze-dried to yield 10.8 mg of pandorabactin A (**1**) as a white powder.

Pandorachelin A (1)
HRMS (ESI⁺): $[M+2H]^{2+}$ = 676.3132 (calculated 676.3135, $C_{50}H_{88}N_{20}O_{24}^{2+}$), $[\alpha]_D^{21.4}$ = 9.16 (c = 0.2 in MeOH)

Isolation of pandorachelin B (4)

P. horticolens was cultivated overnight at 30 °C in MM9 medium without trace elements with orbital shaking at 110 rpm. 1 L MM9 medium with trace elements was inoculated with *P. horticolens* (starting OD_{600} of 0.05), then distributed in 1 L baffled Erlenmeyer flasks and incubated at 30 °C with orbital shaking at 110 rpm for three days. Extraction was performed with Amberlite® XAD16 resin as described above.
Extracts were subjected to preparative RP-HPLC using a Phenomenex Synergi Fusion-RP (250 × 21.20 mm, 4 µm, 80 Å) (solvent A: H_2O + 0.1% TFA, solvent B: 83% aqueous acetonitrile, time program: 3% B to 17% B in 12 min, 17% B to 30% B in 3 min, 30% B to 55% B in 25 min, 55% B to 100% B in 10 min, 7 min 100% B flow rate 18 mL · min^{-1}), t_R = 35.5 min (pandorachelin B (**4**)). The collected fraction was freeze-dried to yield 7.68 mg of pandorachelin B (**4**) as a white powder.

Pandorachelin B (4)

HRMS (ESI⁺): $[M+2H]^{2+}$ = 767.3971 (calculated 767.3970, $C_{62}H_{110}N_{20}O_{25}^{2+}$), $[\alpha]_D^{22.0}$ = –3.81 (c = 0.2 in MeOH),

CAS assay

One liter of CAS agar was prepared following an established protocol.[11] The CAS reagent solution was prepared by dissolving 60.5 mg of chrome azurol S in 50 mL ddH_2O were combined with 10 mL of 1 mM $FeCl_3$ solution in 10 mM HCl (solution 1). While stirring, solution 1 was slowly added to solution 2 (72.9 mg of HDTMA (hexadecyltrimethylammonium bromide) dissolved in 40 mL ddH_2O), and the resulting dark blue solution was autoclaved. The solution was cooled to ca. 50 °C and slowly added to 900 mL of the autoclaved MM9 medium pH 6.8 (without trace elements) with 30.2 g 1,4-piperazinediethanesulfonic acid (PIPES) and 15 g · L^{-1} agar. To this solution, 16.7 mL of L-Leu (100 mM), 5 mL of L-His (60 mM), 10 mL of L-Lys (100 mM), 10 mL of L-Trp (40 mM), 10 mL of L-Met (40 mM), and 20 mL of glucose (50% (*w/v*)) were added. The medium was distributed in petri dishes and once solidified, *P. norimbergensis* was streaked before being incubated at 30 °C for 7 days (Figure S4).

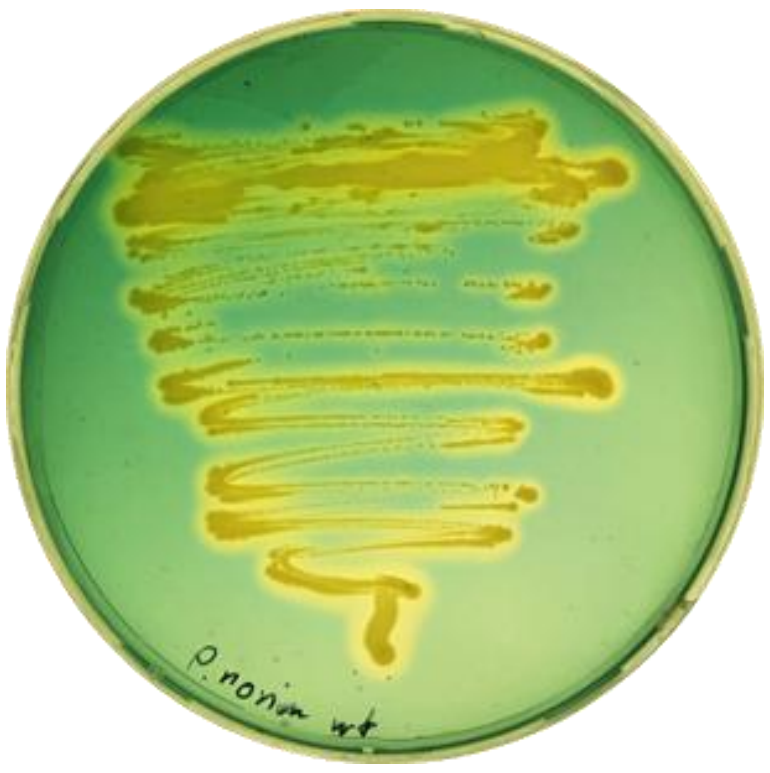

Figure S4. CAS agar plate with cultivated *P. norimbergensis*.

The chrome azurol S (CAS) assay was applied to evaluate the iron-binding properties of pandorachelin A (**1**) and B (**4**). The CAS solution was prepared following a published protocol.[12] For the qualitative analysis, pandorachelin A (**1**) and B (**4**) were individually dissolved in ddH_2O at a concentration of 15 µM. An EDTA solution (15 µM) served as positive control, while ddH_2O was used as negative control. For the assay, 250 µL of each compound or control solution was combined with 250 µL of the CAS solution.

For the quantitative analysis, serial dilutions of the pandorachelin A (**1**) and B (**4**) were prepared directly in a 96-well plate. To each well, 50 µL of the substrate at the designated concentration and 50 µL of CAS assay solution were added. The tested final concentrations were 400, 200, 100, 50, 25, 12.5, 6.25, 3.13, 1.56, 0.78, 0.39 µM. Absorbance at 630 nm was measured using a Thermo Fisher Multiskan Ascent microplate reader. No visible color change was observed after an incubation of 2 h at room temperature, but the well plate was analyzed again after 18 h. An immediate color change at up to 12.5 µM was observed for pandorachelin B (**4**). The assay was conducted in technical triplicates, except for the controls.

For the calculation of the EC_{50} values the absorbance value obtained for each compound concentration was normalized using the formula: ([A630 value of the treated sample at a given concentration – minimum A630 value]/[maximum A630 value – minimum A630 value]) × 100%, where the minimum value is derived from measuring untreated Fe-CAS solution (all Fe in complex with CAS reagent) and the maximum value is derived from measuring CAS solution treated with 200 µM EDTA (all Fe in complex with EDTA). In order to determine the extent of iron binding caused by each compound, EC_{50} values were calculated using GraphPad Prism version 10.1.2 for Windows (GraphPad Software, Boston, MA, USA; www.graphpad.com). The normalized values, corresponding to the percentage of Fe bound to CAS, were plotted against the logarithmic value (log_{10}) of the respective compound concentrations (µM). The data points represent the average, and the error bars represent the standard deviation (±S.D.). The best-fit dose-response curve was determined using a log(agonist) vs. response – variable slope (four parameters) fit. The fitting method was least squares regression, with no weighting and the bottom parameter fixed to zero. Confidence intervals were calculated (95%, profile likelihood) to visualize the extent to which the data defines the true curve.

Generation of *pdnI* mutant of *P. norimbergensis* DSM11628

P. norimbergensis DSM11628 genomic DNA was isolated by using Promega Wizard® DNA Purification kit (Promega GmbH). Two gene fragments were amplified by PCR with the primer pairs PnNRPS-KO1-HindIII/PnNRPS-KO1-PacI and PnNRPS-KO2-KpnI/PnNRPS-KO2-NheI using Phusion Polymerase (New England Biolabs). The obtaining amplicons were purified using the illustra GFX PCR DNA and Gel Band Purification Kit (GE Healthcare Life Sciences) and inserted into pCR bluntII vector, resulting in pCR-PnNRPS-KO1 and pCR-PnNRPS-KO2, respectively. *Pac*I/*Kpn*I-restricted tetracycline resistance gene from pGEM-Tet^R, *Hind*III/*Pac*I-restricted pCR-PnNRPS-KO1, and *Kpn*I/*Nhe*I-restricted pCR-PnNRPS-KO2 were ligated into *Hind*III/*Spe*I-restricted pGL42a_T251A,[7, 8, 13] generating pGL42a_T251A-Δ*PnNRPS*.

P. norimbergensis DSM11628 was grown in 2 mL LB medium at 30 °C with orbital shaking. LB medium was inoculated from the overnight culture (1/100 dilution), before being incubated at 30 °C with orbital shaking until an OD_{600} of 0.4 to 0.6 was reached. The culture was centrifuged at 3,000 × *g*, then the supernatant was discarded. The cell pellet was resuspended in 300 mM sucrose and centrifuged again. After repeating this washing step three times, the washed cells were resuspended in 300 mM sucrose and then subjected to electroporation (2,500 V) with pGL42a_T251A-Δ*PnNRPS* (*ca.* 1 µg) at room temperature. Transformed cells were cultured in LB medium (1 mL) for 5 h at 30 °C with orbital shaking, then spread on LB agar plates containing tetracycline (60 µg · mL^{-1}). After 3 days, several colonies were observed and transferred to LB agar plates containing tetracycline (60 µg · mL^{-1}) and D,L-*p*-chlorophenylalanine (2 mg · mL^{-1}). These mutants were cultured at 30 °C for 2 days and again transferred to LB agar plates containing tetracycline (60 µg · mL^{-1}) and D,L-*p*-chlorophenylalanine (2 mg · mL^{-1}). After cultivation at 30 °C for 3 days, these mutants were checked by colony PCR using the primer pair PnNRPS-fw/PnNRPS-rv.

Table S6. Antibiotic sensitivity test on LB agar plate.

Antibiotic	Concentration	Sensitivity
Ampicillin	100 µg mL^{-1}	Resistant
Apramycin	50 µg mL^{-1}	Resistant
Chloromphenicol	100 µg mL^{-1}	Resistant
Erythromycin	500 µg mL^{-1}	Sensitive
Gentamycin	20 µg mL^{-1}	Resistant
Kanamycin	300 µg mL^{-1}	Resistant
Spectinomycin	50 µg mL^{-1}	Resistant
Tetracycline	60 µg mL^{-1}	Sensitive
Trimethoprim	500 µg mL^{-1}	Resistant

Table S7. Primers used in this study. Underline indicates a restriction site.

Primer	Nucleotide (5' to 3')	Source
Tet-fw-PacI	GGTTTAATTAATCAGATAAAATATTTCTAGATTTCAGTGC	This study
Tet-rv-KpnI	GGTGGTACCAATTCTTGGAGTGGTGAATCCG	This study
PnNRPS-KO1-HindIII	GGTAAGCTTGATTTGCGTGCAGACCGGCG	This study
PnNRPS-KO1-PacI	GGTTTAATTAACCGTCGCCAAGCGTCTGTCC	This study
PnNRPS-KO2-KpnI	GGTGGTACCCTGGGCATGACGCAAGACGG	This study
PnNRPS-KO2-NheI	GGTGCTAGCCGACGCAACACTGGCATCGG	This study
PnNRPS-fw	TCCCTGCTATGGACTGGCCG	This study
PnNRPS-rv	GTTCGTCGACGATCTGCGCC	This study
PdnM-KO1-XhoI	GGTCTCGAGACAAATCGAGCGGACGACGG	This study
PdnM-KO1-PacI	GGTTTAATTAATCGCACCGAGATTGACGACC	This study
PdnM-KO2-KpnI	GGTGGTACCATGGATCACGTTCGGCCTGC	This study
PdnM-KO2-NheI	GGTGCTAGCACTGTCGAGCCAAAGTGCGC	This study
PdnM-fw	AACCTGTCGCCATGACACCC	This study
PdnM-rv	TGTCACAGCGGCAAAGCCGG	This study
PdnM-NheI-2His-fw	GGTGCTAGCTGCGCGGCAGACGCGTCGC	This study
PdnM-BamHI-rv	GGTGGATCCTTTCTAGTGGGGGCGTTCTGCG	This study
Pnori-pdnL-fw-NEB	CTCACTATAGGGCGAATTGGGACAACTTCTTCGCCATCGG	This study
Pnori-pdnL-rv-NEB	CGTTTGATACGAAATGTCATGAGTGATTGCCAGCCGAGGTG	This study
PdnMfort-NEB-fw	TGCCGCGCGGCAGCCATATGGCTAGCTGTACGGCGGATGCGT CGCATTC	This study
PdnMfort-NEB-rv	GTCGACGGAGCTCGAATTCG GATCCTTTGCAACTTTCCGAGGGTTCC	This study
Pnori-pdnN-fw-KpnI	GGTGGTACCCCCCCAATGACATTACCGC	This study
Pnori-pdnN-rv-SacI	GGTGAGCTCTTGAACAGATCGATGAGCGG	This study
Pnori-pdnL-fw2	GGAGTCGATGTTGTTCGACG	This study

Table S8. Plasmids used in this study.

Plasmid	Character	Source or reference
pGEM T-easy	General T/A cloning vector	Promega
pCR bluntII	General blunt end cloning vector	Thermo Fisher Scientific
pCR2.1	General T/A cloning vector	Thermo Fisher Scientific
pACYC184	General cloning vector	Invitrogen
pGL42a-T251A	Suicide plasmid using *pheS* modified with T251A	[7, 8, 13]
pGEM-Tet^R	pGEM T-easy with tetracycline resistance gene cassette (Tet^R) containing *Pac*I/*Kpn*I restriction sites	This study
pCR-PnNRPS-KO1	pCR bluntII with partial *pdnI* gene	This study
pCR-PnNRPS-KO2	pCR bluntII with partial *pdnI* gene	This study
pGL42a_T251A-ΔPnNRPS	pGL42a_T251A containing *pdnI* gene insertional Tet^R gene	This study
pCR-pdnM-KO1	pCR2.1 with partial *pdnM* gene	This study
pCR-pdnM-KO2	pCR2.1 with partial *pdnM* gene	This study
pGL42a_T251A-ΔpdnM	pGL42a_T251A containing *pdnM* gene insertional Tet^R gene	This study
pCR-pdnM12	pCR2.1 with *pdnM* gene without signal peptide gene	This study
pET28-pdnM12	pET28a with *pdnM* gene without signal peptide gene	This study
pET26-pdnM12	pET26b with His_6-*pdnM* gene without signal peptide gene	This study
pGL42a_T251A-pdnLM^{hort}	pGL42a_T251A containing partial *pdnL, pdnM^{hort}*, *Tet*^{R}, partial *pdnI* genes	This study
pCRbluntII-pdnN	pCR bluntII with partial *pdnN* gene	This study
pGL42a_T251A-pdnM^{hort}Swap	pGL42a-T251A containing *pdnLM^{hort}*, *Tet*^{R}, partial *pdnN* genes	This study
pGL42a_T251A-pdnM	pGL42a_T251A containing partial *pdnM*, *Tet*^{R}, partial *pdnI* genes	This study
pGL42a_T251A-pdnM-Tet-N	pGL42a_T251A containing partial *pdnM*, *Tet*^{R}, partial *pdnN* genes	This study

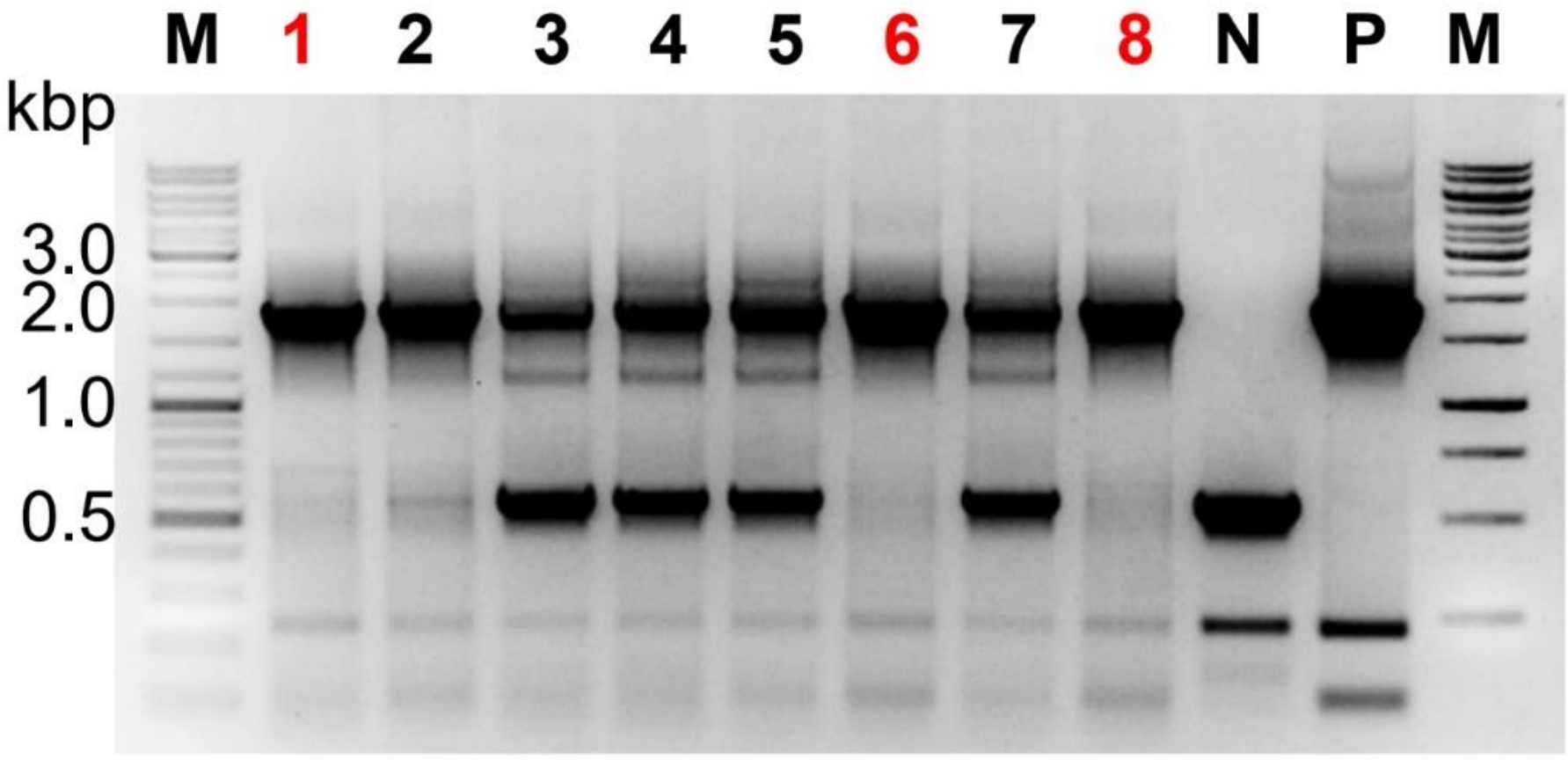

Figure S5. PCR confirmation of *P. norimbergensis* mutants. Template DNA: genomic DNA of *P. norimbergensis* Δ*pdnI* (1–8), genomic DNA of *P. norimbergensis* wild type (N), pGL42a_T251A-Δ*pdnI* (P). M: DNA size marker. The estimated size of amplicons obtained by using the primer pair PnNRPS-fw/PnNRPS-rv, lane 1–8 and P; 1,856 bp, N; 554 bp. Red numbers were selected as double-crossover mutants.

Extraction of *P. norimbergensis* Δ*pdnI*

P. norimbergensis Δ*pdnI* was cultivated overnight at 30 °C in MM9 medium without trace elements supplemented with tetracycline (60 µg · mL^{-1}) with orbital shaking at 150 rpm. 50 mL MM9 medium without trace elements and tetracycline (60 µg · mL^{-1}) was inoculated with *P. norimbergensis* Δ*pdnI* (starting OD_{600} of 0.05) in 300 mL baffled Erlenmeyer flasks and incubated at 30 °C with orbital shaken at 120 rpm for four days. Extraction was performed with Amberlite® XAD16 resin as described above. The cultivation was performed in biological triplicates.

Detection of the compounds

To compare the fragmentation pattern of the compound produced by *P. norimbergensis*, *P. capi* and *P. oxalativorans* in MM9 medium, HRESI-MS/MS analysis was performed.

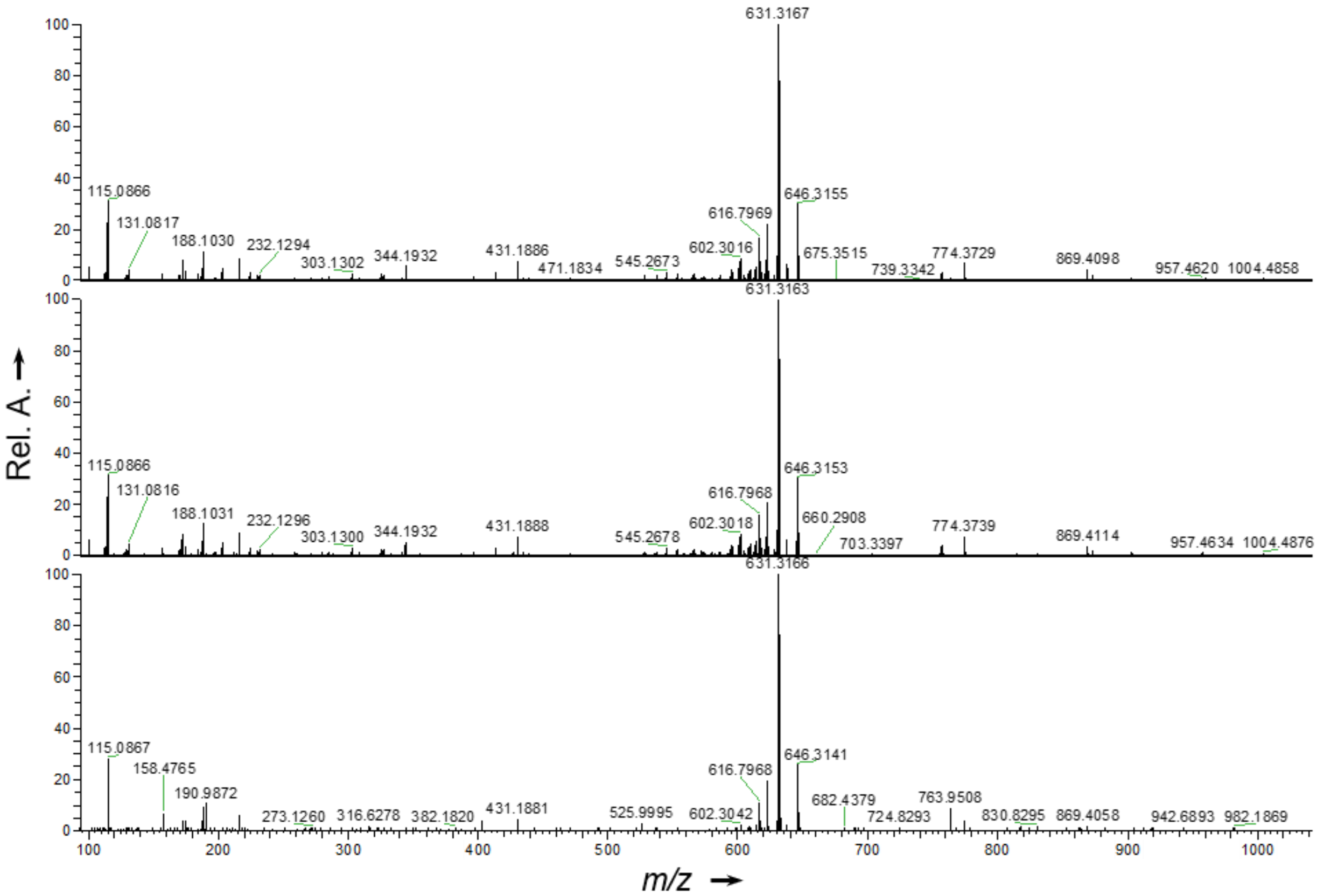

Figure S6. HR-ESI-MS/MS (ESI$^+$, hcd 25) spectra of pandorachelin A (**1**) at *m/z* 676.3135 $[M+2H]^{2+}$ produced by *P. norimbergensis* (top), *P. horticolens* (middle), and *P. capi* (bottom).

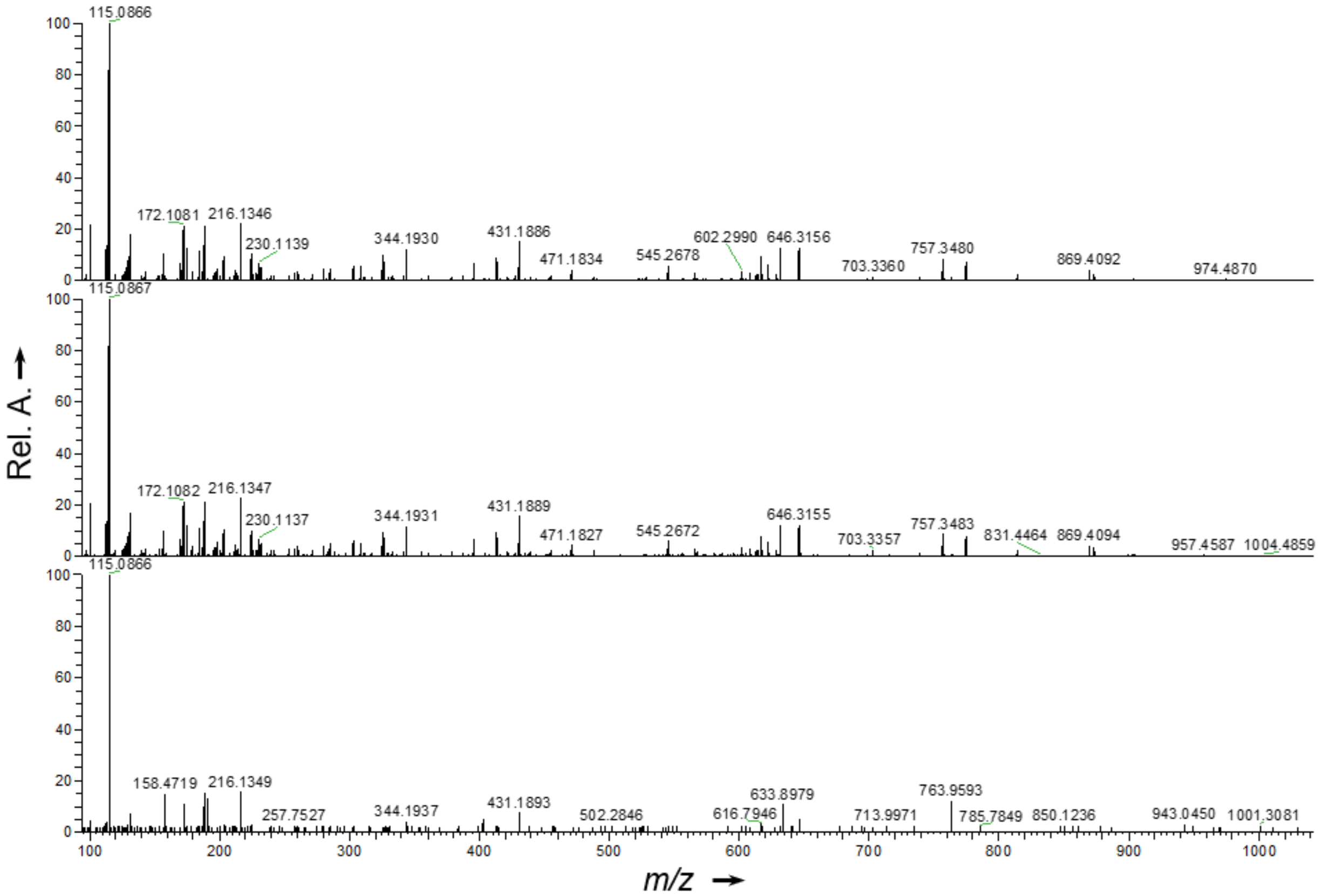

Figure S7. HR-ESI-MS/MS (ESI$^+$, hcd 30) spectra of pandorachelin A (**1**) at *m/z* 676.3135 $[M+2H]^{2+}$ produced by *P. norimbergensis* (top), *P. horticolens* (middle), and *P. capi* (bottom).

Determination of the structure of pandorachelin A (1)

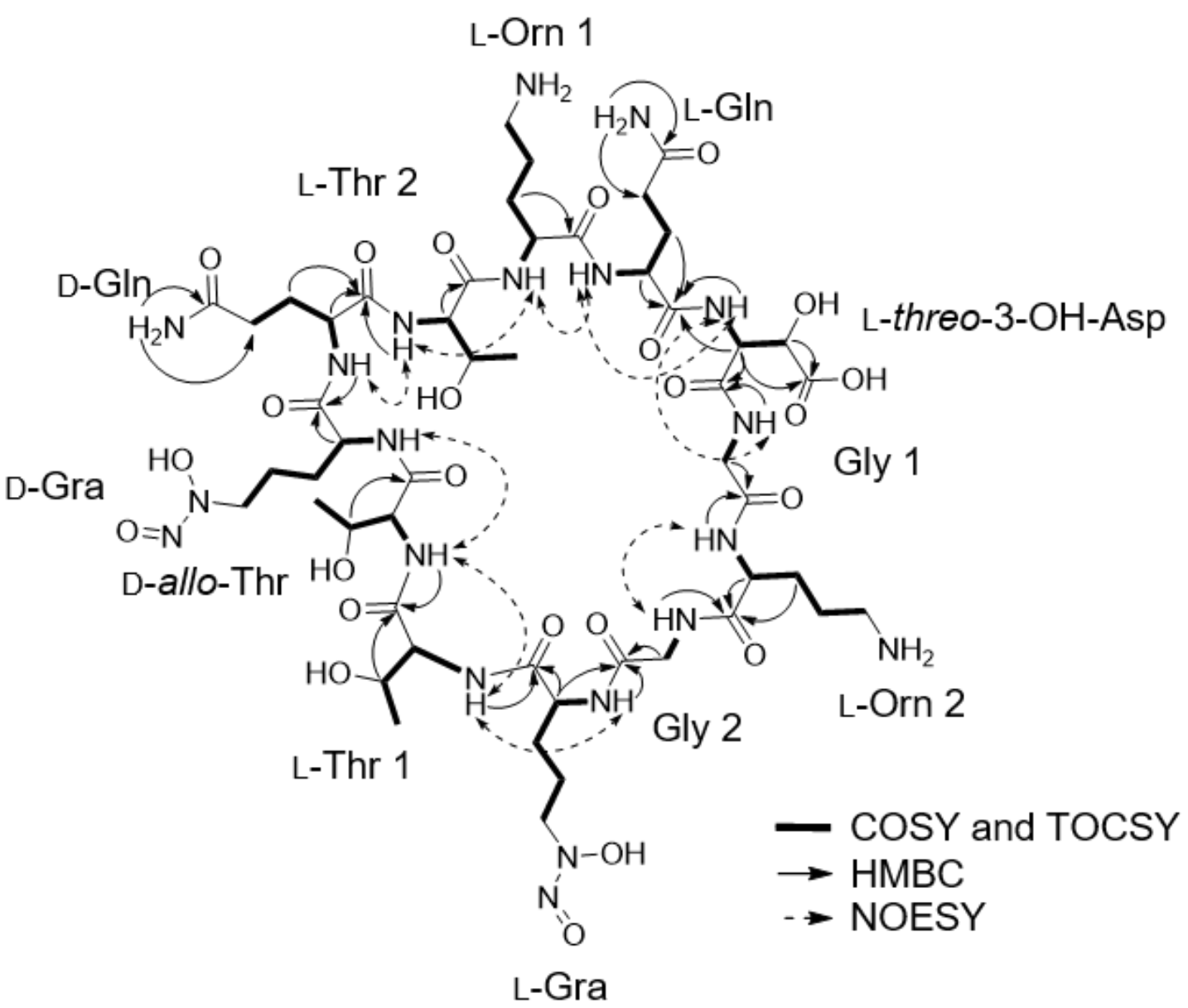

Figure S8. Structure elucidation of pandorachelin A (**1**) with key correlations of ^{1}H-^{1}H-COSY, ^{1}H-^{1}H-TOCSY ^{1}H-^{13}C-HMBC and ^{1}H-^{1}H-NOESY.

Table S9. ^{1}H- and ^{13}C-NMR data of pandorachelin A (**1**) (600 MHz, DMSO-d_6).

Partial structure	Position	δ_C [ppm] (m)	δ_H [ppm] (m, *J* [Hz])	δ_N [ppm]
L-Threonine 1	NH	-	7.88 (1H, d, 6.1)	
	CO	170.2 (C)	-	
	C_α	58.7 (CH)	4.18 (1H, brdd, 7.3, 4.5)	
	C_β	66.3 (CH)	4.03 (1H, m)	
	C_γ	19.9 (CH_3)	1.02 (3H, d, 6.3)	
D-*allo*-Threonine	NH	-	7.74 (1H, d, 7.6)	
	CO	171.0 (C)	-	
	C_α	58.6 (CH)	4.23 (1H, t, 8.0)	
	C_β	67.3 (CH)	3.90 (1H, m)	
	C_γ	20.1 (CH_3)	1.09 (3H, d, 6.1)	
D-Graminine	NH	-	8.25 (1H, m)	
	CO	170.5 (C)	-	
	C_α	52.6 (CH)	4.22 (1H, m)	
	C_β	28.4 (CH_2)	1.75 (1H, m), 1.59 (1H, m)	
	C_γ	23.1 (CH_2)	1.83 (2H, m)	
	C_δ	61.5 (CH_2)	4.06 (2H, m)	
	NOH	-	-	316.9
	NO	-	-	367.3
D-Glutamine	NH	-	7.95 (1H, m)	
	CO	171.9 (C)	-	
	C_α	52.5 (CH)	4.32 (1H, m)	
	C_β	27.6 (CH_2)	1.93 (1H, m), 1.74 (1H, m)	
	C_γ	31.4 (CH_2)	2.11 (2H, m)	
	CO	174.0 (C)	-	
	NH_2	-	7.31 (1H, m), 6.86 (1H, m)	
L-Threonine 2	NH	-	7.81 (1H, d, 8.0)	
	CO	170.8 (C)	-	
	C_α	58.2 (CH)	4.23 (1H, m)	
	C_β	66.6 (CH)	4.14 (1H, m)	
	C_γ	19.9 (CH_3)	1.04 (3H, d, 6.2)	
L-Ornithine 1	NH	-	8.06 (1H, m)	
	CO	171.2 (C)	-	
	C_α	52.7 (CH)	4.27 (1H, m)	
	C_β	28.0 (CH_2)	1.78 (1H, m), 1.56 (1H, m)	
	C_γ	23.5 (CH_2)	1.59 (2H, m)	
	C_δ	38.6 (CH_2)	2.80 (2H, m)	
	NH_2	-	7.66 (2H, m)	
L-Glutamine	NH	-	8.14 (1H, m)	
	CO	171.5 (C)	-	
	C_α	53.3 (CH)	4.20 (1H, m)	
	C_β	27.5 (CH_2)	1.89 (1H, m), 1.76 (1H, m)	
	C_γ	31.4 (CH_2)	2.10 (2H, m)	
	CO	174.1	-	
	NH_2	-	7.23 (1H, s), 6.82 (1H, s)	
L-*threo*-3-OH-Aspartate	NH	-	7.63 (1H, m)	
	CO	169.3 (C)	-	
	C_α	55.3 (CH)	4.73 (1H, dd, 9.0, 2.1)	

	C_β	70.4 (CH)	4.50 (1H, m)	
	COOH	172.9 (C)	-	
Glycine 1	NH	-	8.09 (1H, m)	
	CO	168.7 (C)	-	
	C_α	42.5 (CH_2)	3.90 (1H, m), 3.69 (1H, dd, 16.2, 4.1)	
L-Ornithine 2	NH	-	8.02 (1H, d, 8.0)	
	CO	171.6 (C)	-	
	C_α	51.8 (CH)	4.38 (1H, m)	
	C_β	28.9 (CH_2)	1.72 (1H, m), 1.55 (1H, m)	
	C_γ	23.5 (CH_2)	1.53 (2H, m)	
	C_δ	38.7 (CH_2)	2.78 (2H, m)	
	NH_2	-	7.65 (2H, m)	
Glycine 2	NH	-	8.19 (1H, m)	
	CO	169.3 (C)	-	
	C_α	42.1 (CH_2)	3.87 (1H, m), 3.75 (1H, dd, 15.8, 3.9)	
L-Graminine	NH	-	8.21 (1H, d, 6.8)	
	CO	171.6 (C)	-	
	C_α	52.6 (CH)	4.28 (1H, m)	
	C_β	28.2 (CH_2)	1.71 (1H, m), 1.61 (1H, m)	
	C_γ	23.1 (CH_2)	1.85 (2H, m)	
	C_δ	61.4 (CH_2)	4.05 (2H, m)	
	NOH	-	-	316.9
	NO	-	-	367.3

Table S10. Comparison of our NMR data of ^{1}H- and ^{13}C NMR spectra in DMSO-d_6 (^{1}H at 2.50 ppm, ^{13}C at 39.52 ppm, 600 MHz) of pandorachelin A (**1**) and the data of pandorachelin (**3**) detected by the Butler's group in DMSO-d_6 (^{1}H at 2.54 ppm, ^{13}C at 39.52 ppm, 500 MHz).

δ_C [ppm] (m)		δ_H [ppm]	
Pandorachelin A (**1**)	Pandorachelin (**3**) of Butler's group	Pandorachelin A (**1**)	Pandorachelin (**3**) of Butler's group
174.1	174.1	-	12.77 (COOH)
174.0	174.0	8.25	8.28
172.9	173.1	8.21	8.25
171.9	171.9	8.19	8.22
171.6	171.6	8.14	8.20
171.6	171.6	8.09	8.14
171.5	171.6	8.06	8.09
171.2	171.6	8.02	8.05
171.0	171.3	7.95	7.96
170.8	171.1	7.88	7.93
170.5	170.5	7.81	7.85
170.2	170.3	7.74	7.80
169.3	169.6	7.66	7.76
169.3	169.3	7.66	7.76
168.7	168.7	7.65	7.73
70.4	70.6	7.65	7.73
67.3	67.1	7.63	7.66
66.6	66.6	7.31	7.33
66.3	66.4	7.23	7.26
61.5	61.5	6.86	6.88
61.4	61.4	6.82	6.85
58.7	58.7	-	5.78 (OH)
58.6	58.7	-	5.31 (OH)
58.5	58.3	-	5.05 (OH)
58.2	55.4	-	5.02 (OH)
55.3	53.1	4.73	4.76
53.3	52.9	4.50	4.51
52.7	52.8	4.38	4.42
52.6	52.8	4.32	4.36
52.5	52.4	4.28	4.33
51.8	51.7	4.27	4.30
42.5	42.4	4.23	4.27
42.1	42.1	4.22	4.26
38.7	38.7	4.22	4.25
38.6	38.6	4.20	4.25
31.4	31.4	4.18	4.25
31.4	31.4	4.14	4.18
28.9	28.9	4.06	4.10
28.4	28.5	4.06	4.10
28.2	28.5	4.05	4.09
28.0	28.0	4.05	4.09
27.6	27.5	4.03	4.06

27.5	27.5	3.90	3.94
23.5	23.5	3.90	3.93
23.5	23.5	3.87	3.91
23.1	23.1	3.75	3.78
23.1	23.1	3.69	3.72
20.1	20.1	2.80	2.84
19.9	19.9	2.80	2.84
19.9	19.9	2.78	2.82
		2.78	2.82
		2.11	2.15
		2.11	2.15
		2.10	2.14
		2.10	2.14
		1.93	1.96
		1.89	1.91
		1.85	1.88
		1.85	1.88
		1.83	1.87
		1.83	1.87
		1.78	1.81
		1.76	1.81
		1.75	1.80
		1.74	1.80
		1.72	1.76
		1.71	1.76
		1.61	1.64
		1.59	1.64
		1.59	1.62
		1.59	1.62
		1.56	1.62
		1.55	1.62
		1.53	1.59
		1.53	1.59
		1.09	1.12
		1.09	1.12
		1.09	1.12
		1.04	1.08
		1.04	1.08
		1.04	1.08
		1.02	1.07
		1.02	1.07
		1.02	1.07

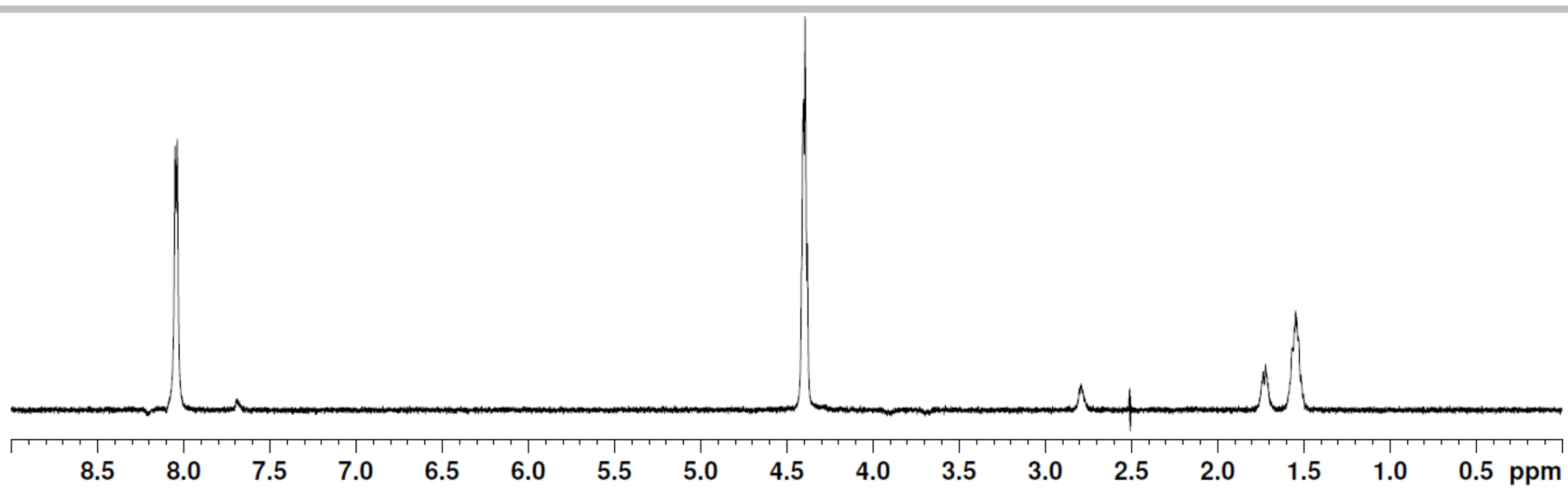

Figure S9. 1D-TOCSY spectrum of amide proton (L-ornitine 2) pandorachelin A (**1**) in DMSO-d_6 (600 MHz).

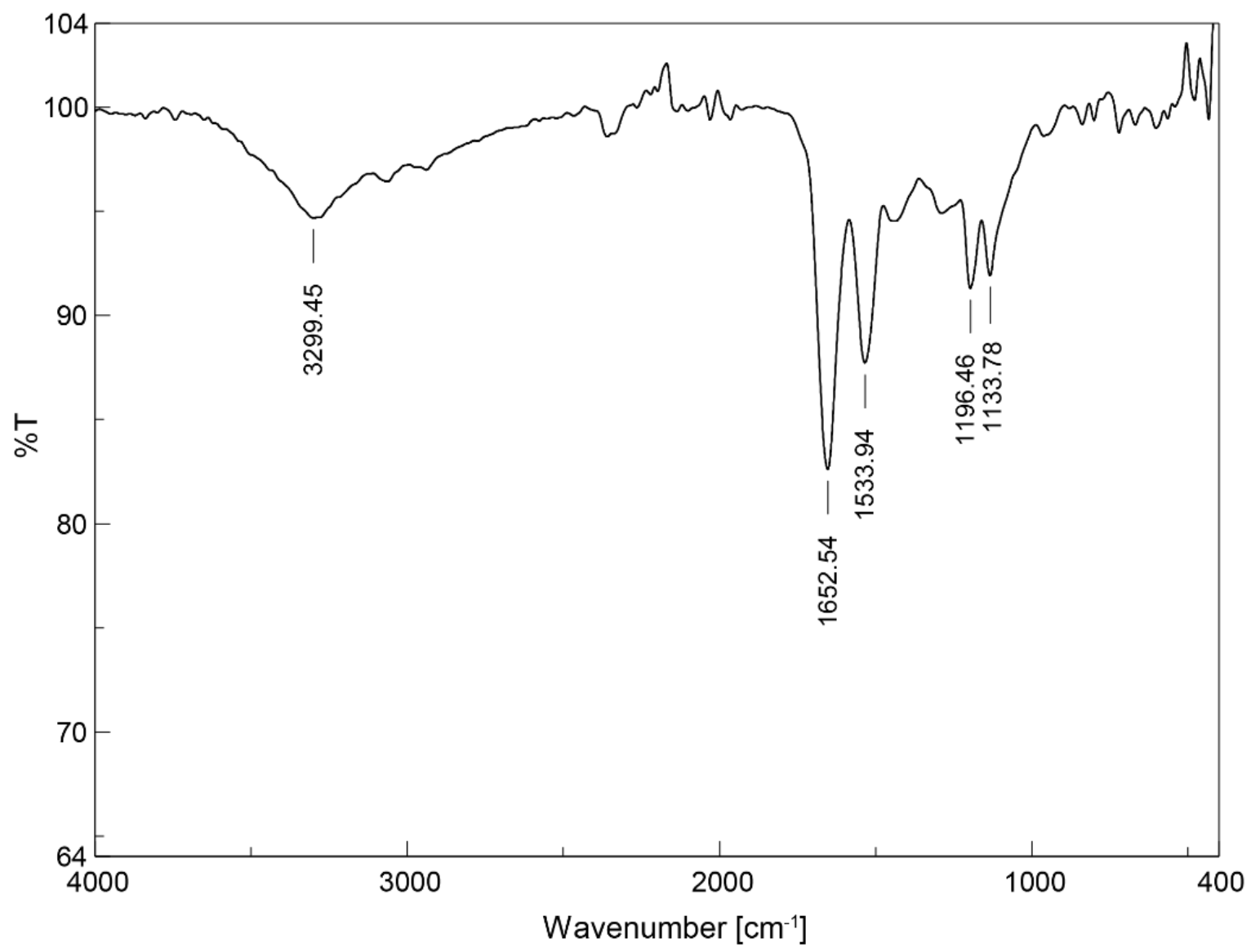

Figure S10. FTIR spectrum of pandorachelin A (**1**).

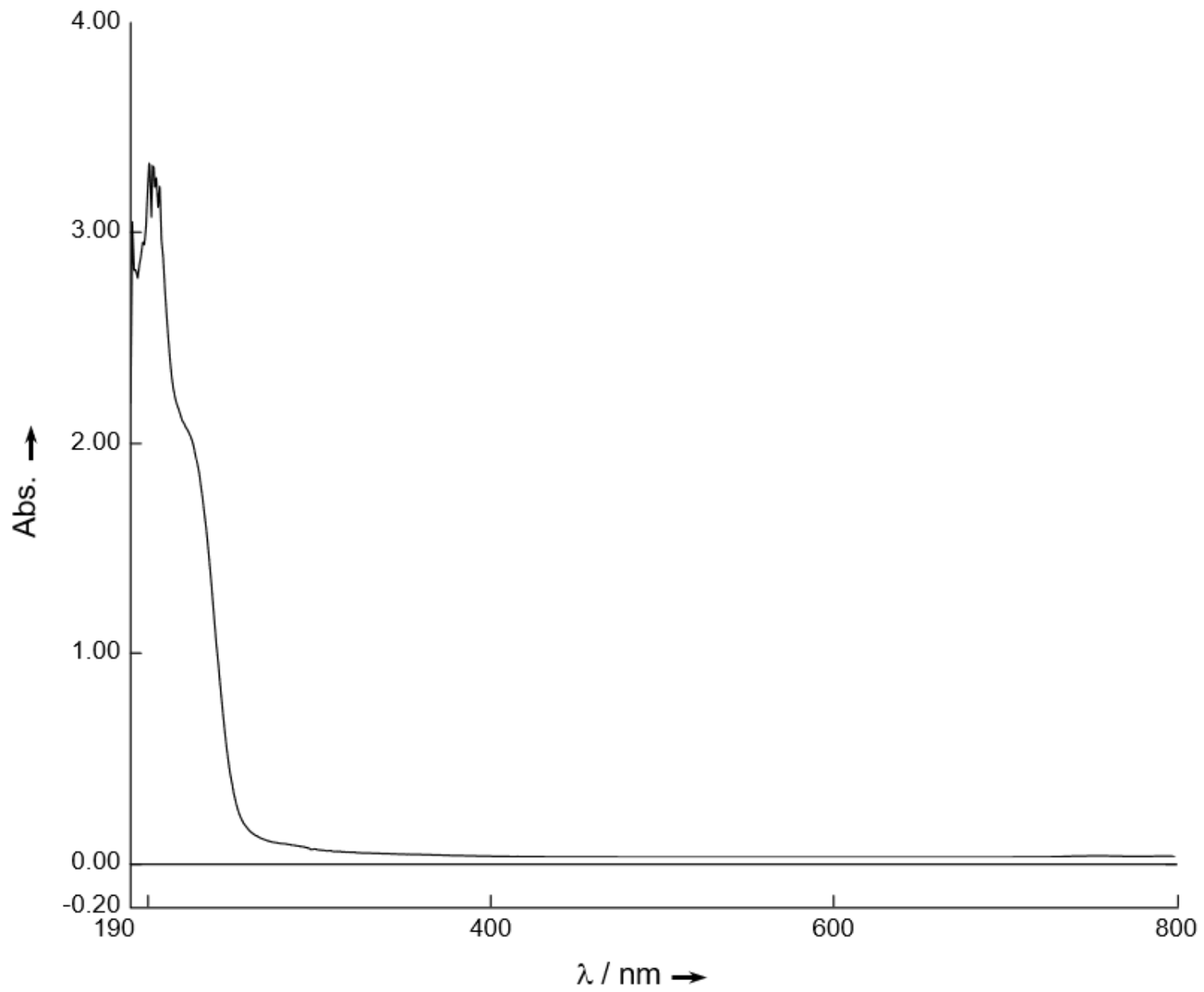

Figure S11. UV-Vis spectrum of pandorachelin A (**1**). Spectrum was measured at a concentration of 133 µg/mL in MeOH.

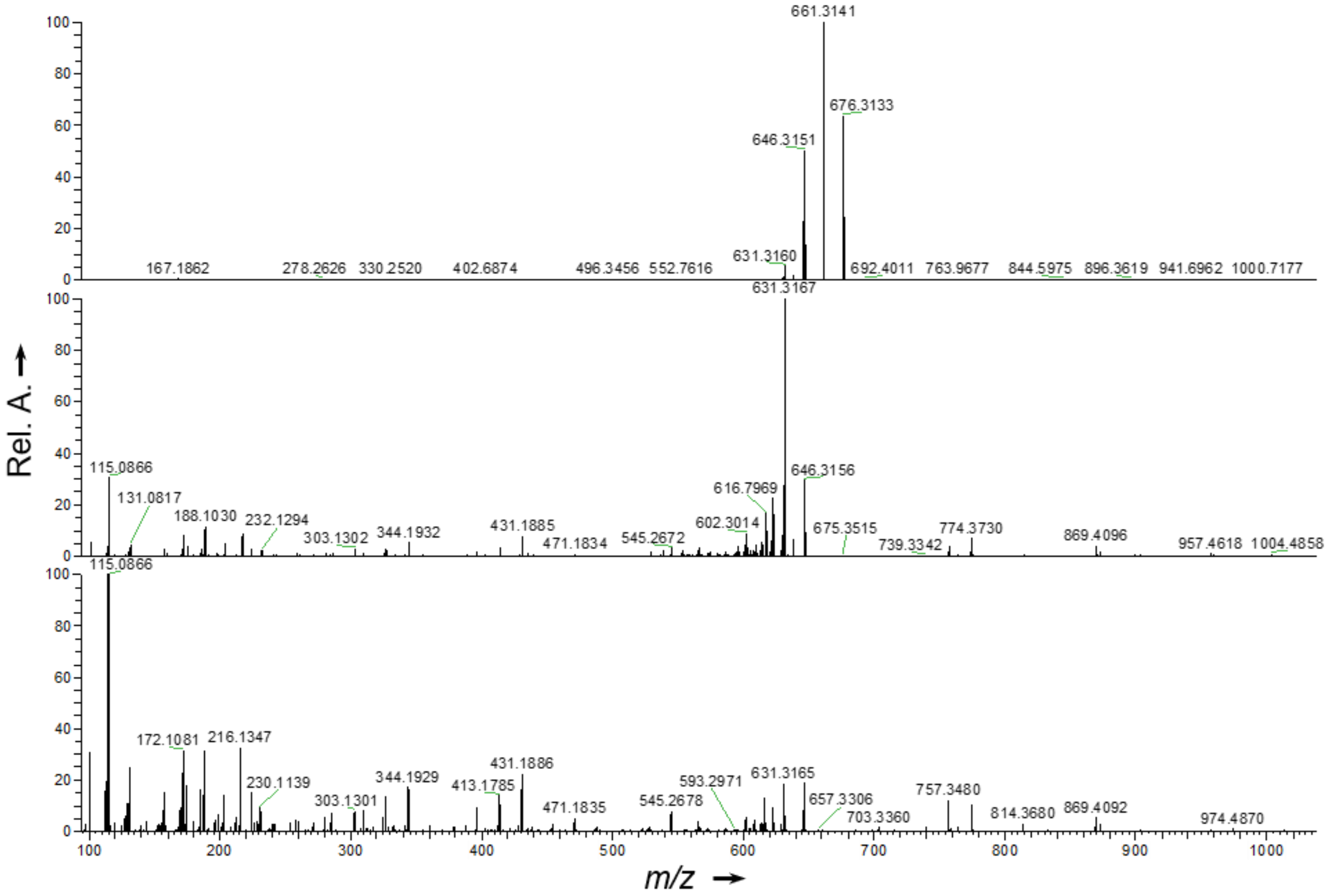

Figure S12. MS/MS (ESI$^+$) fragmentation of pandorachelin A (**1**) hcd 10 (top), hcd 25 (middle), and hcd 30 (bottom).

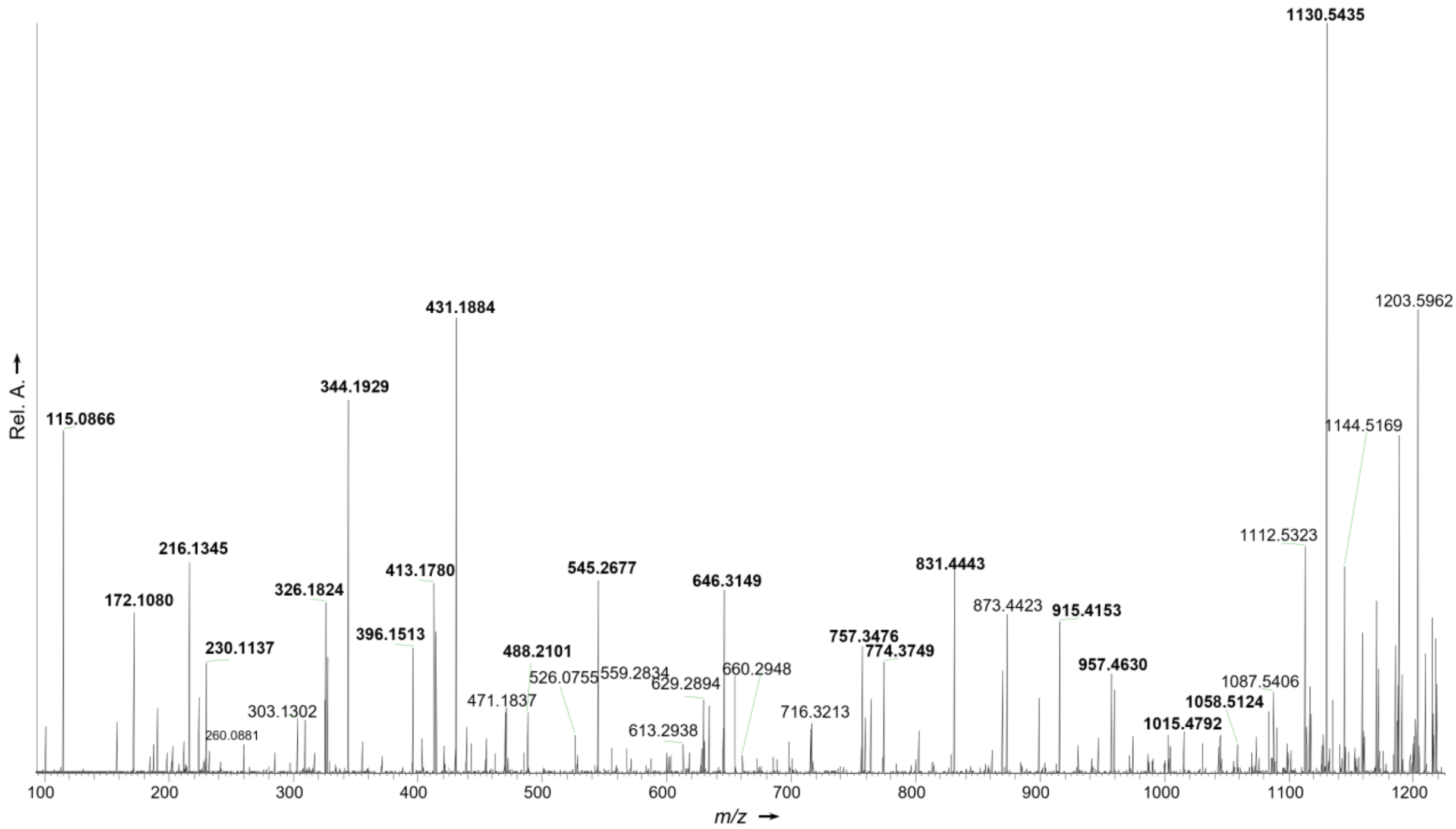

Figure S13. HR-ESI-MS/MS (ESI$^+$, hcd 30) spectra of pandorachelin A (**1**) at *m/z* 1351.6201 [M+H]$^+$. Bold ions highlight those shared with the dataset from Butler's group.

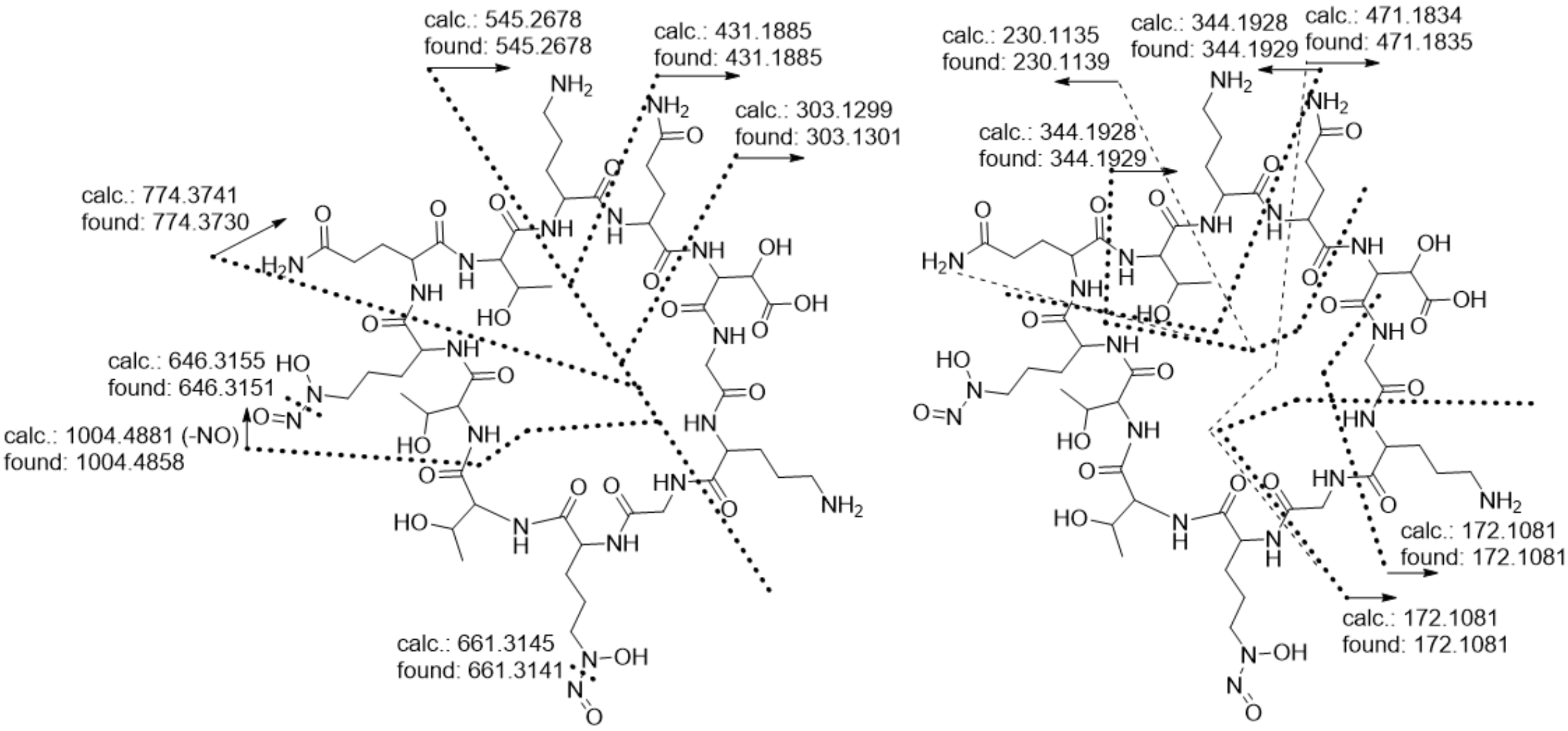

Figure S14. MS/MS fragmentation pattern of pandorachelin A (**1**).

Marfey's analysis of pandorachelin A (1)

Absolute configurations of amino acids from pandorachelin A (**1**) were determined by derivatization of the hydrolysate with 1-fluoro-2,4-dinitrophenyl-5-L-alanine amide (L-FDAA). **1** (1.0 mg) was hydrolyzed with 500 µL of 6 M HCl at 105 °C for 16 h. The resulting mixture of amino acids was freeze-dried and dissolved in 100 µL of water and 50 µL of 1 M $NaHCO_3$ was added. 10 µL of a 10 mg · mL^{-1} solution of L-FDAA in acetone was then added to the reaction solution. The reaction was stirred for 1 h at 40 °C. The same approach was applied to all references. To quench the reaction, 25 µL of 2 M HCl was added to the mixture. The reaction mixture was diluted with 25 µL methanol. A 1:4 dilution of the mixtures was analyzed using HPLC-HRMS with an elution gradient (solvent A: H_2O + 0.1% formic acid, solvent B: acetonitrile + 0.1% formic acid, time program: 5% B to 98% B in 21 min, 1.9 min 98% B, 98% B to 5% B in 0.1 min, 7 min 5% B, flow rate: 0.2 mL · min^{-1}). The elution order of D/L-*threo*-OH-Asp was shown before to be D→ L.[14]
To exclude epimerization during hydrolyzation, pandorachelin A (**1**) was treated with 500 µL of 6 M DCl at 105 °C for 16 h.
The absolute configuration of pandorachelin B (**4**) was determined using the same approach and is identical to the configuration of pandorachelin A (**1**).

Table S11. Retention times in min of reference compounds and pandorachelin A (**1**) hydrolysate.

	L-Thr	D-*allo*-Thr	L-Orn	L-*threo*-3-OH-Asp	D-Glu	L-Glu	D-Gra	L-Gra
Reference	7.52	7.87	6.22 and 6.57	6.33	8.25	7.95	9.02	8.71
Pandorachelin A (**1**) hydrolysate	7.51	7.86	6.22 and 6.59	6.32	8.24	7.96	9.02	8.71

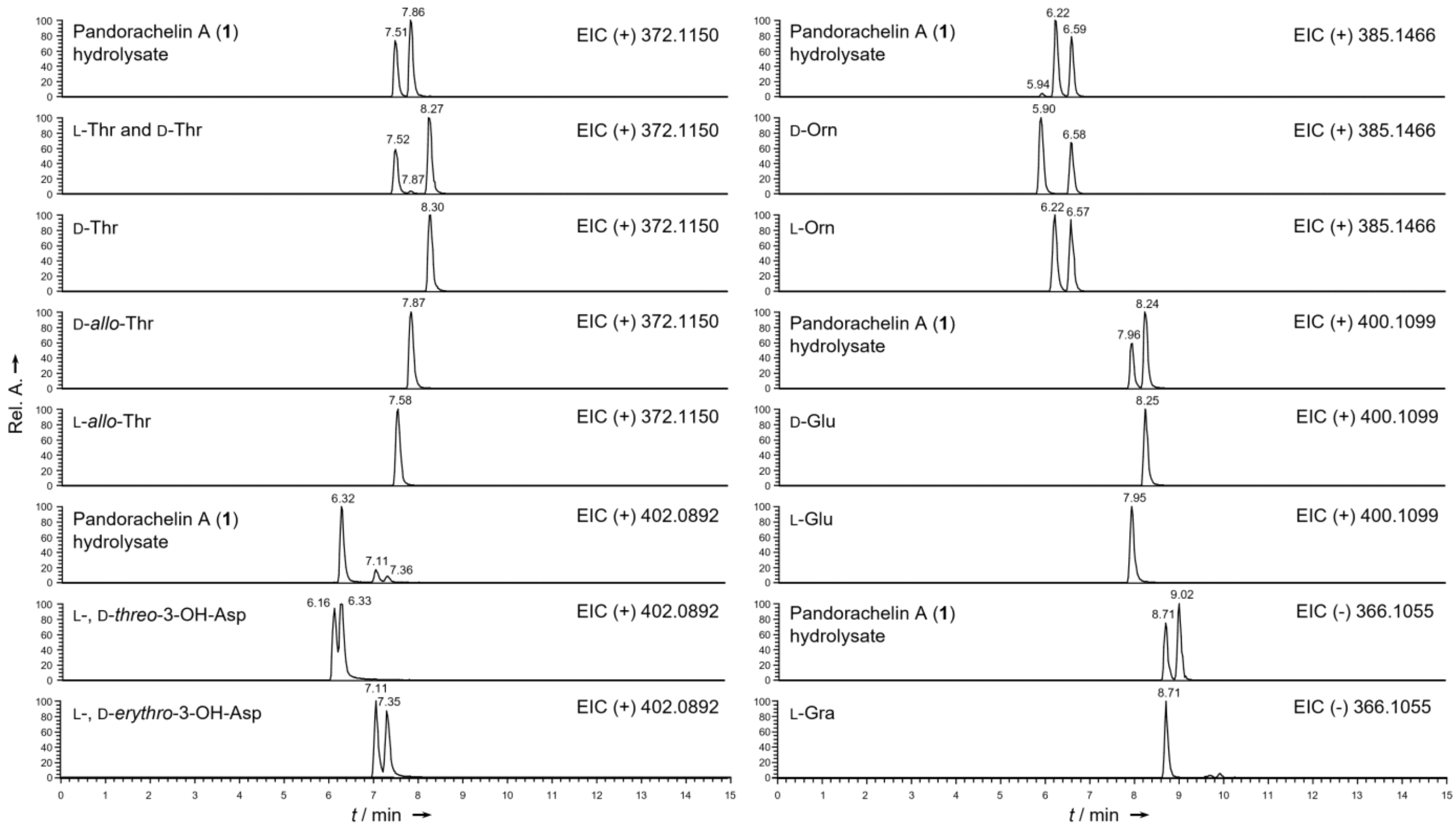

Figure S15. HPLC-HRMS (ESI+) (EIC, extracted ion chromatogram) of pandorachelin A (**1**) hydrolysate and reference compounds.

Preparation and isolation of ^{2}H-labeled pandorachelin A (1)

P. norimbergensis was grown as described above in a slightly modified minimal medium in which either L-threonine, L-arginine or L-glutamine along with L-glutamate was omitted, depending on the labeling experiment. For each of three labeling conditions, 200 mL main cultures with 200 mL were prepared in triplicate and each culture was supplemented with the corresponding isotopically labeled amino acids: 10 mg of L-arginine-2,3,3,4,4,5,5-d_7 or 20 mg of L-glutamine-2,3,3,4,4-d_5 or 20 mg of L-threonine-2,3-d_2. Prior to addition, the amino acids were dissolved in ddH_2O and sterile filtered with a 13 mm syringe filter (0.20 µm). Labeled pandorachelin A (**1**) was subsequently isolated and purified by preparative RP-HPLC as described above. Notably, the incorporation of L-arginine-2,3,3,4,4,5,5-d_7 and L-glutamine-2,3,3,4,4-d_5 in pandorachelin A (**1**) was inefficient.

MS/MS spectra of pandorachelin A (1) labeled with L-Thr-2,3-d_2

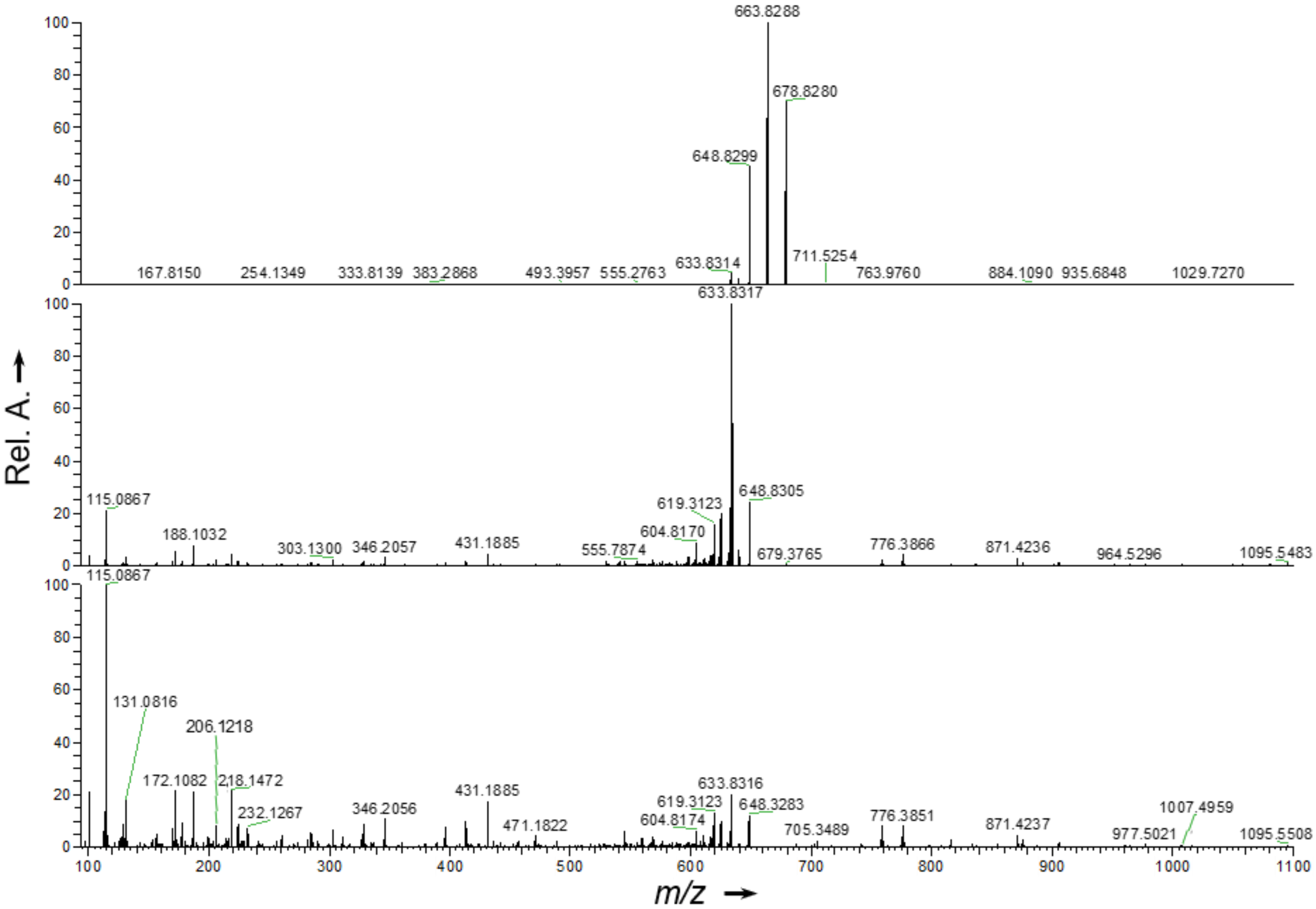

Figure S16. MS/MS (ESI$^+$) fragmentation of ^{2}H-labeled pandorachelin A (**1**) hcd 10 (top), hcd 25 (middle), and hcd 30 (bottom).

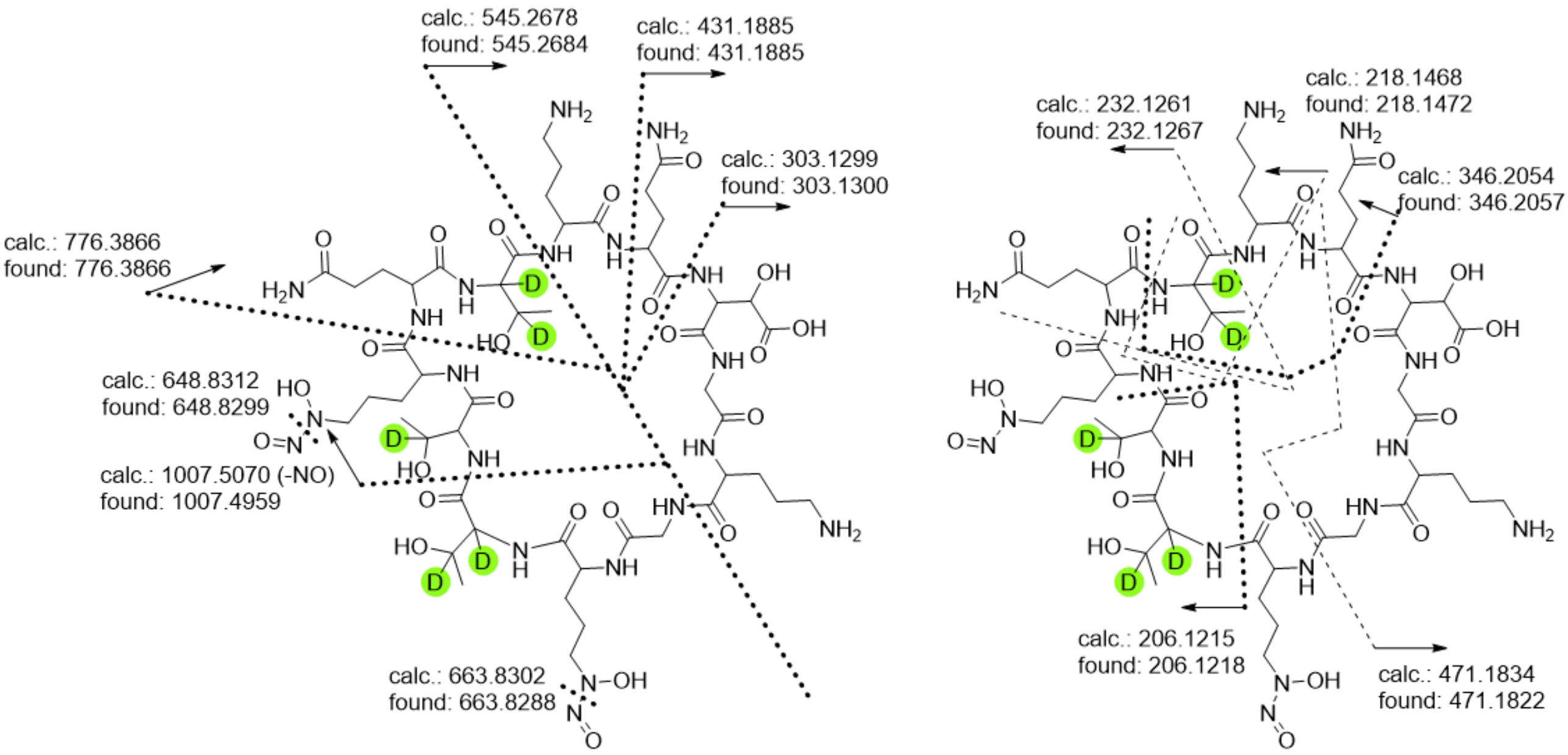

Figure S17. MS/MS fragmentation pattern of ^{2}H-labeled pandorachelin A (**1**).

Preparation and isolation of ^{15}N-labeled pandorachelin A (1)

1 L of *P. norimbergensis* was grown as described above in a slightly modified minimal medium in which $(NH_4)_2SO_4$ was exchanged with $(^{15}NH_4)_2SO_4$. ^{15}N-labeled pandorachelin A (**1**) was subsequently isolated and purified by preparative RP-HPLC as described above. Notably, ^{15}N was not fully incorporated in pandorachelin A (**1**).

Sanger's analysis of pandorachelin A (1)

1 mg pandorachelin A (**1**) was dissolved in 50 µL of water and 50 µL of acetone. After the addition of 50 µL of 1 M $NaHCO_3$, 1.48 µL (2.0 eq) of Sanger's reagent was added. The reaction was stirred for 1 h at 40 °C. L-Thr and Boc-L-Orn were treated the same way. The reaction mixtures were dried under a nitrogen flow. The derivatized pandorachelin A was hydrolyzed with 250 µL of 6 M HCl at 105 °C for 16 h and the derivatized Boc-L-Orn was hydrolyzed for 30 min. The samples were dried and analyzed using HPLC-HRMS.

Determination of the structure pandorachelin B (4)

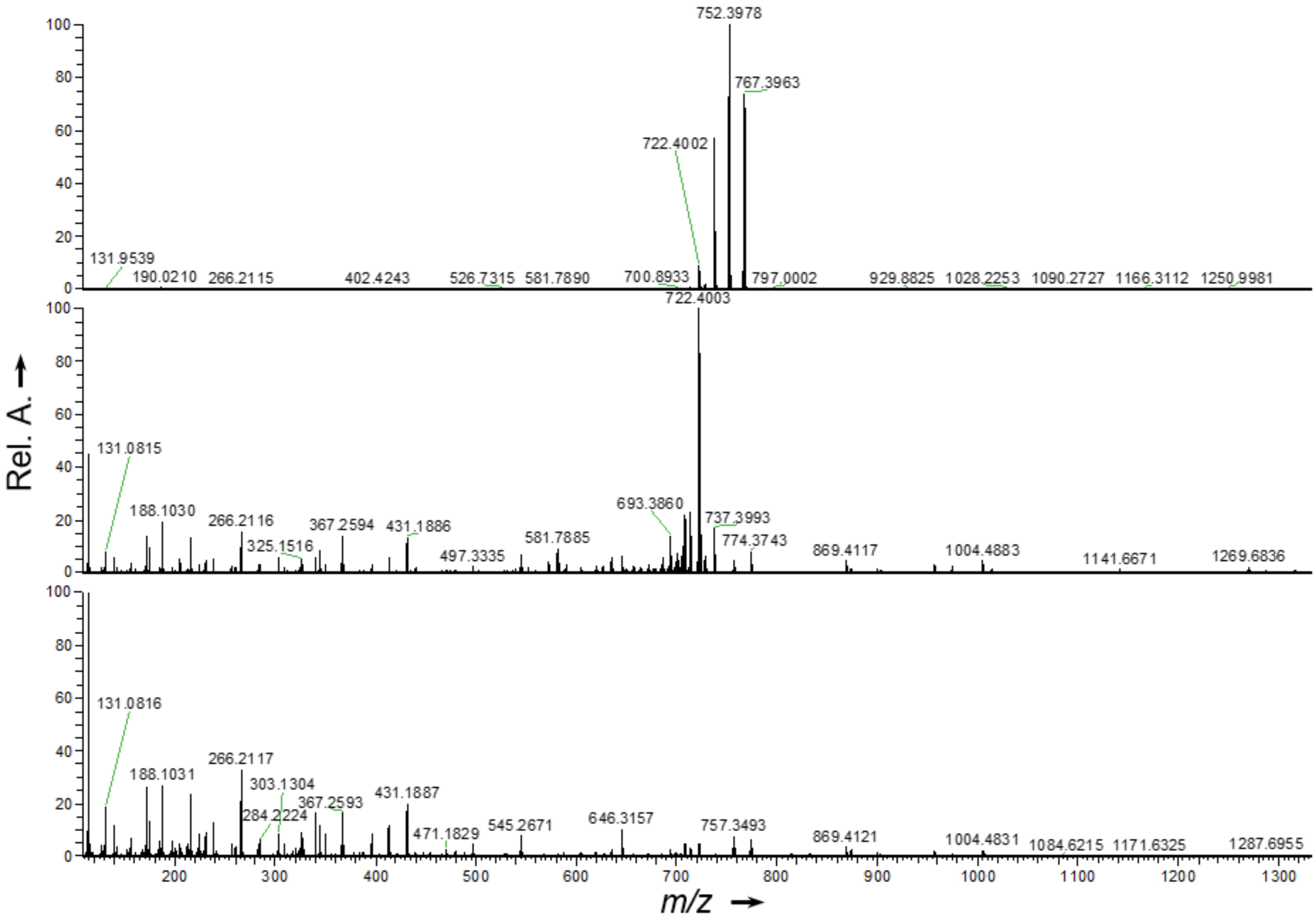

Figure S18. MS/MS (ESI⁺) fragmentation of pandorachelin B (**4**) hcd 10 (top), hcd 25 (middle), and hcd 30 (bottom).

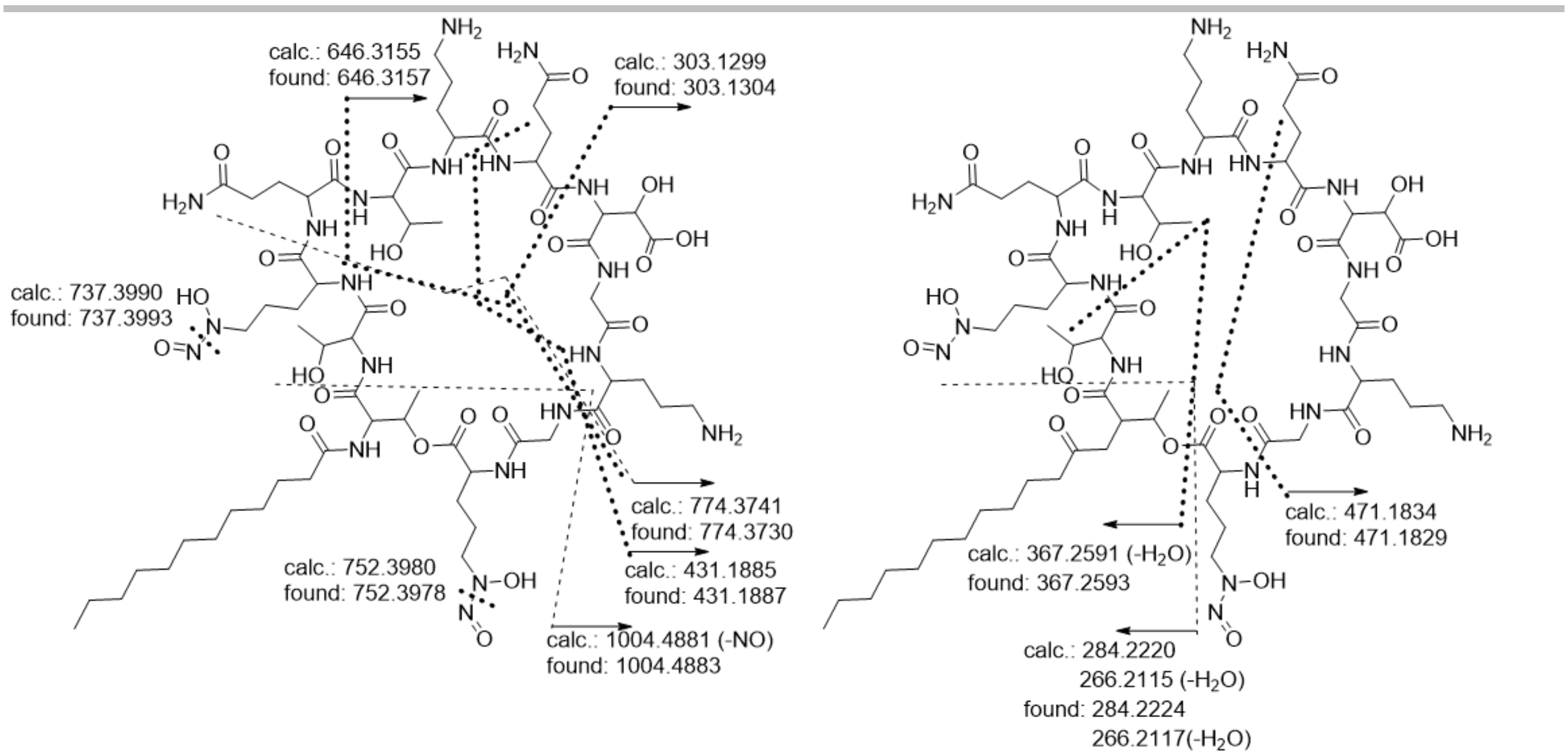

Figure S19. MS/MS fragmentation pattern of pandorachelin B (**4**).

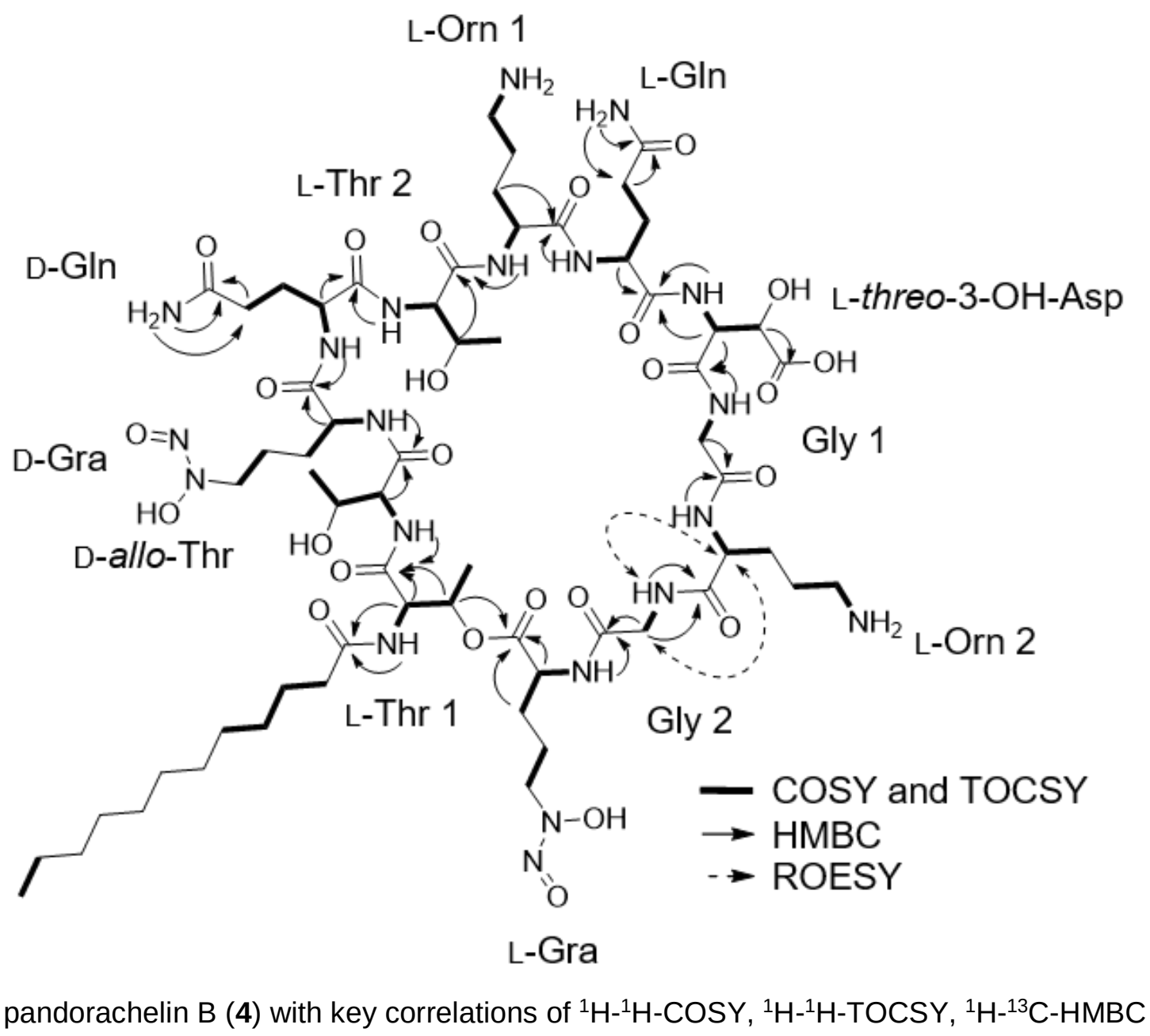

Figure S20. Structure elucidation of pandorachelin B (**4**) with key correlations of ^1H-^1H-COSY, ^1H-^1H-TOCSY, ^1H-^{13}C-HMBC and ^1H-^1H-ROESY.

Table S12. ^{1}H- and ^{13}C-NMR data of pandorachelin B (**4**) (600 MHz, DMSO-d_6).

Partial structure	Position	δ_C [ppm] (m)	δ_H [ppm] (m, J [Hz])
Dodecanoic acid	CO	172.8 (C)	-
	C_α	35.0 (CH_2)	2.24 (1H, m), 2.18 (1H, m)
	C_β	28.9 (CH_2)	1.75 (1H, m), 1.54 (1H, m)
	C_γ	25.3 (CH_2)	1.47 (2H, m)
	C_δ	29.1 (CH_2)^{i}	1.23 (2H, m)
	C_ε	29.1 (CH_2)^{i}	1.23 (2H, m)
	C_ζ	29.0 (CH_2)^{i}	1.23 (2H, m)
	C_η	28.8 (CH_2)^{i}	1.23 (2H, m)
	C_v	28.7 (CH_2)^{i}	1.23 (2H, m)
	C_ι	31.3 (CH_2)	1.23 (2H, m)
	C_κ	22.2 (CH_2)	1.26 (2H, m)
	C_λ	14.0 (CH_3)	0.84 (3H, t, 7.1)
L-Threonine 1	NH	-	7.98 (1H, d, 8.7)
	CO	168.5 (C)	-
	C_α	54.7 (CH)	4.65 (1H, dd, 8.6, 3.9)
	C_β	71.0 (CH)	5.07 (1H, m)
	C_γ	15.8 (CH_3)	1.10 (3H, d, 6.3)
D-*allo*-Threonine	NH	-	8.20 (1H, d, 7.5)
	CO	168.7 (C)	-
	C_α	58.4 (CH)^{i*}	4.23 (1H, m)
	C_β	67.5 (CH)	3.81 (1H, m)
	C_γ	20.3 (CH_3)	1.08 (3H, d, 6.0)
D-Graminine	NH	-	8.04 (1H, d, 7.5)
	CO	171.6 (C)	-
	C_α	51.7 (CH)	4.37 (1H, m)
	C_β	28.8 (CH_2)	1.74 (1H, m), 1.54 (1H, m)
	C_γ	23.5 (CH_2)	1.85 (2H, m)
	C_δ	61.2 (CH_2)	4.05 (2H, m)
D-Glutamine	NH	-	8.01 (1H, d, 6.4)
	CO	171.9 (C)	-
	C_α	52.2 (CH)	4.29 (1H, m)
	C_β	27.5 (CH_2)	1.74 (1H, m), 1.55 (1H, m)
	C_γ	31.4 (CH_2)	2.10 (2H, m)
	CO	173.8 (C)	-
	NH_2	-	7.29 (1H, s), 6.82 (1H, d, 5.4)
L-Threonine 2	NH	-	7.84 (1H, d, 7.7)
	CO	170.6 (C)	-
	C_α	58.6 (CH)^{i*}	4.22 (1H, m)
	C_β	66.4 (CH)	4.10 (1H, m)
	C_γ	20.0 (CH_3)	1.05 (3H, d, 6.3)
L-Ornithine 1	NH	-	7.92 (1H, br. s)
	CO	171.5 (C)	-
	C_α	53.2 (CH)	4.32 (1H, m)
	C_β	28.0 (CH_2)	1.74 (1H, m), 1.55 (1H, m)
	C_γ	23.5 (CH_2)	1.56 (2H, m)
	C_δ	38.5 (CH_2)	2.77 (2H, m)

	NH_2	-	7.72 (2H, m)
L-Glutamine	NH	-	8.25 (1H, m)
	CO	171.3 (C)	-
	C_α	52.7 (CH)	4.21 (1H, m)
	C_β	27.6 (CH_2)	1.91 (1H, m), 1.75 (1H, m)
	C_γ	31.4 (CH_2)	2.12 (2H, m)
	CO	174.1 (C)	-
	NH_2	-	7.24 (1H, br. s), 6.82 (1H, d, 5.4)
L-*threo*-3-OH-Aspartate	NH	-	7.62 (1H, d, 8.4)
	CO	169.4 (C)	-
	C_α	55.3 (CH)	4.71 (1H, m)
	C_β	70.4 (CH)	4.50 (1H, br. s)
	COOH	173.1 (C)	-
Glycine 1	NH	-	8.08 (1H, m)
	CO	171.1 (C)	-
	C_α	42.3 (CH_2)	3.85 (1H, m), 3.73 (1H, m)
L-Ornithine 2	NH	-	8.48 (1H, d, 6.3)
	CO	171.5 (C)	-
	C_α	52.8 (CH)	4.19 (1H, m)
	C_β	28.4 (CH_2)	1.76 (1H, m), 1.59 (1H, m)
	C_γ	23.2 (CH_2)	1.86 (2H, m)
	C_δ	38.6 (CH_2)	2.79 (2H, m)
	NH_2	-	7.72 (2H, m)
Glycine 2	NH	-	8.26 (1H, m)
	CO	168.9 (C)	-
	C_α	41.6	3.88 (1H, m), 3.71 (1H, m)
L-Graminine	NH	-	8.32 (1H, d, 7.0)
	CO	170.5 (C)	-
	C_α	51.2 (CH)	4.39 (1H, m)
	C_β	28.1 (CH_2)	1.79 (1H, m), 1.62 (1H, m)
	C_γ	22.8 (CH_2)	1.74 (2H, m)
	C_δ	61.4 (CH_2)	4.06 (1H, m)

[i] interchangeable signals
[i*] interchangeable signals

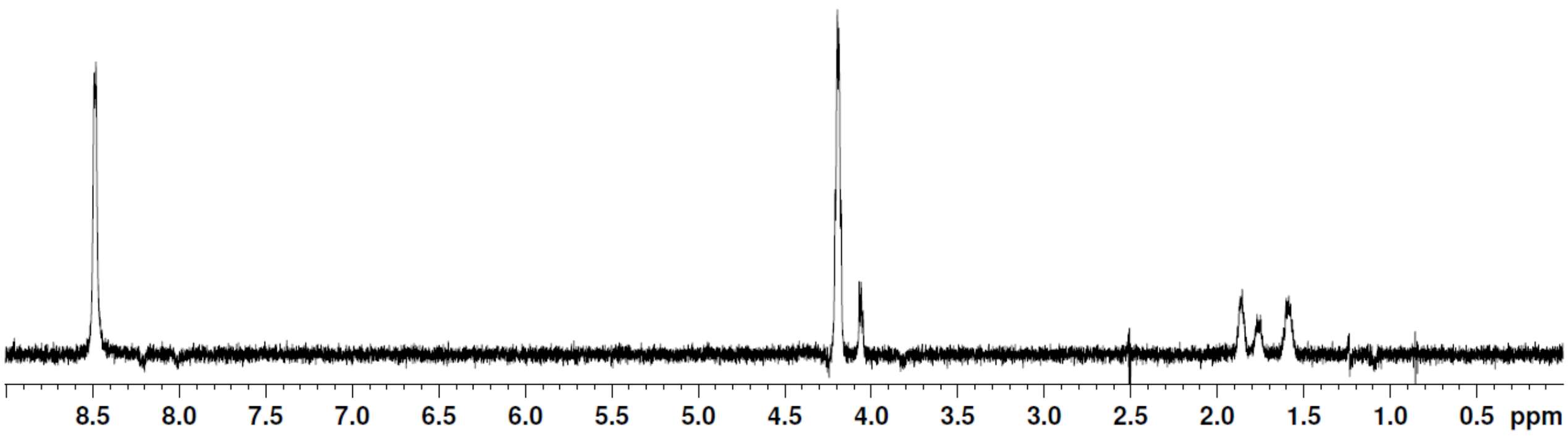

Figure S21. 1D-TOCSY spectrum of amide proton (L-ornitine 1) pandorachelin B (**4**) in DMSO-d_6 (600 MHz).

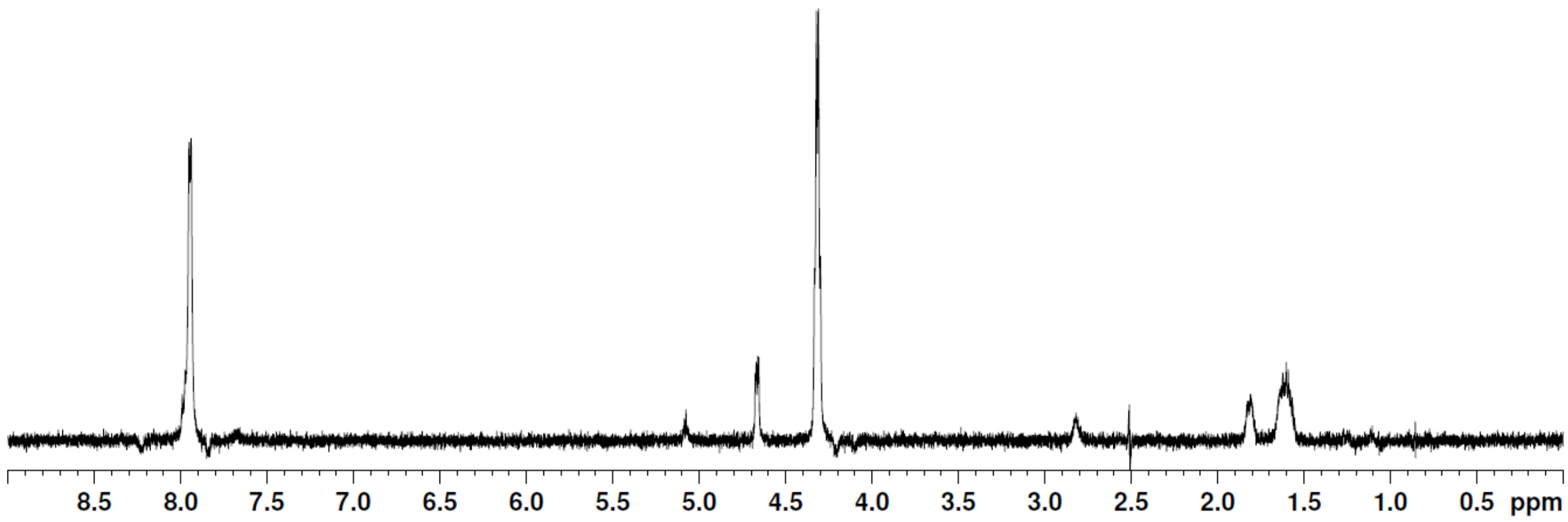

Figure S22. 1D-TOCSY spectrum of amide proton (L-ornitine 2) pandorachelin B (**4**) in DMSO-d_6 (600 MHz).

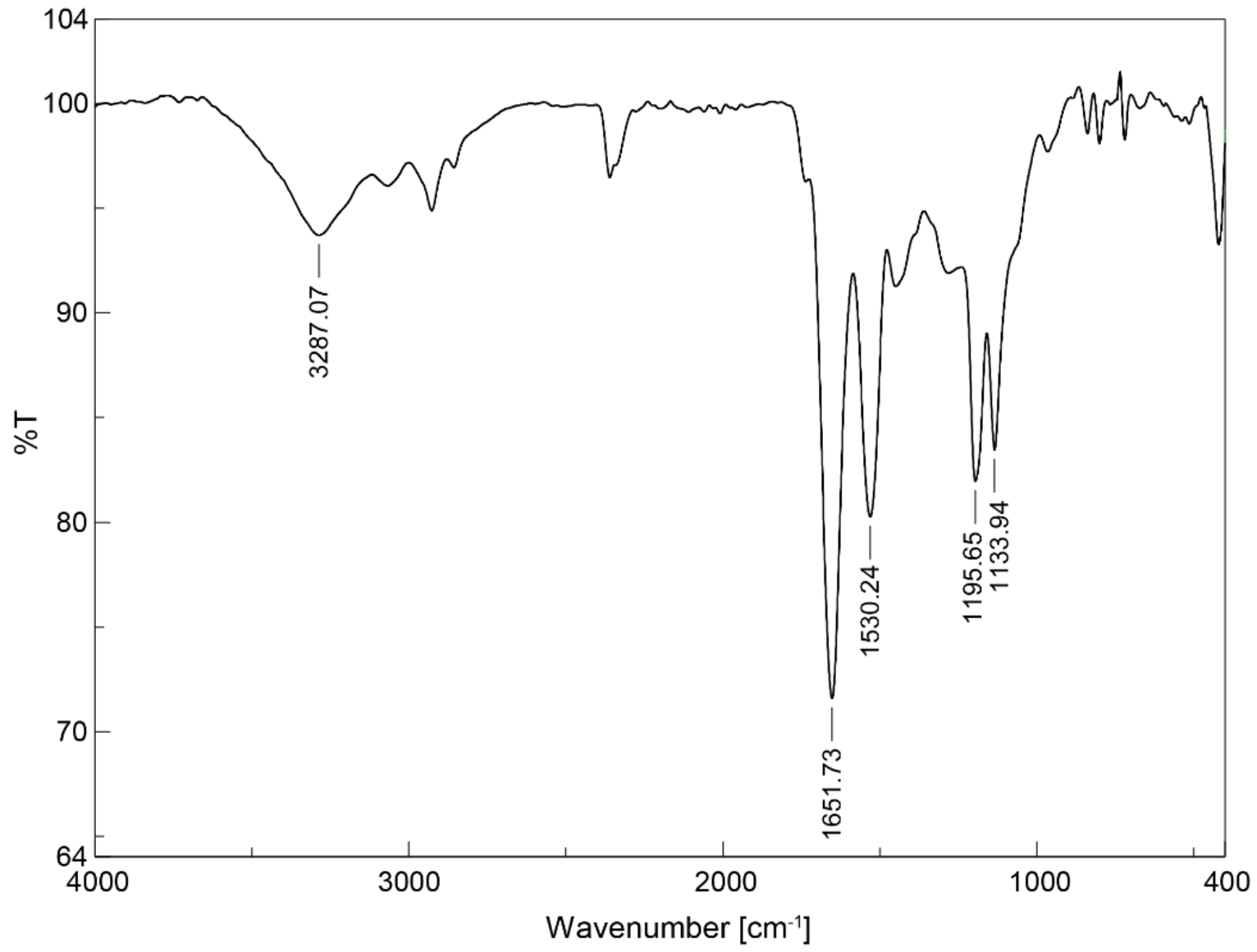

Figure S23. FTIR spectrum of pandorachelin B (**4**).

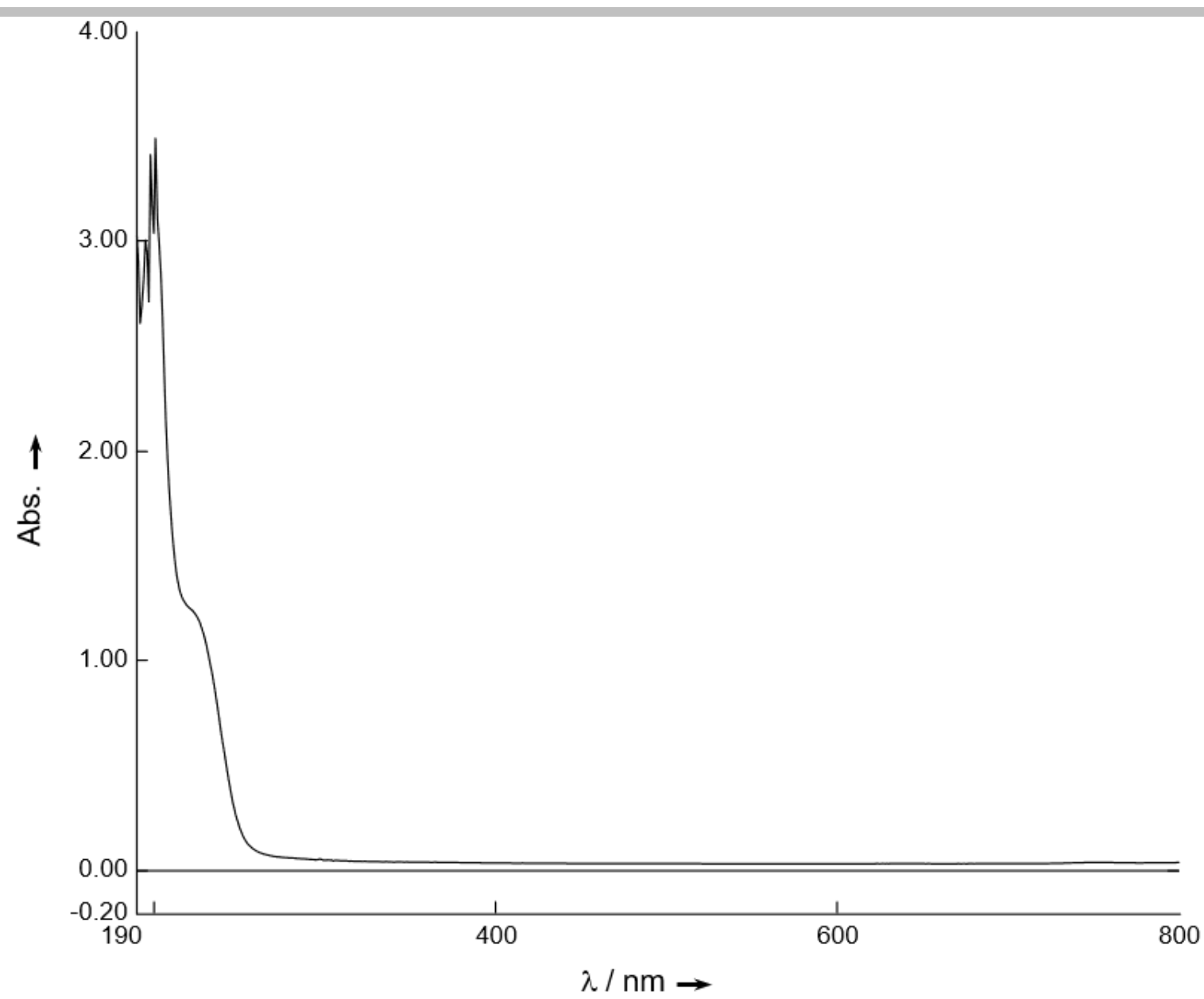

Figure S24. UV-Vis spectrum of pandorachelin B (**4**). Spectrum was measured in MeOH with a concentration of 95 µg/mL.

Basic hydrolysis of pandorachelin B (4)

0.1 mg of pandorachelin B (**4**) was dissolved in 50 µL of H_2O, then 30 µL of $NaHCO_3$ (1 M) was added. The reaction was measured after 30 min, 3 h and 24 h.

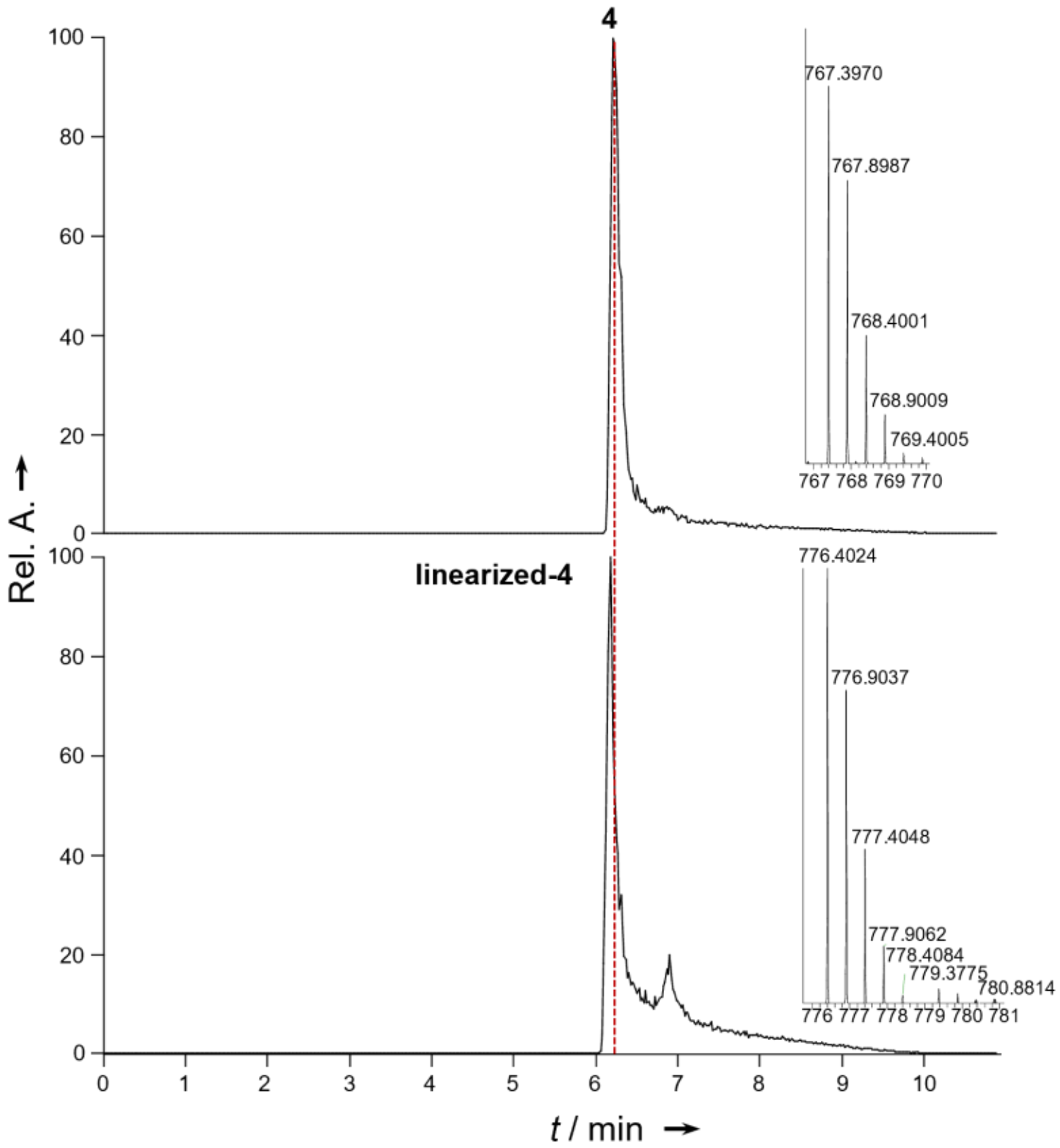

Figure S25. HPLC-HRMS (ESI+) (EIC, extracted ion chromatogram) of the hydrolysis of pandorachelin B (**4**) (*m/z* 767.3970 $[M+2H]^{2+}$) with $NaHCO_3$ (1 M) to linearized **4** (*m/z* 776.4023 $[M+2H]^{2+}$) after 24 h.

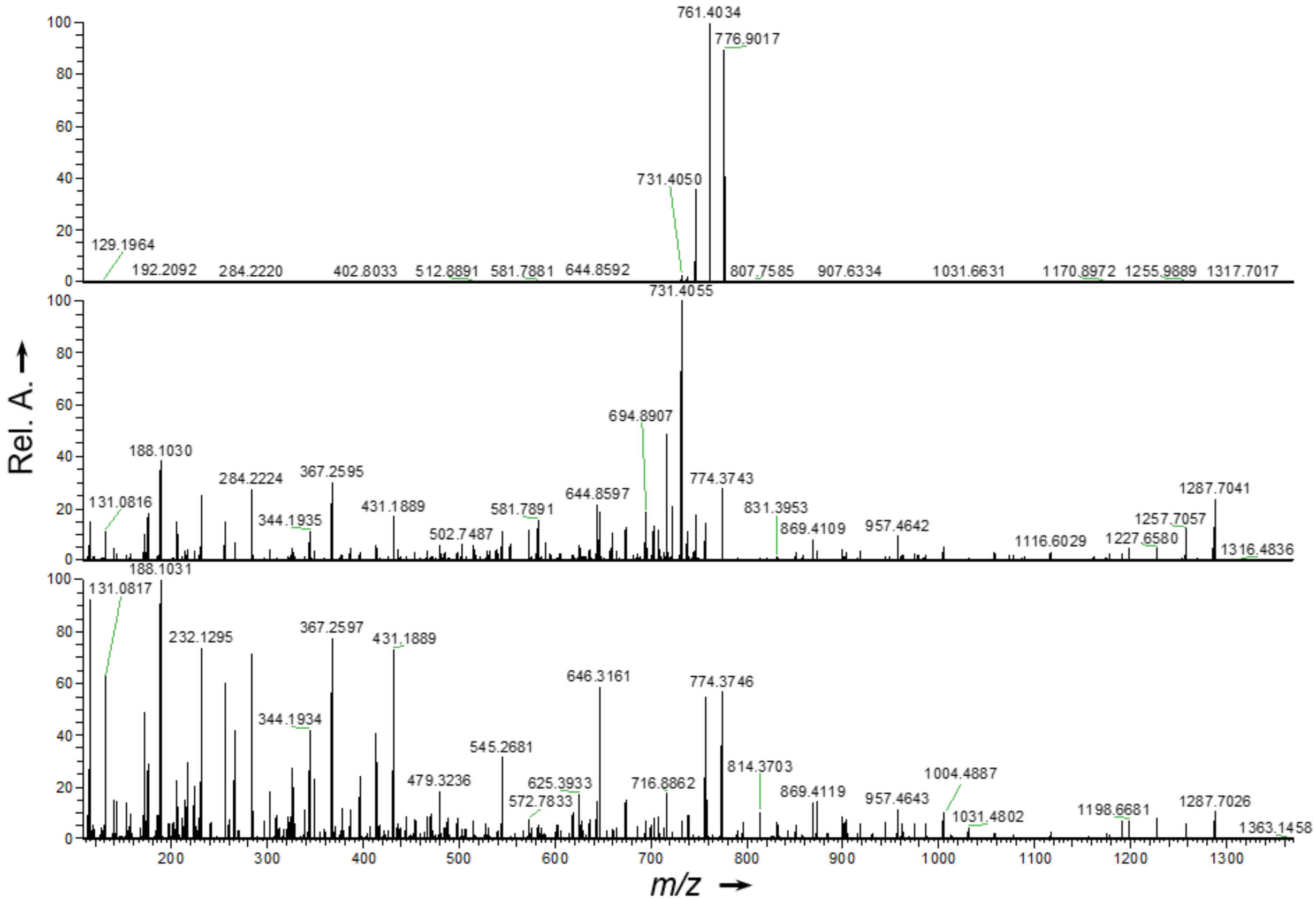

Figure S26. MS/MS (ESI$^+$) fragmentation of linearized pandorachelin B (**4**) hcd 10 (top), hcd 25 (middle), and hcd 30 (bottom).

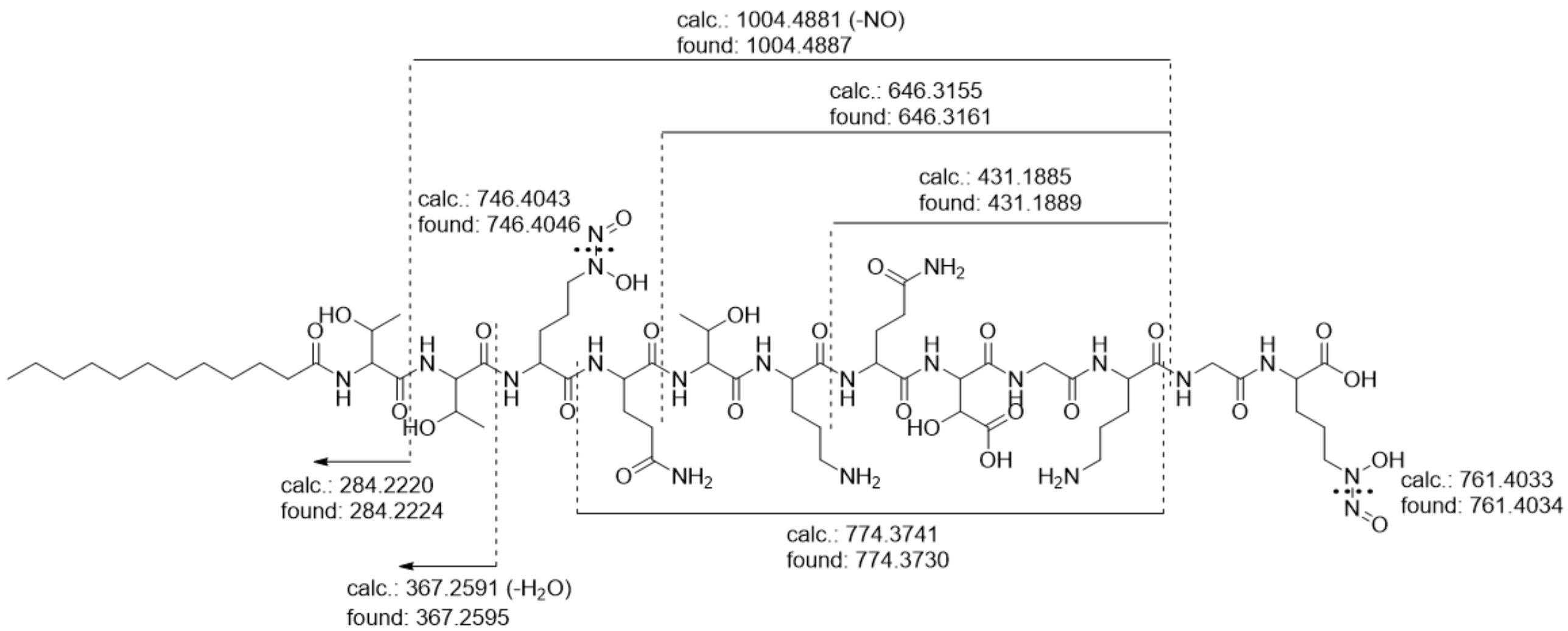

Figure S27. MS/MS fragmentation pattern of linearized pandorachelin B (**4**).

Alignment of the amino sequences of the FAAL domains

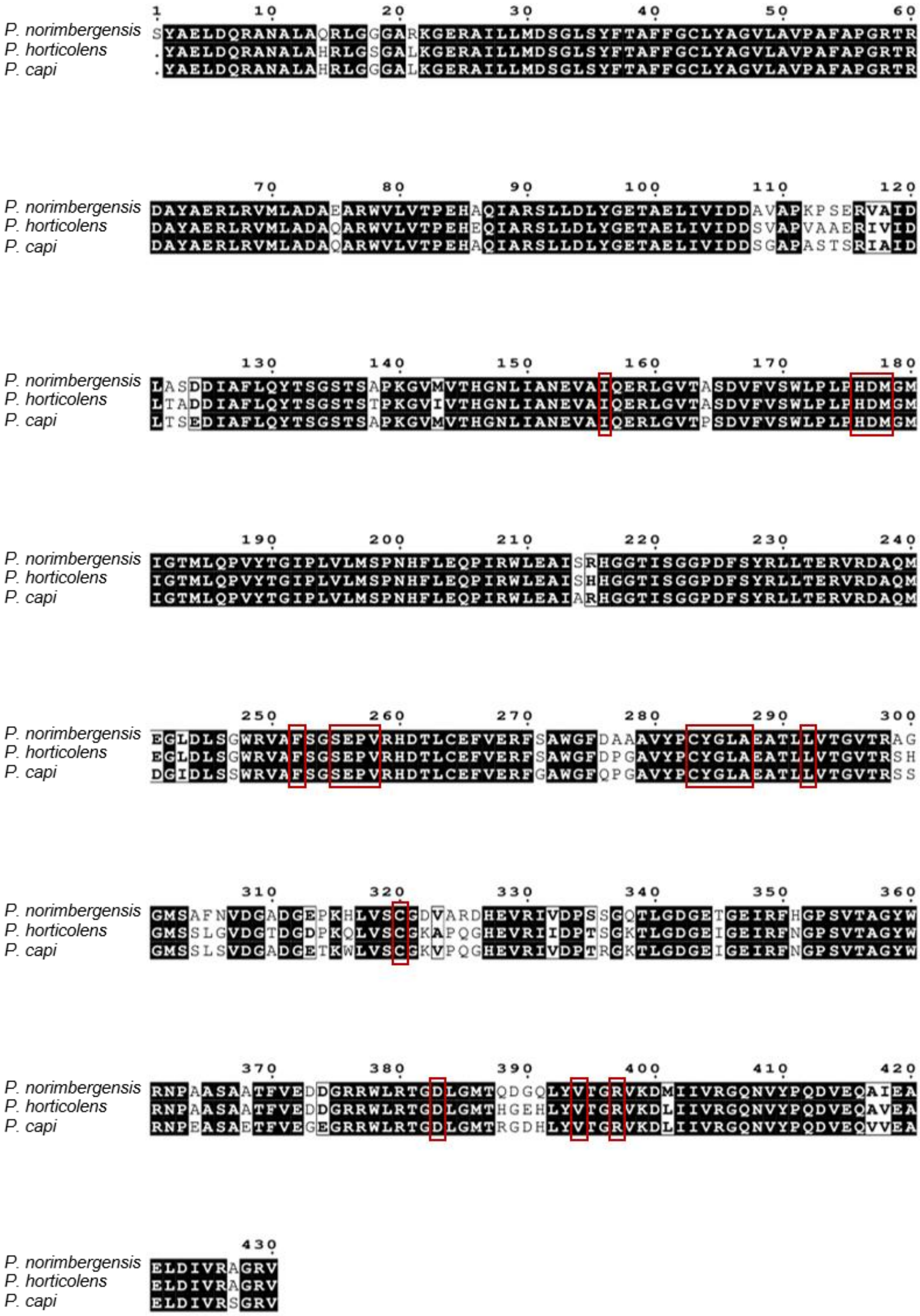

Figure S28. Alignment of amino acid sequences of the FAAL domain in PdnI from *P. norimbergensis*, *P. horticolens* and *P. capi*. The sequence alignment was done using MAFFT 7 and visualized by ESPript 3.0. The active sites were identified using the Conserved Domain Database (CDD) from NCBI and highlighted in red.

Biotransformation of pandorachelin B (4) by *P. norimbergensis* Δ*pdnI*

1.9 mL cultures of *P. norimbergensis* Δ*pdnI* was cultured overnight at 30 °C in MM9 medium supplemented with trace elements and tetracycline (60 µg · mL^{-1}), with orbital shaking at 110 rpm. A solution of pandorachelin B (**4**) in ddH_2O was prepared (2 mM) and after 4 h of incubation, 100 µL of the sterile solution was added to the bacterial culture (100 µM final concentration). Cultures were incubated for 24 h, then extracted with Amberlite® XAD16 resin as described above. The cultivation was performed in technical triplicates.

Bioinformatic analysis of PdnM

Table S13. Proteins that are homologous to PdnM of *Pandoraea norimbergensis*. All homologs are encoded within a putative BGC resembling *pdn* from *P. norimbergensis*.

Strain	Identity	Accession No.
Pandoraea norimbergensis	–	WP_058378715.1
Pandoraea sp. PE-S2T-3	90%	WP_087725820.1
Pandoraea sp. PE-S2R-1	90%	WP_087687507.1
Pandoraea iniqua LMG31009	89%	WP_150790397.1
Pandoraea iniqua LMG31115	89%	WP_150685447.1
Pandoraea horticolens LMG31112	80%	WP_150621410.1
Pandoraea capi	75%	WP_242559656.1
Pandoraea sp. ISTKB	74%	WP_083253799.1
Caballeronia sp. DA-9	55%	WP_438392377.1
Caballeronia sp. LZ035	55%	WP_309907941.1
Caballeronia sp. LZ065	55%	WP_309836368.1
Chitinasiproducens palmae JS23	52%	WP_211435270.1

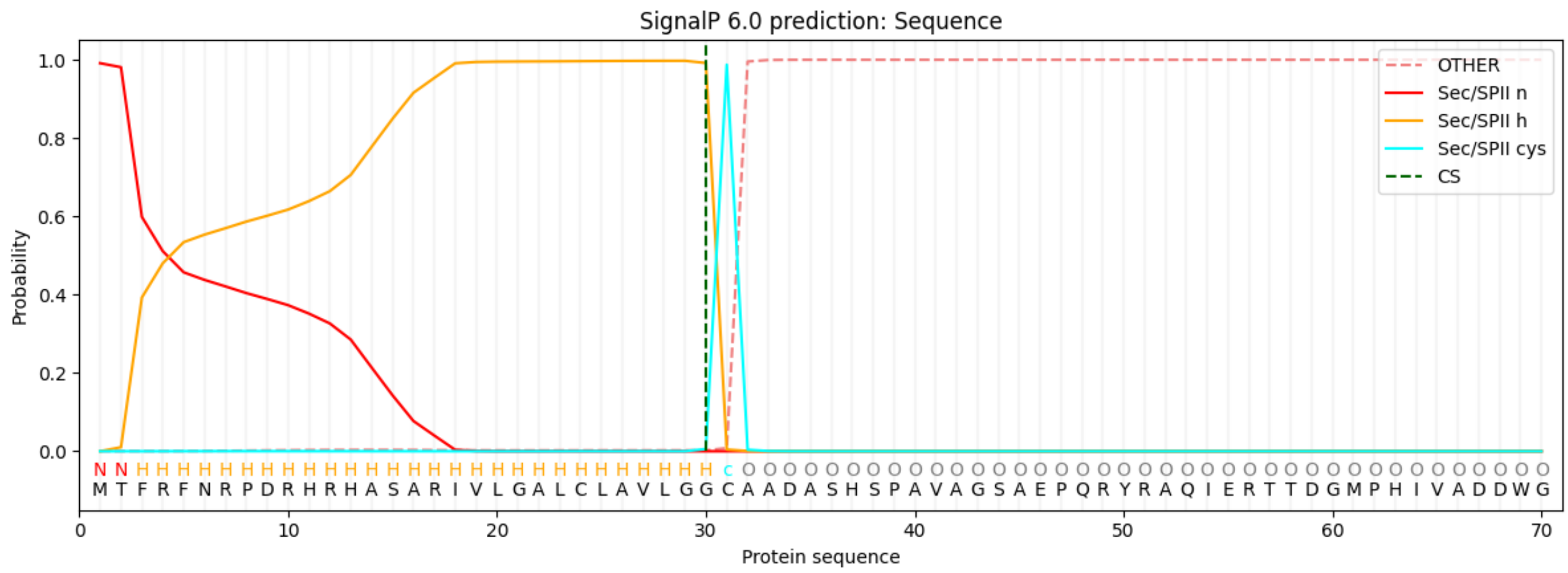

Figure S29. Prediction of signal peptide sequence by SignalP 6.0.[19] Sequence prediction analysis indicated that PdnM possesses a lipoprotein signal peptide (Sec/SPII) and a cleavage site between pos. 30 and 31 with a probability of 0.991783.

```
                        Signal sequence
PVDQ_PSEAE      --------MGM-----RTVLTGLAGMLLGSMMPVQADM----------PRPTGLAADIRW 37
Pcapi_PdnM      MTLRISRTVNPRKFLTLSVSGALCLAILGACATDTSHSPGSASEGS----AQSYRARIER 56
Pnori_PdnM      MTFRFNRPDRHRHASARIVLGALCLAVLGGCAADASHSPAVAGSAEP----QRYRAQIER 56
Photi_PdnM      MTFRIKRSAGHRTILASPALGTLCLAVLGGCTADASHSPAVASHVESQRDRTRYRALIER 60
                                  .   *.  :**.     :.                 * *.
                                             (1)
PVDQ_PSEAE      TAYGVPHIRAKDERGLGYGIGYAYARDNACLLAEEIVTARGERARYFGSEGKS-----SA 92
Pcapi_PdnM      TADGVPHVVANDWGSLGWGAAYVQAQDDLCTMAQAFVTYRGERSRYFGAQGQQPTASTLG 116
Pnori_PdnM      TTDGMPHIVADDWGSLGWGAAYVQAQDDLCTMAQSFVTYRGERSRYFGAKGTQPTDSTLG 116
Photi_PdnM      TTDGVPHVVANDWGSLGWGTAYVQAQDDLCTMAQAFVTYRGERSRYFGAQGTQPTATTLG 120
                *: *:**: *.*  .**:* .*. *:*: * :*: :** ****:****::* .      .

PVDQ_PSEAE      ELDNLPSDIFYAWLNQPEALQAFWQAQTPAVRQLLEGYAAGFNRFLREAD--------GK 144
Pcapi_PdnM      ELDNLDSDFFFRFVADDARLQAYRDAQTPQFRALVDGFAAGYNRYLNDWQRGGASGVGPA 176
Pnori_PdnM      DIDNLDSDVFFRLVADDARLQAYREAQTPQFRELVDGFAAGYNRYLQDWQRNK---TAPS 173
Photi_PdnM      DVGNLDSDFFFRFVADDTRLQAYRAAQTPQFRELVEGFAAGYNRYLNDWQRNK---TASS 177
                ::.** **.*:  : :    ***:  **** .* *::*:***:**:*.: :
                  (1)
PVDQ_PSEAE      TTSCLGQPWLRAIATDDLLRLTRRLLVEGGVGQFADALVAAAPPGAEKVALSGEQAFQV- 203
Pcapi_PdnM      HAACRNTPWVTTISPEDVYRRLFAANLAGGMARFAGRVVSAKPPGNAPASPQAQDGAAST 236
Pnori_PdnM      HAACRSEPWVTAISPDDVYRRLFAANLAGGMARFAGRVASAMPPGKTSGVLQPQGSTSPV 233
Photi_PdnM      HAACRSEPWVTAITPEDVYRRLFAANLAGGMARFAGRVASAIPPDTPSSAPQRQSSGASI 237
                 ::* . **: :*: :*: *       : **:.:**. :.:* **.       . : .
                                    S                      H
PVDQ_PSEAE      --AEQRRQRFRL----ERGSNAIAVGSERSA------DGKGMLLANPHFPWNGAMRFYQM 251
Pcapi_PdnM      DKLALRPGAFDVGGGRGIGSNALAFGAQGSARGTSGQGGASVLLGNPHWFLEGPDRFYQM 296
Pnori_PdnM      EKLALREGELDVGGGRGIGSNALAFGAQGSA------GAQSVLLGNPHWFLEGPDRFYQM 287
Photi_PdnM      EKLALRAGEFEVGGGRGIGSNALAFGAQGSARSPDGKAAQSVLLGNPHWFLEGPDRFYQM 297
                    *   : :      ****:*.*:: **        . .:**.***:  :*  *****
                                                    V
PVDQ_PSEAE      HLTIPGRLDVMGASLPGLPVVNIGFSRHLAWTHTVDTSSHFTLYRLALDPKDPRRYLVDG 311
Pcapi_PdnM      RLTLPGQLDVSGVSFLGVPVVMIGFNHNVAWTHTVSAARRFGLFQLSLAPDSDTTYRVDG 356
Pnori_PdnM      RLTVPGQLDVSGVSFLGVPVVMIGFNHDIAWTHTVSAARRFGLFQLSLAPNSDTTYLVDG 347
Photi_PdnM      RLTMPGQLDVSGASFLGVPVVMIGFNHNIAWTHTVSAARRFGLFQLSLAPDSDTTYLVDG 357
                :**:**:*** *.*: *:*** ***.:.:******.:: :* *::*:* *..   * ***

PVDQ_PSEAE      RSLPLEEKSVAIEVRGADGKLSRVEHKVYQSIYGPLV---------VWPGKLDWNRSEAY 362
Pcapi_PdnM      KPVAMTPVVVTVPVRESDGTVRPHTRTLYRTALGPVVNLASLSPALQWRATAPGNPGLAF 416
Pnori_PdnM      KPVAMTPVTITVPVREADGTVHPRSRTLYRTALGPVVNLGAMSPALQWRAGADGKPGQAF 407
Photi_PdnM      KPVAMTPVAITVPVRETDGTVHPRMRTLYRTAMGPVVNLGAMSPALQWRAAAQGKPGQAF 417
                : : :    ::: ** :**.:    .:*::  **:*            * .    : . *:

PVDQ_PSEAE      ALRDANLENTRVLQQWYSINQASDVADLRRRVEALQGIPWVNTLAADEQGNALYMNQ-SV 421
Pcapi_PdnM      VIRDINAENYRVFANFLAWGRASSLDEFVGIARREAATPWVNTVAIGRDDPRVWFGDVGA 476
Pnori_PdnM      VIRDINADNYRVFANFLAWGRATSLDDFVGIARREAATPWVNTVAIGRDDPRVWFGDVGA 467
Photi_PdnM      VIRDINADNYRVFANFLAWGRATSLDEFVKIARRDAATPWVNTVAIGRDDPRVWFGDVGA 477
                .:** * :* **: :: .:*:.: ::   ..   . *****:* ..:.  ::::.: ..
                          (2)                          (2)
PVDQ_PSEAE      VPYLKPELIPACAIPQ-----LVAEGLPALQGQDSRCAWSRDPAAAQAGITPAAQLPVLL 476
Pcapi_PdnM      MPNVPDSLAARCTPPLGKALASQVPGVPFLDGSRSTCDWQTSPGAAQAGALPADAMPQMF 536
Pnori_PdnM      VPNVPDALAAQCTPPLGKAFAAQAPGVPFLDGSRSACGWQSSPKAAQAGALPADAMPQLF 527
Photi_PdnM      IPNVPDTLAERCIPPLGKAFAAQAPGVPFLDGARSACNWPSSPDAAQAGALPADAMPQMF 537
                :* :  *   *  *        . *:* *:* * * * * .* *****  **  :* ::
                        N
PVDQ_PSEAE      RRDFVQNSNDSAWLTNPASPLQGFSPLVSQEKP---IGPRARYAL--SRLQGKQPLEAKT 531
Pcapi_PdnM      RNDYVANMNNTHWIVNPAQPLTGFAAVTGQERTEMGLRPRLGFRIAEQRMRGSDGLGAPG 596
Pnori_PdnM      RHDYVANMNNTHWIVNPAQPLTGFAAVTGGERSEMGLRPRLGFRLADQRIHGSDGLGAPG 587
Photi_PdnM      RNDYVANMNNTHWIVNPAQPLTGFAAVTGREGTEMGLRPRLGFRLADQRIQGSDGLGAPG 597
                *.*:* * *:: *:.***.***.** :*: :.. *       : **  : :   .*::*.: * *
                                                           (3)
PVDQ_PSEAE      LEEMVT----------ANHVFSADQVLPDLLRLCRDN----------------------- 558
Pcapi_PdnM      ASVDTVARAALDARAMSALLFKSQ----ALASACRADGRRQIVTVEPSGDPQGDQPGKTA 652
Pnori_PdnM      ATADSVARAALDARSMSALLFKSQ----AVASACKPDGGRQIVSVQPIEKDRN-----AT 638
Photi_PdnM      ANADTVARAALDARSMSALLFKSQ----AVANACRADGGRQIVTVEAFEKAQI-----PA 648
                     .          :  :*.::     :   *: :
```

```
                                 (3)
PVDQ_PSEAE      -QGEKSLARACAALAQWDRGANLDSGSGFVYFQRFMQR--FAELDGAWKEPFDAQRPLDT 615
Pcapi_PdnM      APRDVDIAPACAVLKKWDDKGGPFAQ-GAVLWNAWWAQIKAVPEDKRFAVPFDPQRPLDT 711
Pnori_PdnM      PARDVDIAEACRVLQRWDNTAGPFAQ-GAVLWDAWWAQIKSVPEDKRFAVPFDPQRPLDT 697
Photi_PdnM      QARDVDIAPACAVLQRWDNTGGPFAQ-GAVLWDTWWAQIKSVPEDKRFAVPLDPQHPLDT 707
                   : .:* ** .* :**  ..  :  * * :: :  :   .  *  :  *:* *:****

PVDQ_PSEAE      PQGIALDRPQVATQVRQALADAAAEVEKSGIPDGARWGDLQVSTRGQERIAIPGGDGHFG 675
Pcapi_PdnM      PATLKLDGKTLANGLGVAV----LRLREGGFTPDVETGSAMYATRNGVRIPWFGGCDDEG 767
Pnori_PdnM      PDTLNVDGKTLANWLGIAV----LRLREGGFTPDVETGSAMYAVRNGVRIPWFGGCDDEG 753
Photi_PdnM      PNTLNVDGKTLAKWLGVAV----LRLREGGFTPDVETGSAMYAMRNGVRIPWFGGCDDEG 763
                *  : :*   :*. :  *:      .:.:.*:  ... *.   : *.  **   ** .. *

PVDQ_PSEAE      VYNAIQS----VRKGD--HLEVVGGTSYIQLVTFPEEGP--KARGLLAFSQSSDPRSPHY 727
Pcapi_PdnM      YFTSGCNSDARIEDGALKIDAHAFSNSYMQIVTFGANGGPVDARTLMAGSQADDPASPHF 827
Pnori_PdnM      YFTSGCNSDARIENGALKIDAHAFSNSYLQVVTFGANGGPVDARTLMAGSQSDDPASAHF 813
Photi_PdnM      YFASGCNRDARIEDGALKIDAHAFSNSYMQVVTFGASGGPVDARTLMAGSQSDDPASPHF 823
                 : :  .    :..*        . ..**:*:***  .*   .** *:* **:.** * *:

PVDQ_PSEAE      RDQTELFSRQQWQTLPFSDRQIDADPQLQ--RLSIRE----------------------- 762
Pcapi_PdnM      ADGTLRYSKQAWVARPLDAVTSATSAKPDSGGAWLDSAQATPFVLSDVGASATLPARWQA 887
Pnori_PdnM      ADGTLRYSKQAWVARPLDTTRT------VDGALWLDSTQATPTLLSNMPKGKDIPARWGA 867
Photi_PdnM      ADGTLRYSKQAWVARPLDAVSL------QDGGQWLDSAQATPASLSDSAVAKTVAARWLT 877
                 * *  :*:* * : *:.                : .

PVDQ_PSEAE      ------------------------------------------------------------ 762
Pcapi_PdnM      LASQGVPAREASELLGTSEAALVATEQGSLIVPLRMGREDFPAMLANLHRLGGLEVMVRT 947
Pnori_PdnM      LSKQGMPAREASELLGTSEAELVAAEQGSGVVPLRVAPSDLRAMLARLHRLGRIEVMVRT 927
Photi_PdnM      LAAQGVPAREASELLGTSEADLVATEQGSVIVPLRTGPDNLPAMLANLHRLGRLEVMVRT 937

PVDQ_PSEAE      ------------------------------------------------------------ 762
Pcapi_PdnM      EQGIGEKIVRSSKPLFDANGHATSALGMTLDVSRWQSAYAVTRYYGPKREPYRQLQFFDA 1007
Pnori_PdnM      EHGIAEQVIRSPKPLFDASGKATGALDMTVDASRWASAYAVTRYYGPKREPYRQLQFFDT 987
Photi_PdnM      EQGIGERVIHSSKPLFDAAGKATNTLAMTVDASRWVSAYAVTRDYGPKREPYRQLQFFDA 997

PVDQ_PSEAE      ------------------------------------------------------------ 762
Pcapi_PdnM      QGRNIAKMTVLDSKPAAHVMDYDAFVASYRAGPQDIASIK-----GATPKPVAAQVPVTQ 1062
Pnori_PdnM      QGRNIAKITSVDE---QHVTDYDAFVAAFRAPGTQGHVVA----PPGRTAPATSPDQAAY 1040
Photi_PdnM      QGRNIAKITALDE---THMTDYDALVASFRASADNVPPPISRAGAAASAAPVGSQPAVAK 1054

PVDQ_PSEAE      ------------------------------------------------------------ 762
Pcapi_PdnM      GRCALASDDGRRARMALADALDAARTNGWPVRLGVNNGAASLAQDGRIEKIENIGGGWLS 1122
Pnori_PdnM      NLCQRMDQDGRRARLALADALDAAKSGGWPVRIDVSNDGASLAQAGRIEQVRNIGGGWLS 1100
Photi_PdnM      NQCRRVEHDGRRARLALADALDAAKANGWPVRIGVSNDGASLAQEGRIGNVHNIGGGWLR 1114

PVDQ_PSEAE      ------------------------------------------------------------ 762
Pcapi_PdnM      VQIRHFTLHVRASGIAAQCADISAQDEANRKSEDITWFDSFGAPVVSLALAPQATAQQRE 1182
Pnori_PdnM      VQIRDFTLHVRALGIAARCDDLGKN------DGQITLLDSRGVPSVSLSLTRQATEQQHQ 1154
Photi_PdnM      VQIREFTLHVRASGIAGRCDDRDGQ------YGQITLLDGRGAPSVSLMLAPQATAAQRQ 1168

PVDQ_PSEAE      ----------------------------------------------       762
Pcapi_PdnM      RWLAMWQKGDAPALAVSRRSP-------------------------       1203
Pnori_PdnM      RWLALWQENPTASGKQAISQNAPTR---------------------       1179
Photi_PdnM      RWLALWRANATSALPAPQPQNNHKDNIARVARHERRAGRNPRKVA       1213
```

Figure S30. Multiple sequence alignment of PdnM and PvdQ. The alignment was prepared using Clustal Omega (https://www.ebi.ac.uk/jdispatcher/msa/clustalo). Signal peptide sequences are colored gray. Red “C” indicates a disulfide bond-forming cysteine and those with the same numbers in parentheses are paired. Catalytic residues are indicated in bold blue. Substrate binding sites of PvdQ are highlighted in yellow.

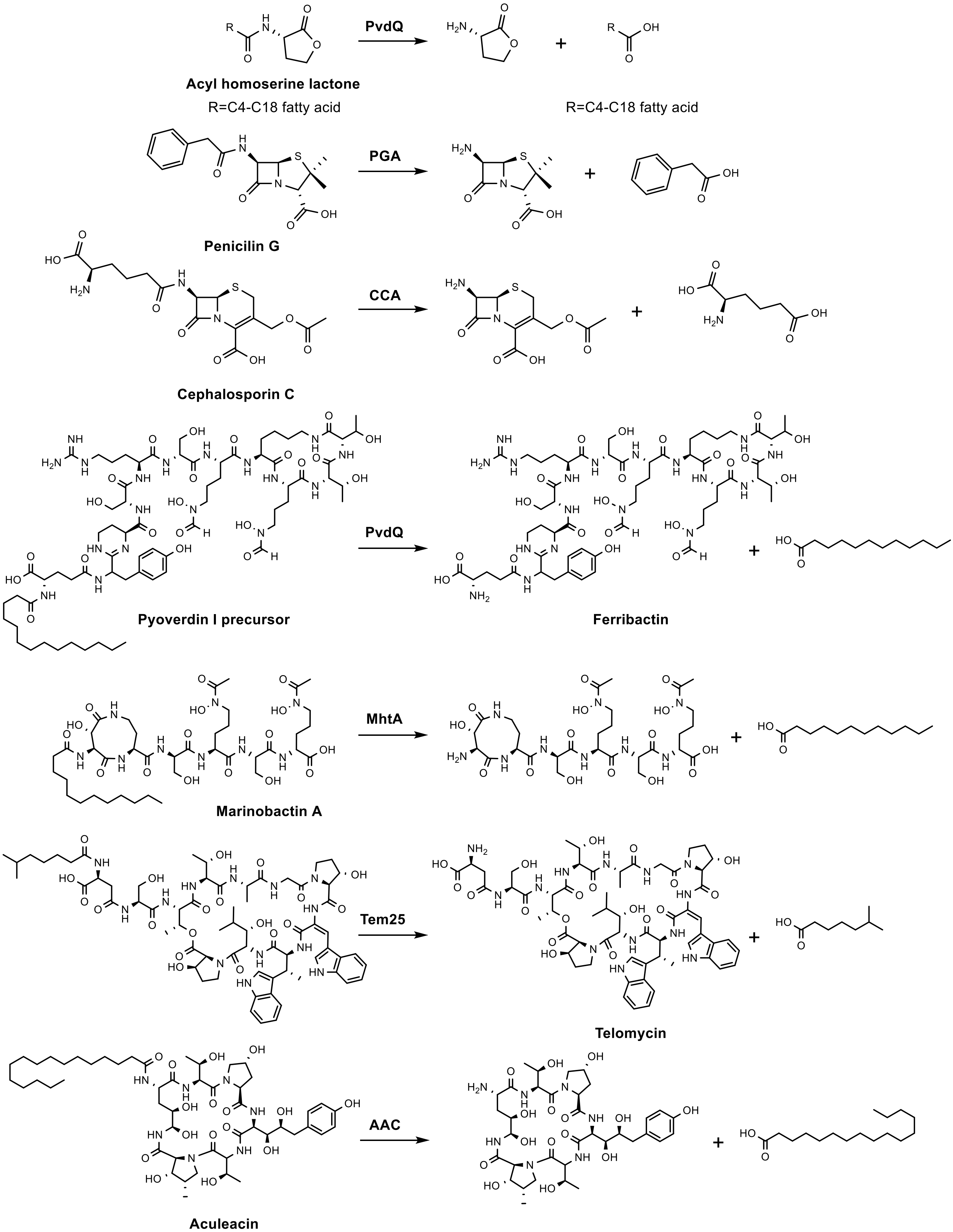

Figure S31. Function of fatty acid acylases found in bacteria. PGA, penicillin G acylase; CCA, cephalosporin C acylase; AAC, aculeacin acylase.

Preparation of maximum likelihood phylogenetic tree

The PdnM homologous proteins were obtained from NCBI server (https://www.ncbi.nlm.nih.gov/) using BLASTp suite with database of UniProtKB/Swiss-Prot and the protein data bank PDB (https://www.rcsb.org/). The amino acid sequences of these proteins (PdnM used only NTN hydrolase domain) were aligned using MAFFT 7[16] (https://mafft.cbrc.jp/alignment/server/) using the default setting expect for the scoring matrix for amino acid sequences; JTT100, Mafft-homologs; ON. An unrooted fast maximum-likelihood-based tree was generated using IQ-Tree[21] (https://www.hiv.lanl.gov/content/sequence/IQTREE/iqtree.html) with automatic model selection mode (ModelFinder[22]) resulting in the amino acid replacement model WAG+F+R4. Phylogenetic bootstrap analysis was performed by ultrafast approximate bootstrap.[23] The tree was displayed using MEGA7.[24]

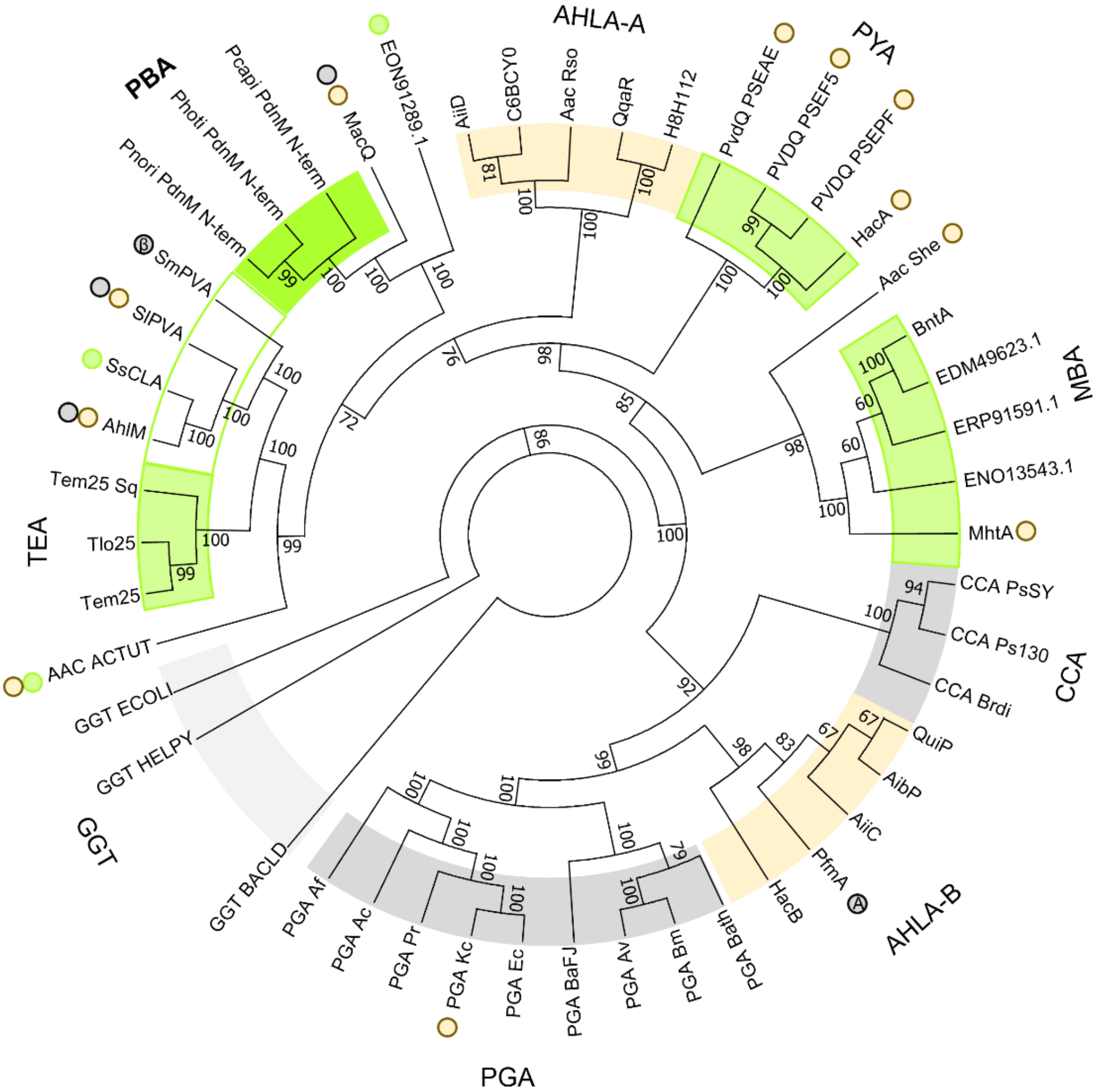

Figure S32. Maximum likelihood phylogenetic tree for PdnM and related acylase enzymes. Color codes indicate similar substrates. The numbers at the nodes indicate the ultrafast bootstrap score (1,000 replicates, shown value: %) for reliability of the different groups. GGT, γ-glutamyltranspeptidase; PGA, penicillin G acylase; AHLA-B, acyl homoserine lactone (AHL) acylase type B; CCA, cephalosporin C acylase; MBA, marinobactin acylase; PYA, pyoverdine I precursor acylase; AHLA-A, AHL acylase type A; PBA, pandorachelin B acylase; TEA, telomycin acylase. Small colored circles show reported acylase activities. "A" and "β" in gray circle indicate ampicillin and β-lactam, respectively.

Table S14. List of acylases used for phylogenetic tree.

Protein	Function	Source	Accession number
GGT_BACLD	γ-Glutamyltranspeptidase	*Bacillus licheniformis* ATCC 14580	4Y23
GGT_ECOLI	γ-Glutamyltranspeptidase	*Escherichia coli* K12	2E0W
GGT_HELPY	γ-Glutamyltranspeptidase	*Helicobacter pylori* ATCC 700392 / 26695	2NQO
PGA_Ac	Penicillin G acylase	*Achromobacter* sp. CCM 4824	AAY25991.1
PGA_Af	Penicillin G acylase	*Alcaligenes faecalis* ATCC 19018	AAB71221.1
PGA_Av	Penicillin G acylase	*Arthrobacter viscosus* ATCC 15294	P31956.1
PGA_Bath	Penicillin G acylase	*Bacillus thermotolerans* MTCC 8252	6NVY
PGA_BaFJ	Penicillin G acylase	*Bacillus* sp. FJAT-27231	8BRR
PGA_Bm	Penicillin G acylase	*Bacillus megaterium* ATCC 14945	Q60136.1
PGA_Ec	Penicillin G acylase	*Escherichia coli* ATCC11105	P06875.2
PGA_Kc	Penicillin G/AHL acylase	*Kluyvera citrophila* DSM 2660	P07941.1
PGA_Pr	Penicillin G acylase	*Providencia rettgeri*	AAP86197.1
AiiC	AHL acylase B	*Nostoc* sp. PCC 7120	BAB75623.1
AibP	AHL acylase B	*Brucella melitensis* bv. 1 str.16M	AAL53453.1
HacB	AHL acylase B	*Pseudomonas syringae* pv. *syringae* B728a	AAY39885.1
PfmA	AHL/ampicillin acylase B	*Pseudoalteromonas flavipulchra* JG1	ASS36259.1
QuiP	AHL acylase B	*Pseudomonas aeruginosa* PAO1	AAG04421.1
CCA_PsSY	Cephalosporin acylase	*Pseudomonas* sp. SY-77	3S8R
CCA_Ps130	Cephalosporin acylase	*Pseudomonas* sp. 130	4E57
CCA_Brdi	Cephalosporin acylase	*Brevundimonas diminuta*	1KEH
BntA	Marinobactin acylase	*Marinobacter sp.* DS40M6	AJD87516.1
MhtA	Marinobactin/AHL acylase	*Marinobacter nanhaiticus* D15-8W	ENO13542.1
ENO13543.1	Putative marinobactin acylase	*Marinobacter nanhaiticus* D15-8W	ENO13543.1
ERP91591.1	Putative marinobactin acylase	*Marinobacter sp.* ES-1	ERP91591.1
EDM49623.1	Putative marinobactin acylase	*Marinobacter algicola* DG893	EDM49623.1
Aac_She	AHL acylase	*Shewanella sp.* MIB015	BAF94155.1
PvdQ_PSEAE	Pyoverdin I precursor/AHL acylase	*Pseudomonas aerugnosa* PAO1	4WKS
PVDQ_PSEF5	Pyoverdin I precursor/AHL acylase	*Pseudomonas protegens* Pf5 (multi strains)	Q4KCM5.1
PVDQ_PSEPF	Pyoverdin I precursor/AHL acylase	*Pseudomonas fluorescens* Pf0-1	Q3KD51.1
HacA	Pyoverdin I precursor/AHL acylase	*Pseudomonas syringae* pv. *syringae* B728a	AAY37014.1
Aac_Rso	AHL acylase A	*Ralstonia pseudosolanacearum* GMI1000	WP_011002462.1
AiiD	AHL acylase A	*Ralstonia sp.* XJ12B	AAO41113.1
C6BCY0	AHL acylase A	*Ralstonia pickettii* strain 12D	C6BCY0
H8H112	AHL acylase A	*Deinococcus gobiensis* DSM 21396	H8H112
QqaR	AHL acylase A	*Deinococcus radiodurans* R1	WP_010889514.1
EON91289.1	Putative marinobactin acylase	*Marinobacter lipolyticus* SM19	EON91289.1
MacQ	AHL/Penicilin G acylase	*Acidovorax* sp. MR-S7	5C9I
Pcapi_PdnM_N-term	Pandorachelin B acylase	*Pandraea capi*	WP_242559656.1
Photi_PdnM_N-term	Pandorachelin B acylase	*Pandoraea horticolens* LMG31112	WP_150621410.1
Pnori_PdnM_N-term	Pandorachelin B acylase	*Pandoraea norimbergensis*	WP_058378715.1
AhlM	AHL/penicilin G acylase	*Streptomyces sp.* M664	AAT68473.1
SlPVA	Putative AHL/penicillin G acylase	*Streptomyces lavendulae* subsp. *lavendulae*	AAU09670.1
SmPVA	β-lactam acylase	*Streptomyces mobaraensis*	BAF51977.1
SsCLA	FR901379 acylase	*Streptomyces sp.* FERM BP-5809	BAD07025.1
Tem25	Telomycin acylase	*Streptomyces canus* ATCC 12647	AKQ13300.1

Tem25_Sq	Putative telomycin acylase	*Streptomyces qaidamensis* S10	AMW13854.1
Tlo25	Putative telomycin acylase	Streptomyces canus ATCC 12647	ALV86871.1
AAC_ACTUT	Aculeacin-A/AHL acylase	*Actinoplanes utahensis*	P29958.1

Generation of *pdnM* mutant of *P. norimbergensis* DSM11628

The *pdnM* gene knockout plasmid pGL42a_T251A-Δ*pdnM* was prepared as described above using the primer pairs PdnM-KO1-XhoI/PdnM-KO1-PacI and PdnM-KO2-KpnI/PdnM-KO2-NheI. The *P. norimbergensis* DSM11628 cultures and transformation were as described for the *pdnI* mutant. The mutants were checked by colony PCR using the primer pairs PdnM-fw/PdnM-rv.

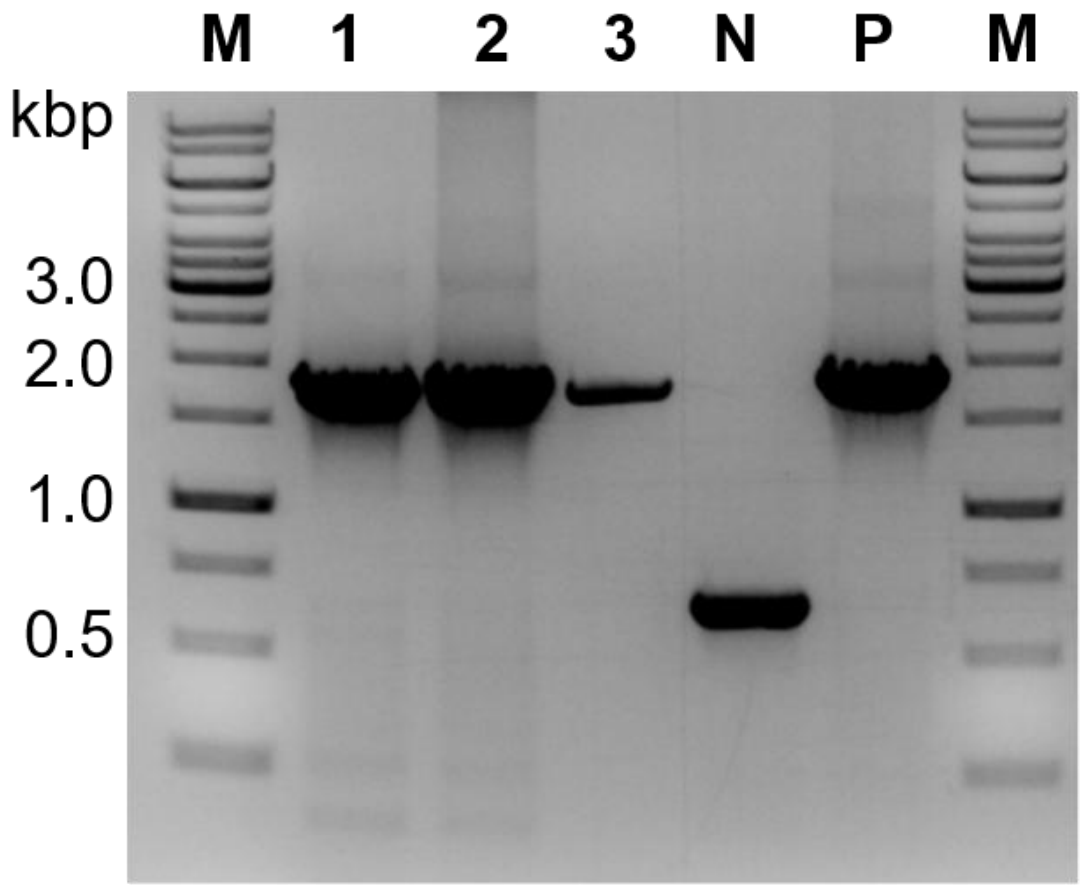

Figure S33. PCR confirmation of *P. norimbergensis* Δ*pdnM* mutants. Template DNA: genomic DNA of *P. norimbergensis* Δ*pdnM* (**1–3**), genomic DNA of *P. norimbergensis* wild type (**N**), pGL42a_T251A-Δ*pdnM* (**P**). **M**: DNA size marker. The estimated size of amplicons obtained by using the primer pair PdnM-fw/PdnM-rv, lane **1–3** and **P**; 1,720 bp, **N**; 623 bp.

Extraction of *P. norimbergensis* Δ*pdnM*

P. norimbergensis Δ*pdnM* was cultivated overnight at 30 °C in MM9 medium with trace elements supplemented with tetracycline (75 µg · mL^{-1}) with orbital shaking at 120 rpm. 10 mL of MM9 medium with trace elements and tetracycline (75 µg · mL^{-1}) was inoculated with *P. norimbergensis* Δ*pdnM* (starting OD_{600} of 0.05) in 50 mL baffled Erlenmeyer flasks and incubated at 30 °C with orbital shaken at 120 rpm for four days. For extraction Amberlite® XAD16 resin was used as described above. The cultivation was performed in biological triplicates with technical duplicates.

Heterologous production and purification of PdnM (His$_6$-α-chain and β-chain-His$_6$)

The gene *pdnM* was amplified by PCR (template: genomic DNA of *P. norimbergensis*) with the primers PdnM-NheI-2His-fw and PdnM-BamHI-rv using Phusion Polymerase. The amplicon was gel-purified as before and inserted into pCR2.1 T/A cloning vector. The obtained plasmid was restricted with *Bam*HI and *Nhe*I and the gene fragment was ligated into *Bam*HI- and *Nhe*I-restricted pET28a, generating pET28-*pdnM*12. This plasmid was restricted with *Nco*I and *Bam*HI and the obtained His$_6$-*pdnM* gene fragment was ligated into the *Nco*I- and *Bam*HI-restricted pET26b, generating pET26-*pdnM*12. This plasmid was transformed into electrocompetent *E. coli* Rosetta2 (DE3). The resulting strain was grown at 37 °C in 200 mL LB medium containing kanamycin (50 µg · mL^{-1}) and chloramphenicol (12.5 µg · mL^{-1}) in a baffled Erlenmeyer flask. Gene expression was induced with IPTG (0.5 mM) once an OD_{600} of 1.2 was reached, followed by incubation at 16 °C for 18 h. Cells were harvested by centrifugation (8,000 × *g*) at 4 °C for 10 min and resuspended in 40 mL of 10 mM Tris-HCl (pH 8.0). After the cells were centrifuged at 4,000 × *g* for 10 min at 10 °C, the residue was resuspended in 40 mL of 50 mM Tris-HCl (pH 8.0), 500 mM sucrose. The swelled cells were centrifuged at 5,000 × *g* for 10 min at 10 °C. The precipitates were resuspended in 25 mL of 1 mM Tris-HCl (pH 8.0) and kept at 0 °C for 10 min. After the suspension was centrifuged at 5,000 × *g* at 4 °C for 10 min, the supernatant was further centrifuged at 12,000 × *g* at 4 °C for 30 min.

The periplasmic extract was adjusted to 50 mM Tris-HCl (pH 8.0) and then filtered by Chromafil (0.45 µm, Macherey-Nagel). The filtrate was subjected to a Ni-NTA agarose column (1.5 mL, Qiagen). The resin was washed with 100 mL of a buffer solution (50 mM Tris HCl (pH 8.0), 300 mM NaCl, 5% glycerol, and 10 mM imidazole). The PdnM fragments were eluted with 10 mL of a buffer solution (50 mM Tris HCl (pH 8.0), 300 mM NaCl, 5% glycerol, and 250 mM imidazole). The eluents containing PdnM fragments were concentrated with Amicon Ultra-3K (Millipore) to yield ~4.9 mg as a mixture of protein fragments in 50 mM HEPES buffer (pH 7.5).

In-gel tryptic digestion of PdnM

In-gel tryptic digestion of the SDS-PAGE gel cube (selected band) was performed as reported by Shevchenko and co-workers.[25]

a) MALDI-TOFMS

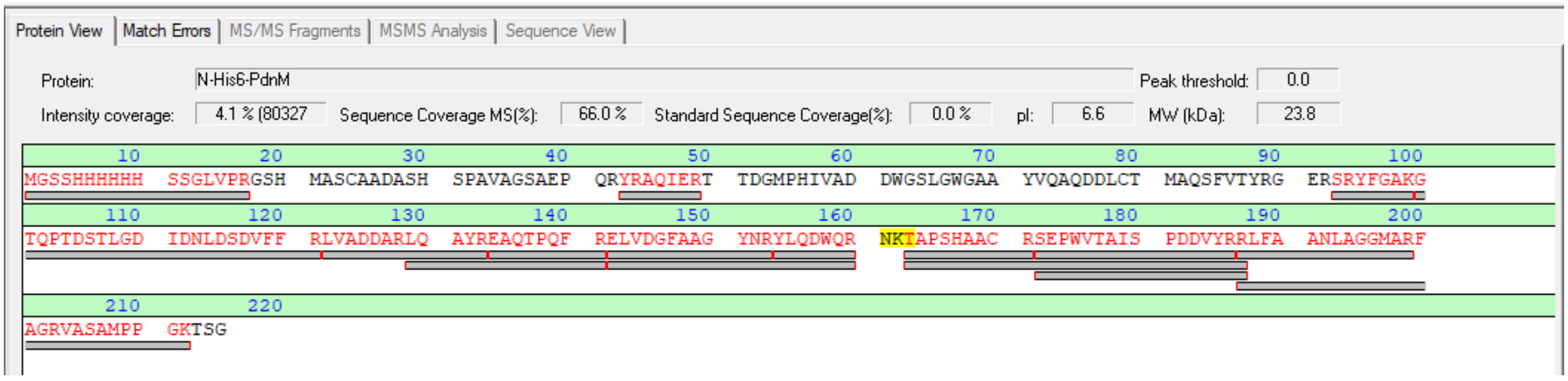

b) HPLC-MS/MS

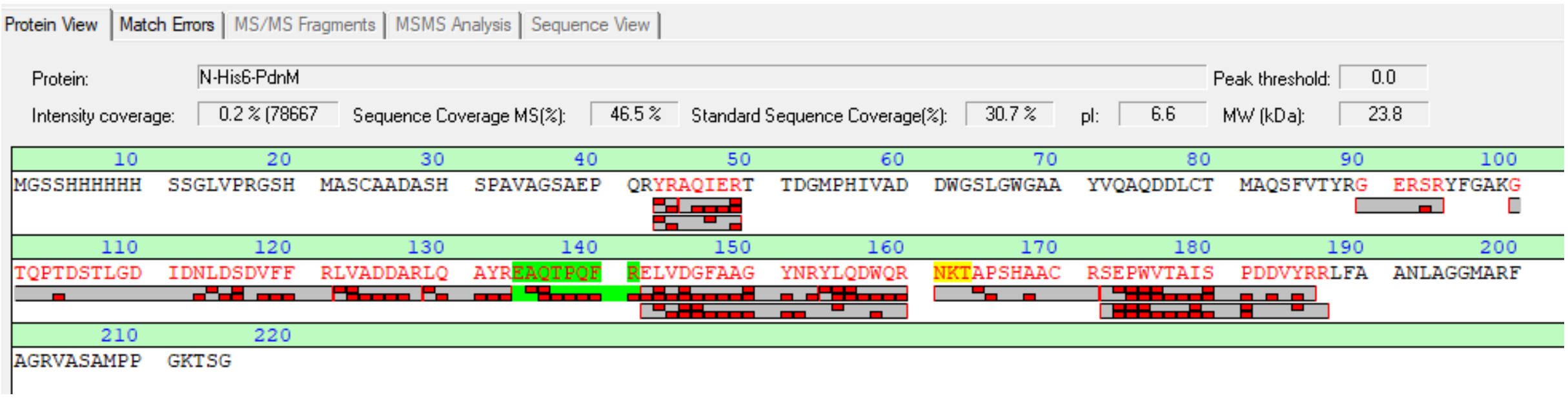

Figure S34. Fingerprint analysis of peptide fragments of tryptic-digested His_6-PdnM α-chain. a) Results obtained by BioTools using MALDI-TOFMS data. b) Results obtained by BioTools using HPLC-MS/MS data.

a) MALDI-TOFMS

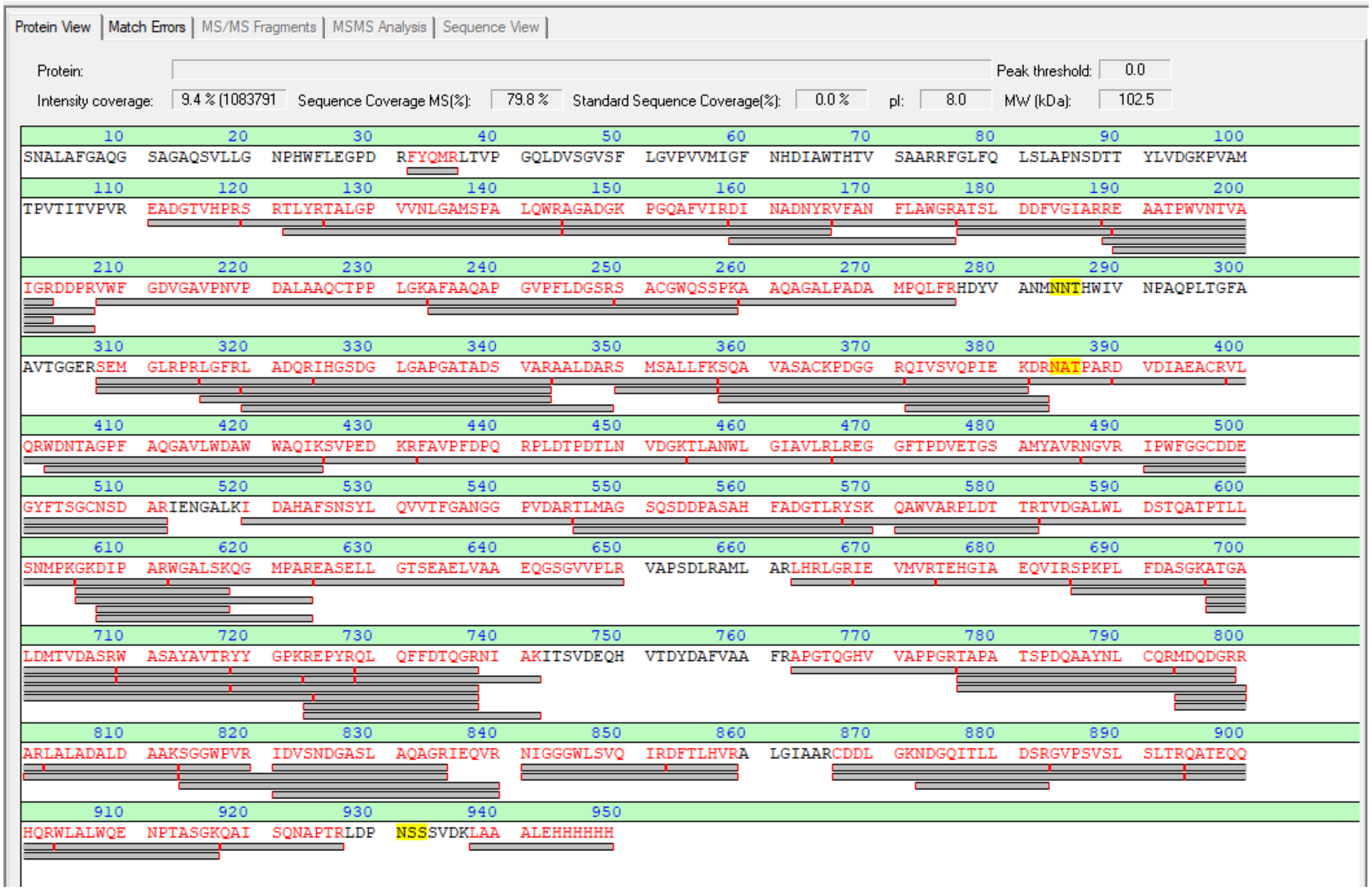

b) HPLC-MS

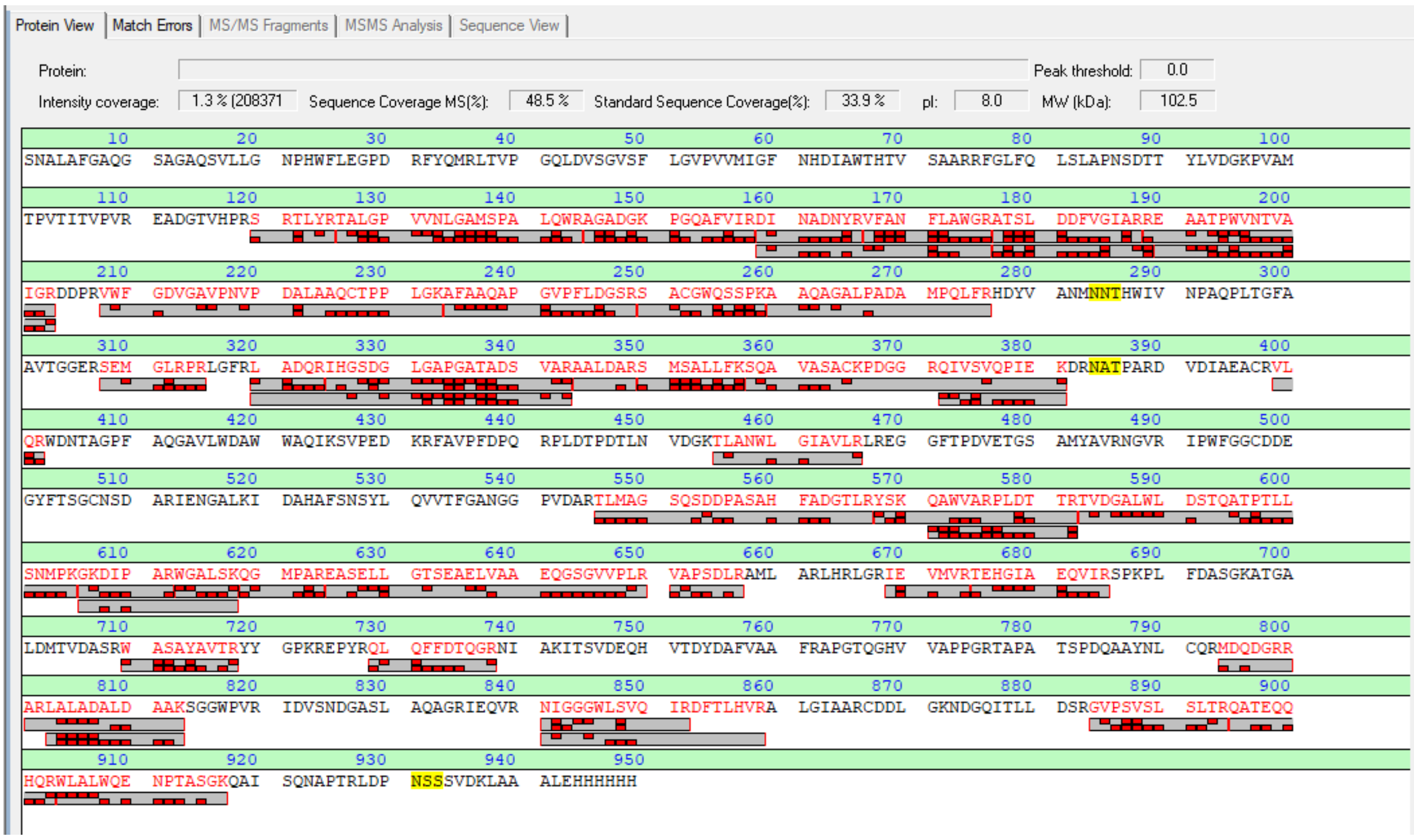

Figure S35. Fingerprint analysis of peptide fragments of tryptic-digested PdnM-His$_6$ β-chain. a) Results obtained by BioTools using MALDI-TOFMS data. b) Results obtained by BioTools using HPLC-MS/MS data.

SUPPORTING INFORMATION

a) MALDI-TOFMS

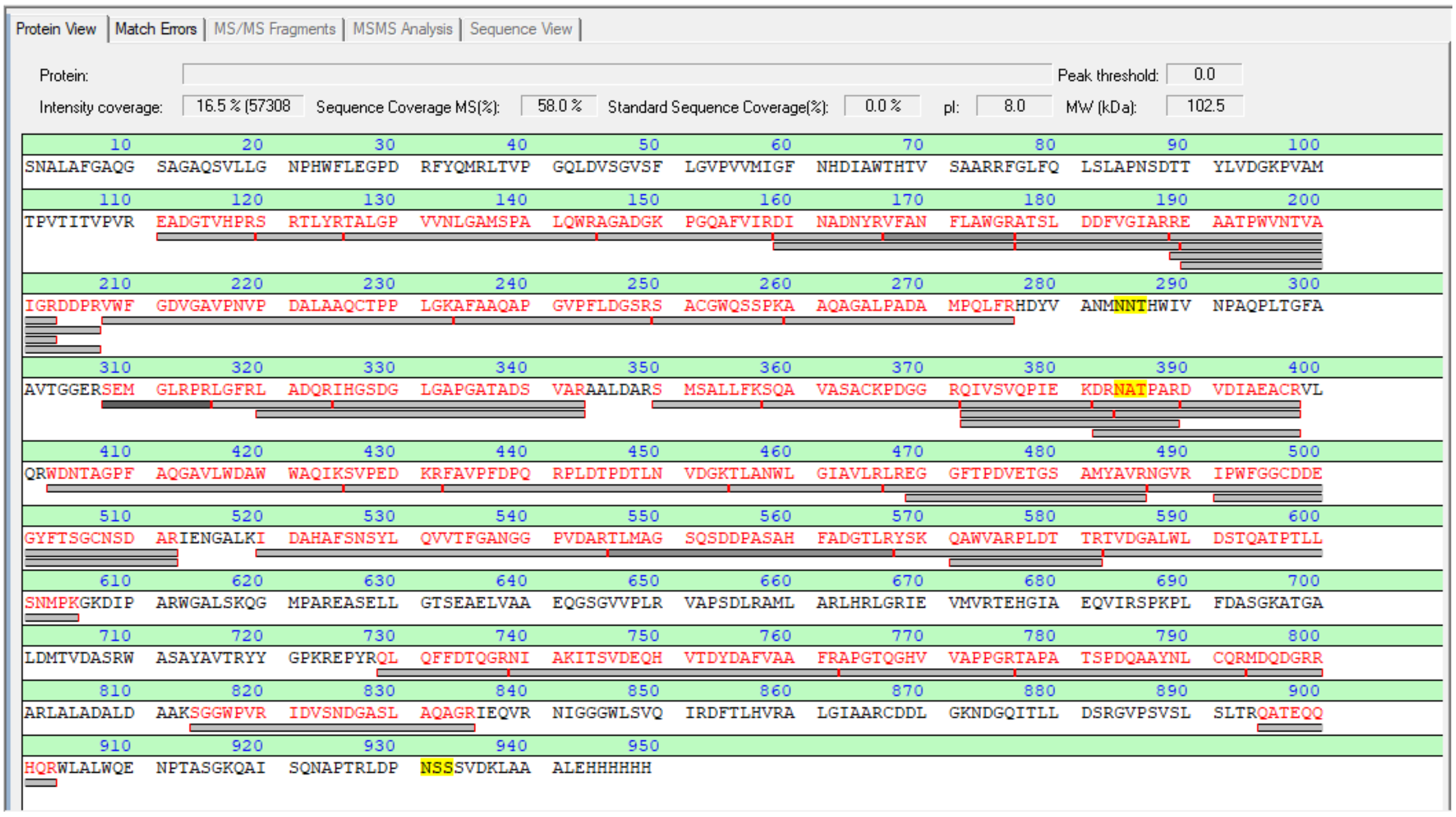

b) HPLC-MS/MS

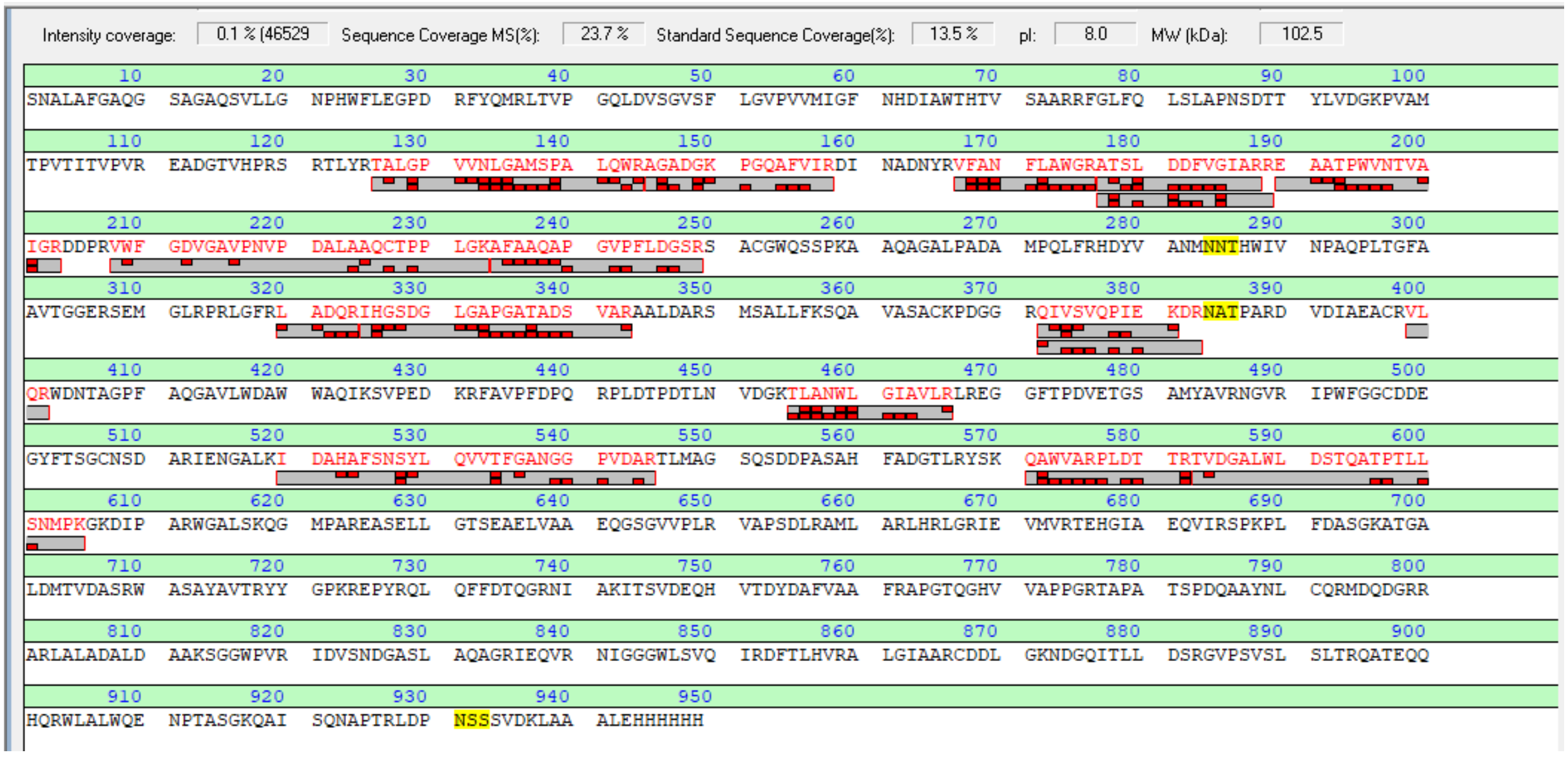

Figure S36. Fingerprint analysis of peptide fragments of tryptic-digested PdnM-His$_6$ β-chain (degraded, asterisk in Figure 4f). a) Results obtained by BioTools using MALDI-TOFMS data. b) Results obtained by BioTools using HPLC-MS/MS data.

Acylase activity assay of PdnM

A 50 µL reaction mixture consisted of 100 nM PdnM or heat-inactivated PdnM and pandorachelin B (0.1 mM) in buffer at varying pHs: 50 mM HEPES at pH 8.0, 7.5, 7.0; 50 mM MES at pH 6.0 and 5.0. After incubation at 30 °C for 30 min, the reaction was stopped with 1 µL of formic acid and cooled at −20 °C for 10 min. The supernatant obtained by centrifugation (14,000 × *g*) at 4 °C for 10 min was used for LC-MS analysis.

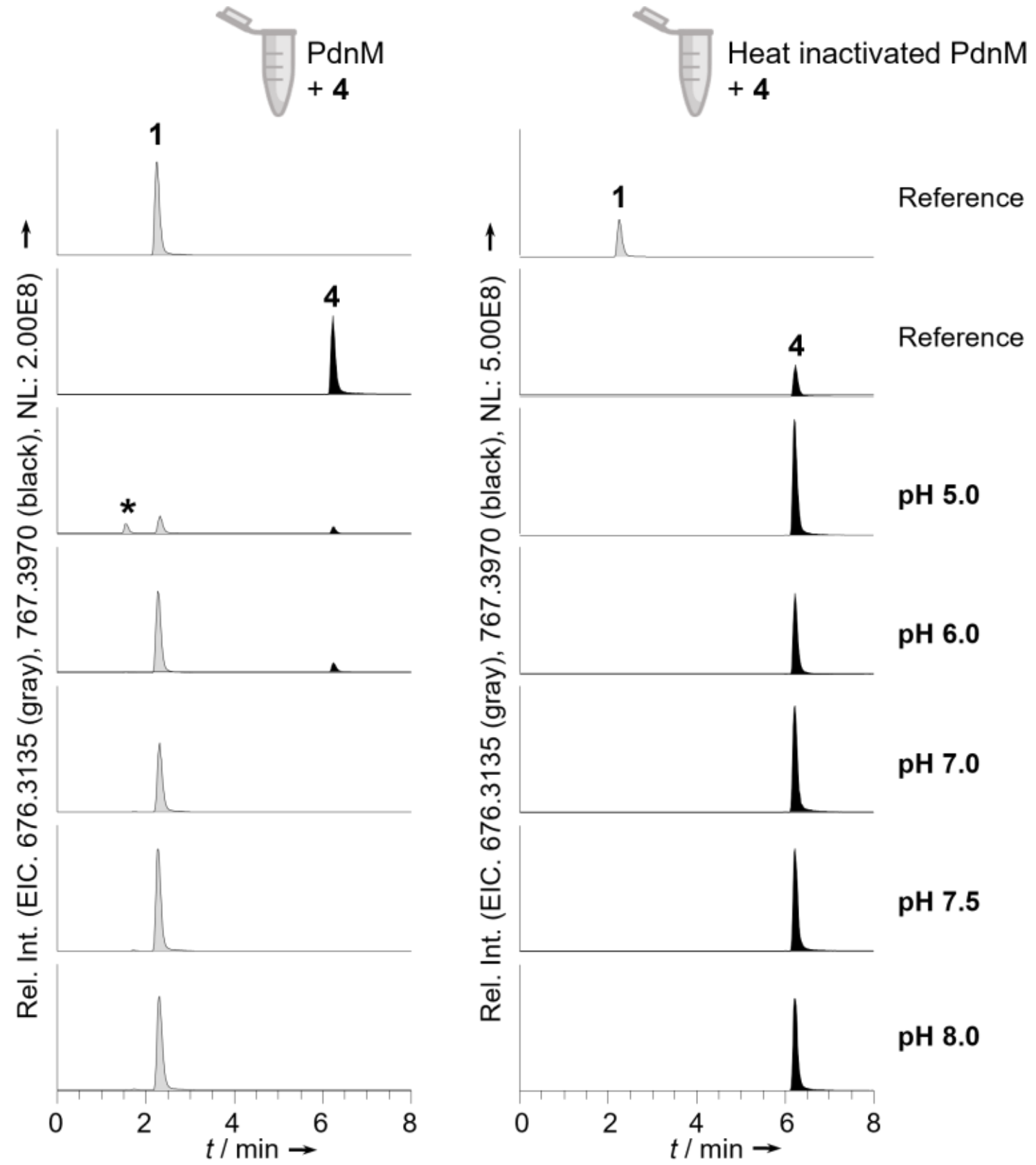

Figure S37. Acylase activity of PdnM demonstrating the conversion of pandorachelin B (**4**) (EIC 767.3970 $[M+2H]^{2+}$, black) to pandorachelin A (**1**) (EIC 676.3135 $[M+2H]^{2+}$, grey) at varying pH values. The left panel shows the reference and active enzyme reactions (* exhibits *m/z* of pandorachelin A lactone (**6**)), whereas the right panel presents the heat-inactivated PdnM control reaction.

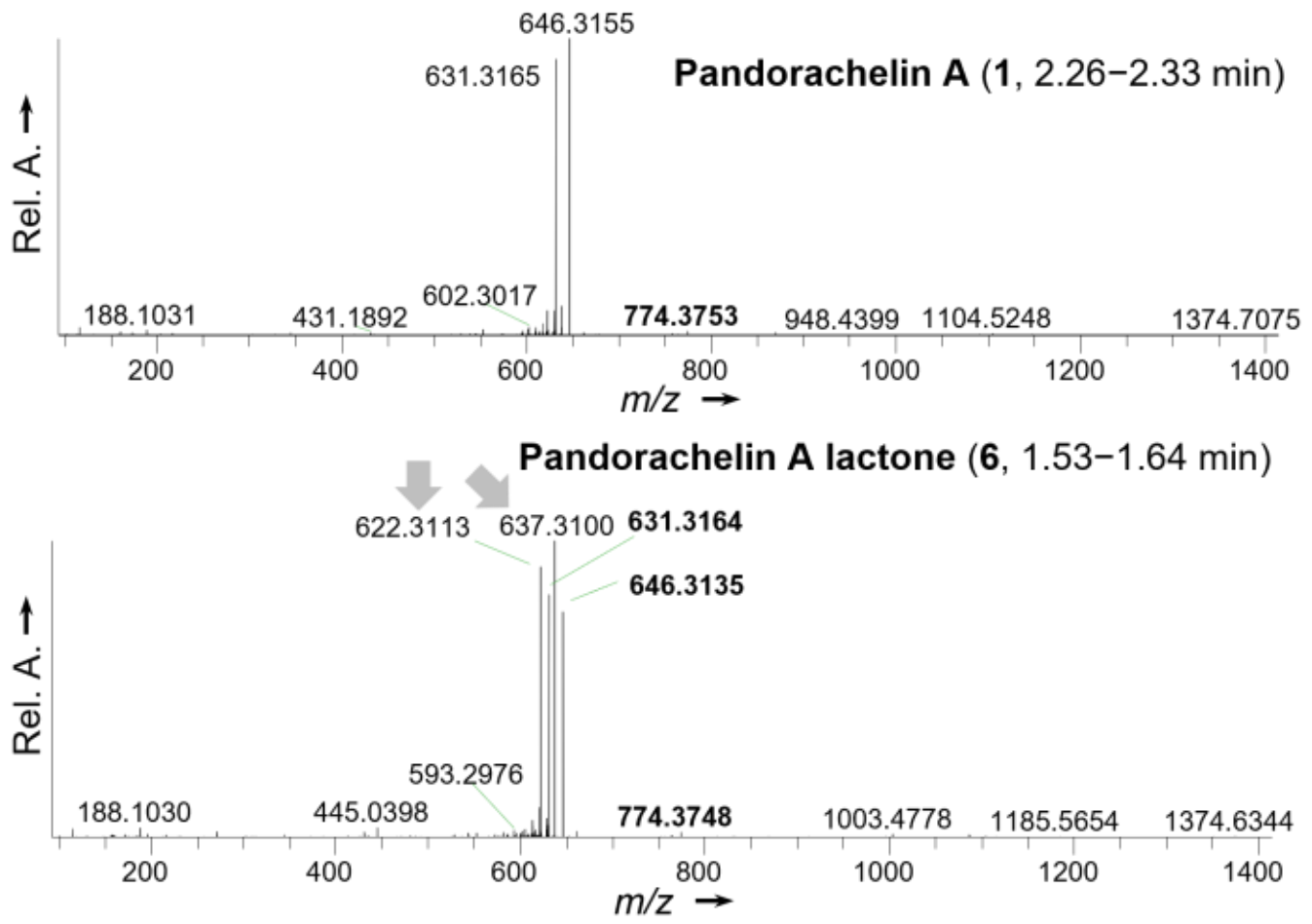

Figure S38. MS/MS (ESI$^+$, *m/z* 676.3135 $[M+2H]^{2+}$ hcd20) fragmentation comparison of pandorachelin A (**1**) and pandorachelin A lactone (**6**). Bold ions are fragment ions that are common to **1** and **6**. Gray arrows indicate assigned specific fragment ions observed in **6**.

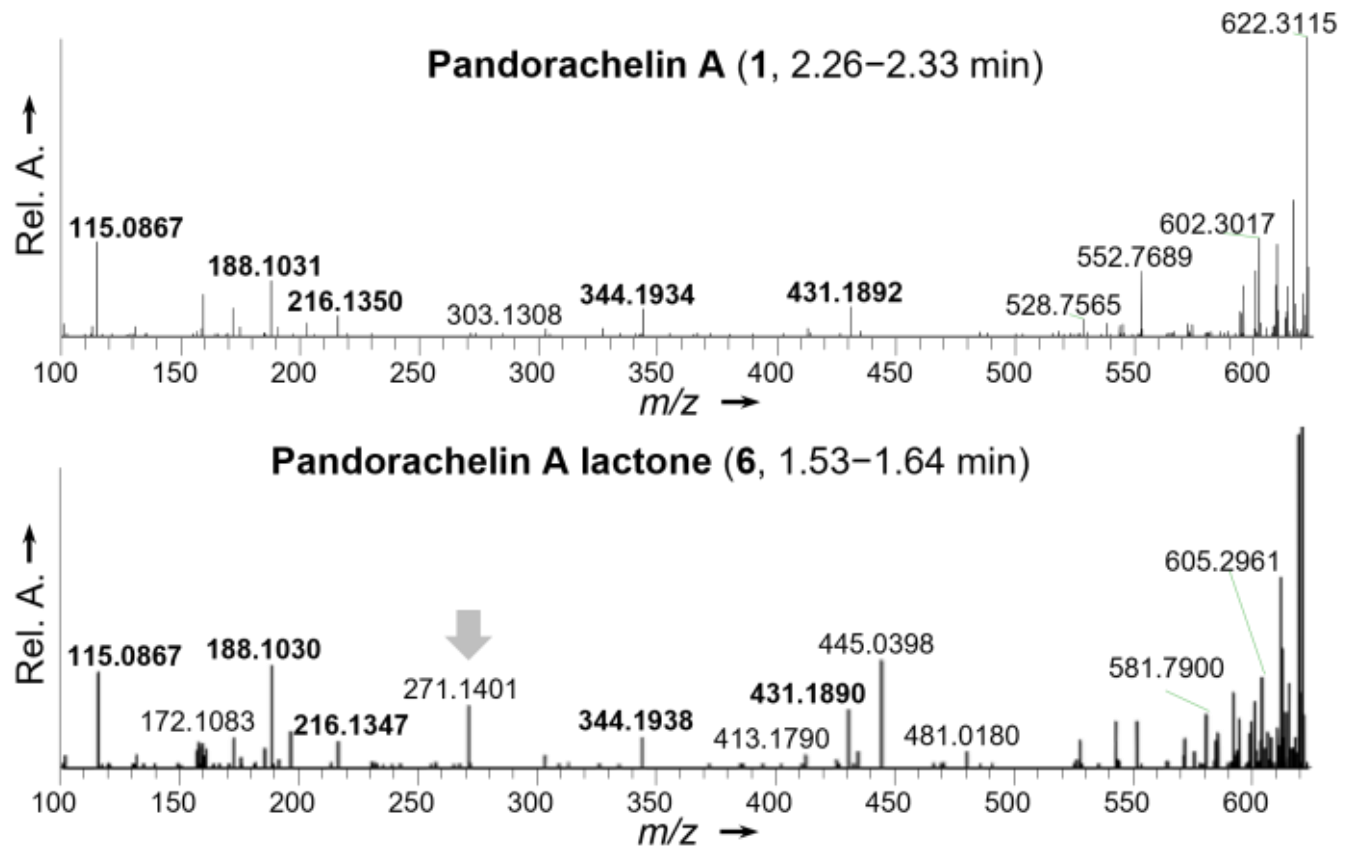

Figure S39. MS/MS (ESI$^+$, *m/z* 676.3135 $[M+2H]^{2+}$ hcd20, *m/z* 100–650) fragmentation comparison of pandorachelin A (**1**) and pandorachelin A lactone (**6**). Bold ions are fragment ions that are common to **1** and **6**. Gray arrow indicates an assigned specific fragment ion observed in **6**.

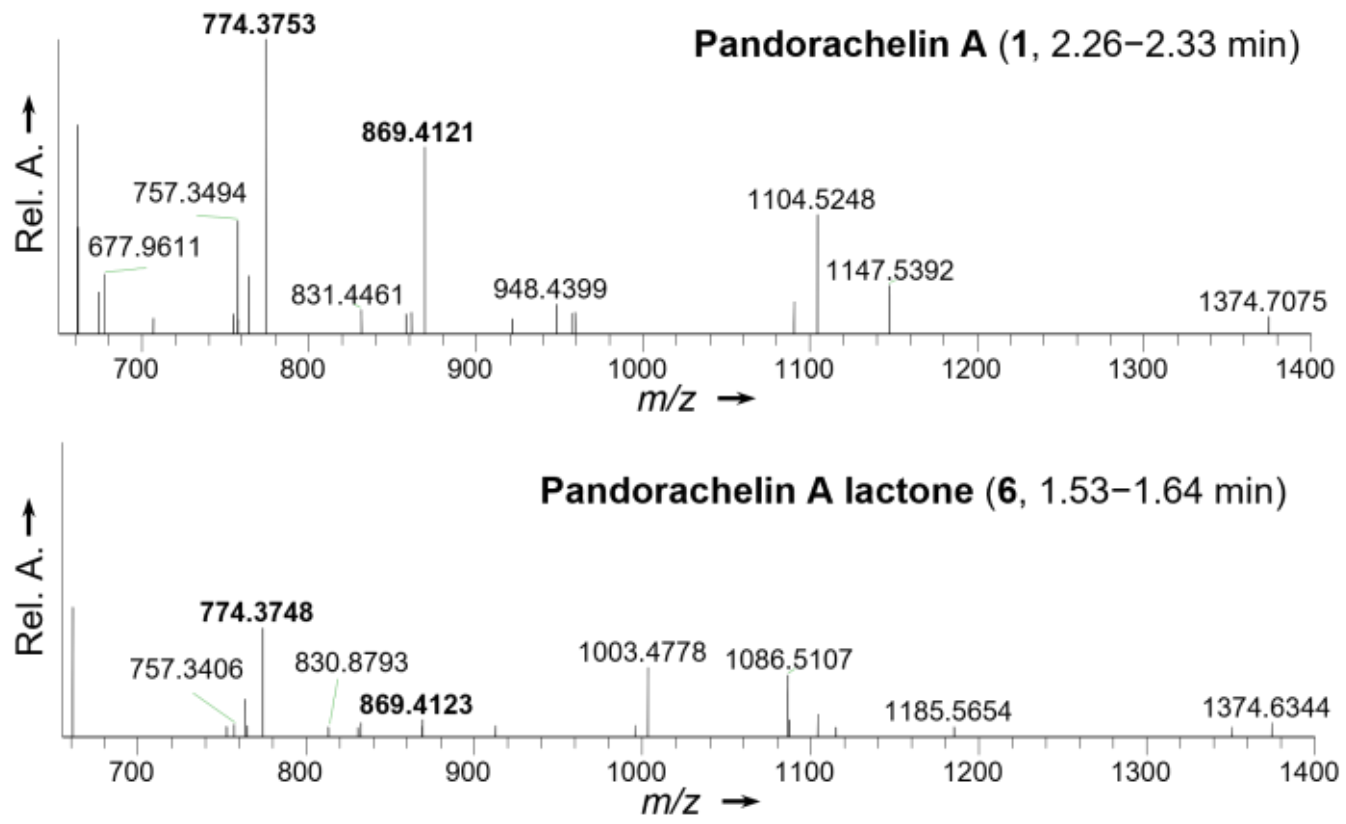

Figure S40. MS/MS (ESI$^+$, *m/z* 676.3135 $[M+2H]^{2+}$ hcd20, *m/z* 550–1400) fragmentation comparison of pandorachelin A (**1**) and pandorachelin A lactone (**6**). Bold ions are fragment ions that are common **1** and **6**.

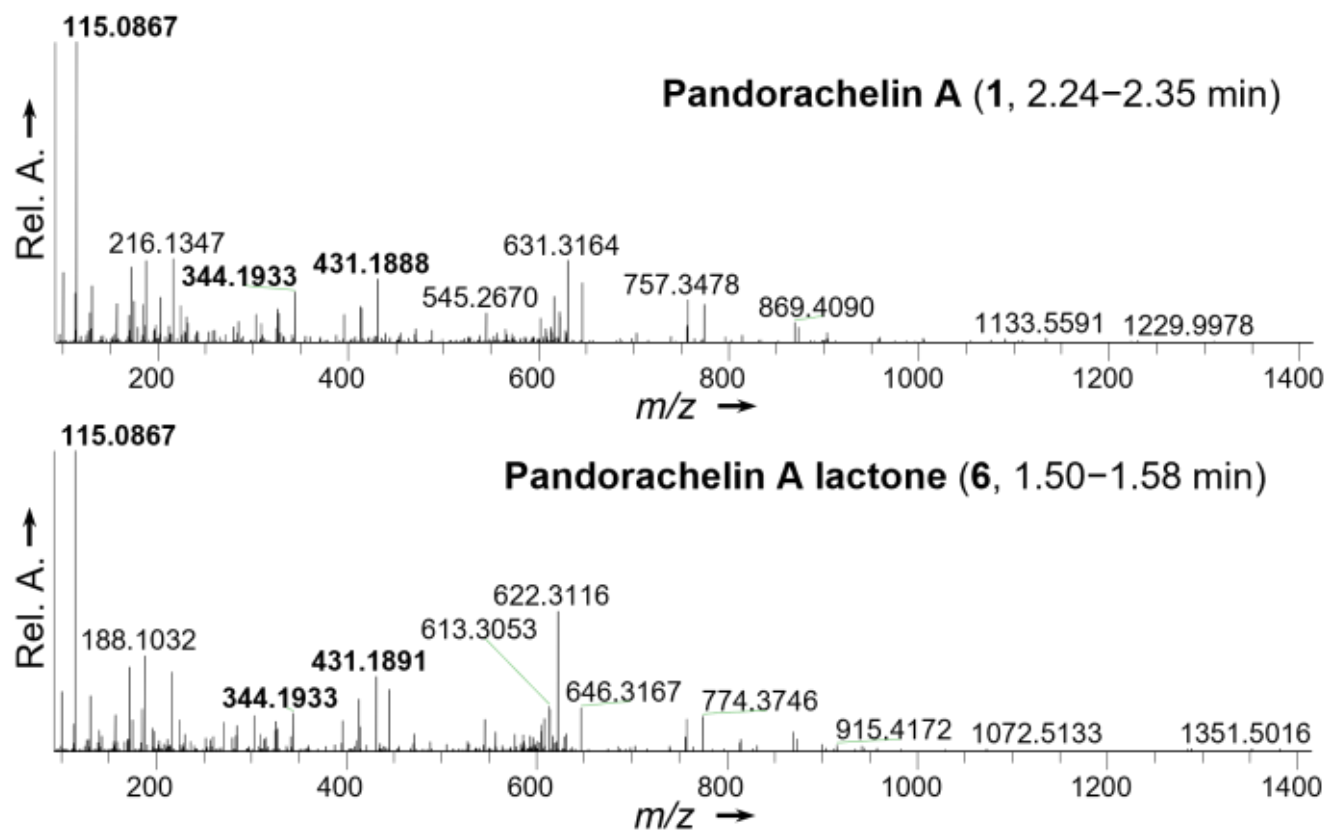

Figure S41. MS/MS (ESI$^+$, *m/z* 676.3135 $[M+2H]^{2+}$ hcd30) fragmentation comparison of pandorachelin A (**1**) and pandorachelin A lactone (**6**). Bold ions show the same fragment ions between **1** and **6**.

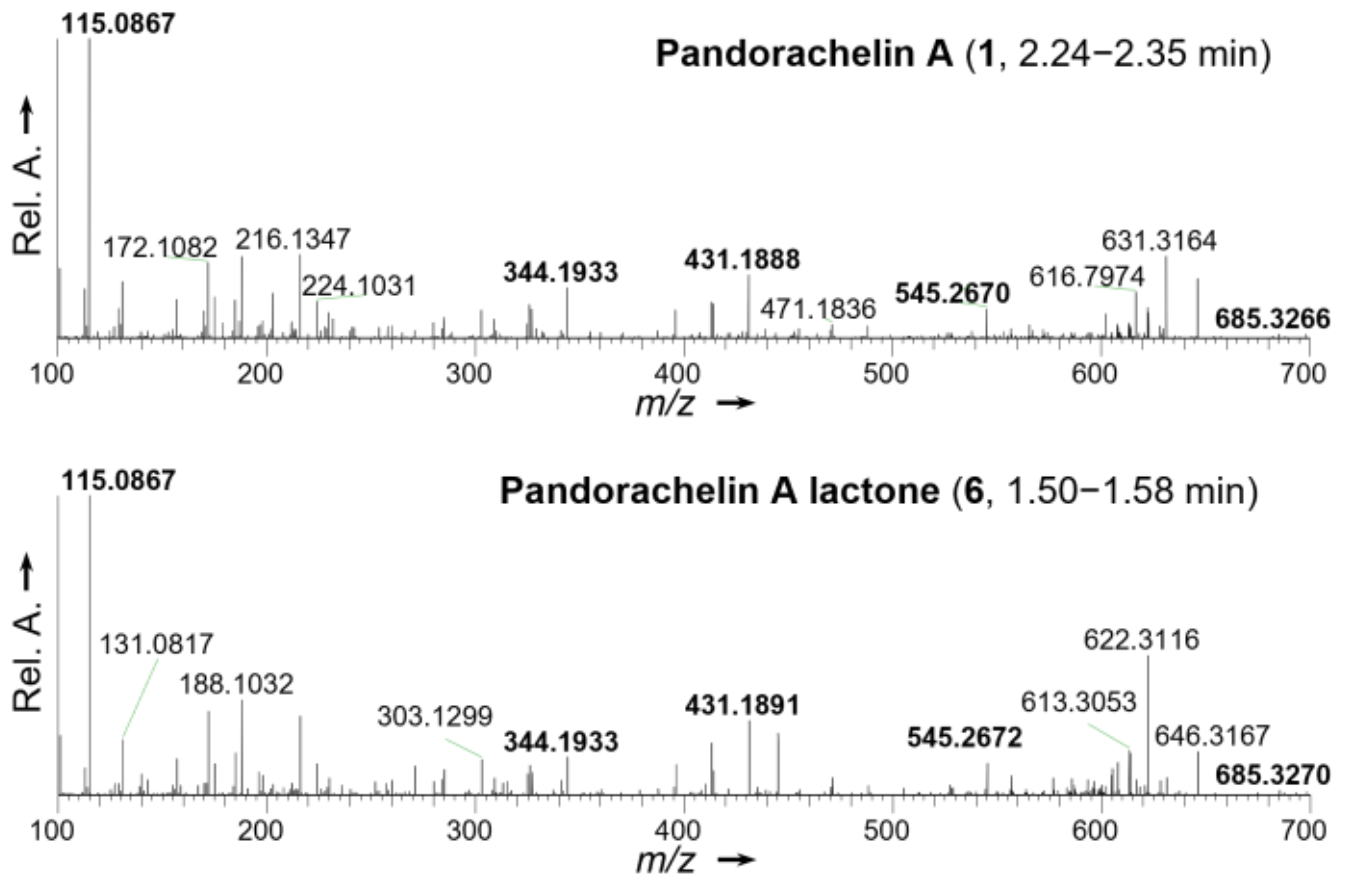

Figure S42. MS/MS (ESI$^+$, *m/z* 676.3135 $[M+2H]^{2+}$ hcd30, *m/z* 100–700) fragmentation comparison of pandorachelin A (**1**) and pandorachelin A lactone (**6**). Bold ions show the same fragment ions between **1** and **6**.

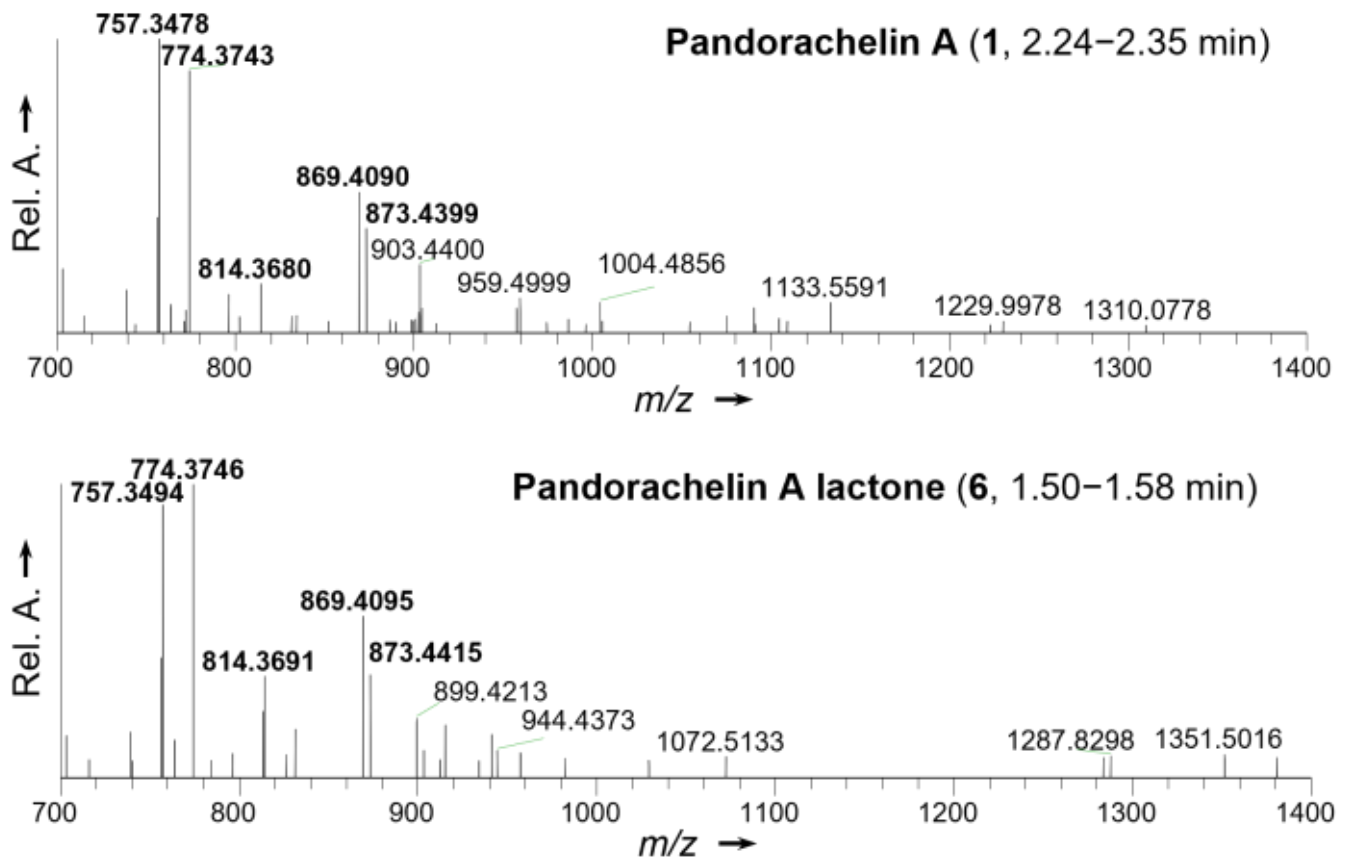

Figure S43. MS/MS (ESI$^+$, *m/z* 676.3135 $[M+2H]^{2+}$ hcd30, *m/z* 700–1400) fragmentation comparison of pandorachelin A (**1**) and pandorachelin A lactone (**6**). Bold ions show the same fragment ions between **1** and **6**.

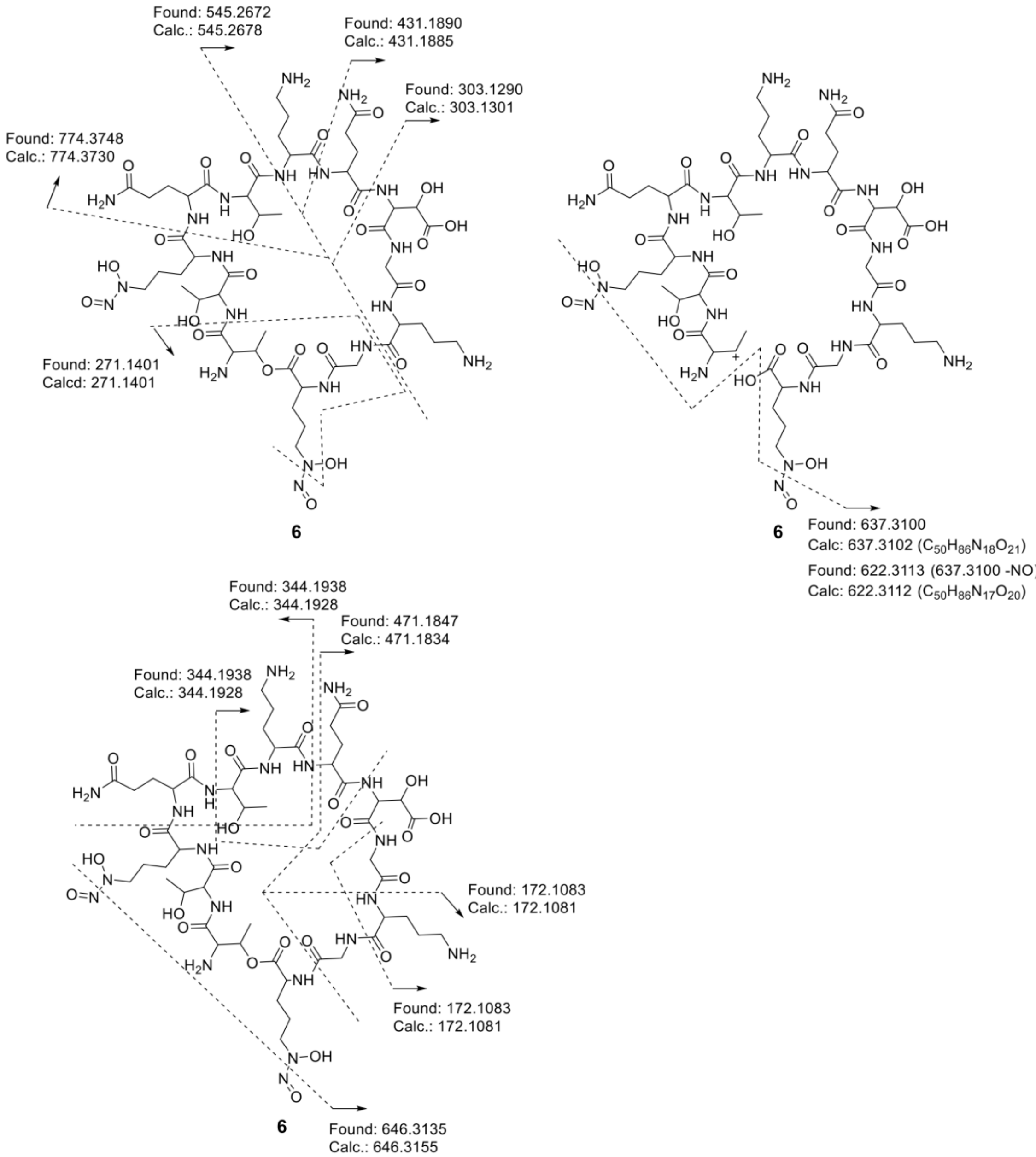

Figure S44. MS/MS fragmentation pattern of linearized pandorachelin A lactone (**6**).

Swarming assay

P. norimbergensis wild type, *P. norimbergensis* Δ*pdnM, P. norimbergensis* Δ*pdnI*, *P. norimbergensis pdnM^{hort}*, *P. norimbergensis pdnM-tet-N*, and *P. horticolens* were grown overnight at 30 °C in MM9 medium with trace elements with orbital shaking at 120 rpm. The mutant cultures were supplemented with tetracycline (75 µg · mL^{-1}). Fresh overnight cultures were adjusted to an OD_{600} of 0.07 and 10 µL of each culture was spotted onto minimal medium soft agar and cultivated at 30 °C for 3 days. For the pandorachelin B (**4**) concentration-dependent swarming assay with *P. norimbergensis* Δ*pdnI* inoculation, 0.5, 0.1, 0.04, 0.02, 0.01, 0.005, and 0.001 mM of pandorachelin B (**4**)-containing soft agar plates were prepared. The assay was performed in technical triplicates.

Extraction of the soft agar

Agar was crushed with a spatula and placed in Erlenmeyer flasks with a stirring bar and MeOH was added. Flasks were shaken for 2 h. The suspensions were filtered through filter paper and the solvent was removed under reduced pressure. The residue was resuspended in 1.2 mL MeOH and filtered again through a CHROMAFIL Xtra PTFE filter (0.20 µm), and dried using SpeedVac vacuum concentrator. The samples were dissolved in H_2O for HPLC-HRMS analysis.

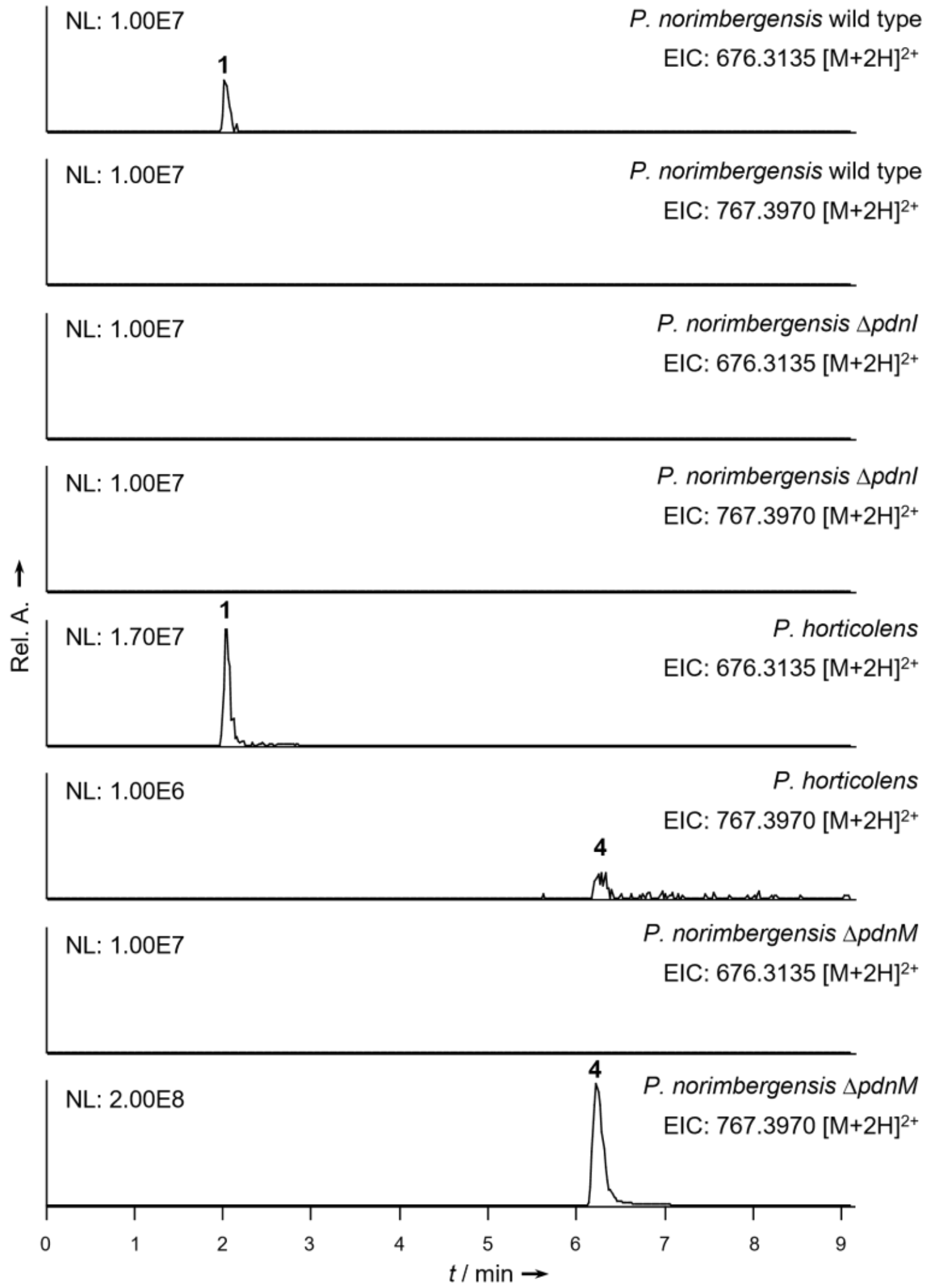

Figure S45. HPLC-HRMS (ESI+) (EIC, extracted ion chromatogram) of pandorachelin A (**1**) (*m/z* 676.3135 $[M+2H]^{2+}$) and pandorachelin B (**4**) (*m/z* 767.3970 $[M+2H]^{2+}$) of *P. norimbergensis* wild type, *P. norimbergensis* ∆*pdnM* and *P. norimbergensis* ∆*pdnI* and *P. horticolens* soft agar extracts after 3 days.

Generation of *pdnM*^{hort} mutant and its negative control mutant of *P. norimbergensis* DSM11628

Two gene fragments were amplified by PCR with the primer pairs Pnori-pdnL-fw-NEB/ Pnori-pdnL-rv-NEB (template: genomic DNA of *P. norimbergensis*) and Phort-pdnM-fw-NEB/ Phort-pdnM-rv-NEB (template: genomic DNA of *P. horticolens*) using Phusion Polymerase. The obtained amplicons were purified using the Monarch® DNA Gel Extraction Kit (New England Biolabs) and assembled with *Xho*I/*Pac*I-restricted pGL42a-T251A-Δ*PnNRPS* using NEBuilder (New England Biolabs), resulting in pGL42a-T251A-pdnLM^{hort}. Another gene fragment was amplified by PCR with the primer pair Pnori-pdnN-fw-KpnI/ Pnori-pdnN-rv-SacI (template: genomic DNA of *P. norimbergensis*) using Phusion Polymerase. The obtained amplicon was purified using the Monarch® DNA Gel Extraction Kit and inserted into pCR bluntII vector to give pCR bluntII-pdnN. This plasmid was restricted with *Kpn*I/*Sac*I and the obtained fragment was ligated into *Kpn*I/*Sac*I-restricted pGL42a_T251A-pdnLM^{hort}, generating pGL42a_T251A-pdnM^{hort}Swap.
The partial *pdnM* gene fragment was amplified by PCR with the primer pair Pnori-PdnM-fw-NEBneg / Pnori-PdnM-rv-NEBneg (template: genomic DNA of *P. norimbergensis*) using Phusion Polymerase. The obtained amplicon was purified using the Monarch® DNA Gel Extraction Kit and assembed with *Xho*I/*Pac*I-restricted pGL42a-T251A-Δ*PnNRPS* using NEBuilder, resulting in pGL42a_T251A-pdnM. The *Kpn*I/*Sac*I-restricted gene fragment derived from pCR bluntII-pdnN was cloned into *Kpn*I/*Sac*I-restricted pGL42a_T251A-pdnM, resulting pGL42a_T251A-pdnM-tet-N.
P. norimbergensis DSM11628 was grown in 2 mL LB medium at 30 °C with orbital shaking. LB medium was inoculated from the overnight culture (1/100 dilution), before being incubated at 30 °C with orbital shaking until an OD$_{600}$ of 0.4 to 0.6 was reached. The culture was centrifuged at 3,000 × *g*, then the supernatant was discarded. The cell pellet was resuspended in 300 mM sucrose and centrifuged again. After repeating this washing step three times, the washed cells were resuspended in 300 mM sucrose and then subjected to electroporation (2,500 V) with pGL42a_T251A-pdnM^{hort}Swap (*ca.* 1 µg) and pGL42a_T251A-pdnM-tet-N, individually, at room temperature. Transformed cells were cultured in 1 mL LB medium for 4 h at 30 °C with orbital shaking and then plated on LB agar plates containing tetracycline (60 µg · mL^{-1}). After 3 days, several colonies were observed and transferred to LB agar plates with tetracycline (60 µg · mL^{-1}) and D,L-*p*-chlorophenylalanine (2 mg · mL^{-1}). Obtained mutants were cultured and genomic DNA was extracted using MasterPure DNA purification kit (Epicentre) and then checked by PCR using the primer pairs Pnori-pdnL-fw2/Phort-pdnM-rv, Phort-pdnMspecific-fw/Phort-pdnMspecific-rv, Phort-pdnM-fw/Pnori-pdnN-rv, TETforward/Pnori-pdnOspecific-rv for *P. norimbergensis pdnM*^{hort} mutants, and Pnori-pdnM-fw/Pnori-pdnN-rv for *P. norimbergensis pdnM-tet-N* mutants.

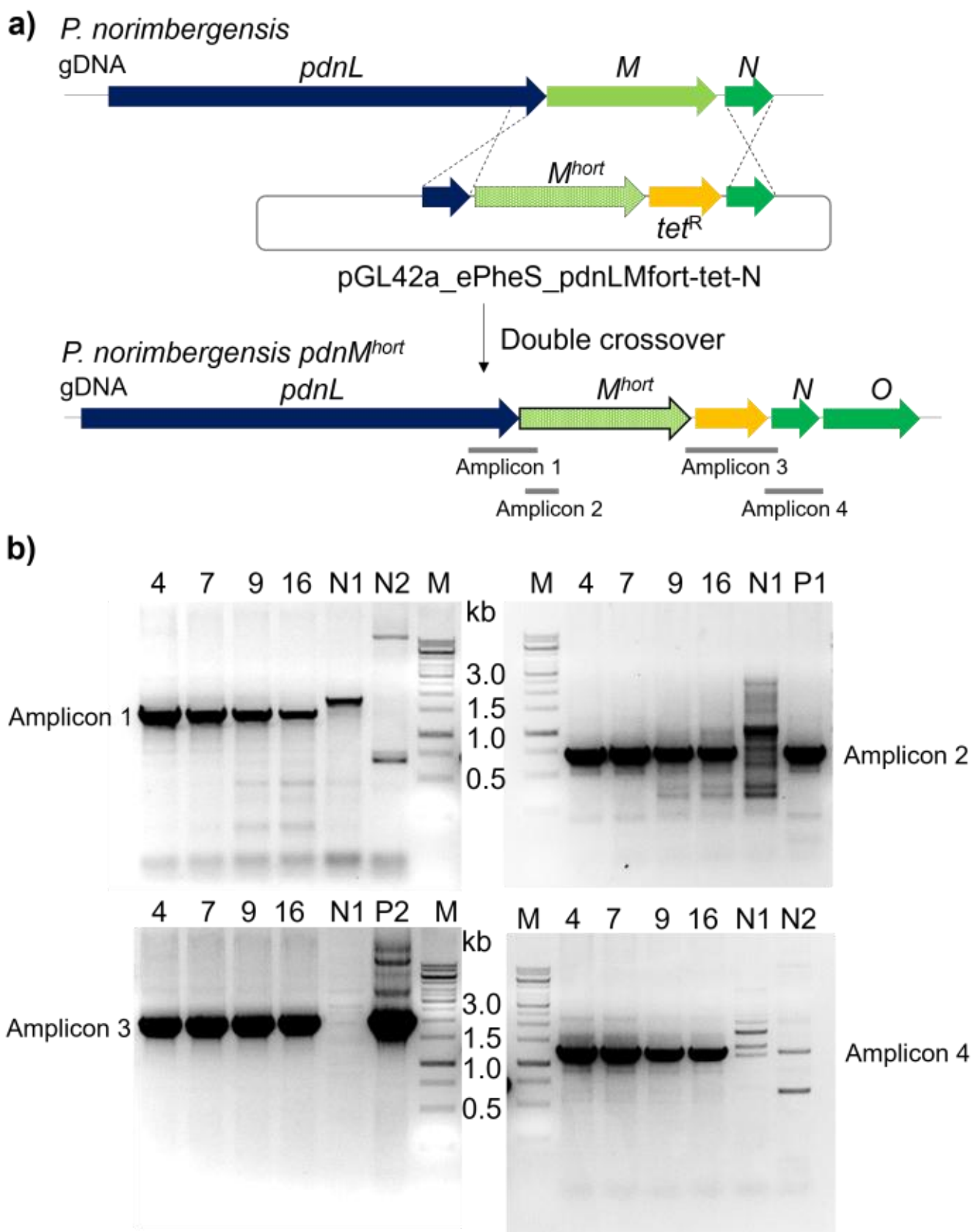

Figure S46. Generation and PCR confirmation of double-crossover mutants *P. norimbergensis pdnM*^{hort}. a) Schematic *pdnM* gene swap in *P. norimbergensis* by double-crossover event. b) PCR confirmation of *P. norimbergensis pdnM*^{hort}. Amplicons 1–4 correspond to those in schematic illustrated in a). Template DNA: genomic DNA of *P. norimbergensis pdnM*^{hort} mutants (4, 7, 9, 16), genomic DNA of *P. norimbergensis* wild type (N1), pGL42a_T251A-pdnM^{hort}Swap (N2, P2), genomic DNA of *P. horticolens* wild type (P1). M: DNA size marker. The estimated size of amplicons obtained by using the primer pairs Pnori-pdnL-fw2/Phort-pdnM-rv (1401 bp), Phort-pdnMspecific-fw/ Phort-pdnMspecific-rv (694 bp), Phort-pdnM-fw/Pnori-pdnN-rv (1915 bp), TETforward/Pnori-pdnOspecific-rv (1208 bp).

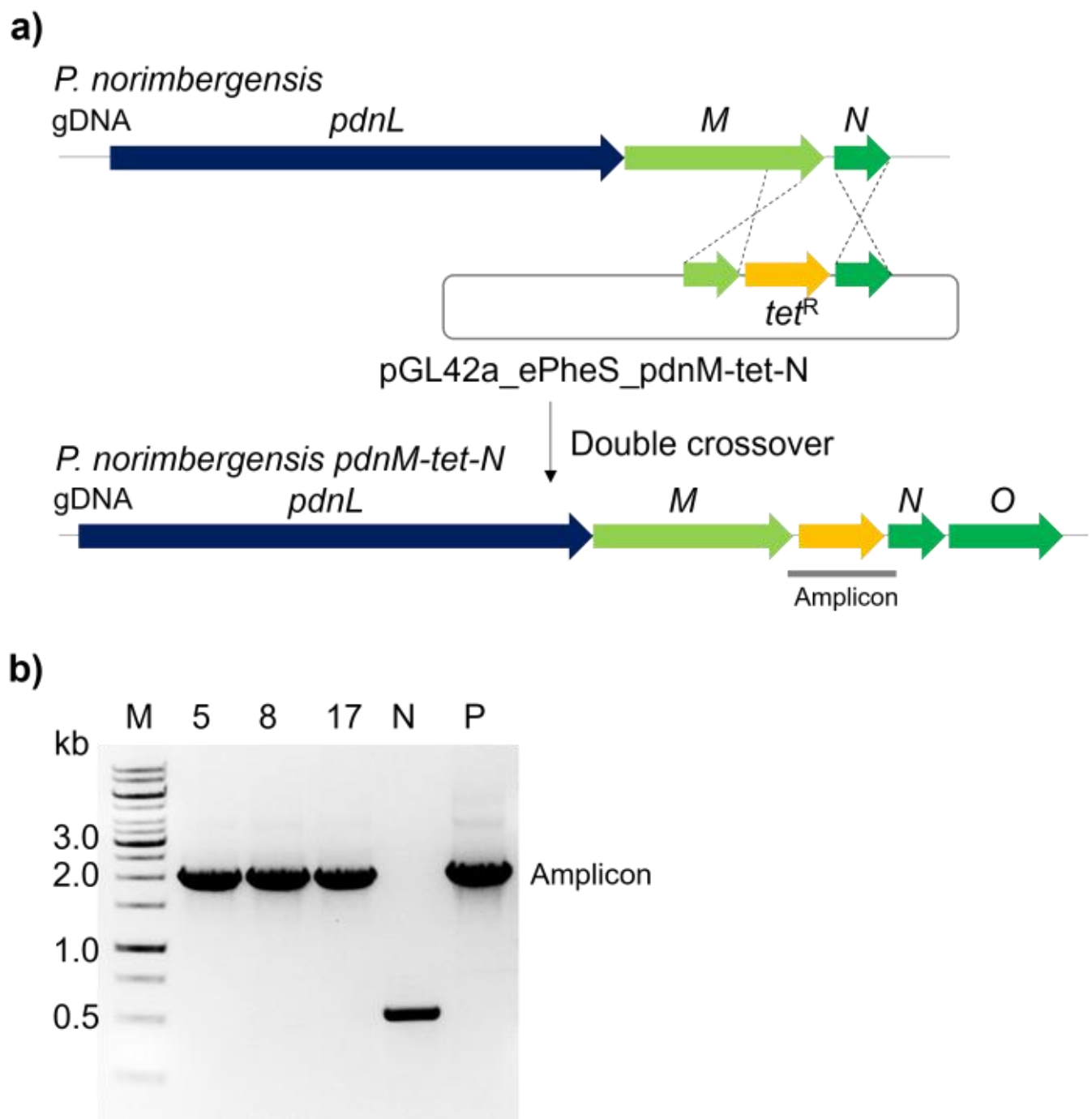

Figure S47. Preparation and PCR confirmation of double-crossover mutants *P. norimbergensis pdnM-tet-N*. a) Schematic *tet*^{R} gene insertion in *P. norimbergensis* by double-crossover event. b) PCR confirmation of *P. norimbergensis pdnM-tet-N*. Amplicon corresponds to those in schematic illustrated in a). Template DNA: genomic DNA of *P. norimbergensis pdnM-tet-N* mutants (5, 8, 17), genomic DNA of *P. norimbergensis* wild type (N), pGL42a_T251A-pdnM-tet-N (P). M: DNA size marker. The estimated size of amplicons obtained by using the primer pair Phort-pdnM-fw/Pnori-pdnN-rv (mutant: 1948 bp, wild-type: 529 bp).

Extraction and quantitative analysis of pandorachelin production by *P. norimbergensis*, *P. horticolens*, *P. norimbergensis pdnM*^{hort}, and *P. norimbergensis pdnM-tet-N*

P. norimbergensis, P. horticolens, P. norimbergensis pdnM^{hort}*,* and *P. norimbergensis pdnM-tet-N* were grown overnight in MM9 medium without trace elements (supplemented with 60 µg · mL^{-1} tetracycline for mutant strains) at 30 °C with orbital shaking at 150 rpm and. 50 mL volumes of MM9 medium without trace elements (supplemented with 60 µg · mL^{-1} tetracycline for mutant strains) in 100 mL baffled Erlenmeyer flasks were inoculated from the overnight cultures (starting OD_{600} of 0.1), before being incubated at 30 °C with orbital shaking at 150 rpm for three days. After cultivation, the cultures were centrifuged at 8,000 × *g* for 10 min at 15 °C. The supernatant was mixed withAmberlite® XAD16 resin (2 × 4 cm) twice and then washed with 50 mL of ddH_2O. Organic compounds were eluted with 50 mL MeOH and 50 mL acetone. After the solvent was removed under reduced pressure, the residue was dissolved in 2 mL of ddH_2O. The culture experiments were performed in biological triplicates.
The RP-HPLC analysis was performed by Shimadzu CLASS-VP system. The sample (10 µL) was subjected to reversed-phase HPLC column (Eclipse plus C18, 3.0 × 150 mm, 3.5 µm, 95 Å, Agilent Technologies) using a gradient system, solvent A: H_2O + 0.1% formic acid, solvent B: acetonitrile, time program: 2 min 2% B, 2% B to 65% B in 13 min, 65% B to 2% B in 1 min, flow rate 1 mL · min^{-1}.
The area sizes at $UV_{\lambda max}$ = 220 nm of pandorachelin A (**1**) and B (**4**) from standards and above-mentioned culture extracts were manually integrated. The obtained values were calculated in Excel and then plotted using GraphPad Prism 10.6.1.

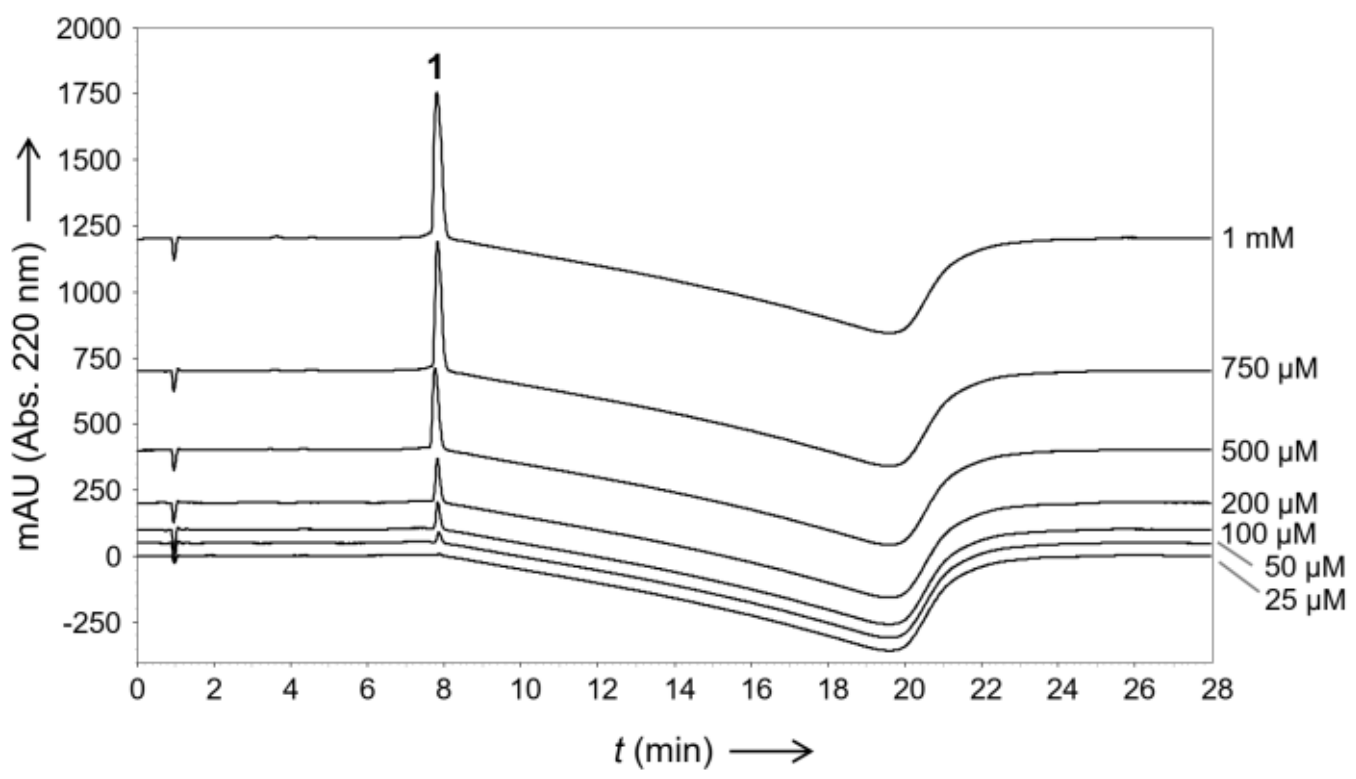

Figure S48. RP-HPLC profile of pandorachelin A (**1**) at different concentrations.

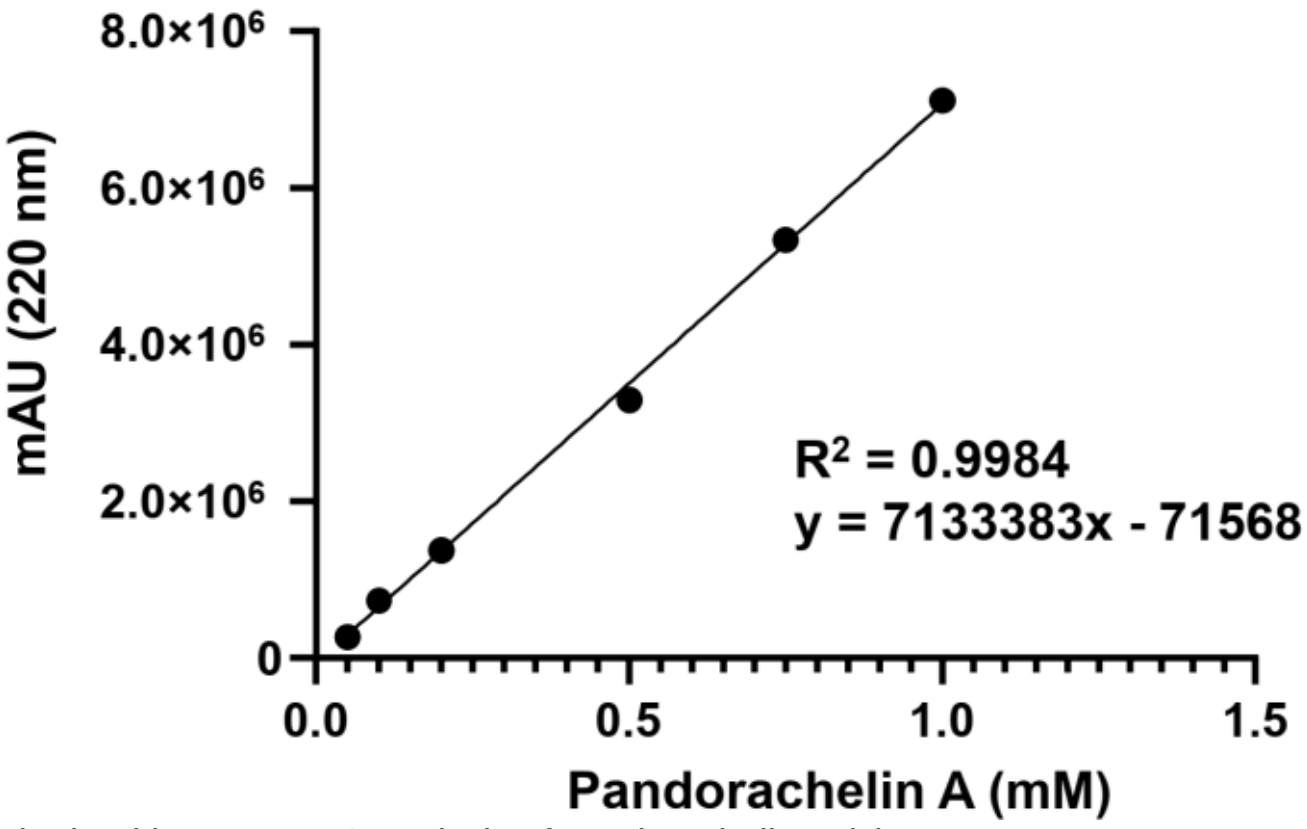

Figure S49. The standard concentration obtained by RP-HPLC analysis of pandorachelin A (**1**).

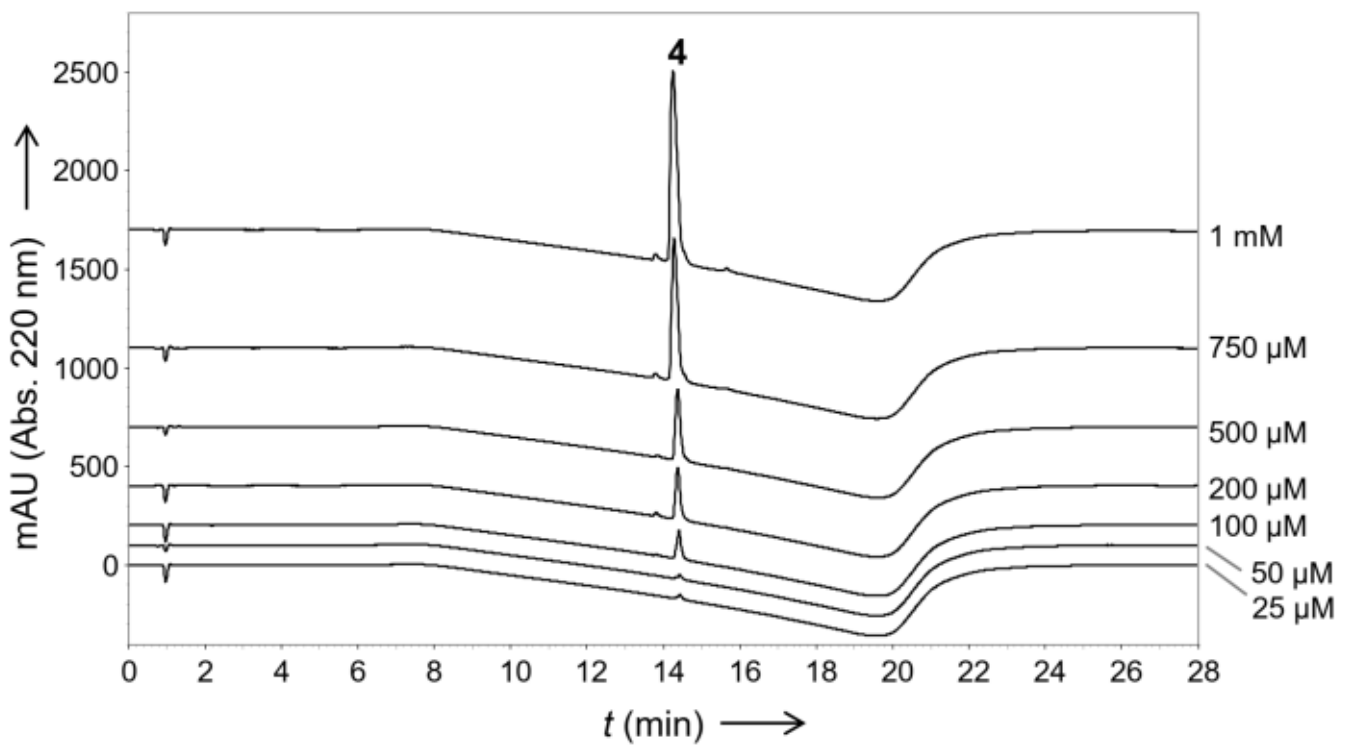

Figure S50. RP-HPLC profile of pandorachelin B (**4**) at different concentrations.

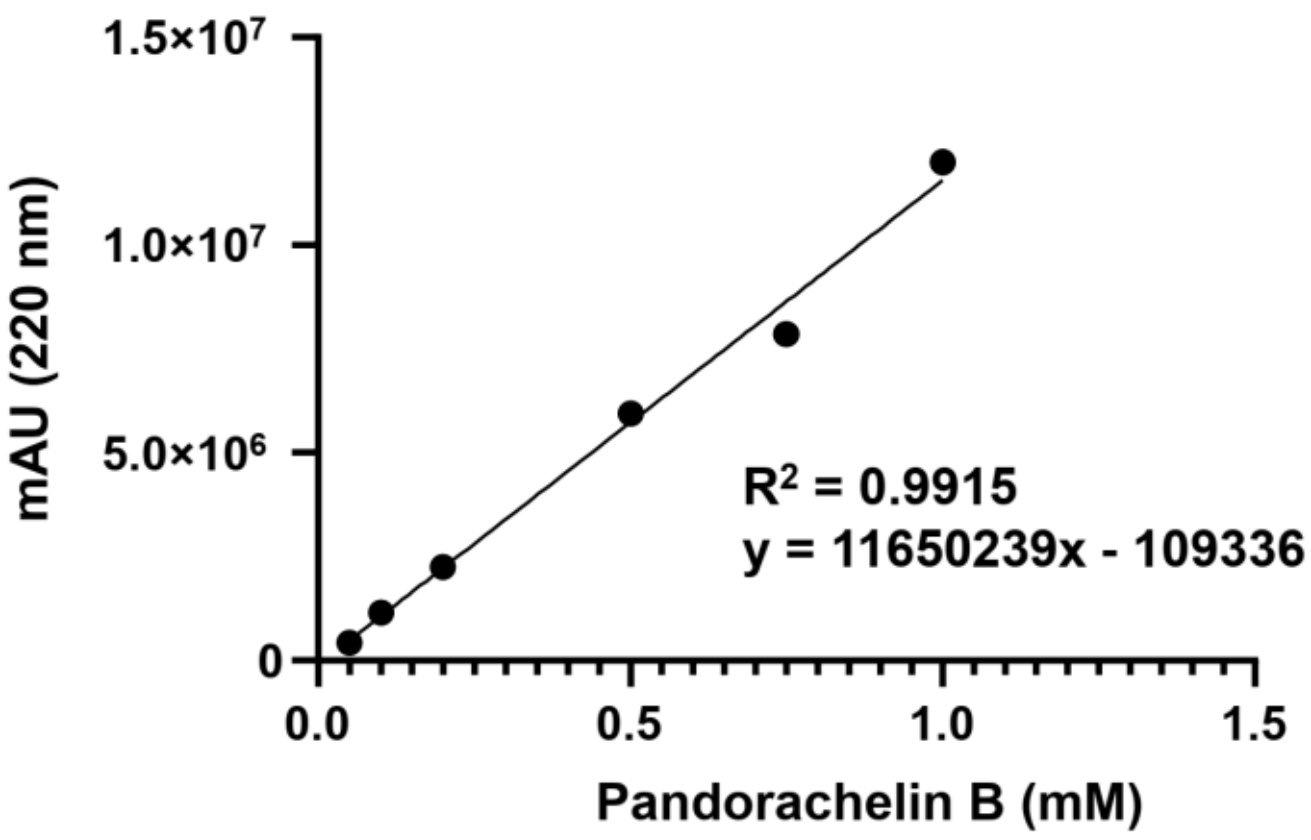

Figure S51. The standard concentration obtained by RP-HPLC analysis of pandorachelin B (**4**).

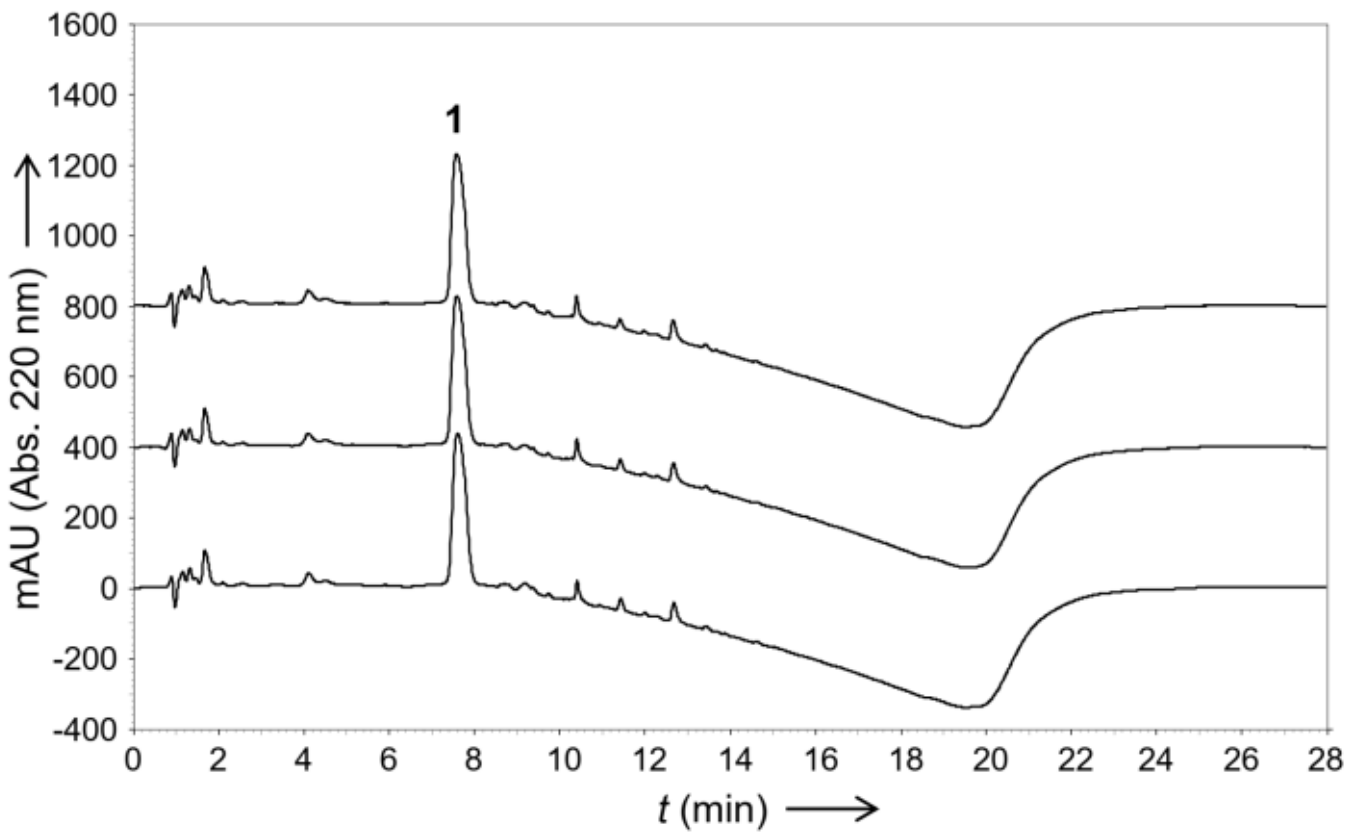

Figure S52. RP-HPLC profile of the XAD16 extracts of *P. norimbergensis* wild-type cultures. **1**: pandorachelin A.

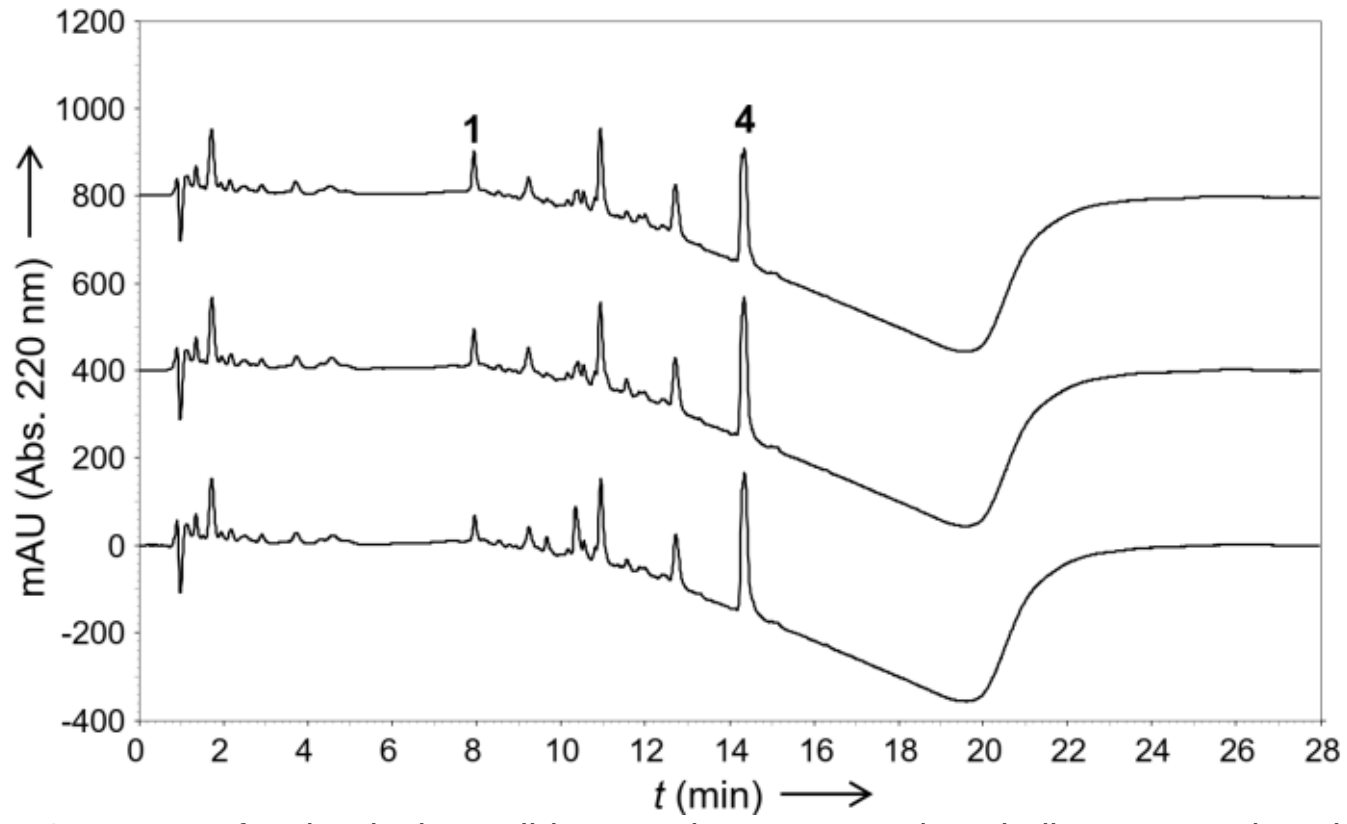

Figure S53. RP- HPLC profile of the XAD16 extracts of *P. horticolens* wild-type cultures. **1**: pandorachelin A, **4**: pandorachelin B.

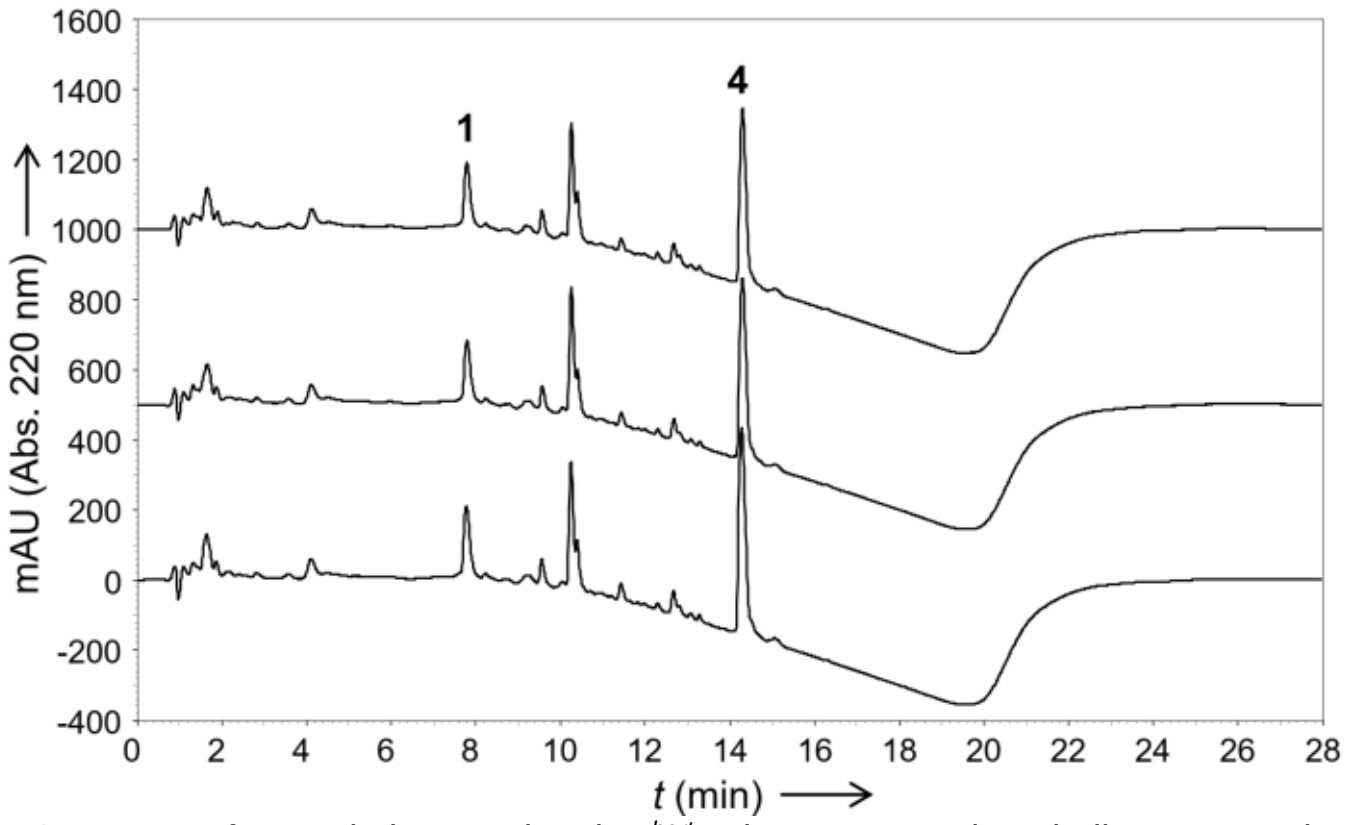

Figure S54. RP-HPLC profile of the XAD16 extracts of *P. norimbergensis pdnM^{hort}* cultures. **1**: pandorachelin A, **4**: pandorachelin B.

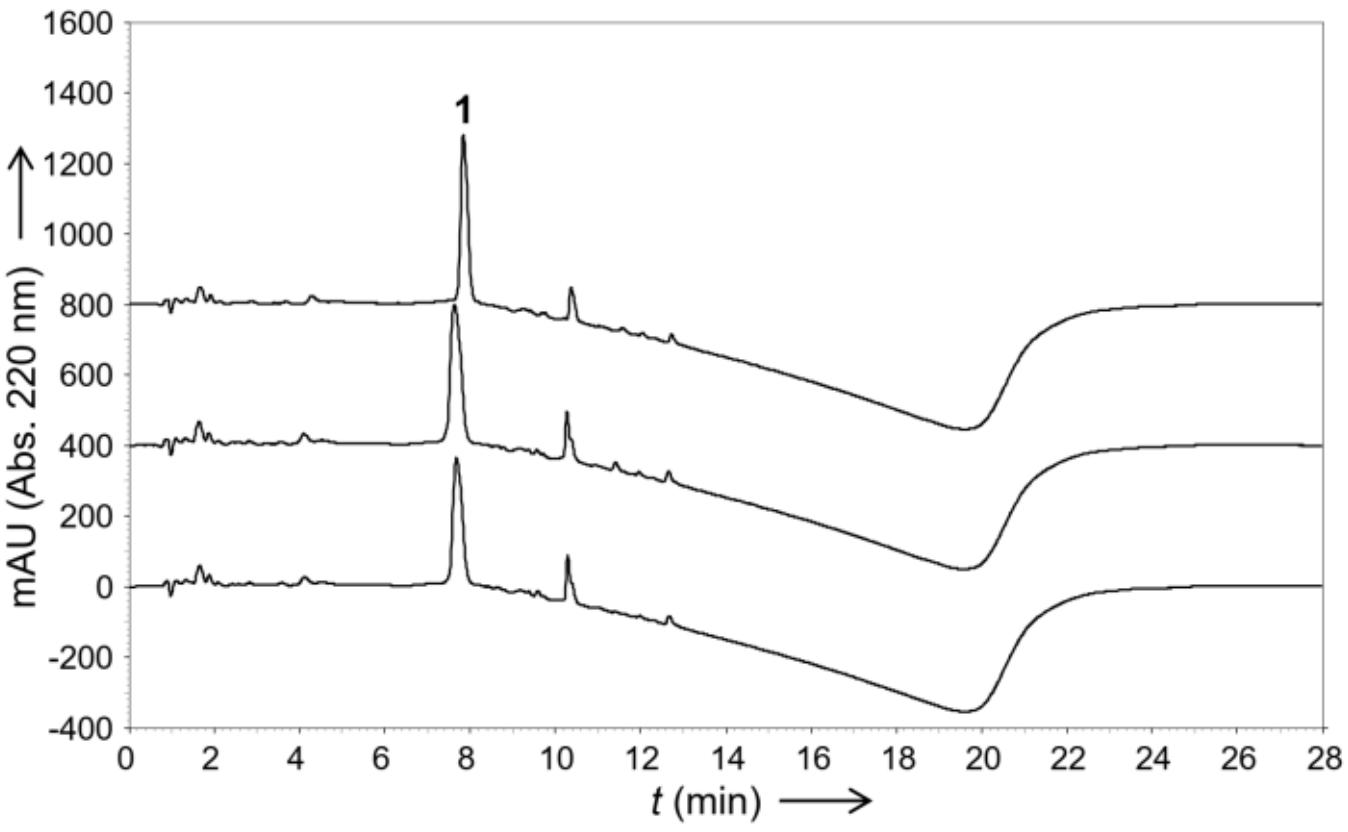

Figure S55. RP-HPLC profile of the XAD16 extracts of *P. norimbergensis pdnM-tet-N* cultures. **1**: pandorachelin A.

Table S15. Quantification of pandorachelins produced by *Pandoraea* species for 3 days. AU, absorbance unit at 220 nm. Av, average, SD, standard deviation.

Strain	Pandorachelin A (**1**) mM in 50 mL	Pandorachelin B (**4**) mM in 50 mL	OD_{600}
P. norimbergensis wild-type	0.0489	0	8.19
	0.0488	0	7.55
	0.0502	0	6.25
	Av. 0.0439	−	−
	SD. 0.0008	−	−
P. norimbergensis pdnM-tet-N	0.0297	0	4.39
	0.0376	0	4.37
	0.0307	0	4.42
	Av. 0.0327	−	−
	SD. 0.0008	−	−
P. forticolens wild-type	0.0035	0.0104	5.82
	0.0031	0.0118	5.75
	0.0023	0.0119	5.72
	Av. 0.0030	Av. 0.0114	−
	SD. 0.0008	SD. 0.0009	−
P. norimbergensis $pdnM^{fort}$	0.0102	0.0179	5.25
	0.0098	0.0189	5.50
	0.0114	0.0220	5.72
	Av. 0.0105	Av. 0.0196	−
	SD. 0.0043	SD. 0.0022	−

NMR spectra of pandorachelin A (1)

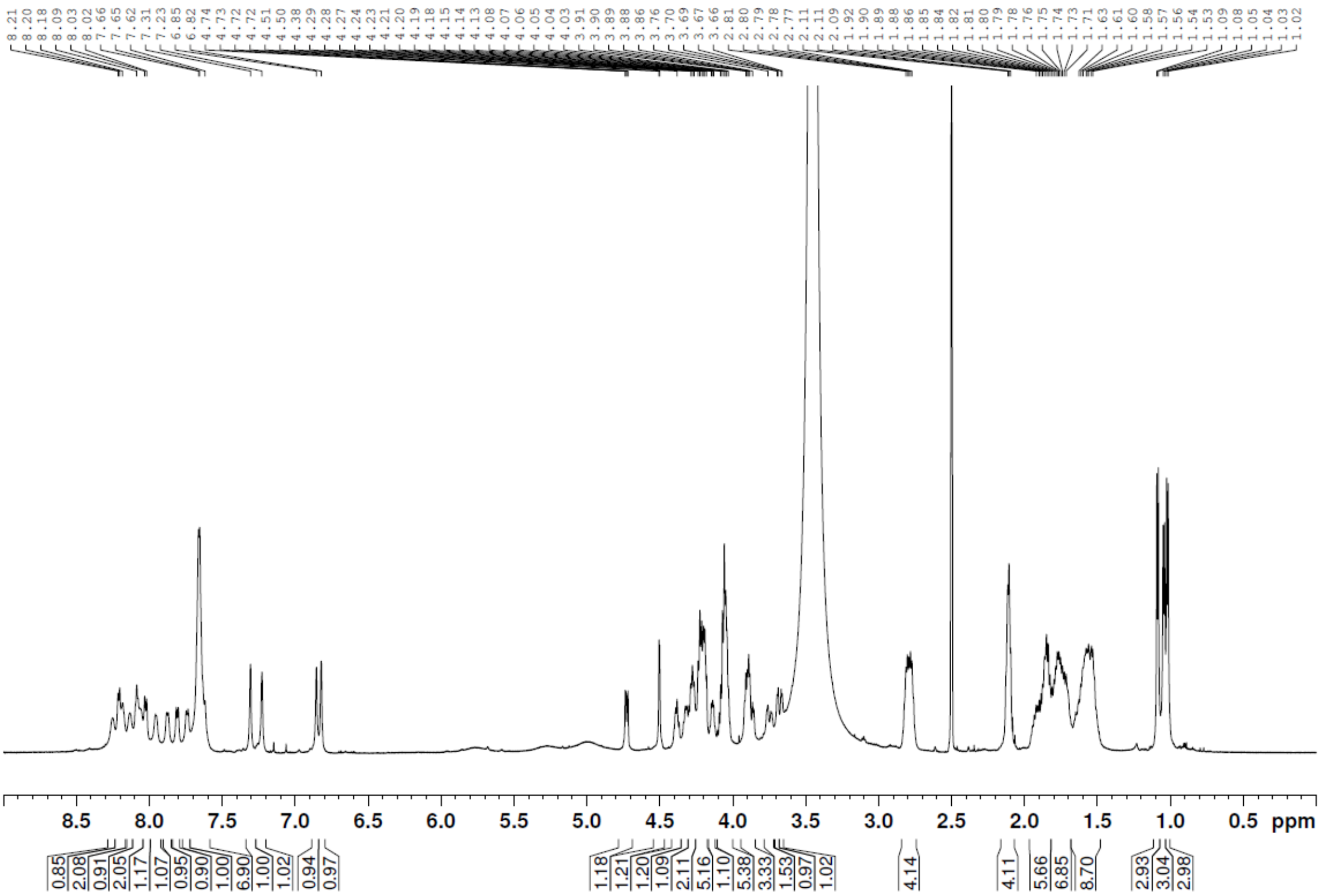

Figure S56. ¹H-NMR spectrum of pandorachelin A (**1**) in DMSO-d_6 (600 MHz).

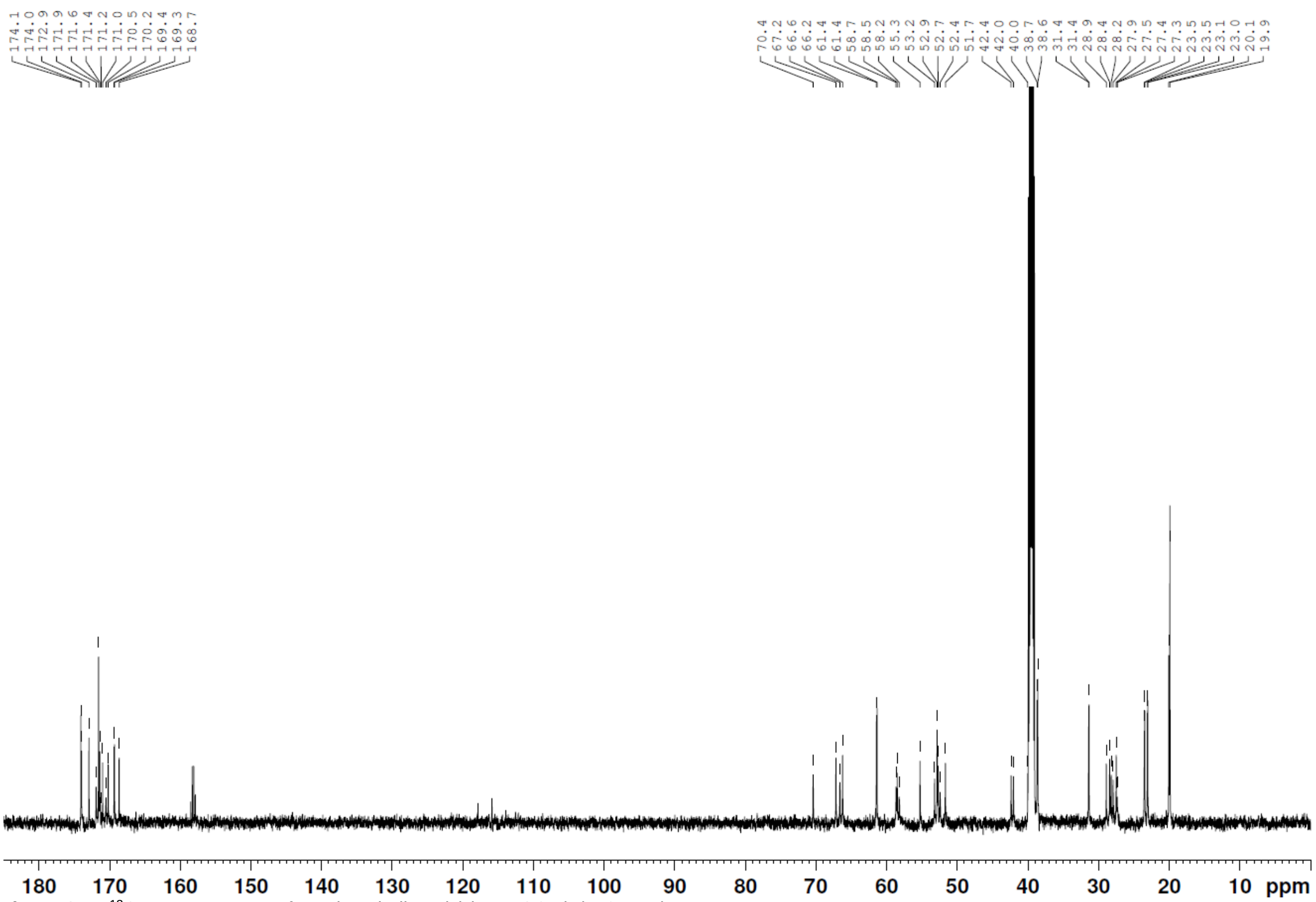

Figure S57. ¹³C-NMR spectrum of pandorachelin A (**1**) in DMSO-d_6 (150 MHz).

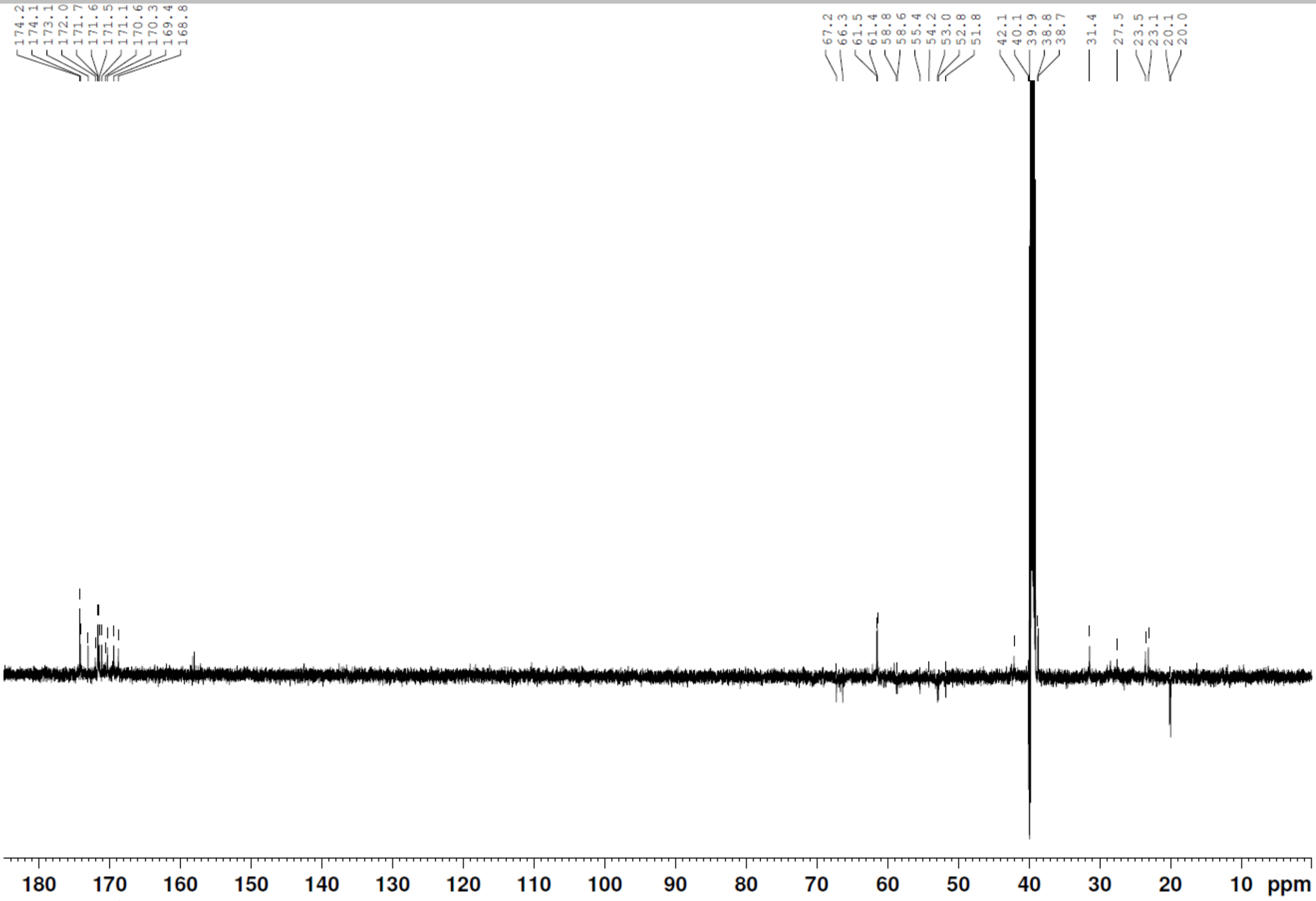

Figure S58. ^{13}C-Jmod-NMR spectrum of pandorachelin A (**1**) in DMSO-d_6 (150 MHz, C and CH_2 are positive, CH and CH_3 are negative).

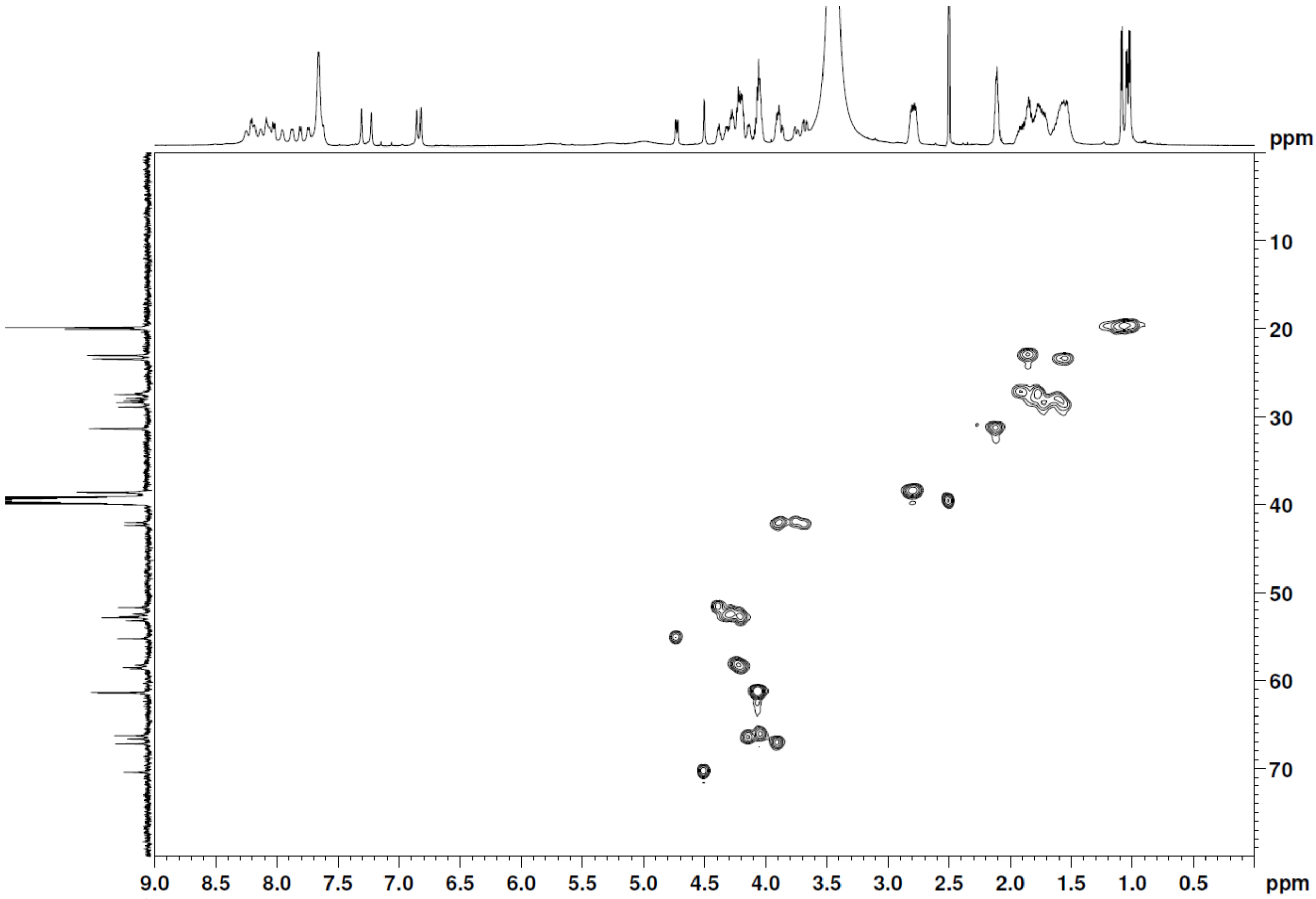

Figure S59. ^{1}H-^{13}C-HSQC spectrum of pandorachelin A (**1**) in DMSO-d_6 (600 MHz).

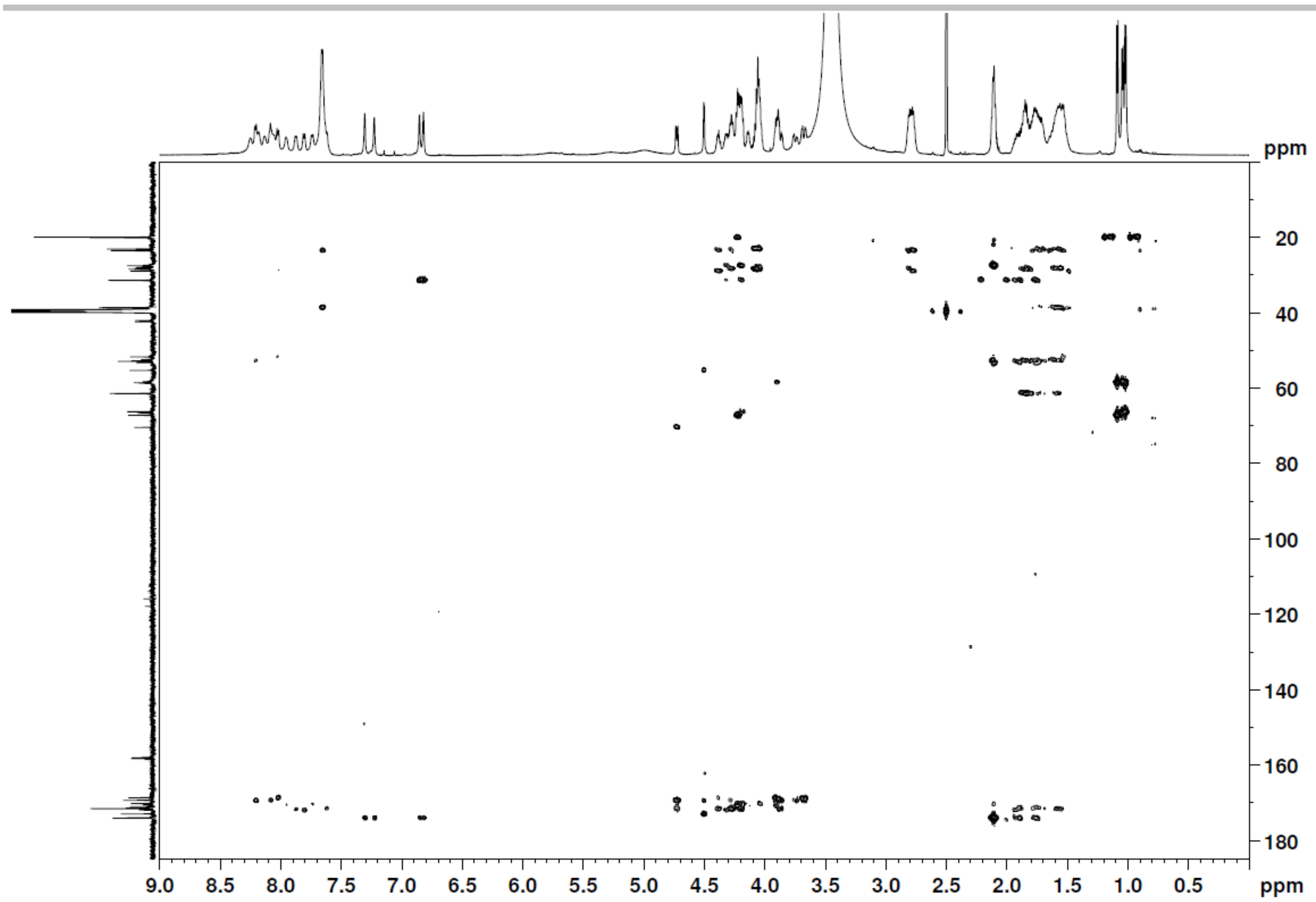

Figure S60. ^{1}H-^{13}C-HMBC spectrum of pandorachelin A (**1**) in DMSO-d_6 (600 MHz).

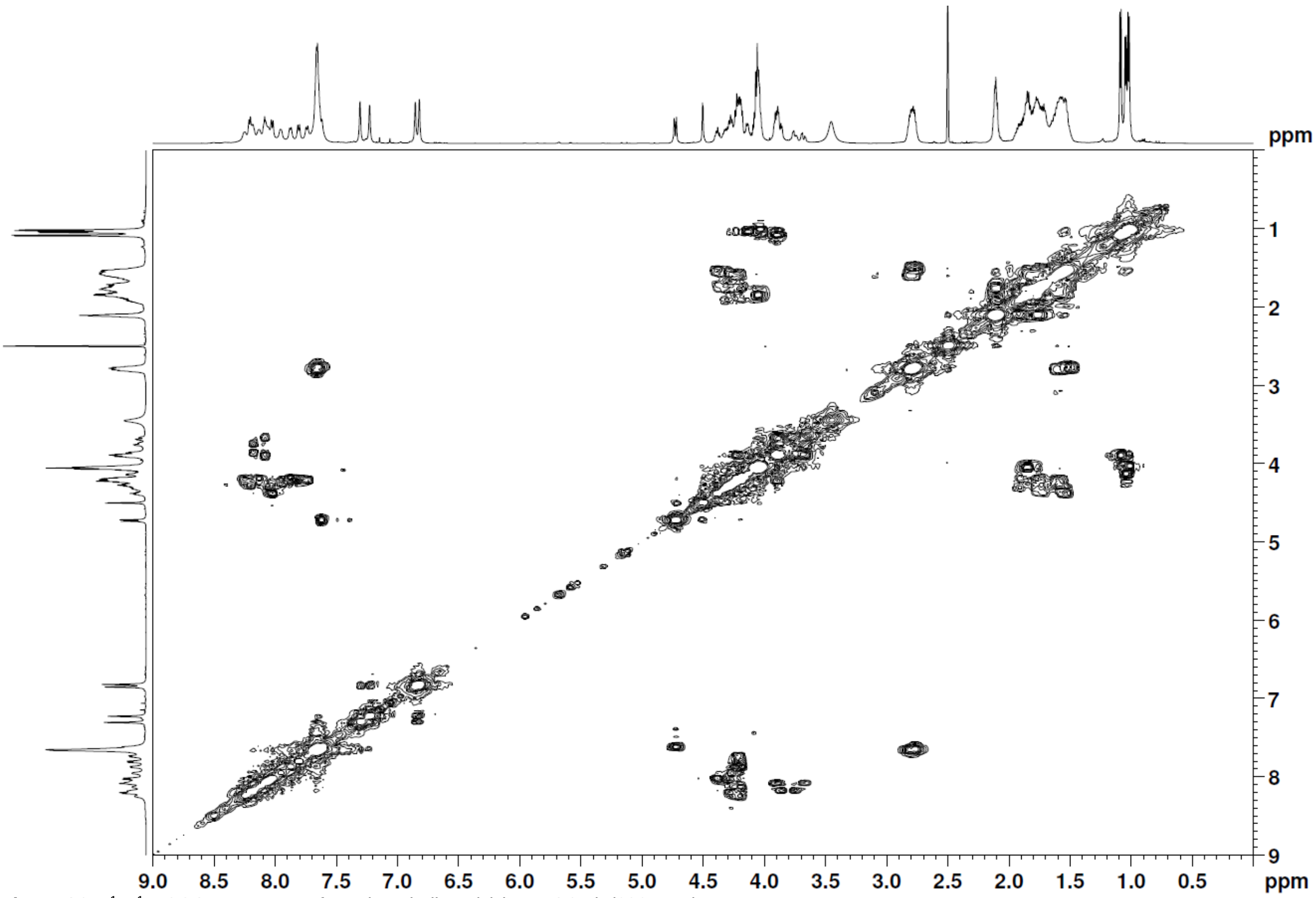

Figure S61. ^{1}H-^{1}H-COSY spectrum of pandorachelin A (**1**) in DMSO-d_6 (600 MHz).

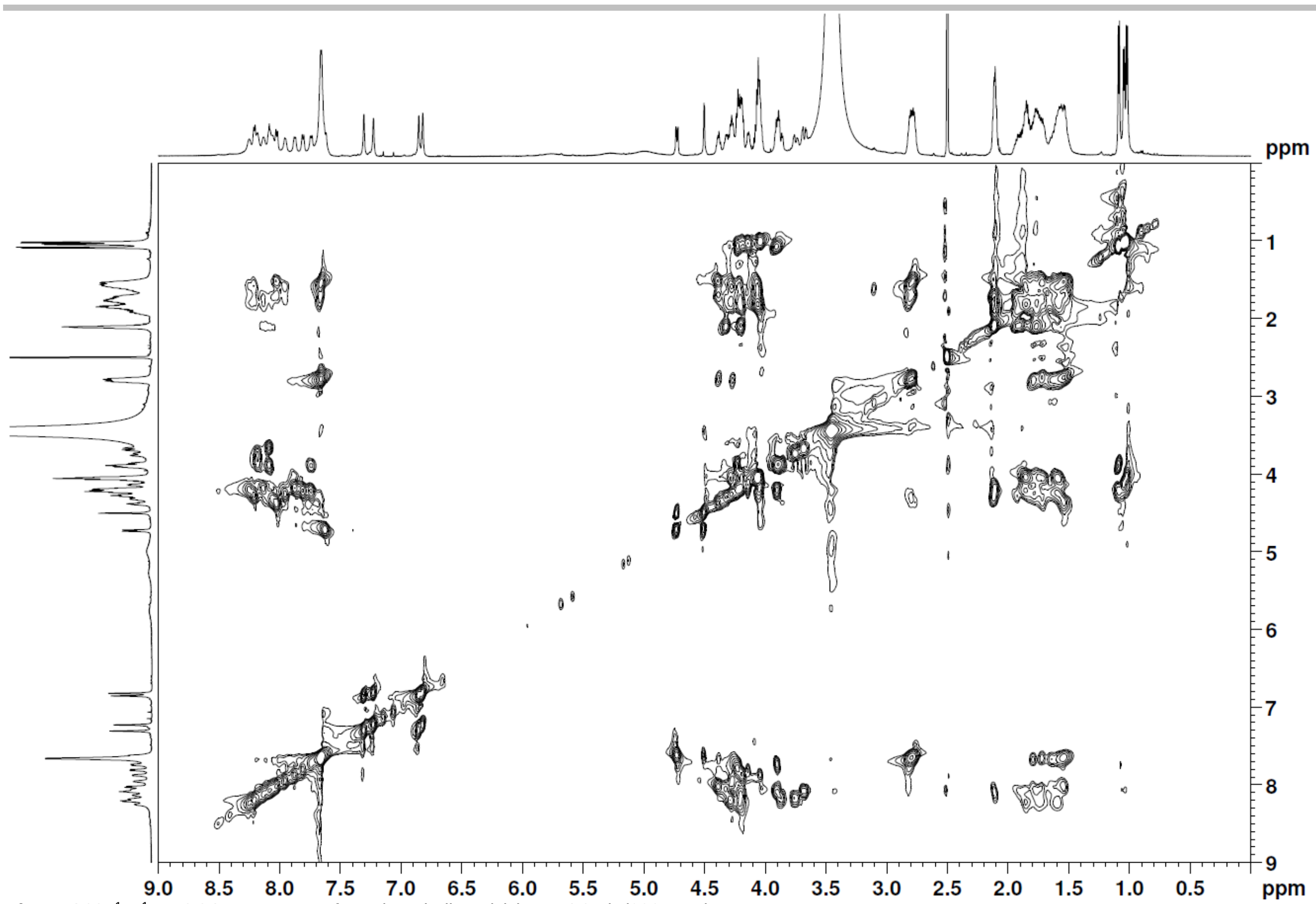

Figure S62. ^{1}H-^{1}H-TOCSY spectrum of pandorachelin A (**1**) in DMSO-d_6 (600 MHz).

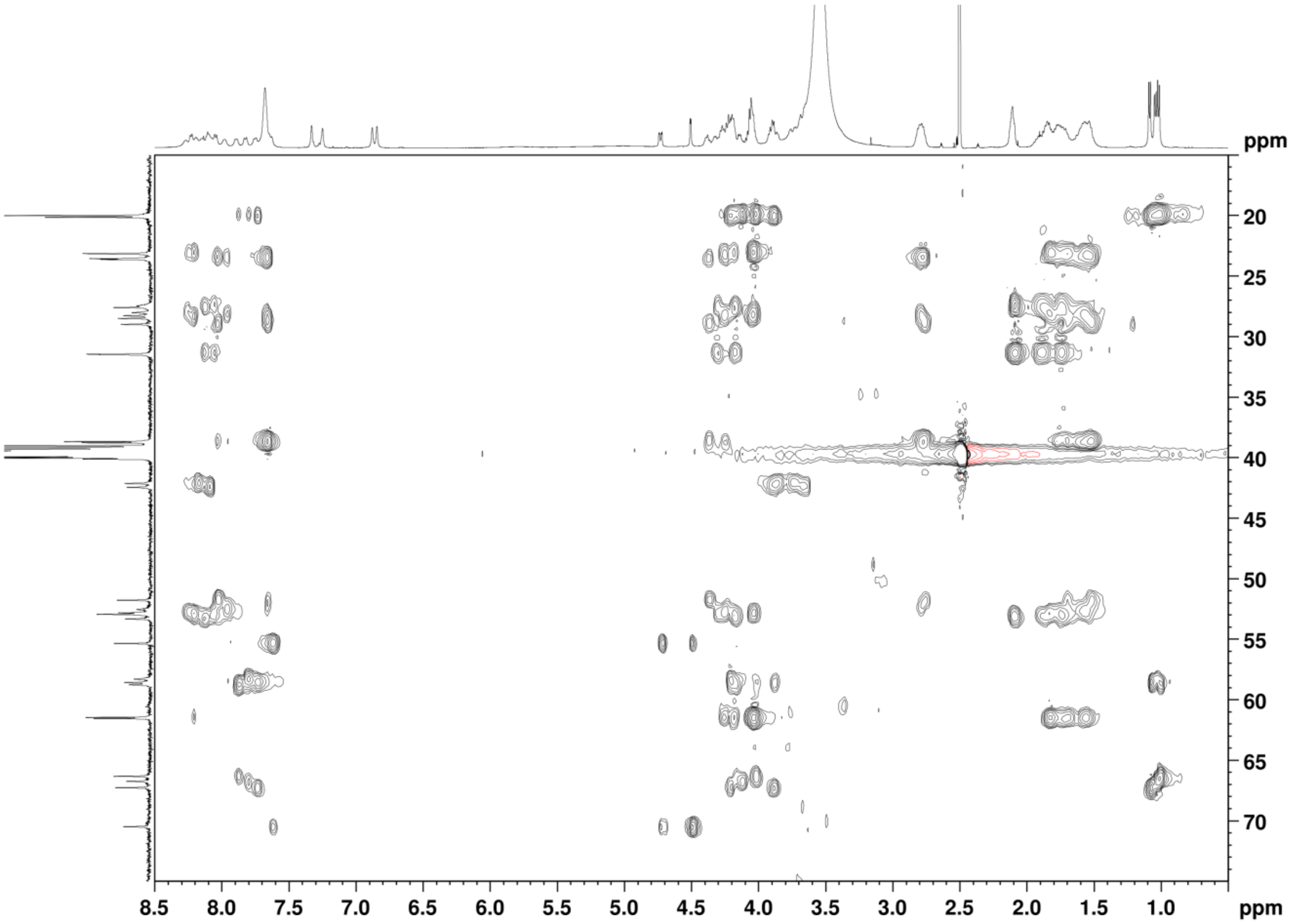

Figure S63. ^{1}H-^{13}C-HSQC-TOCSY spectrum of pandorachelin A (**1**) in DMSO-d_6 (500 MHz).

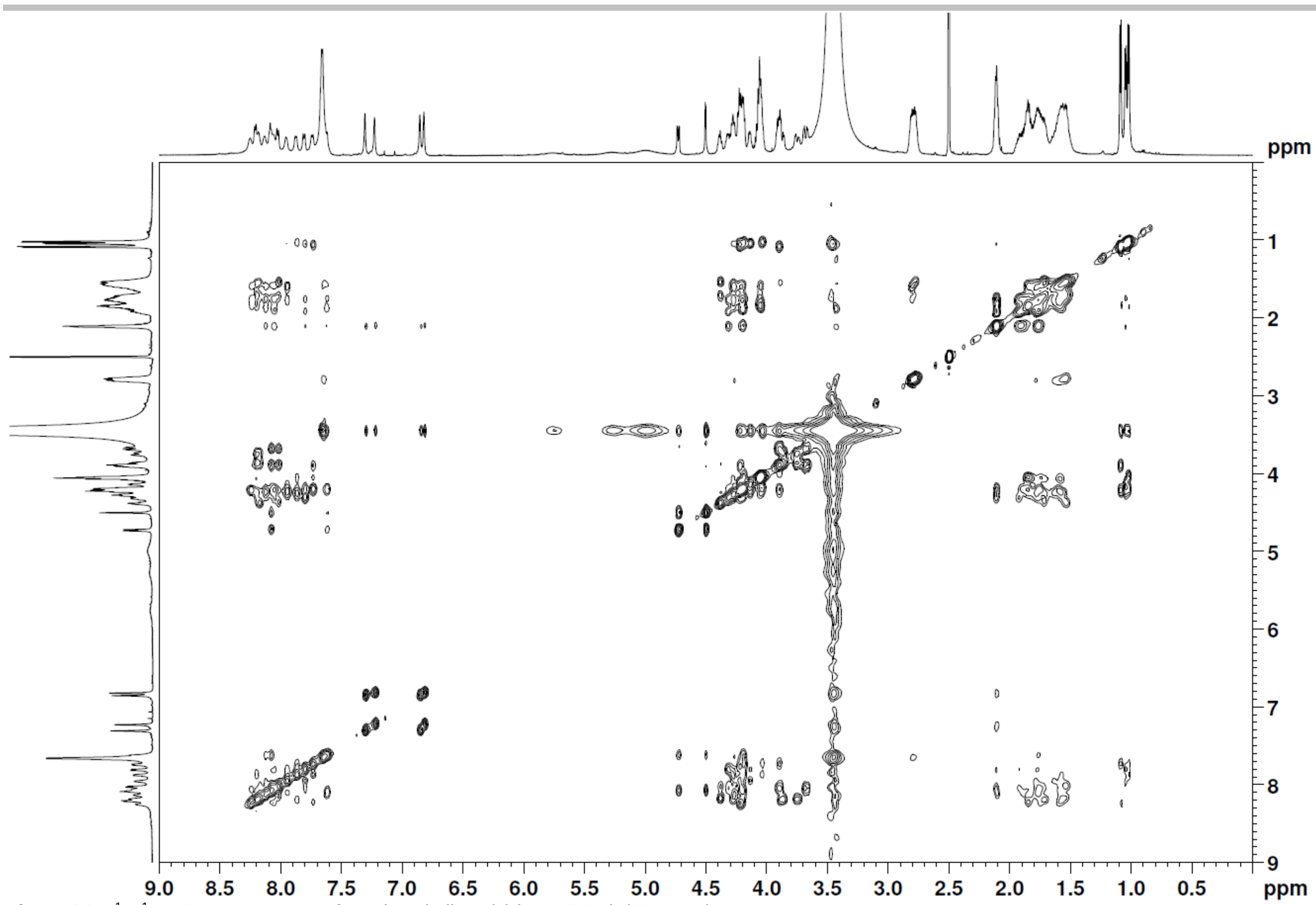

Figure S64. ^{1}H-^{1}H-NOESY spectrum of pandorachelin A (**1**) in DMSO-d_6 (600 MHz).

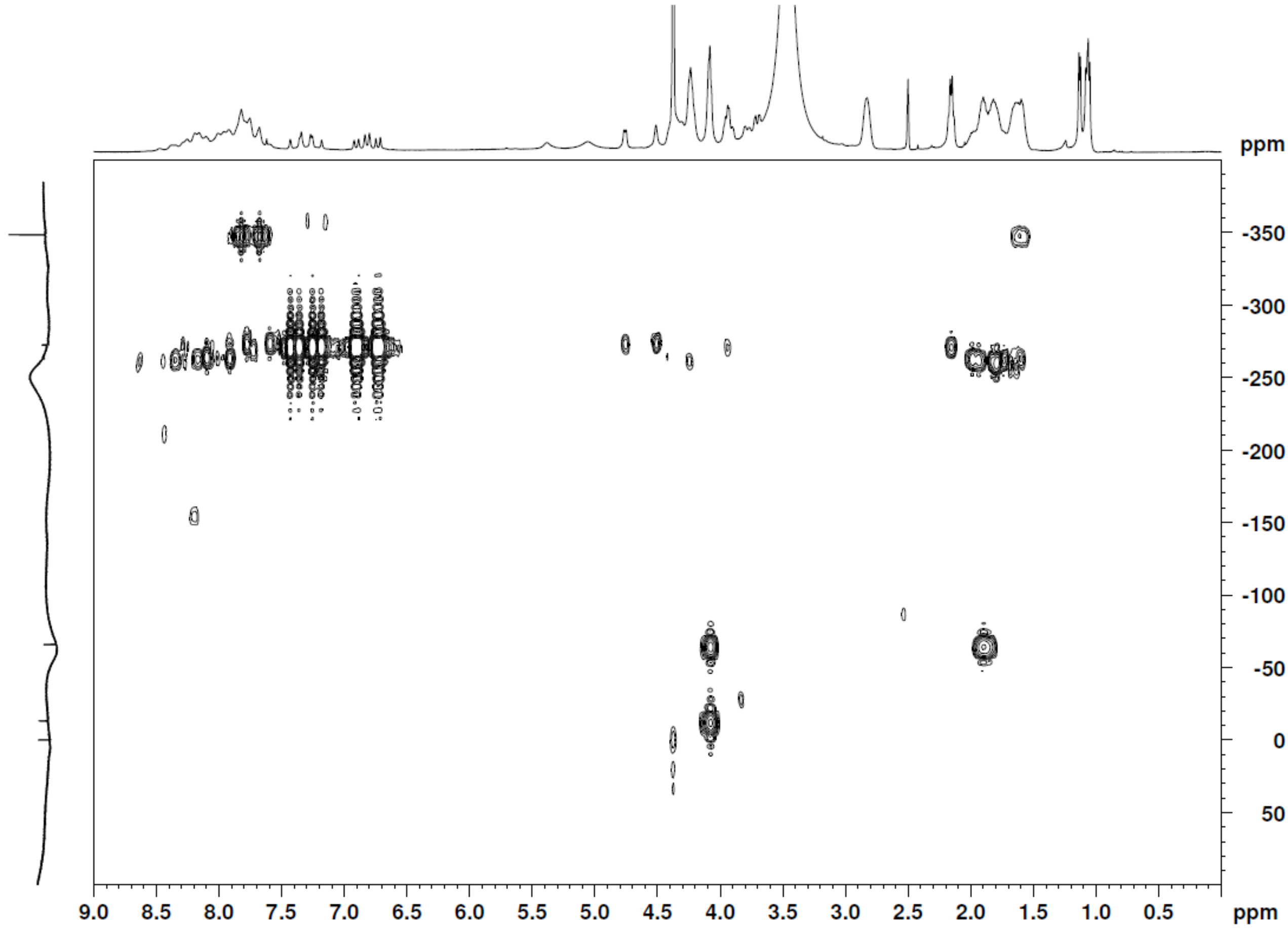

Figure S65. ^{1}H-^{15}N-HMBC spectrum of ^{15}-N-labeled pandorachelin A (**1**) in DMSO-d_6/CD_3NO_2 3:1 (500 MHz, spectrum is calibrated on CD_3NO_2).

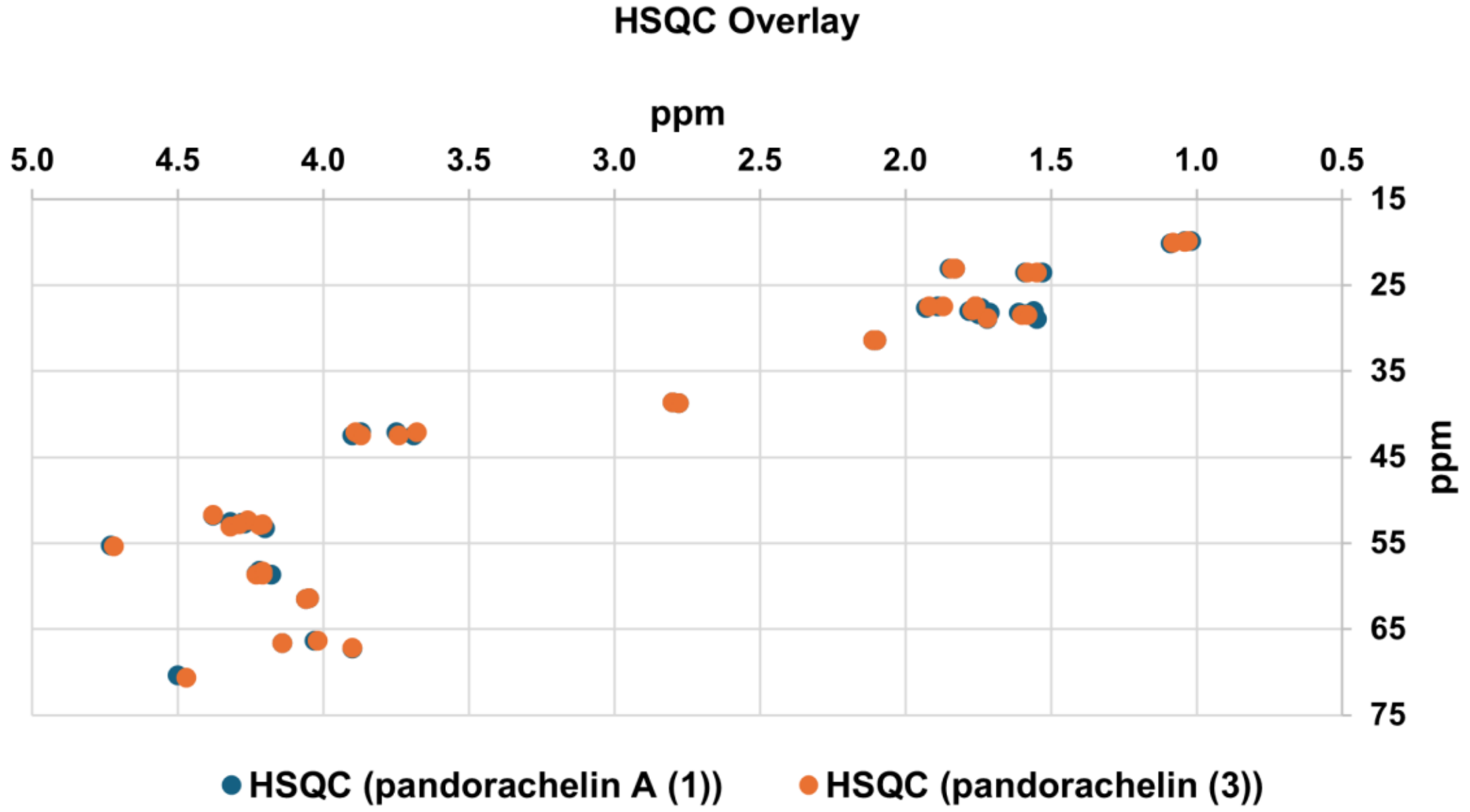

Figure S66. ^{1}H-^{13}C-HSQC spectrum comparison of pandorachelin A (**1**) in DMSO-d_6 (600 MHz) (blue) and pandorachelin (**3**) from the Bulter group (orange).

NMR spectra of pandorachelin B (4)

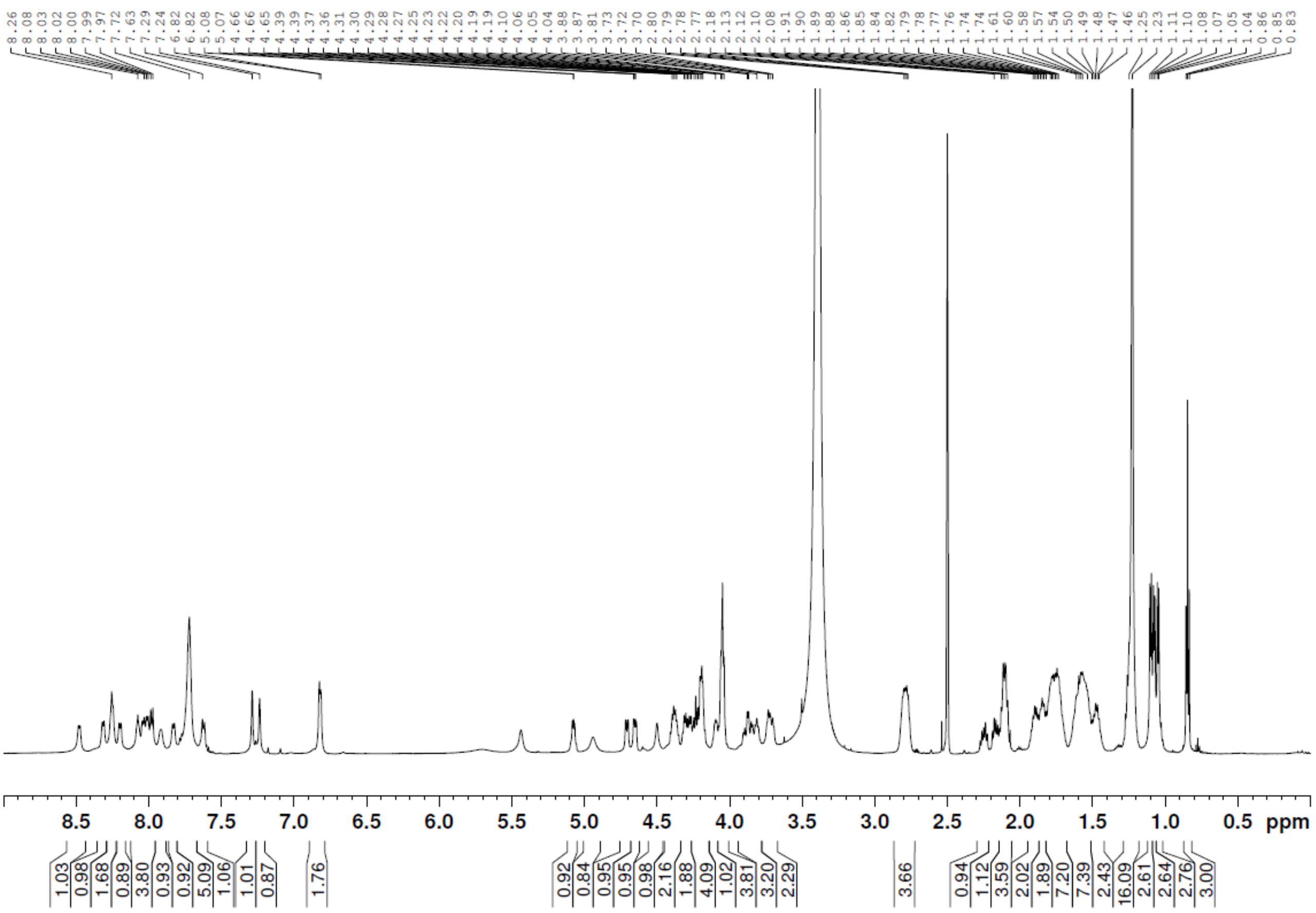

Figure S67. ^{1}H-NMR spectrum of pandorachelin B (**4**) in DMSO-d_6 (600 MHz).

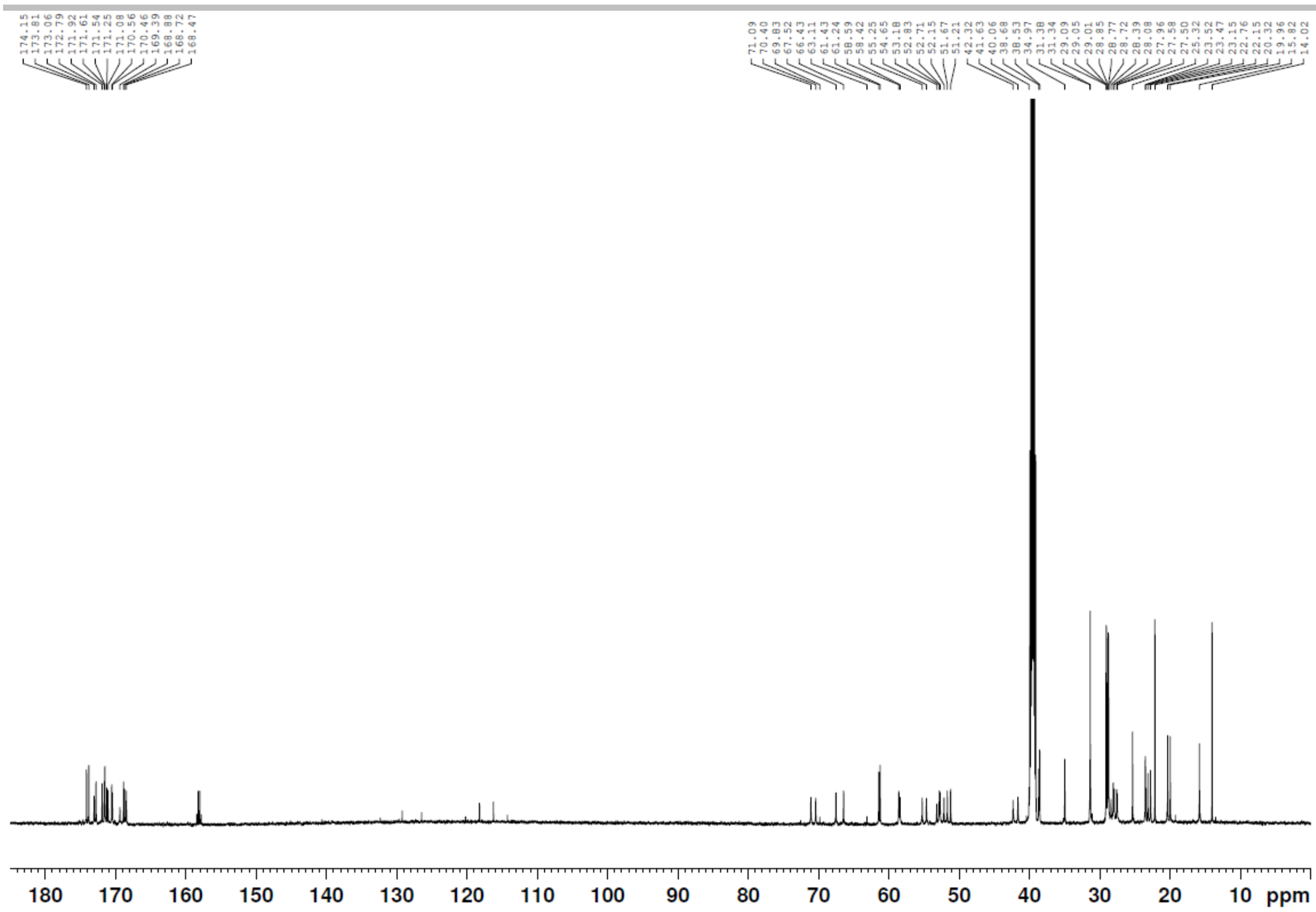

Figure S68. ^{13}C-NMR spectrum of pandorachelin B (**4**) in DMSO-d_6 (150 MHz).

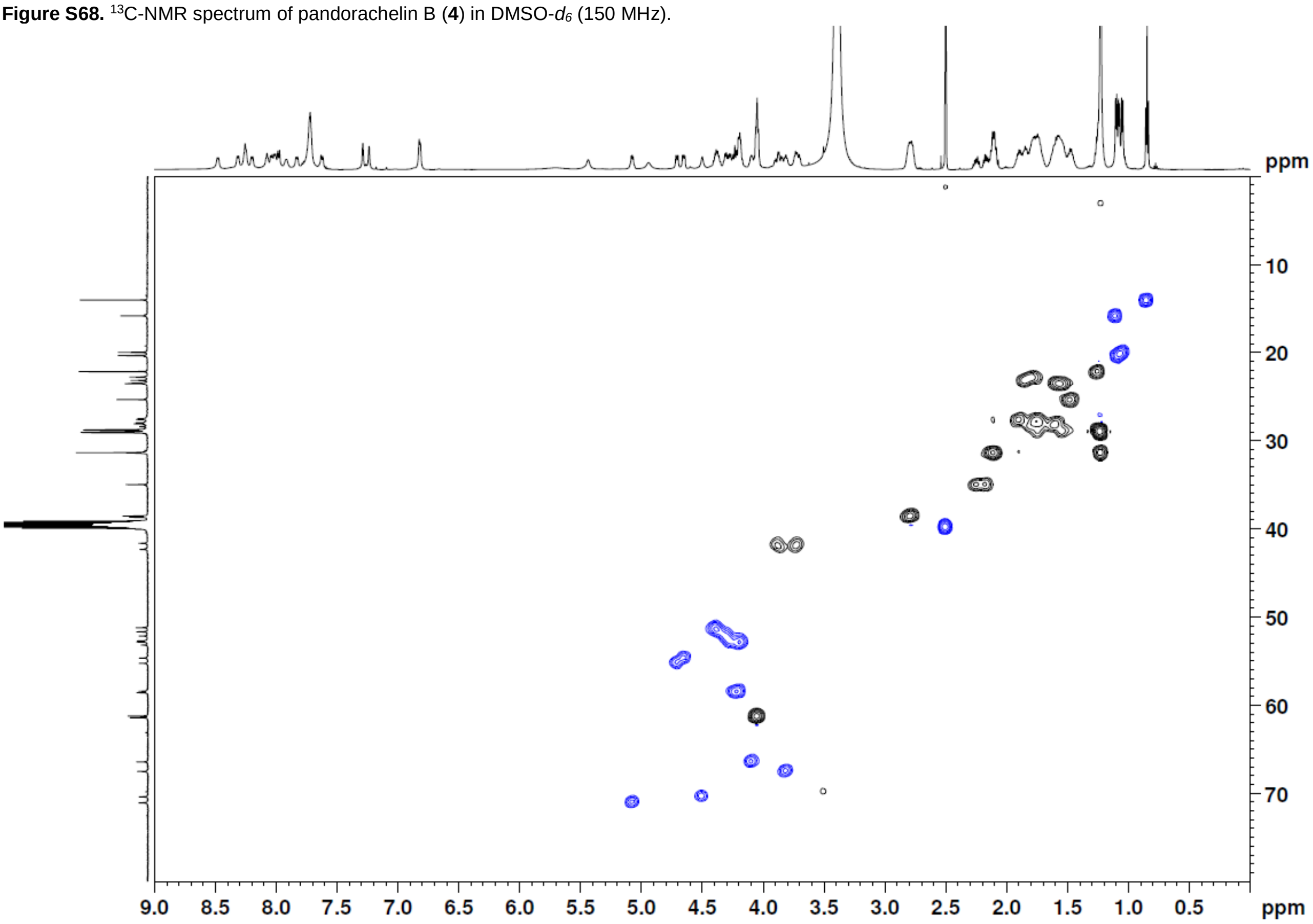

Figure S69. ^{1}H-^{13}C-HSQC spectrum of pandorachelin B (**4**) in DMSO-d_6 (600 MHz, CH and CH_3 blue colored, CH_2 black colored).

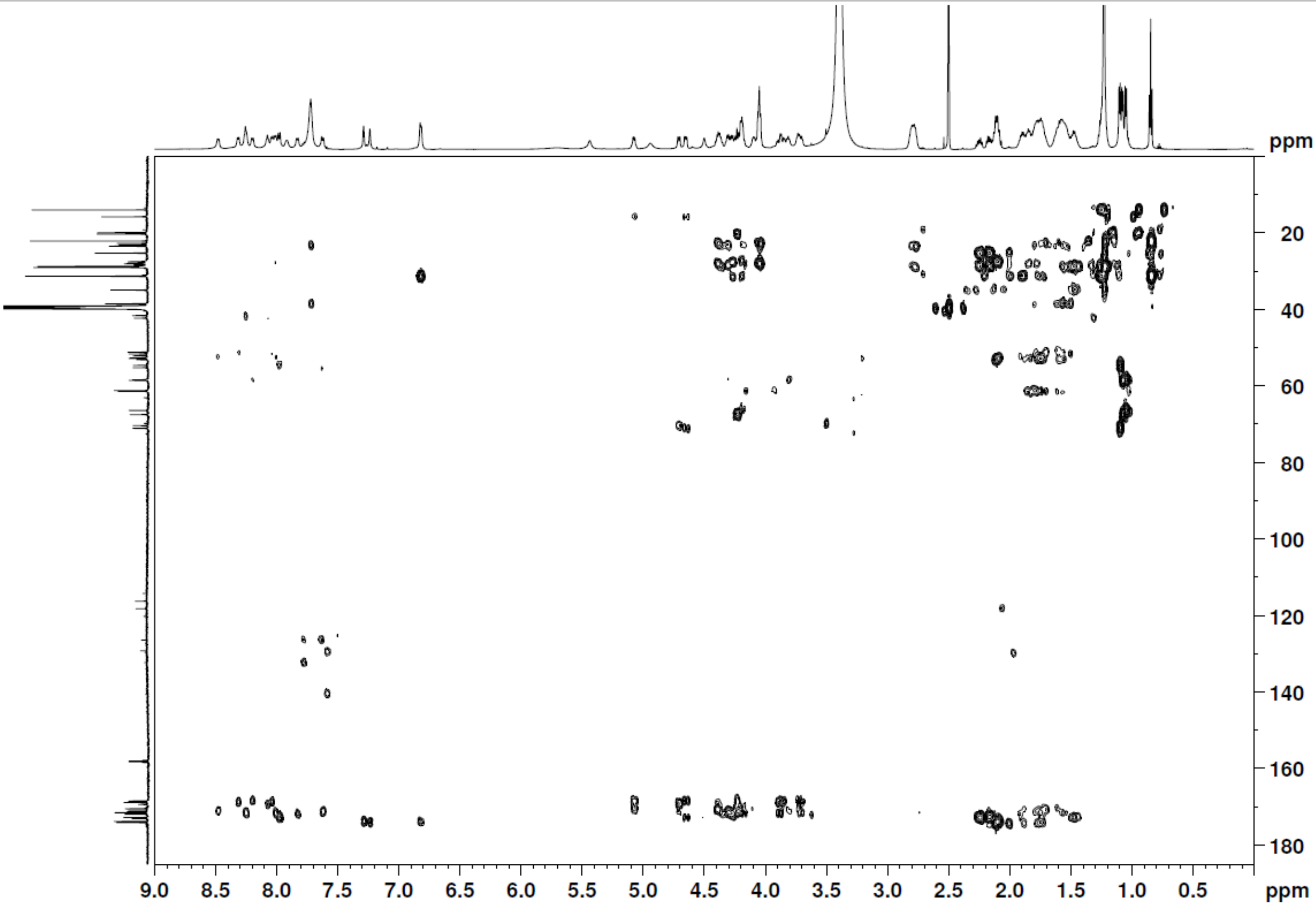

Figure S70. ^{1}H-^{13}C-HMBC spectrum of pandorachelin B (**4**) in DMSO-d_6 (600 MHz).

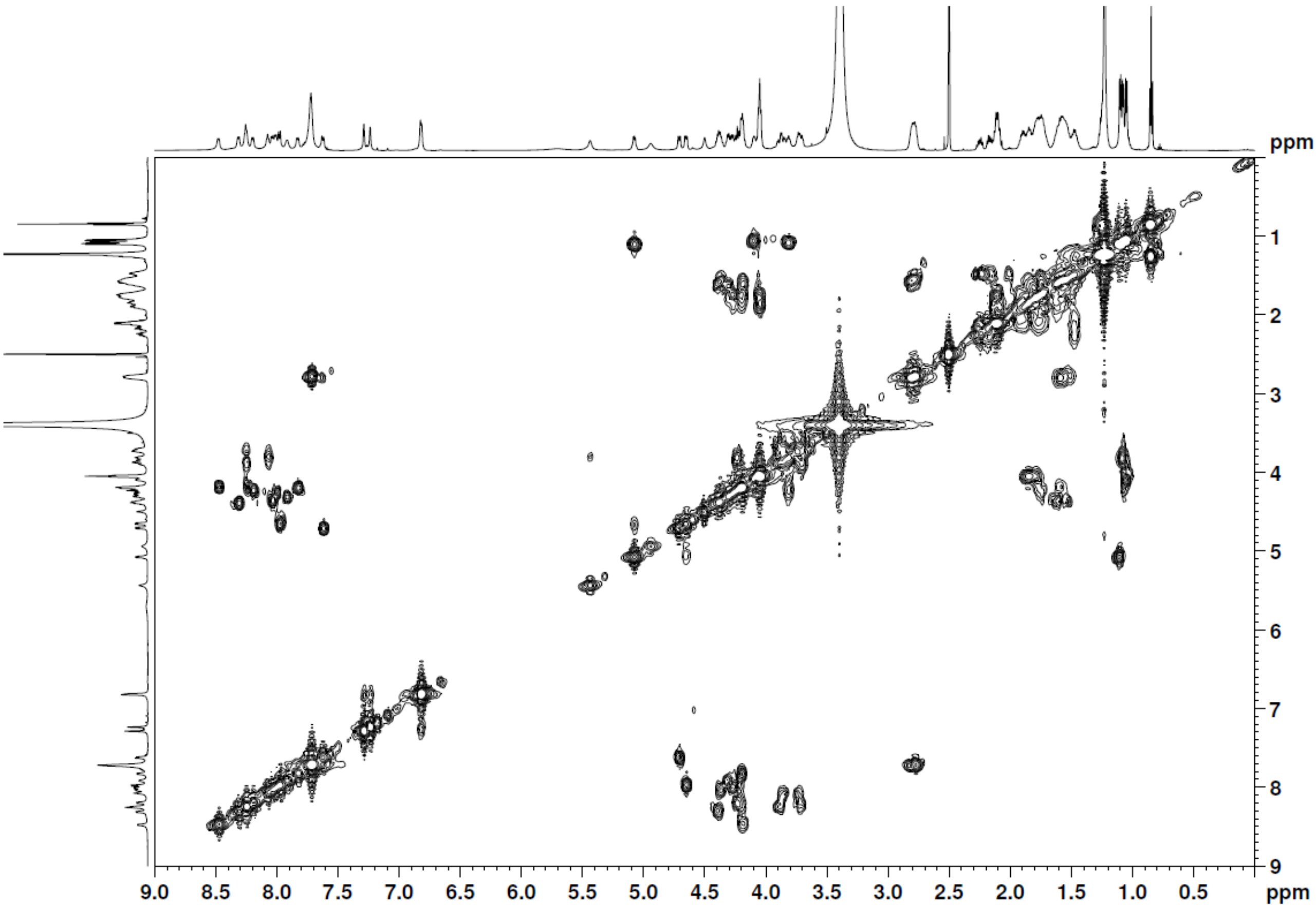

Figure S71. ^{1}H-^{1}H-COSY spectrum of pandorachelin B (**4**) in DMSO-d_6 (600 MHz).

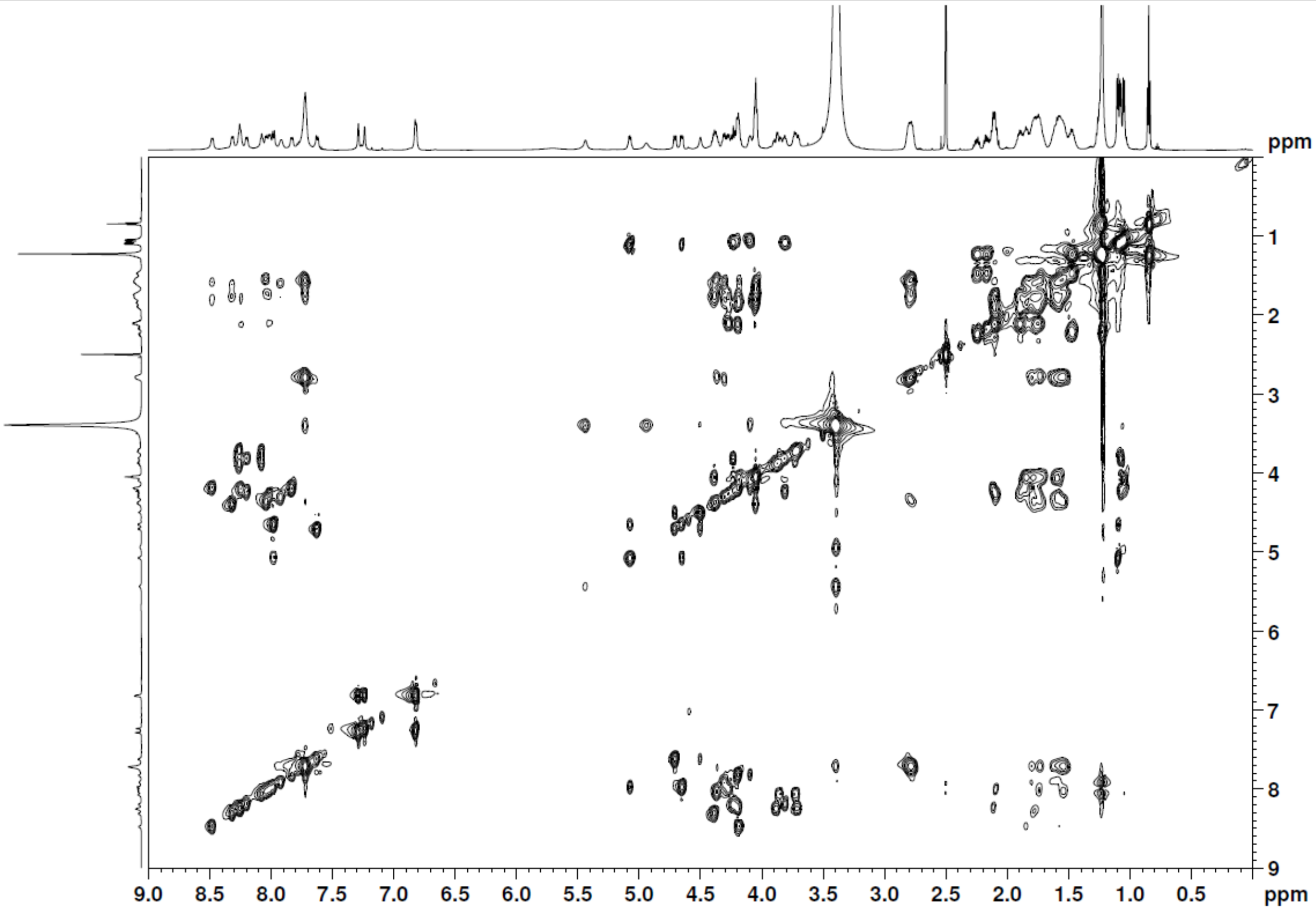

Figure S72. ^{1}H-^{1}H-TOCSY spectrum of pandorachelin B (**4**) in DMSO-d_6 (600 MHz).

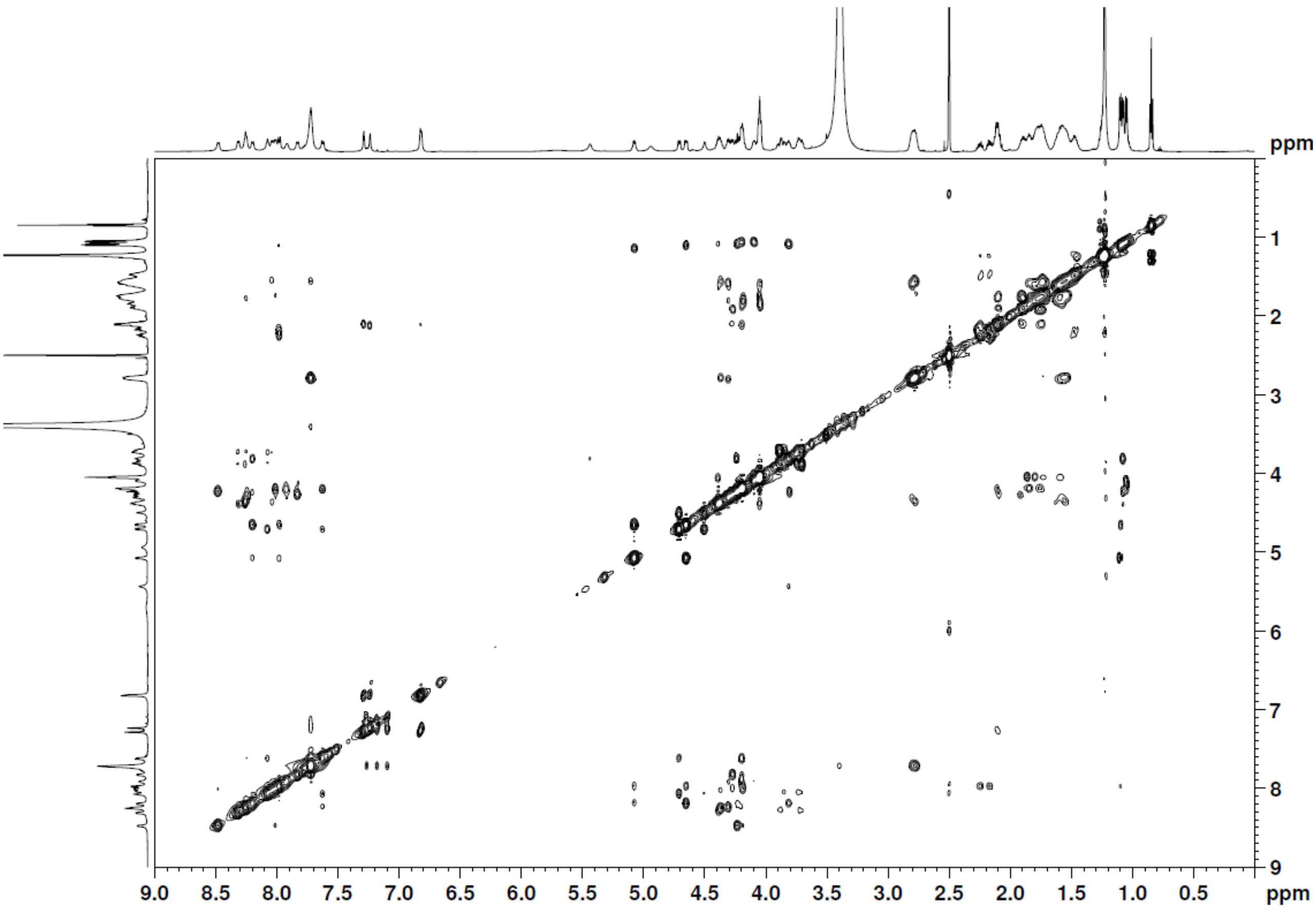

Figure S73. ^{1}H-^{1}H-ROESY spectrum of pandorachelin B (**4**) in DMSO-d_6 (600 MHz).

References

[1] N. Oberg, R. Zallot, J. A. Gerlt, *J. Mol. Biol.* **2023**, *435*, 168018.
[2] R. Zallot, N. Oberg, J. A. Gerlt, *Biochemistry* **2019**, *58*, 4169.
[3] K. Blin, S. Shaw, L. Vader, J. Szenei, Z. L. Reitz, H. E. Augustijn, J. D. D. Cediel-Becerra, V. de Crecy-Lagard, R. A. Koetsier, S. E. Williams, P. Cruz-Morales, S. Wongwas, A. E. Segurado Luchsinger, F. Biermann, A. Korenskaia, M. M. Zdouc, D. Meijer, B. R. Terlouw, J. J. J. van der Hooft, N. Ziemert, E. J. N. Helfrich, J. Masschelein, C. Corre, M. G. Chevrette, G. P. van Wezel, M. H. Medema, T. Weber, *Nucleic Acids Res.* **2025**, *53*, W32.
[4] S. F. Altschul, W. Gish, W. Miller, E. W. Myers, D. J. Lipman, *J. Mol. Biol.* **1990**, *215*, 403.
[5] B. O. Bachmann, J. Ravel, *Methods Enzymol.* **2009**, *458*, 181.
[6] L. J. Liermann, A. S. Barnes, B. E. Kalinowski, X. Zhou, S. L. Brantley, *Chem. Geol.* **2000**, *171*, 1.
[7] R. Hermenau, J. L. Mehl, K. Ishida, B. Dose, S. J. Pidot, T. P. Stinear, C. Hertweck, *Angew. Chem. Int. Ed.* **2019**, *58*, 13024.
[8] R. Hermenau, J. L. Mehl, K. Ishida, B. Dose, S. J. Pidot, T. P. Stinear, C. Hertweck, *Angew. Chem.* **2019**, *131*, 13158.
[9] A. S. Griffin, S. A. West, A. Buckling, *Nature* **2004**, *430*, 1024.
[10] C. Jenul, S. Sieber, C. Daeppen, A. Mathew, M. Lardi, G. Pessi, D. Hoepfner, M. Neuburger, A. Linden, K. Gademann, L. Eberl, *Nat. Commun.* **2018**, *9*, 1297.
[11] B. C. Louden, D. Haarmann, A. M. Lynne, *J. Microbiol. Biol. Educ.* **2011**, *12*, 51.
[12] B. Schwyn, J. B. Neilands, *Anal. Biochem.* **1987**, *160*, 47.
[13] K. Miyazaki, *BioTechniques* **2015**, *58*, 86.
[14] K. Fujii, Y. Ikai, T. Mayumi, H. Oka, M. Suzuki, K.-i. Harada, *Anal. Chem.* **1997**, *69*, 3346.
[15] S. Kuraku, C. M. Zmasek, O. Nishimura, K. Katoh, *Nucleic Acids Res.* **2013**, *41*, W22.
[16] K. Katoh, J. Rozewicki, K. D. Yamada, *Brief. Bioinform.* **2019**, *20*, 1160.
[17] X. Robert, P. Gouet, *Nucleic Acids Res.* **2014**, *42*, W320.
[18] J. Wang, F. Chitsaz, M. K. Derbyshire, N. R. Gonzales, M. Gwadz, S. Lu, G. H. Marchler, J. S. Song, N. Thanki, R. A. Yamashita, M. Yang, D. Zhang, C. Zheng, C. J. Lanczycki, A. Marchler-Bauer, *Nucleic Acids Res.* **2023**, *51*, D384.
[19] F. Teufel, J. J. Almagro Armenteros, A. R. Johansen, M. H. Gislason, S. I. Pihl, K. D. Tsirigos, O. Winther, S. Brunak, G. von Heijne, H. Nielsen, *Nat. Biotechnol.* **2022**, *40*, 1023.
[20] K. D. Clevenger, R. Wu, J. A. Er, D. Liu, W. Fast, *ACS Chem. Biol.* **2013**, *8*, 2192.
[21] L. T. Nguyen, H. A. Schmidt, A. von Haeseler, B. Q. Minh, *Mol. Biol. Evol.* **2015**, *32*, 268.
[22] S. Kalyaanamoorthy, B. Q. Minh, T. K. F. Wong, A. von Haeseler, L. S. Jermiin, *Nat. Methods* **2017**, *14*, 587.
[23] B. Q. Minh, M. A. Nguyen, A. von Haeseler, *Mol. Biol. Evol.* **2013**, *30*, 1188.
[24] S. Kumar, G. Stecher, K. Tamura, *Mol. Biol. Evol.* **2016**, *33*, 1870.
[25] A. Shevchenko, M. Wilm, O. Vorm, M. Mann, *Anal. Chem.* **1996**, *68*, 850.
